# Drimane-Pyrone-Type Meroterpenoids and Co-Isolated Compounds from the Deep-Sea Fungus *Penicillium rubens* MABC05 and Their Anti-Ferroptotic and Cytotoxic Activities

**DOI:** 10.3390/md24090326

**Published:** 2026-09-17

**Authors:** Lingyan Liu, Xinjia Yang, Caixia Hu, Ping Wang, Zhuhua Luo, Qi Lv, Yan Zhang, Zhongbin Cheng

**Affiliations:** 1 Key Laboratory of Tropical Biological Resources of Ministry of Education, School of Pharmaceutical Sciences, Hainan University, Haikou 570228, China; 2 Key Laboratory of Marine Biogenetic Resources, Third Institute of Oceanography, Ministry of Natural Resources, Xiamen 361005, China

**Keywords:** *Penicillium rubens*, drimane-pyrone-type meroterpenoids, tetrahydroxanthone-ergosterol hybrid, ferroptosis, cytotoxicity

## Abstract

Ferroptosis is a critical driver of tubular necrosis, and its effective inhibition holds significant potential for mitigating renal injury. In this study, chemical investigation of the strain *Penicillium rubens* MABC05 yielded nine drimane-pyrone-type meroterpenoids (**1**–**9**), including six previously undescribed analogues, a previously undescribed tetrahydroxanthone-ergosterol hybrid (**10**), four steroids (**11**–**14**), and three xanthone derivatives (**15**–**17**). Their structures were elucidated via comprehensive spectroscopic analysis, supported by single-crystal X-ray diffraction (Mo K*α* for **1** and **3**, and Cu K*α* for **6**) and ^13^C NMR calculations (**2** and **10**). The absolute configurations of **1**–**5** and **10** were assigned by ECD calculation. Compounds **1**–**6** represent a rare group of meroterpenoids containing a fused drimane-type sesquiterpene and pyrone skeleton. Additionally, two storage-induced oxidative artifacts of **10** were also characterized. These compounds were evaluated for anti-ferroptotic and cytotoxic activities. Compound **15** exhibited inhibition against RSL3-induced ferroptosis in human renal proximal tubular epithelial cells with an EC_50_ value of 12.8 μM; it effectively reduced MDA levels, elevated GSH content, and suppressed lipid radical generation. Compounds **11** and **16** exhibited cytotoxicity against MCF-7 cells with IC_50_ values of 4.3 and 8.4 μM, respectively. Compound **15** represents a promising chemotype for ferroptosis inhibitors with potential for mitigating renal tubular injury.

## 1. Introduction

The talarolutins and penicillipyrones constitute a small group of meroterpenoids (drimane-pyrone-type meroterpenoids), featuring a drimane-type sesquiterpene fused with a 2-methyl-pyrone (or its hydrogenated derivative) moiety [1,2]. The proposed biosynthesis of these meroterpenoids is initiated by the ionization of farnesyl diphosphate (FPP), generating an allylic carbocation that attacks the electron-rich pyrone ring to afford the C-farnesylated intermediate. Subsequent epoxidation, ring-opening, and cyclization (forming either 6/6 or 6/5 bicyclic systems) proceed on the sesquiterpenoid framework. Notably, a key carbocation rearrangement occurs during the cyclization stage for the 6/5 bicyclic systems, leading to a distinct carbon-carbon connectivity between the pyrone ring and the sesquiterpene unit (penicillipyrones). Moreover, the enol tautomerism of the 4-hydroxy-2-pyrone core and the subsequent intramolecular nucleophilic addition of the enolic hydroxyl to the sesquiterpene carbocation dictate the final carbonyl orientation in the pyrone moiety. The structural variety of drimane-pyrone-type meroterpenoids arises from oxidation of the ring bearing the gem-dimethyl group to generate alcohol, ketone, or alkene functionalities, or from the saturation of the methyl-substituted double bond in the pyrone ring. To date, only six members, talarolutins A–D and penicillipyrones A–B, have been reported from the endophytic *Talaromyces minioluteus* and a marine *Penicillium* sp., respectively, and penicillipyrone B elicited significant induction of quinone reductase.

Although *Penicillium notatum* (a historical synonym of *P. rubens*) has long been famous for penicillin production, the systematic chemical investigation of *P*. *rubens* as a source of diverse metabolites has only begun in recent years [3,4,5,6,7,8,9,10,11], leading to the identification of the common polyketide citrinin [4], new meroterpenoids with an oxygenated seudenone core and isoprenyl unit containing side chain [3,6,9], new drimane-seudenone-type meroterpenoids [3,7], new amino-bis-tetrahydrofuran derivatives [6], rare steroids bearing a 6/6/6/6/5 pentacyclic system [10], a new linear sesquiterpenoid [9], and a novel polyketide with bicyclo[2.2.2]undecane skeleton [7].

**Figure 1:**
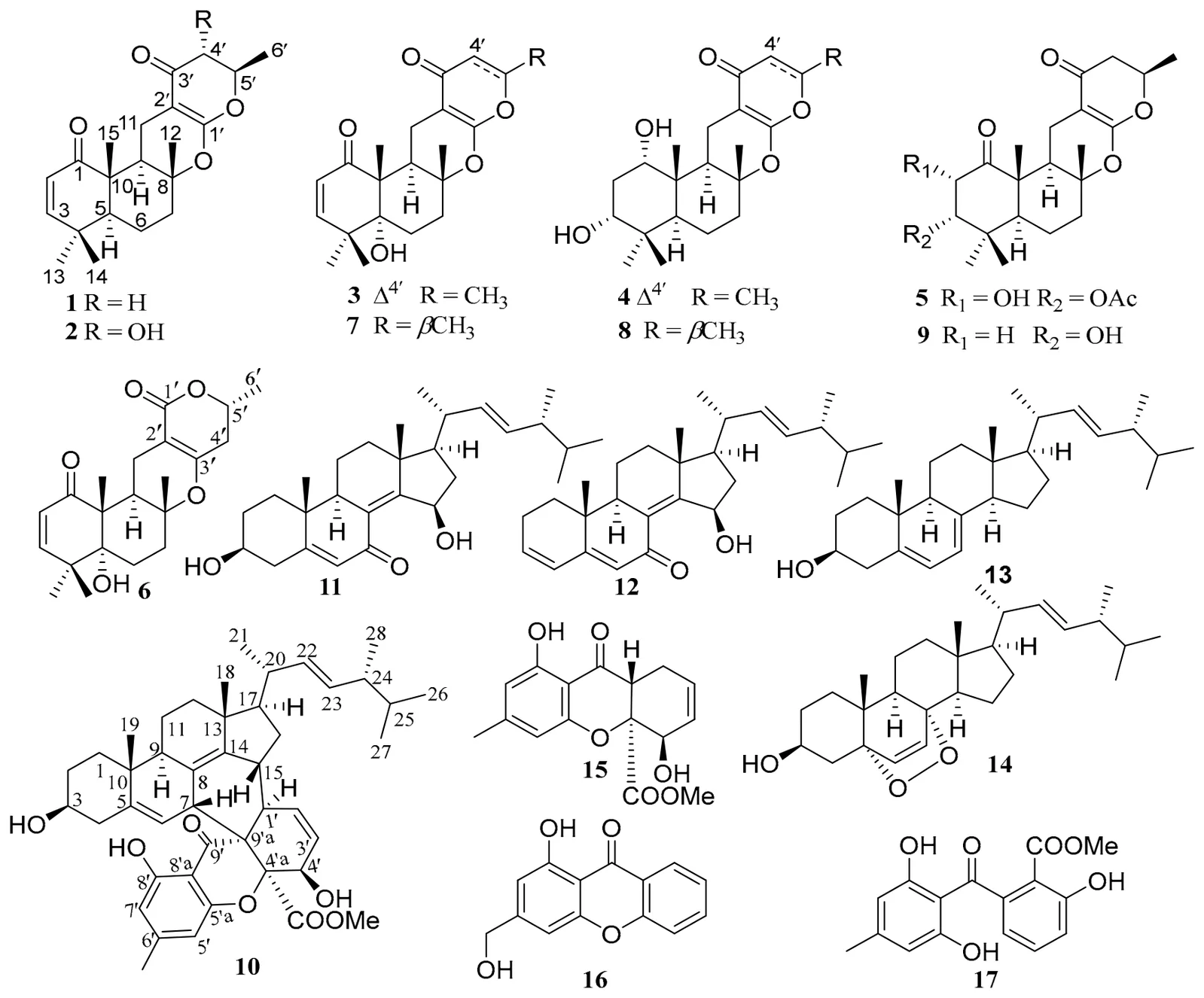
Structures of compounds **1**–**17** from the fungus *Penicillium rubens* MABC05.

As part of our ongoing program to discover bioactive molecules from marine-derived fungi [12,13,14,15], ^1^H NMR analysis of the EtOAc extract of *Penicillium rubens* MABC05 revealed talarolutin D (**7**) as its major constituent (Figure S1). Despite the fact that drimane-pyrone meroterpenoids possess novel scaffolds and promising pharmacological potential, the limited number of congeners and the lack of comprehensive bioactivity evaluations have impeded the assessment of their therapeutic prospects. Therefore, we conducted a chemical investigation of the metabolites of this strain using a ^1^H NMR-guided isolation strategy, with a focus on the drimane-pyrone meroterpenoids. Specifically, the isolation was directed by monitoring the characteristic ^1^H NMR signals of the drimane-pyrone skeleton, including the four tertiary methyl singlets (δ_H_ 0.80–1.50) and the distinctive pyrone methyl doublet at ca. δ_H_ 1.45 or olefinic methyl singlet at ca. δ_H_ 2.20 in the subfractions. As a result, 17 compounds, including six previously undescribed (**1**–**6**) and three known (**7**–**9**) drimane-pyrone meroterpenoids, a previously undescribed tetrahydroxanthone–ergosterol hybrid (**10**), four known sterols, and three xanthone derivatives, were obtained (Figure 1). It should be stated that a SciFinder search indicated that compounds sharing the same planar structures as **1** and **4**–**6** are commercially available. However, the complete absence of any literature reports (and thus any physical or spectroscopic data) and unassigned relative and absolute configurations precluded their unambiguous identification. Therefore, compounds **1** and **4**–**6** are reported as new compounds in this study, with their relative and absolute configurations established here for the first time. Herein, the isolation, structural elucidation, and bioactivity evaluation of these compounds were described.

## 2. Results and Discussion

### 2.1. Structural Elucidation

**Figure 2:**
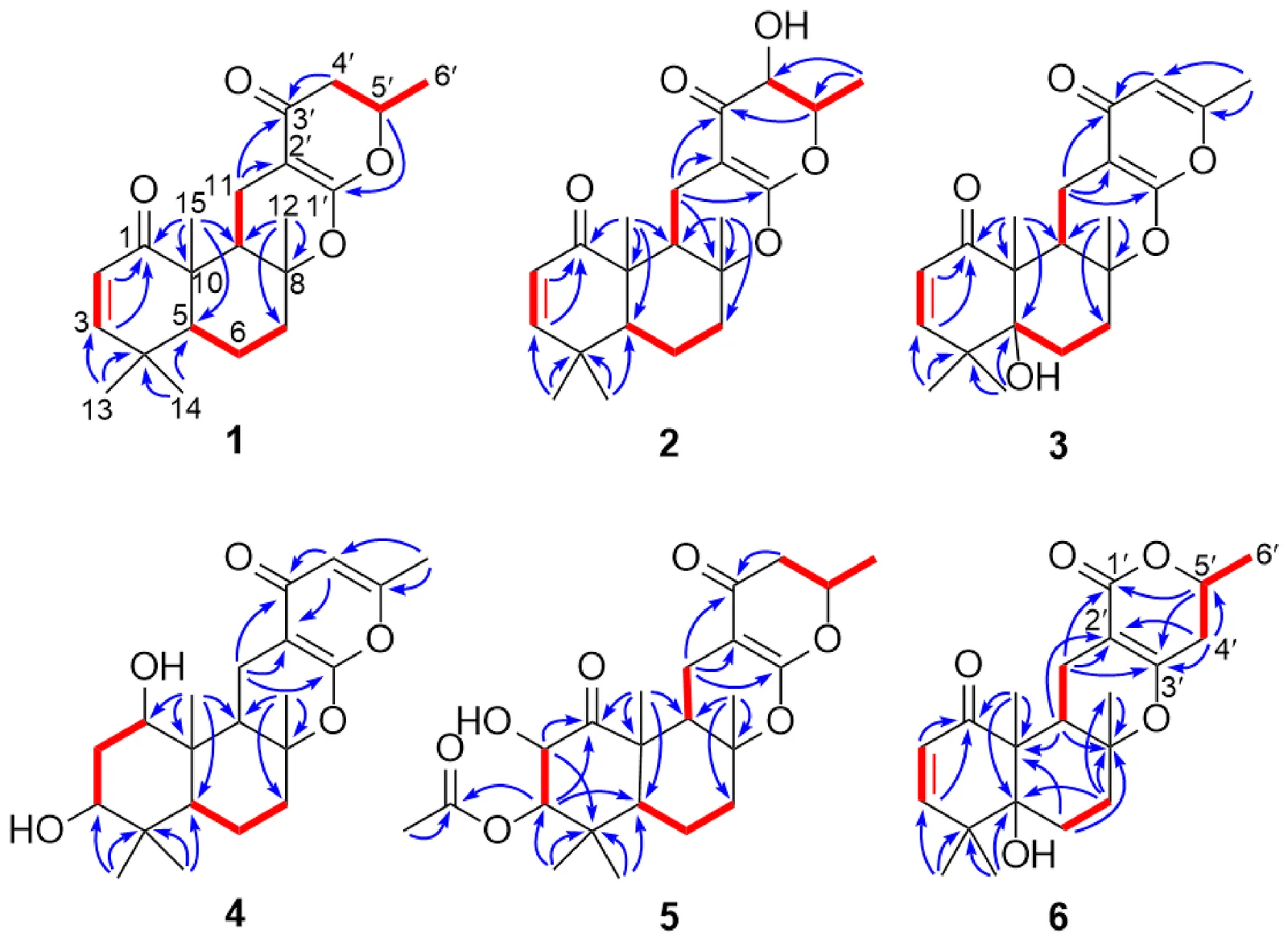
Key COSY (▬) and HMBC (→) correlations of **1**–**6**.

**Figure 3:**
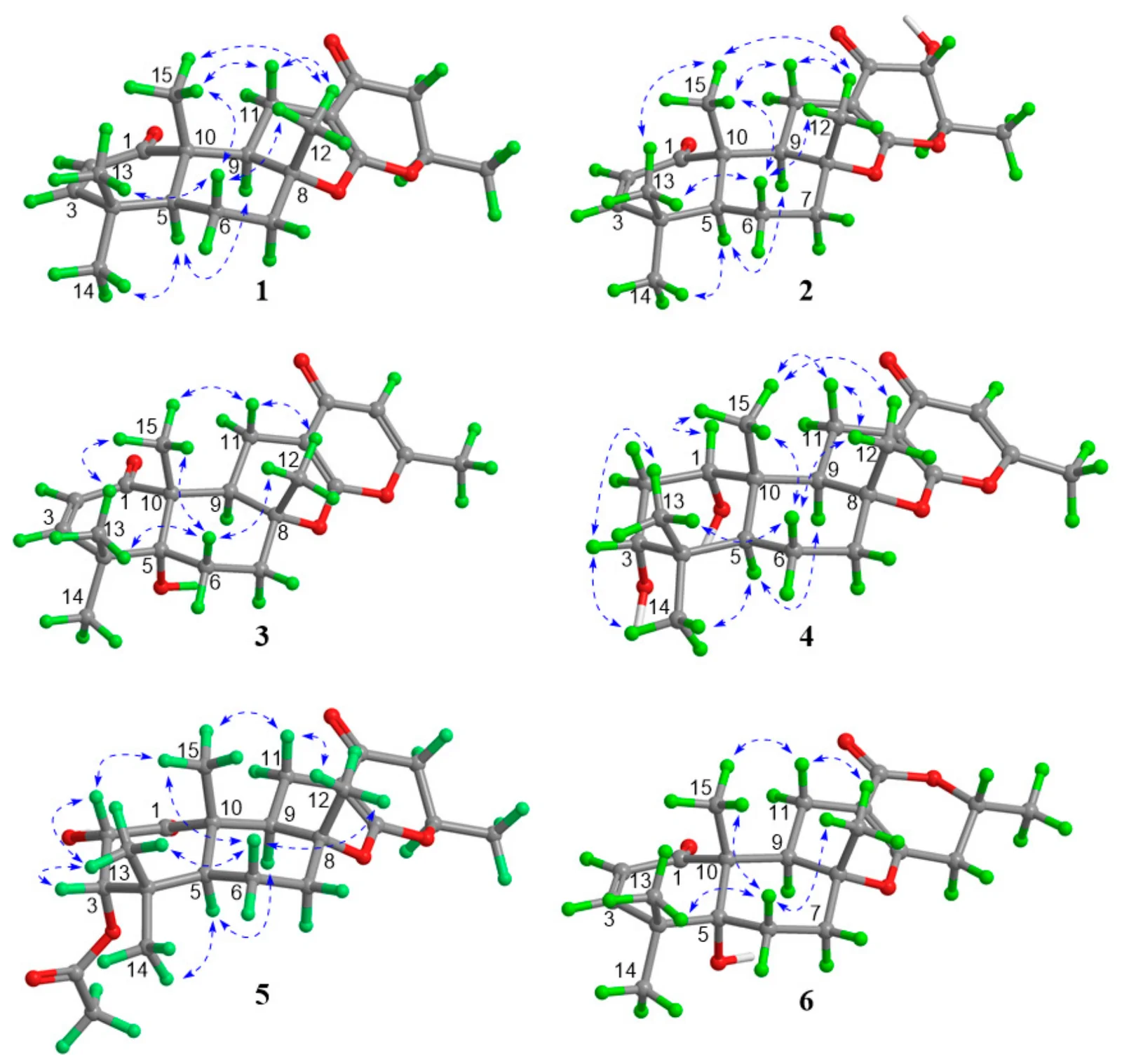
Key NOESY correlations (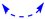) of **1**–**6**.

**Figure 4:**
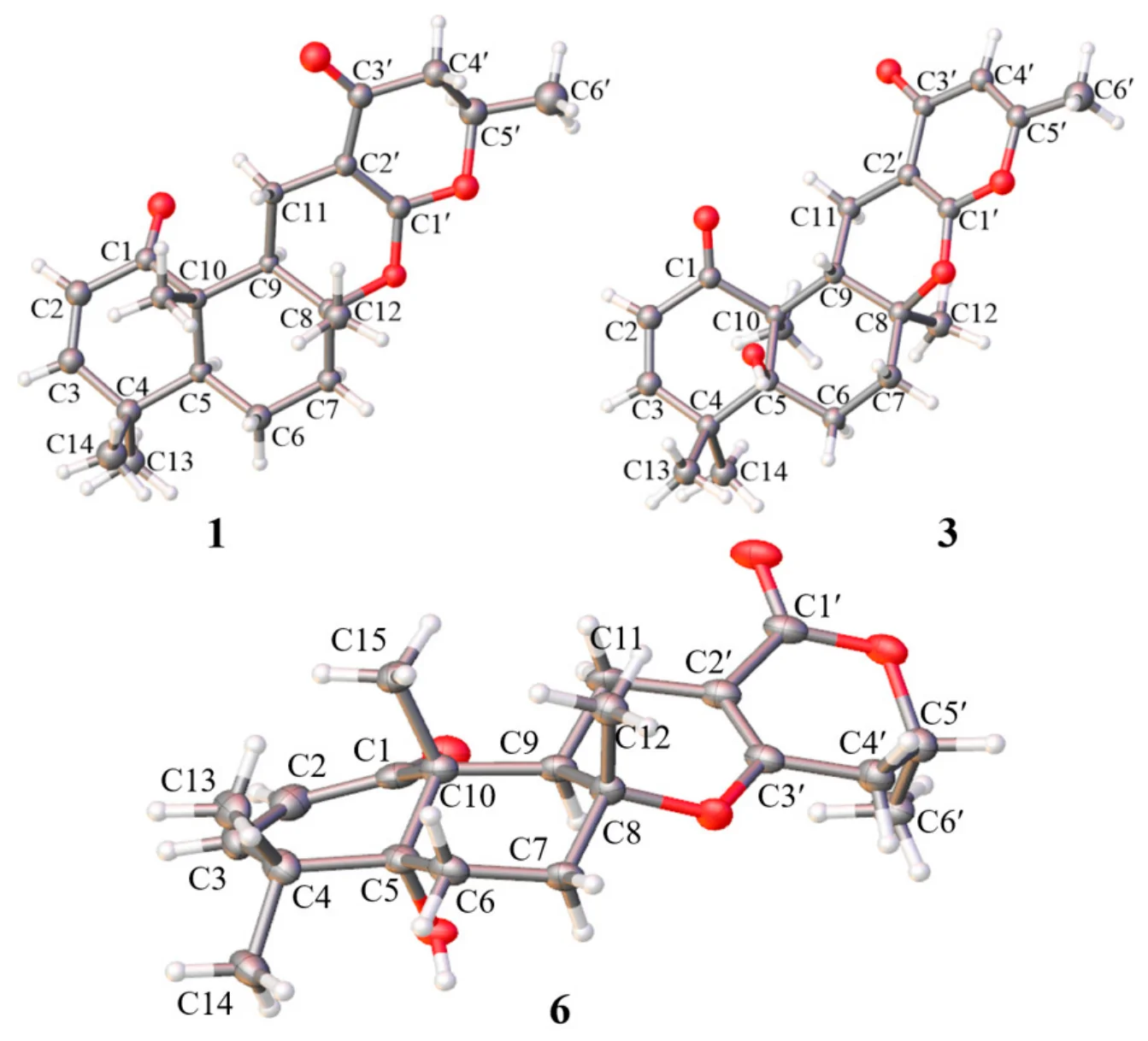
X-ray crystal structures of compounds **1** and **3** determined using Mo K*α* radiation, and compound **6** determined using Cu K*α* radiation.

The molecular formula of penirubenoid A (**1**) was determined to be C_21_H_28_O_4_ by HRESIMS ion at *m*/*z* 345.2054 [M + H]^+^ (calcd. 345.2066), accounting for eight degrees of unsaturation. The ^1^H NMR spectrum exhibited resonances attributable to four methyl singlets (δ_H_ 1.28, 1.18, 1.082, 1.079), one secondary methyl doublet (δ_H_ 1.43, d, *J* = 6.3 Hz), an oxymethine proton (δ_H_ 4.57, ddq, *J* = 13.9, 6.3, 3.2 Hz), and a *cis*-disubstituted double bond [δ_H_ 6.33 (d, *J* = 10.2 Hz) and 5.70 (d, *J* = 10.2 Hz)], together with multiple aliphatic signals (Table 1). The ^13^C NMR data, assisted by HSQC, resolved 21 carbon signals, which were classified as five methyls (δ_C_ 31.0, 20.4, 21.7, 20.3, 14.9), four sp^3^ methylenes (δ_C_ 42.6, 38.9, 19.8, 17.6), three sp^3^ methines (δ_C_ 75.5, 50.8, 43.7) including one oxygenated methine, three sp^3^ quaternary carbons (δ_C_ 83.1, 47.3, 36.2), two carbonyls (δ_C_ 204.3, 191.0), and two olefinic moieties [*δ*_C_ 154.7/123.9 and 168.1/91.1]. Since the two carbonyls and two double bonds accounted for four of the eight degrees of unsaturation, the remaining four required a tetracyclic framework for compound **1**. These data revealed the structural features for a talarolutin-type meroterpenoid, closely related to the co-isolated known analogue talarolutin D (**7**). The key structural difference was the presence of an additional methine (δ_H_ 1.66; δ_C_ 50.8) in **1** instead of the oxygenated carbon at δ_C_ 78.4 (C-5) observed in **7**, implying replacement of the hydroxy group by a hydrogen atom. This deduction was supported by the COSY correlation between H-5 (δ_H_ 1.66) and H_2_-6 (δ_H_ 1.79, 1.49), and further corroborated by HMBC cross-peaks from the gem-dimethyl protons (δ_H_ 1.079 and 1.082) to C-5 (δ_C_ 50.8) (Figure 2). The gross structure was determined as depicted. The NOESY correlations (Figure 3) from H-11β (δ_H_ 2.00) to H_3_-15 (δ_H_ 1.18) and H_3_-12 (δ_H_ 1.28) and from H-5 (δ_H_ 1.66) to H-9 (δ_H_ 1.90) indicated H_3_-15 and H_3_-12 were in the same orientation, while H-5 and H-9 were in the opposite direction. Thus, the relative configurations at C-5, C-8, C-9, and C-10 were successfully assigned, while the configuration at C-5′ could not be established from NOESY data, as this stereocenter was too remote from the others to yield diagnostic correlations. Nevertheless, the NMR data of the pyrone moiety were almost identical to those of talarolutins A–D (the structure of talarolutin A was confirmed by single-crystal X-ray diffraction analysis) [1], suggesting that C-5′ possessed the same relative configuration as that of talarolutins A–D. This assignment was ultimately confirmed by single-crystal X-ray diffraction analysis of **1** using Mo K*α* radiation (Figure 4).

**Figure 5:**
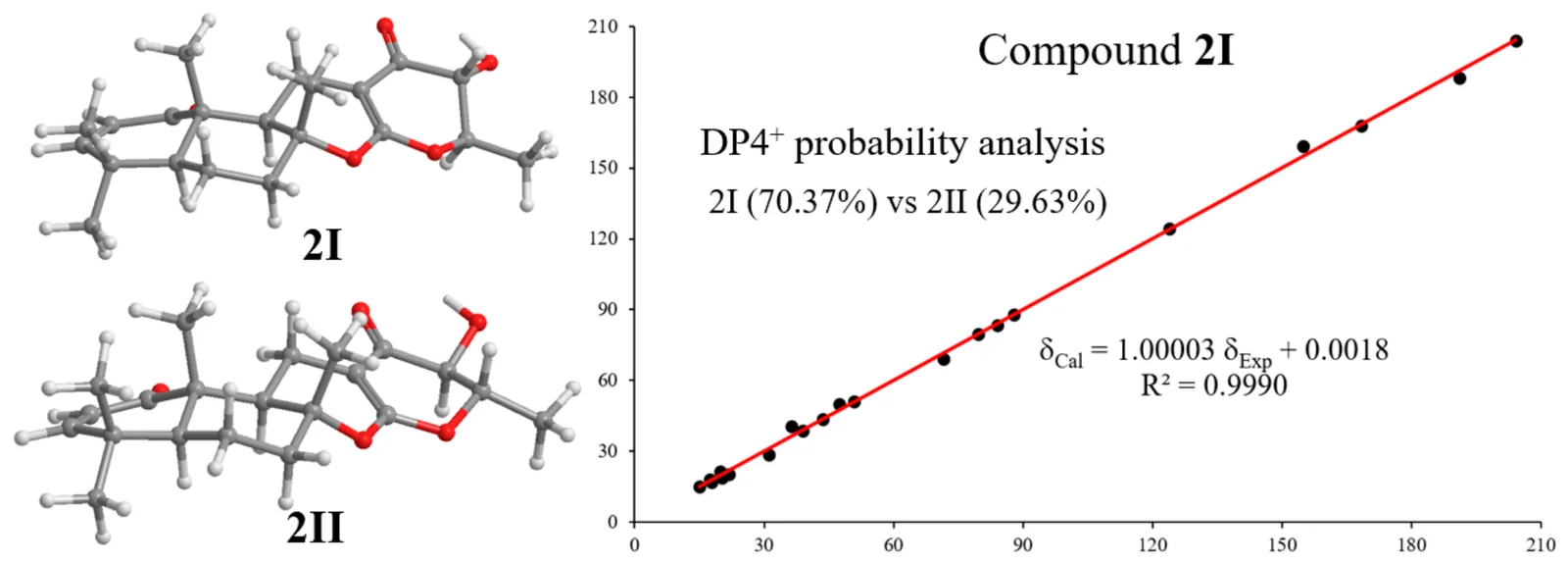
Two plausible structural candidates (**2I** and **2II**) and regression analysis of experimental and calculated ^13^C NMR chemical shifts (ppm) of **2I**.

The molecular formula of compound **2** was established as C_21_H_28_O_5_ by HRESIMS. Its NMR data were very similar to those of **1**, with obvious differences being the presence of an additional methine (δ_H_ 3.86, d, *J* = 12.2 Hz; δ_C_ 71.6) and the upfield-shifted chemical shifts in H-5′. These spectral distinctions suggested that **2** is a hydroxylated derivative of **1**, with the hydroxy group most plausibly located at C-4′. This structural assignment was corroborated by the HMBC correlations (Figure 2) from H_3_-6′ to the methine carbons C-5′ and C-4′, and was further substantiated by the H-4′/H-5′/H_3_-6′ spin system established by the COSY spectrum. The relative configuration of the drimane moiety was determined to be the same as that of **1** by comparison of the NMR data, and was confirmed by NOESY correlations (Figure 3). The coupling constant *J*_H-4′/H-5′_ (12.2 Hz) was indicative of the *trans*-relationship of H-4′ and H-5′ in an axial orientation. Similar to **1**, the relative configuration of C-4′ and C-5′ relative to the drimane moiety could not be assigned from the NOESY data, leaving two plausible structural candidates (Figure 5 and Table S1). Subsequent DP4^+^ probability analysis of the ^13^C NMR data supported **2I** as the correct isomer (Figure 5 and Table S2), a structural assignment also consistent with biogenetic considerations.

Penirubenoid C (**3**) had the molecular formula of C_21_H_26_O_5_ as established by HRESIMS, indicating one more degree of unsaturation than **7**. Its NMR data were closely related to those of **7**, with a key difference being the replacement of the original methyl doublet (δ_H_ 1.44; δ_C_ 20.4) and the CH_2_-4′–CH-5′ (δ_H_ 4.60, 2.49, 2.36; δ_C_ 75.6, 42.6) by one olefinic methyl (δ_H_ 2.20; δ_C_ 19.2) and one additional double bond (δ_H_ 6.0; δ_C_ 112.0, 160.4). This suggested that the 2,3-dihydro-2-methyl-4-pyrone unit in **7** was converted into a 2-methyl-γ-pyrone moiety. The deduction was evidenced by the HMBC correlation from the olefinic methyl protons (δ_H_ 2.20) to the new olefinic carbons (δ_C_ 112.0, 160.4). The relative configuration of **3** was determined to be the same as that of **7** by NOESY analysis (Figure 3) and confirmed by single-crystal X-ray crystallography (Figure 4).

Penirubenoid D (**4**) was assigned the molecular formula C_21_H_30_O_5_ by HRESIMS. Comparison of the 1D NMR data showed that the differences between **4** and **8** mirrored those between **3** and **7**, indicating a 2-methyl-γ-pyrone moiety, which was confirmed by HMBC correlations from the olefinic methyl (δ_H_ 2.21) to C-4′ (δ_C_ 111.4) and C-5′ (δ_C_ 160.7). The relative configuration of **4** was determined by NOESY analysis (Figure 3).

The molecular formula of **5** was determined to be C_23_H_32_O_7_ by HRESIMS, accounting for seven degrees of unsaturation. The ^1^H and ^13^C NMR data of **5** closely resembled those of **1**, with the most notable differences being the presence of two additional oxygenated methines (δ_H_ 5.31, 4.96; δ_C_ 83.4, 68.8) and an acetoxy group (δ_H_ 2.02; δ_C_ 20.7, 170.0) instead of the double bond Δ^2^ in **1**. The structure of **5** was subsequently established by detailed 2D NMR analysis (Figure 2 and Figure 3). In particular, the ^1^H-^1^H COSY cross-peaks between the two oxymethine protons H-2 (δ_H_ 4.96) and H-3 (δ_H_ 5.31), together with the HMBC correlations from both H-2 and H-3 to the carbonyl carbon C-1 (δ_C_ 210.3) and from H-3 to the acetoxy carbonyl carbon (δ_C_ 170.0), assigned the hydroxy group to C-2 and the acetoxy group to C-3. The NOESY correlations from H-2 to H_3_-15 (δ_H_ 1.30) and H_3_-13 (δ_H_ 1.24) and from H-5 (δ_H_ 1.73) to H-9 (δ_H_ 2.15) and from H-11β (δ_H_ 1.97) to H_3_-12 (δ_H_ 1.298) and H_3_-15 (δ_H_ 1.305) revealed that the two cyclohexane rings were *trans*-fused and adopted chair-chair conformations. These data also indicated that H-2 and the methyl groups CH_3_-12, CH_3_-13 and CH_3_-15 were β-axial oriented, whereas H-5 and H-9 were in α-axial orientation. The coupling constant of *J*_H-2–H-3_ (4.0 Hz) reflected an axial–equatorial relationship of H-2 and H-3. A comparison of the NMR data of the dihydropyran-4-one moiety in compounds **1** and **5** indicated that C-5′ in **5** possessed the same relative configuration as that in **1**. Thus, the structure of **5** was determined as depicted.

Compound **6** had a molecular formula of C_21_H_28_O_5_ as determined by the HRESIMS, the same as that of **7**. The ^1^H NMR data of **6** were nearly identical to **7**, except for the splitting pattern of the methylene protons H_2_-11 and H_2_-4′. In **6**, the protons of both methylene groups (H_2_-11 and H_2_-4′) each displayed additional small allylic couplings (ca. 1 Hz) compared with those in **7**. This observation suggested the presence of a (CH_2_-11)–C=C–(CH_2_-4′) fragment in **6**. Further comparison of their ^13^C NMR data revealed that the carbonyl carbon of the pyrone moiety was significantly upfield shifted from 191.0 in **7** to 165.7 ppm in **6**, indicating the presence of an α-pyrone moiety in **6** (the α-pyrone moiety did not undergo the keto-enol tautomerization process to give the γ-pyrone unit) instead of the γ-pyrone unit in **7**. The HMBC correlations from H_2_-11 to C-1′, C-2′, C-3′, from H_2_-4′ to C-3′ and C-5′, and from H-5′ to C-1′ and C-3′, along with the COSY relationship of H_2_-4′/H-5′/H_3_-6′ confirmed the presence of the α-pyrone moiety and its connection with the drimane unit via C-2′ and C-3′. The relative configuration of the drimane moiety was assigned by NOESY analysis (Figure 3), while the relative configuration at C-5′ and the absolute configuration of **6** were unambiguously established by single-crystal X-ray diffraction using Cu Kα radiation [Flack parameter = 0.1(3)], which also revealed the α-axial orientation of the 6′-CH_3_ group in the solid state.

**Figure 6:**
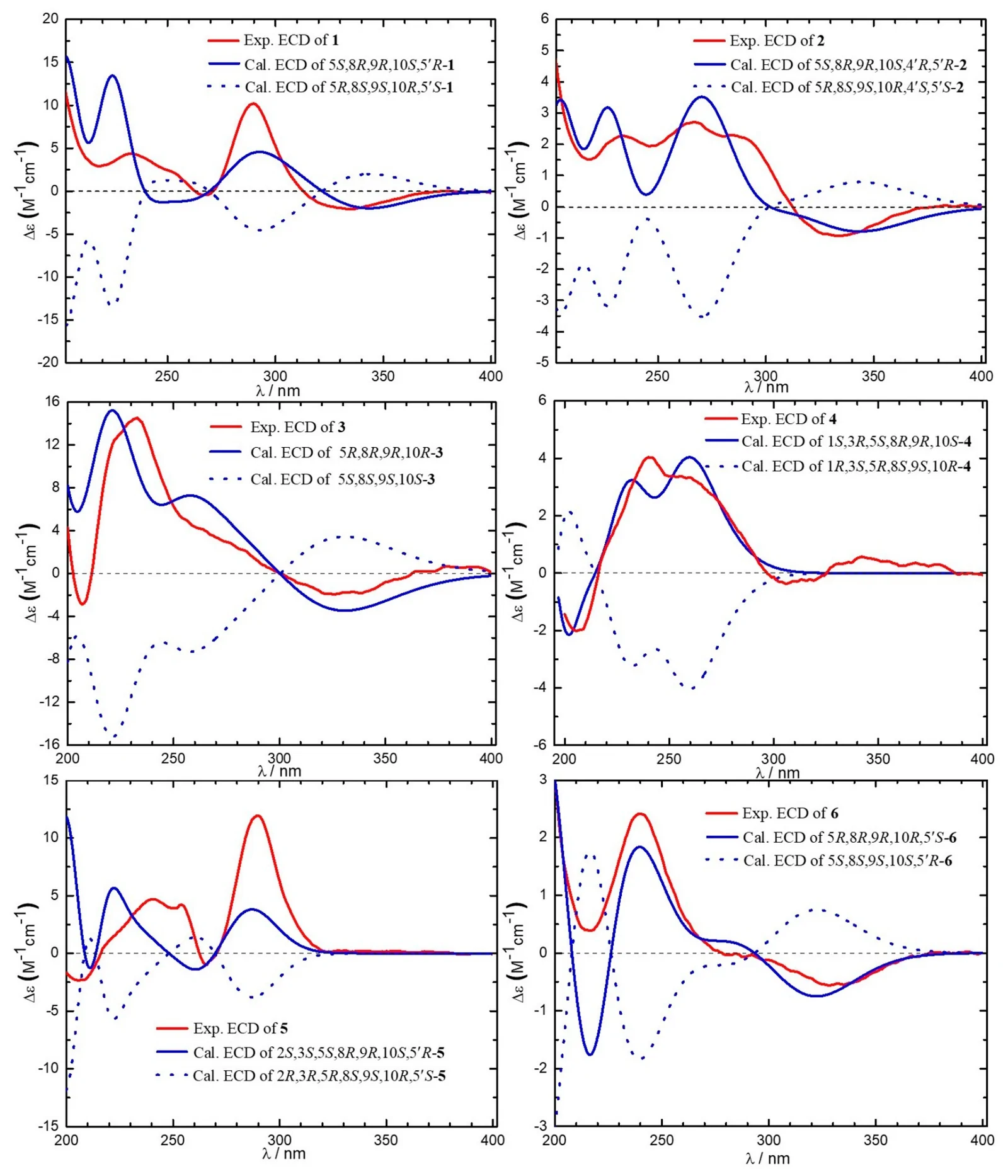
Experimental and calculated ECD spectra of **1**–**6**.

The absolute configurations of compounds **1**–**5** were assigned by comparison of their experimental and calculated ECD spectra (Figure 6). For compound **6**, the ECD calculation was also performed and the result was fully consistent with that determined by single-crystal X-ray diffraction.

**Figure 7:**
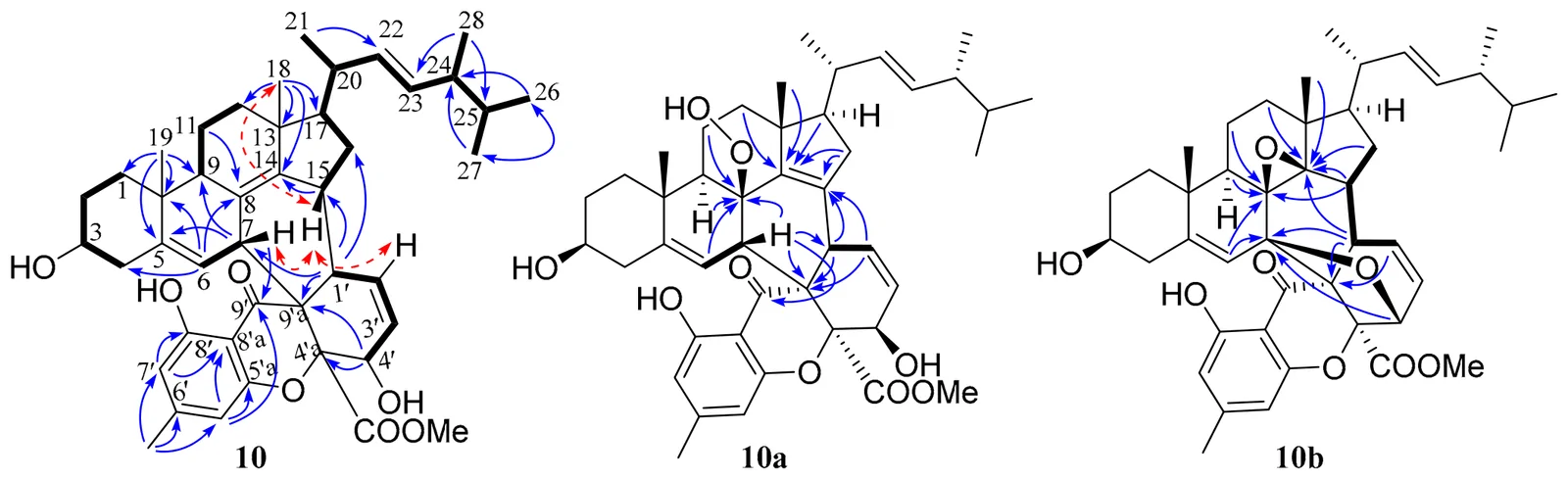
Key COSY (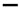), HMBC (→), NOESY (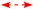) correlations of **10** and the two oxidative artifacts **10a** and **10b**.

The molecular formula of peniruxanthosterol (**10**) was assigned as C_44_H_56_O_7_ by the HRESIMS. Its structure was established as a tetrahydroxanthone–ergosterol hybrid by analysis of the NMR data (Table 2), comprising subunits related to compounds **11** and **15**. In the tetrahydroxanthone subunit, the COSY correlation (Figure 7) between the olefinic proton H-2′ (δ_H_ 5.95) and the methine H-1′ (δ_H_ 2.90), together with HMBC correlations from both H-2′ and H-1′ to the non-protonated carbon C-9′a (δ_C_ 54.9) defined a C-1′ (δ_C_ 46.1)–C-9′a fragment, which distinguished this fragment from that (a CH-1′–C-9′a unit) in **15**. For the ergosterol unit, the COSY cross-peak of H-6 (δ_H_ 5.86)/H-7 (δ_H_ 3.34) and HMBC correlations from H-7 to C-5 (δ_C_ 143.6), C-9 (δ_C_ 46.8), and C-14 (δ_C_ 149.4) indicated replacement of the carbonyl group C-7 (δ_C_ 191.1) in **11** by a methine group in **10**. In addition, the significantly upfield-shifted chemical shifts in CH-15 (δ_H_ 2.10, m; δ_C_ 41.5) suggested the absence of a hydroxy group at this position. These differences indicated that the two fragments are linked via this four-carbon junction (C-1′, C-9′a, C-7, C-15). The COSY correlation between H-15 and H-1′, along with the HMBC cross-peaks from H-1′ to C-14 (δ_C_ 149.4), C-15 (δ_C_ 41.5), and C-16 (δ_C_ 37.2), confirmed the direct linkage between CH-1′ and CH-15. Furthermore, the HMBC correlations from H-7 to C-1′, C-9′a, and C-9 established the connectivity between C-9′a and CH-7, thereby completing the assembly of the hybrid skeleton.

**Scheme 1:**
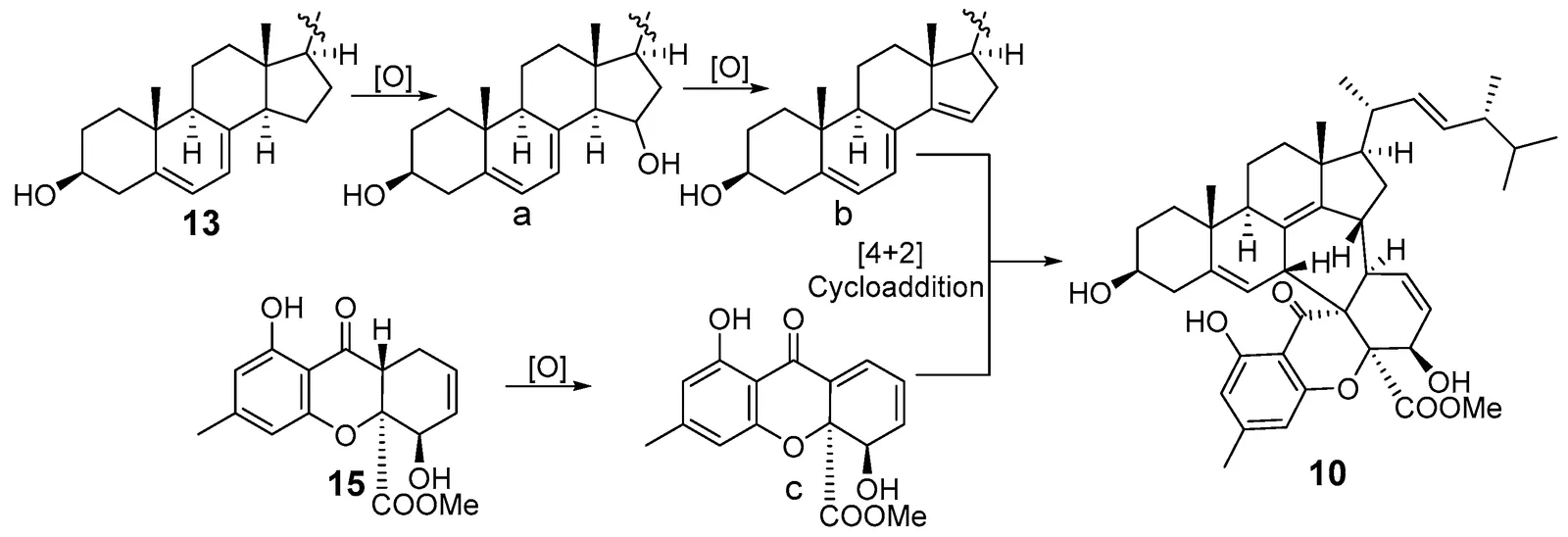
Putative pathway for the biogenesis of **10**.

It should be noted that compound **10** may be an artifact formed during the extraction and isolation procedure. Its biogenesis is proposed to proceed via the convergent assembly of ergosterol (**13**) and the tetrahydroxanthone (4*R*,4a*S*,9a*R*)-1,9a-dihydronidulalin A (**15**) (Scheme 1), analogous to the proposed biosynthesis of the rare ergosterol derivative rubensteroid from *Penicillium rubens* AS-130 [10]. On this basis, compound **13** undergoes sequential enzymatic oxidations, including regioselective hydroxylation at C-15 followed by enzymatic desaturation, to furnish the highly reactive dienophile **b** bearing the double bond Δ^14^. Concurrently, **15** is oxidatively dehydrogenated to generate the conjugated diene **c**. These highly activated intermediates undergo a stereospecific intermolecular [4 + 2] cycloaddition, constructing the bridging six-membered ring and ultimately affording the octacyclic hybrid **10**.

**Figure 8:**
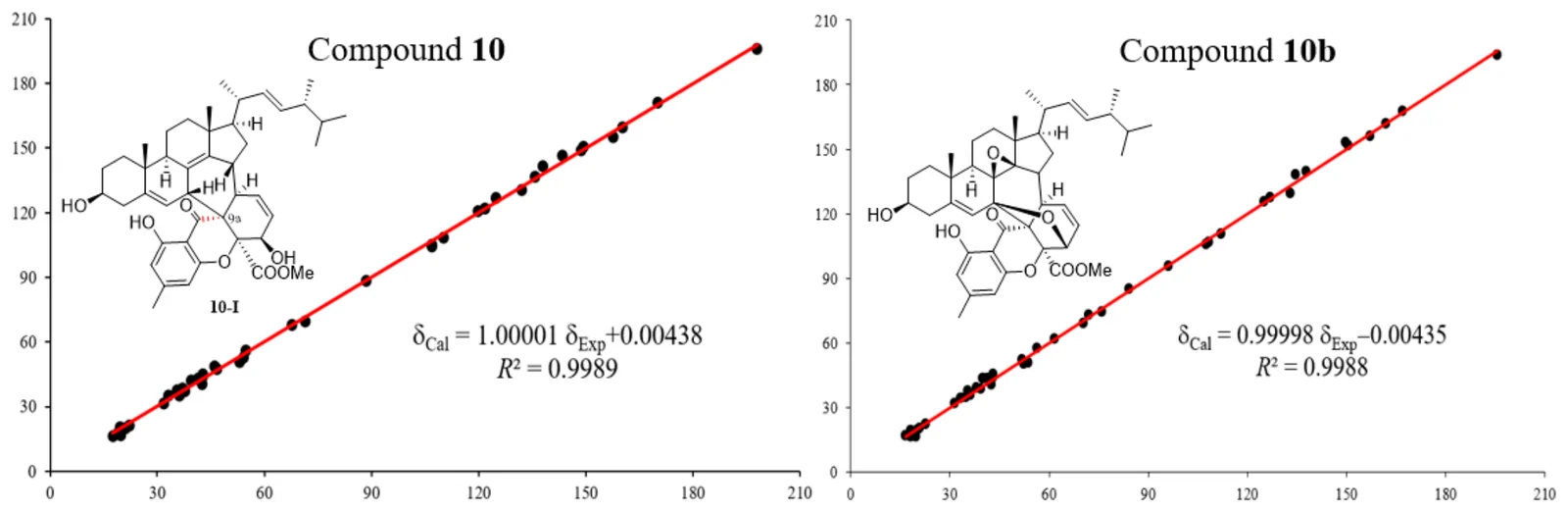
Regression analysis of experimental and calculated ^13^C NMR chemical shifts (ppm) of **10** and **10b**.

**Figure 9:**
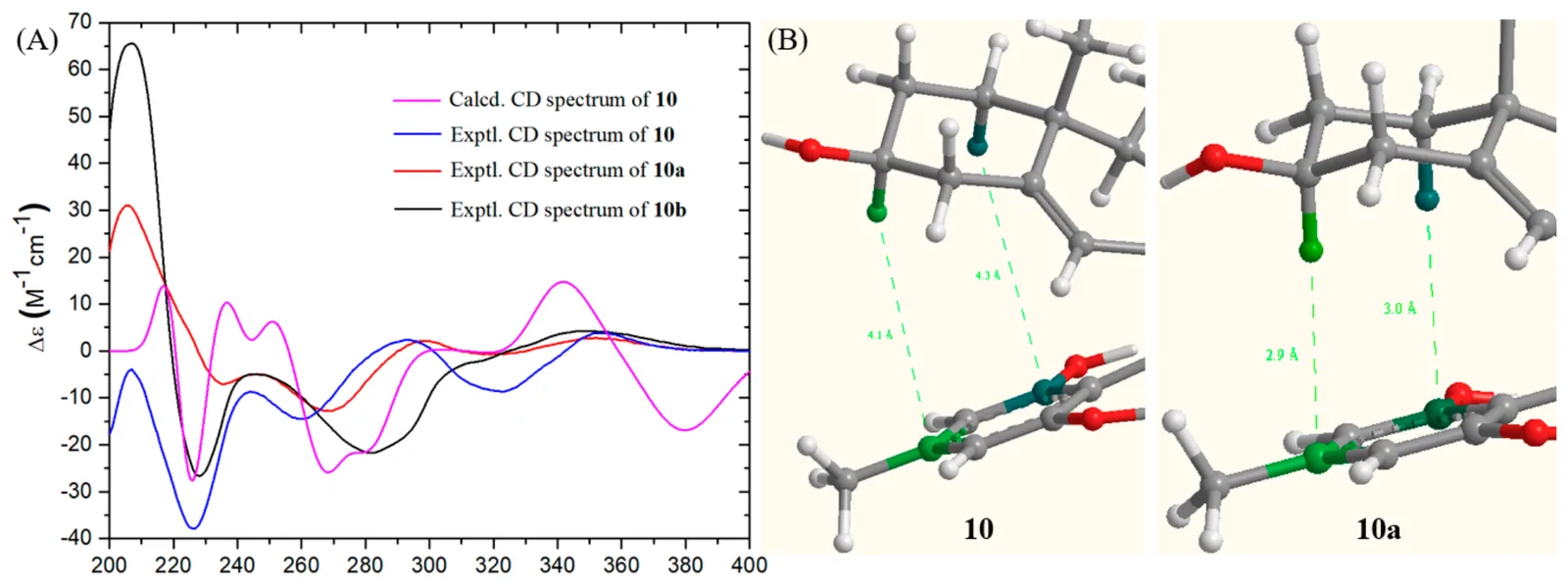
(**A**) Experimental and calculated ECD spectra of **10** and experimental ECD spectra of **10a** and **10b**. (**B**)The distances between H-1α (cyan sphere) and H-3 (green sphere) and the benzene ring of the xanthone moiety are indicated by dashed green lines. In **10**, the distances from H-3 and H-1α to the xanthone benzene ring are 4.1 Å and 4.3 Å, respectively. (**B**) In 10a, the distances are shortened to 2.9 Å and 3.0 Å, respectively.

The relative configuration of the octacyclic core in **10**, except for that at C-9′a, was established by analysis of its NOESY data (Figure 7 and Figure S48). The absolute configurations at C-20 and C-24 in the sterol side chain were proposed to be identical to those of **13** on the basis of biogenetic considerations. Particularly, the NOESY cross-peaks from H-15 (δ_H_ 2.10) to H_3_-18 (δ_H_ 0.92) and H-7 (δ_H_ 3.34) indicated that these protons are β-oriented, whereas the correlation between H-15 and H-2′ (δ_H_ 5.95) suggested that H-1′ is α-oriented. However, the configuration at C-9′a could not be determined by the NOESY data. Consequently, **10** may be either of the two C-9′a epimers (9′a*S* or 9′a*R*). To differentiate between these two structural candidates, we performed quantum chemical ^13^C NMR chemical shift calculations to assign the correct configuration at C-9′a. Comparison of the experimental and calculated ^13^C NMR data revealed that 9′a*S*-epimer (Figure 8, *R*^2^ = 0.9989, Table S3) exhibited markedly better agreement with the experimental values than 9′a*R*-epimer. Notably, for the 9′a*R*-epimer (Table S4), the calculated chemical shifts for C-3′ (131.4) and C-4′ (75.0) displayed significant deviations from the experimental data (119.8 and 67.7, respectively), with discrepancies of approximately 10 ppm. In addition, the significant upfield-shift in H-3, compared with those of **11**, **13** and **14** (δ_H_ 2.82 vs. 3.75, 3.64 and 3.97), is likely attributed to the shielding effect exerted by the aromatic ring of the xanthone moiety. This spatial proximity, which can only be achieved in the 9′a*S*-epimer, provided further indirect evidence supporting the assignment of 9′a*S*-configuration. Thus, the structure of **10** was established as depicted. The absolute configuration of **10** was confirmed by ECD calculation (Figure 9A).

**Figure 10:**
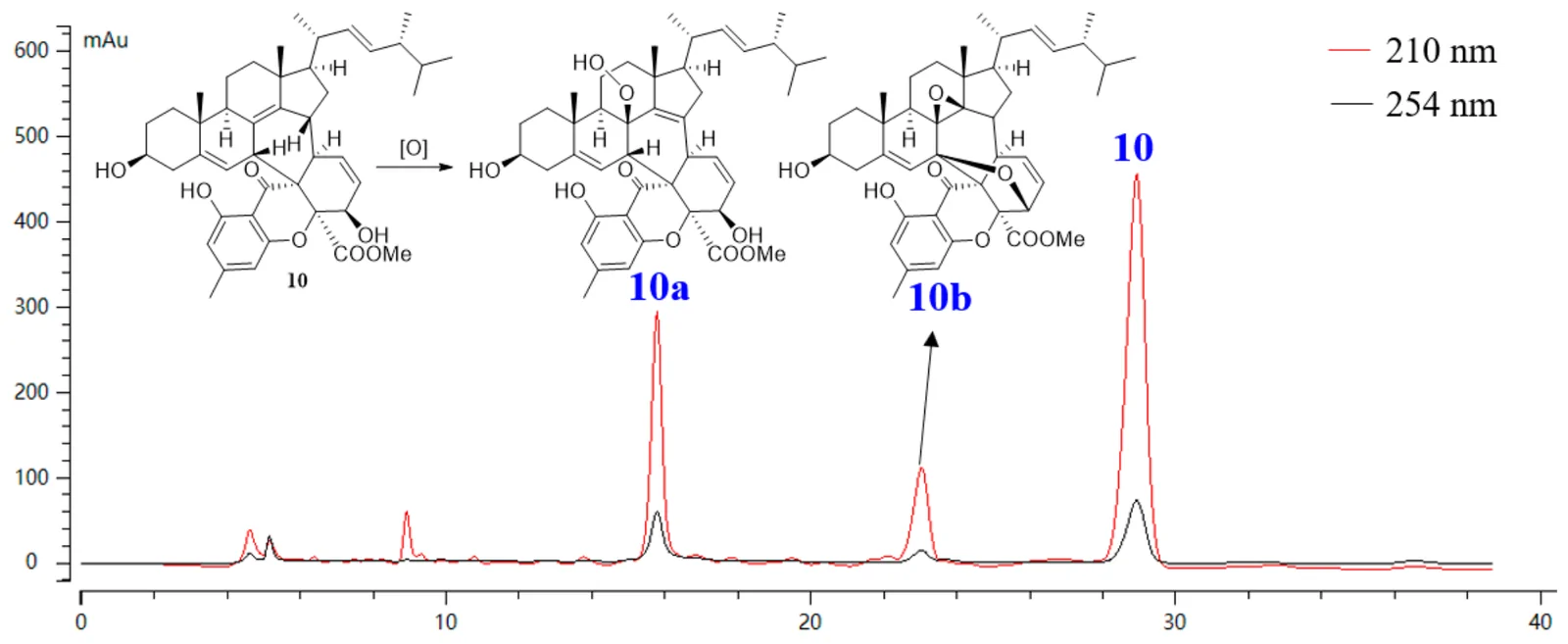
HPLC profiles of compound **10** and its two oxidative storage artifacts (**10a** and **10b**) detected after 48 months of preservation as a neat solid at 4 °C.

In addition, two minor related analogues were isolated, but their high-quality NMR data were unavailable owing to the limited sample amounts. It is worth noting that TLC and HPLC analyses of compound **10**, which was kept at 4 °C over a period of four years (Figure 10), showed the presence of two significant impurities. These two impurities were further isolated, and their ^1^H NMR spectra proved to be identical to those of the two minor analogues mentioned above.

Compound **10a** was assigned the molecular formula C_44_H_56_O_9_. Comparison of its NMR data with those of **10** indicated that **10a** contains one additional oxygenated quaternary carbon and one fewer methine carbon. HMBC correlations from H-6, H-7, H-9, and H_2_-11 located this oxygenated carbon at C-8. The double bond originally between C-8 and C-14 was found to have migrated to C-14 and C-15, as established by the HMBC correlations from H-9, H_2_-16, H-17, and H_3_-18 to C-14, and from H_2_-16, H-1′, and H-2′ to C-15. Consequently, the remaining HO_2_ unit required by the molecular formula was assigned to a hydroperoxy group at C-8, which was further corroborated by the characteristic hydroperoxy proton signal at δ_H_ 7.19 (br s) and the carbon resonance at δ_C_ 84.4. The β-orientation of the hydroperoxy group at C-8 was assigned based on a combination of NMR spectroscopic evidence and molecular modeling. Compared with compound **10**, the upfield shifts in H-1α (0.50 vs. 0.72) and H-3 (2.41 vs. 2.82 ppm) in **10a** suggested that the benzene ring of the xanthone moiety is in closer proximity to the leftmost ring of the steroid moiety. This spatial compression effect is fully consistent with the β-configuration of the hydroperoxy group, as revealed by Chem3D energy-minimized models, which showed a significantly shorter interring distance (cal. 3.0 in **10a** vs. 4.2 Å in **10**) when the hydroperoxy group adopted the β-orientation (Figure 9B).

Compound **10b** was assigned the molecular formula C_44_H_54_O_8_, indicating that **10b** possessed one more degree of unsaturation than **10**. The NMR data revealed that **10b** lacked the Δ^8(14)^ present in **10**, and instead contained three additional oxygenated non-protonated carbons (δ_C_ 84.2, 61.7, and 72.0), which were assigned to C-7, C-8, and C-14, respectively, based on HMBC correlations (Figure 7). The remaining two degrees of unsaturation were accounted for by two additional rings. The C-4′-O-C-7 ether bridge was established by the strong HMBC correlation from H-4′ to C-7 and the downfield shift in C-4′ (δ_C_ 67.7 vs. 76.0). In addition, the chemical shifts in C-8 (δ_C_ 61.7) and C-14 (δ_C_ 72.0), together with the remaining degree of unsaturation, supported the presence of an epoxy ring between C-8 and C-14. In precursor **10**, the 4′-hydroxy group was spatially close to C-7, which was activated by the adjacent two double bonds. This favorable arrangement facilitated an intramolecular nucleophilic attack of the 4′-hydroxy oxygen on C-7 upon oxidative activation, leading to the formation of the β-oriented ether bridge in **10b**. The β-orientation of the epoxy moiety was proposed to be governed by the steric hindrance on the α-face of the C-8 and C-14, which was provided by H-9, H-12α, H-17, H-1′, and the carbonyl group of the xanthone moiety. The relative configuration of **10b** was confirmed by the good correlation between the experimental and calculated ^13^C NMR chemical shifts (Figure 8).

### 2.2. Biological Activity Assessment

The isolated compounds were evaluated for their anti-ferroptotic and cytotoxic activities.

#### 2.2.1. Compound **15** as a Ferroptosis Inhibitor

**Figure 11:**
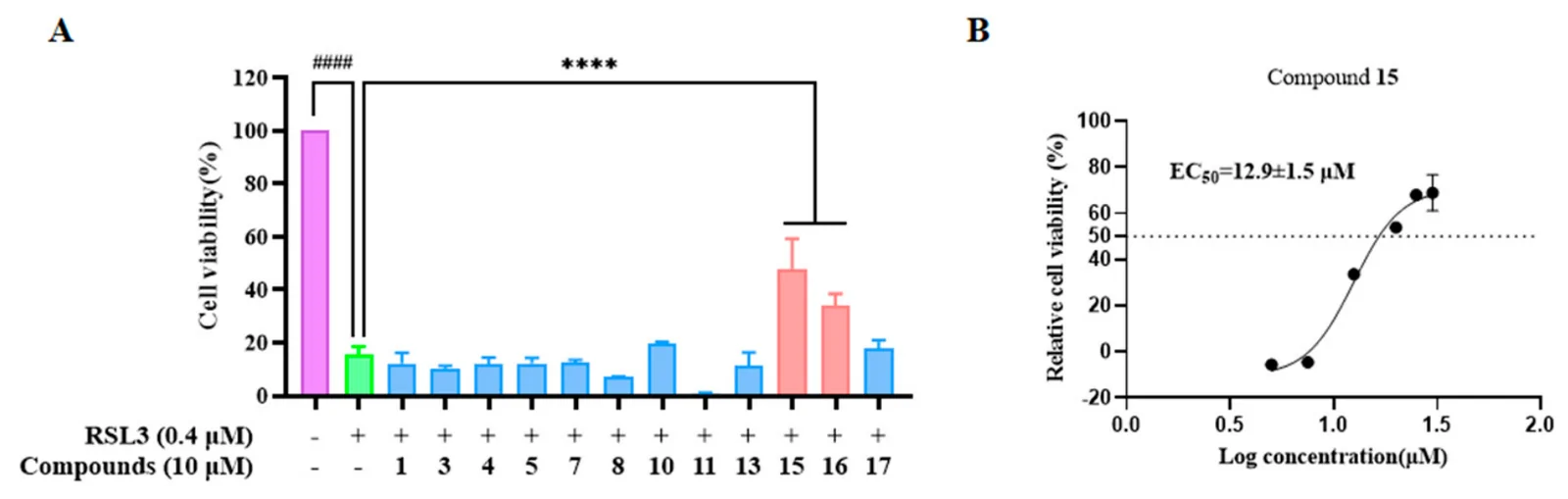
Compound **15** inhibited ferroptosis in HK-2 cells. (**A**) Viability of HK-2 cells treated with tested compounds (10 μM) and RSL3 (0.4 μM). (**B**) Dose-dependent protective effect of **15** (5-30 μM) in RSL3-induced HK-2 cells. Data are presented as mean ± SD (*n* = 3). ^####^ *p* < 0.0001 vs. Control; **** *p* < 0.0001 vs. RSL3-treated groups. DMSO: vehicle control; Fer-1 (1 μM): positive control.

The anti-ferroptotic activity of these compounds was evaluated in RSL3-induced HK-2 cells, with the exception of compounds **2**, **6**, **9**, **12**, and **14** due to insufficient amounts. As shown in Figure 11A, treatment with 0.4 μM RSL3 markedly reduced cell viability to 15% of untreated controls. Notably, at 10 μM, compounds **15** and **16** exhibited cytoprotective effects, restoring viability to 47.8% and 34%, whereas other compounds showed negligible protective effects (<30% viability). To further characterize the potency of **15**, concentration-response experiments (5–30 μM) were conducted. Ferrostatin-1 (Fer-1), a canonical ferroptosis inhibitor, was used as the reference. As depicted in Figure 11B, **15** exerted a dose-dependent protective effect, with cell viability increasing from 24% (5 μM) to 78% (30 μM) (EC_50_ = 12.8 ± 1.6 μM). Notably, its maximal efficacy at 30 μM restored viability to 78%, positioning **15** as a promising ferroptosis inhibitor. Unfortunately, the limited quantity of purified **16** prevented us from conducting further biological testing.

**Figure 12:**
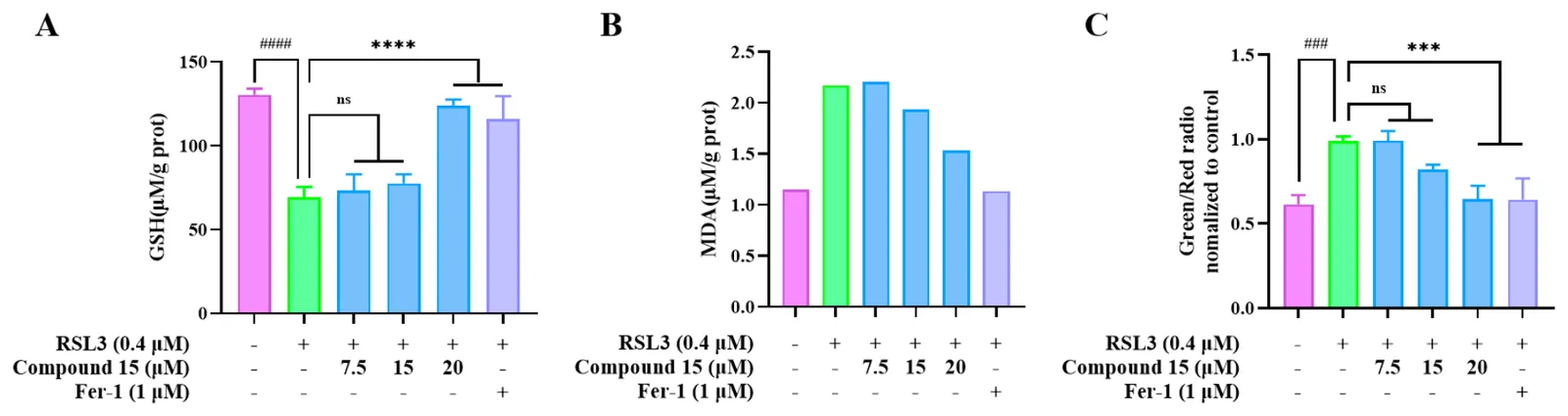

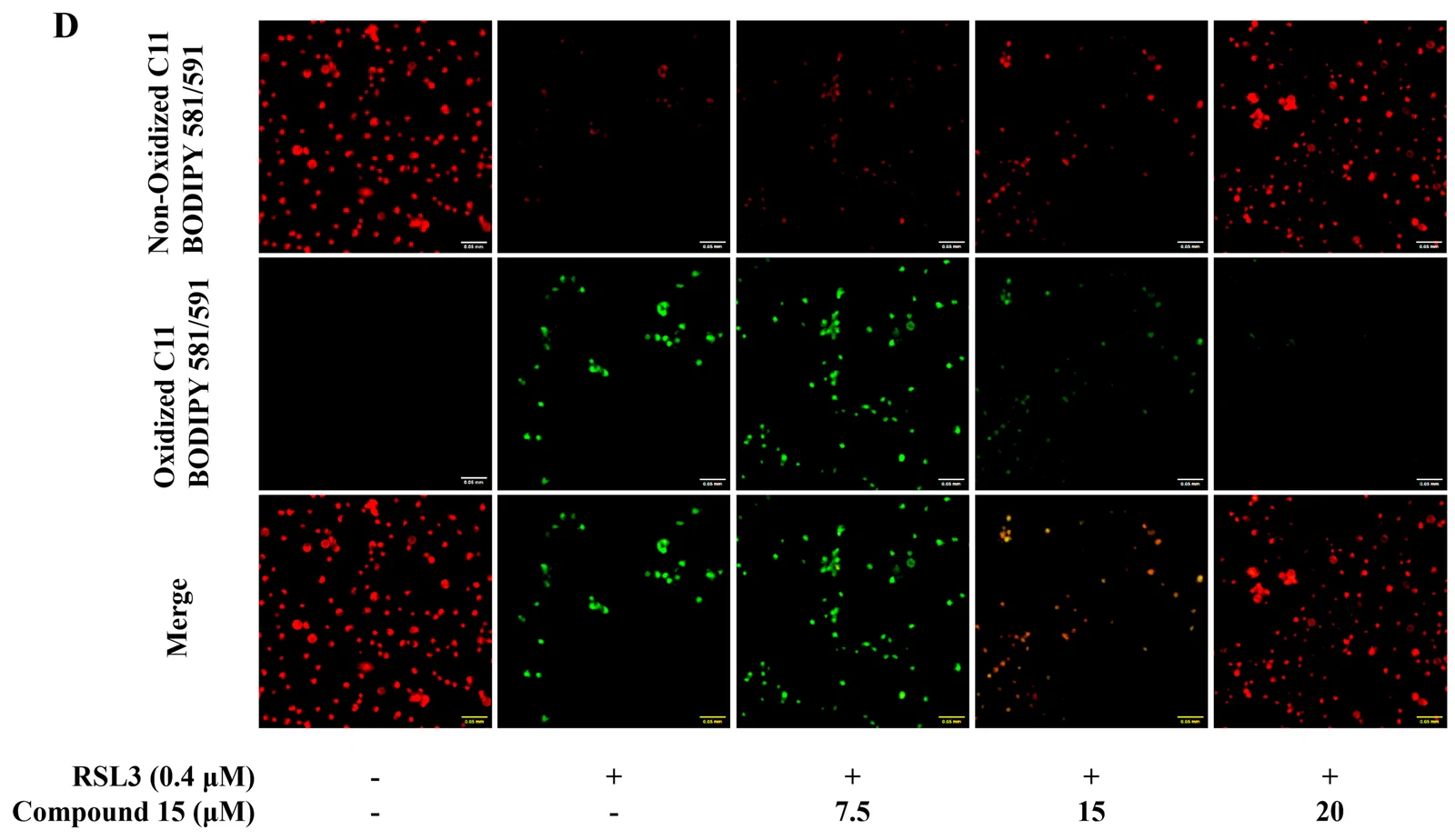
Compound **15** attenuated ferroptosis by inhibiting lipid peroxidation. (**A**) GSH content; (**B**) MDA levels; (**C**,**D**) C11 BODIPY fluorescence ratio (green/red) reflecting lipid peroxidation. Data are presented as mean ± SD (*n* = 3–6); ^###^ *p* < 0.001, ^####^ *p* < 0.0001 vs. control; *** *p* < 0.001, **** *p* < 0.0001 vs. RSL3; ns, not significant.

Given the ferroptosis-inhibitory activity of compound **15** (Figure 11), we further explored the underlying mechanism through comprehensive analysis of oxidative stress markers and lipid peroxidation dynamics. As shown in Figure 12A,B, treatment with 0.4 μM RSL3 for 24 h induced pronounced lipid peroxidation in HK-2 cells, and intracellular glutathione (GSH) levels decreased to 53.1% of control levels, concurrently with a 1.9-fold increase in malondialdehyde (MDA) levels compared to untreated controls. Notably, cotreatment with 20 μM **15** significantly reversed these effects, normalizing MDA levels to 1.33-fold that of the control and restoring GSH to 94.8% of control levels. These data indicate that **15** attenuates RSL3-induced oxidative stress. Further analysis using fluorescence imaging with the oxidation-sensitive fluorescent probe C11-BODIPY^581/591^ revealed that RSL3 markedly intensified lipid peroxidation, increasing the green/red fluorescence ratio to 1.61-fold of the control (Figure 12C,D). However, cotreatment with compound **15** dose-dependently attenuated this ratio to 1.3-fold at 15 μM and to the control level (1.0-fold) at 20 μM, clearly indicating suppression of lipid peroxidation.

#### 2.2.2. Compounds **11** and **16** Exhibited Cytotoxic Activity Against MCF-7 Cells

**Figure 13:**
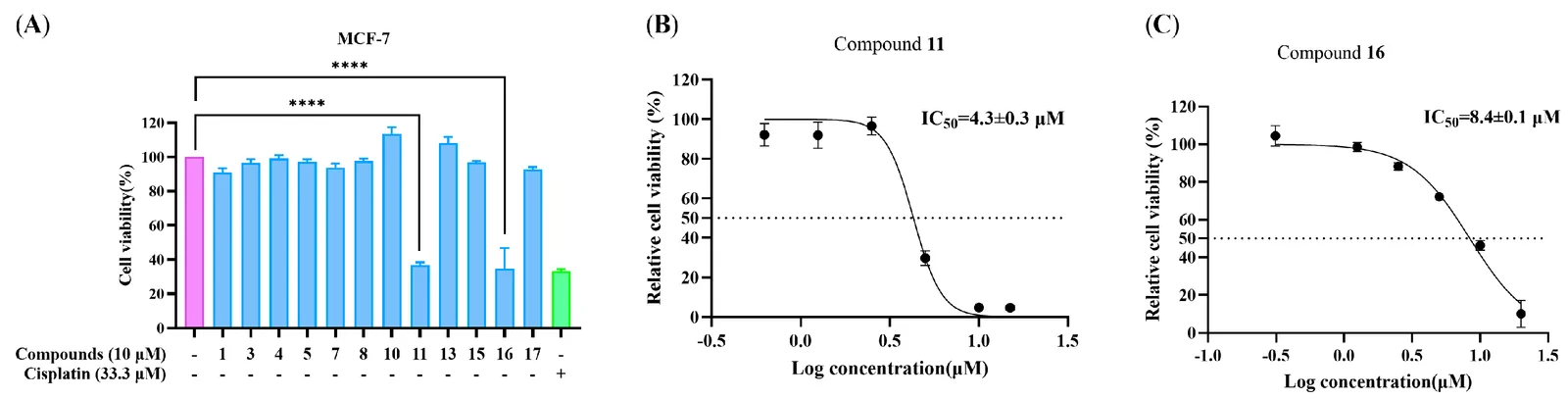
Cytotoxic evaluation of selected compounds in MCF-7 human breast cancer cells. (**A**) Cell viability of MCF-7 cells treated with the tested compounds at 10 μM. Cisplatin (33.3 μM) served as positive control. (**B**,**C**) IC_50_ curves of compound **11** and **16**. Data are presented as mean ± SD (*n* = 3). **** *p* < 0.0001 vs. vehicle control.

The same set of compounds was tested for cytotoxicity against MCF-7 cells (Figure 13), with compounds **2**, **6**, **9**, **12**, and **14** excluded for the same reason. Single-concentration cytotoxic screening revealed that compounds **11** and **16** exhibited antiproliferative effects against MCF-7 cells at 10 μM. After treatment with compound **11**, the cell viability decreased to approximately 36%, while compound **16** reduced cell viability to around 34%. Their inhibitory effects were comparable to that of cisplatin (33.3 μM), the positive control. Subsequent dose–response assays further confirmed the cytotoxic activity, and the IC_50_ values of compounds **11** and **16** were calculated to be 4.3 ± 0.3 μM and 8.4 ± 0.1 μM, respectively. Compound **11** displayed stronger cytotoxic activity than compound **16** against MCF-7 human breast cancer cells. Cytotoxicity against non-cancerous cell lines was not assessed in the current study.

## 3. Materials and Methods

### 3.1. General Experimental Procedure

Specific rotations were determined on a Rudolph Autopol VI polarimeter (Rudolph Research Analytical, Hackettstown, NJ, USA) at the sodium D-line (589 nm) and 25 °C. UV spectra were recorded on a UV-2600 spectrometer (Shimadzu Co., Kyoto, Japan) at room temperature over 190–400 nm. ECD spectra were recorded on a Chirascan V100 spectrometer (Applied Photophysics, Surrey, UK) at room temperature over 200–400 nm with a bandwidth of 1.0 nm, a scan rate of 100 nm/min, and a response time of 1 s. The NMR spectra were acquired on Bruker AVANCE III HD 400 MHz and 600 MHz spectrometers (Bruker, Fällanden, Switzerland) using solvent signals (Methanol-*d*_4_: δ_H_ 3.31/δ_C_ 49.0; CDCl_3_: δ_H_ 7.26/δ_C_ 77.0) as references. All spectra were recorded at 298 K. The relaxation delay (*d*_1_) was 1.5 s for ^1^H and 2.0 s for ^13^C. The number of scans (NS) was 16 for ^1^H, 1024 for ^13^C, and 8–64 for 2D experiments. HRESIMS spectra were acquired on an Agilent 6546A Q-TOF mass spectrometer (Agilent Technologies, Singapore) and a Bruker FTMS spectrometer (Bruker Daltonics, Bremen, Germany), both equipped with ESI sources and operated in both positive and negative ion modes over *m*/*z* 100–1000. Mass resolution was 30,000 (FWHM at *m*/*z* 118) with mass accuracy < 0.8 ppm. Samples were separated on a C18 column (2.1 × 50 mm, 1.8 μm) eluted with 80% aq. acetonitrile isocratically at 0.3 mL/min over 5 min, with 5 μL injection. Semi-preparative high-performance liquid chromatography (HPLC) was undertaken on a Shimadzu LC-6AD pump (Shimadzu Co., Kyoto, Japan) equipped with a UV detector, employing a YMC-Pack ODS-A HPLC (YMC Co., Ltd., Kyoto, Japan) column (250 mm × 10 mm, *S*-5 μm, 12 nm). Single-crystal X-ray diffraction data were collected on two diffractometers: Cu K*α* data were acquired on a Rigaku XtaLAB Synergy-R diffractometer (Rigaku Corporation, Tokyo, Japan; Cu K*α*, *λ* = 1.54184 Å, 1.2 kW) and reduced using CrysAlisPro (version 1.171.44.91a); Mo Kα data were collected on a Bruker D8 VENTURE diffractometer (Bruker AXS Inc., Madison, WI, USA; Mo K*α*, *λ* = 0.71073 Å) and reduced using SAINT (version 8.40B). All structures were solved using SHELXT (version 2014/5) and refined using SHELXL (version 2019/3) within the Olex2 (version 1.5) interface.

### 3.2. Fungal Strain and Identification

The strain MABC05-5-M4 was isolated from the deep-sea water sample (−5644 m) collected from the Western Pacific Ocean (DY27I-MABC05). The strain was identified as *Penicillium rubens* by Changxu Huang (from Prof. Zhuhua Luo’s group) based on microscopic examination and internal transcribed spacer (ITS) sequencing. Its ITS sequence was deposited in GenBank under accession number KP200040, and the strain was preserved at the Key Laboratory of Marine Biogenetic Resources, Third Institute of Oceanography, Ministry of Natural Resources (MCCC 3A00591).

### 3.3. Fermentation, Extraction, and Isolation

The fermentation was carried out in 50 Fernbach flasks (500 mL), each containing 80 g of rice. Artificial seawater (100 mL) was added to each flask, and the contents were soaked for three hours before autoclaving at 15 psi for 30 min. After cooling to room temperature (r.t.), each flask was inoculated with 3.0 mL of the spore inoculum and incubated at r.t. for 30 days. The fermented material from all flasks was combined, soaked in EtOAc (5 L), and ultrasonically extracted for 1 h. The EtOAc solvent was concentrated to give 7.21 g of crude extract.

The extract was fractionated by MCI gel column chromatography (CC) with MeOH/H_2_O (20:80 → 100:0) as eluent to yield 9 fractions (Fr.1–Fr.9). Fraction 5 (2.7 g) was separated by an ODS silica gel CC (55 g) using a gradient elution of MeOH/H_2_O (10:90 → 60:40, 500 mL per gradient) to yield **6** subfractions (Fr.5.1–Fr.5.6). Fr.5.3 (0.15 g) was purified by HPLC (YMC-Pack ODS-A column, 250 × 10 mm, S-5 μm, 12 nm) using MeCN/H_2_O (52:48, 2 mL/min) as the mobile phase to afford compound **1** (20.0 mg, t*_R_* 29.0 min, yield 0.28%). Compounds **2** (3.1 mg, t*_R_* 31.6 min, yield 0.04%) and **6** (1.3 mg, t*_R_* 39.7 min, yield 0.02%) were obtained from fractions Fr.5.4 (0.38 g) and Fr.5.5 (1.37 g) by HPLC using MeCN/H_2_O (65:35, 2 mL/min). The fraction Fr.5.6 (0.97 g) was further separated on an ODS silica gel CC using MeOH/H_2_O (40:60 → 90:10) as eluent to yield 5 subfractions (Fr.5.6.1–Fr.5.6.5). Fr.5.6.2 (30.0 mg) was purified by HPLC using MeCN/H_2_O (56:44, 2 mL/min) as the mobile phase to afford compound **8** (3.7 mg, t*_R_* 37.9 min, yield 0.05%). Fr.5.6.4 (78.2 mg) was purified by HPLC using MeCN/H_2_O (47:53, 3 mL/min) as the mobile phase to afford compound **5** (3.5 mg, t*_R_* 32.8 min, yield 0.05%) and compound **11** (6.2 mg, t*_R_* 47.2 min, yield 0.09%). Fr.5.6.5 (87.6 mg) was purified by HPLC using MeCN/H_2_O (42:58, 2.5 mL/min) as the mobile phase to afford compound **13** (5.5 mg, t*_R_* 20.3 min, yield 0.08%) and compound **12** (2.6 mg, t*_R_* 36.5 min, yield 0.04%). Fr.6 (2.3 g) was separated on an ODS silica gel CC using a gradient elution of MeOH/H_2_O (40:60 → 90:10) to yield 12 subfractions (Fr.6.1–Fr.6.12). Fr.6.3 (91.8 mg) was purified by HPLC using MeOH/H_2_O (57:43, 3 mL/min) to afford compounds **3** (4.3 mg, t*_R_* 23.1 min, yield 0.06%) and **7** (3.6 mg, t*_R_* 34.1 min, yield 0.05%). Fr.6.4 (81 mg) was purified by HPLC using MeOH/H_2_O (60:40, 2 mL/min) to afford compounds **4** (5.3 mg, t*_R_* 23.6 min, yield 0.07%) and **9** (1.2 mg, t*_R_* 40.9 min, yield 0.02%). Fr.7 (1.1 g) was purified by ODS silica gel CC using a gradient elution of MeOH/H_2_O to yield 7 subfractions (Fr.7.1–Fr.7.7). Fr.7.4 (0.61 g) was purified by HPLC using MeOH/H_2_O (47:53, 3 mL/min) to afford compound **16** (15.6 mg, t*_R_* 16.9 min, yield 0.22%) and compound **17** (6.3 mg, t*_R_* 25.1 min, yield 0.09%). Fraction F.8 (1.7 g) was purified on an ODS silica gel CC eluting with MeOH/H_2_O (30:70 → 100:0) to yield 9 subfractions (Fr.8.1–Fr.8.9). Fr.8.7 (109.3 mg) was purified by HPLC using MeCN/H_2_O (47:53, 3 mL/min) to afford compounds **14** (1.3 mg, t*_R_* 23.1 min, yield 0.02%) and **15** (3.6 mg, t*_R_* 34.1 min, yield 0.05%). Fr.8.8 (62.6 mg) was purified by HPLC using 100% MeCN (3 mL/min) as the mobile phase to afford compound **10** (13.8 mg, t*_R_* 29 min, yield 0.19%). After storage for 48 months, compound **10** was repurified by HPLC using 100% MeCN (3 mL/min) to afford compounds **10a** (1.6 mg, t*_R_* 14.6 min), **10b** (0.9 mg, t*_R_* 22.3 min), and **10** (4.1 mg, t*_R_* 28.4 min). All yields were calculated based on the initial crude extract (7.21 g).

Penirubenoid A (**1**): Colorless crystals (MeOH); [α]^25^_D_ +476.5 (*c* 0.1, MeOH); UV (MeOH) *λ*_max_ 219, 281 nm; ECD (*c* 2.9 × 10^−4^ M, MeOH) *λ*_max_ (Δ*ε*) 290 (+10.20), 266 (−0.43), 233 (+4.36) nm; ^1^H and ^13^C NMR data, see Table 1; ESIMS *m*/*z* 345.2054 [M + H]^+^ (calcd for C_21_H_29_O_4_^+^, 345.2066).

Penirubenoid B (**2**)*:* Colorless oil; [α]^25^_D_ +45 (*c* 0.05, MeOH); ECD (*c* 5.3 × 10^−4^ M, MeOH) *λ*_max_ (Δ*ε*) 333 (−0.93), 267 (+2.71), 234 (+2.28) nm; ^1^H and ^13^C NMR data, see Table 1; ESIMS *m*/*z* 361.2126 [M + H]^+^ (calcd for C_21_H_29_O_5_^+^, 361.2015).

Penirubenoid C (**3**)*:* Colorless crystals (MeOH); [α]^25^_D_ +240.0 (*c* 0.1, MeOH); UV (MeOH) *λ*_max_ 258 nm; ECD (*c* 2.8 × 10^−4^ M, MeOH) *λ*_max_ (Δε) 325 (−1.91), 235 (+14.52) nm; ^1^H and ^13^C NMR data, see Table 1; ESIMS *m*/*z* 359.1845 [M + H]^+^ (calcd for C_21_H_27_O_5_^+^, 359.1853).

Penirubenoid D (**4**)*:* Colorless oil; [α]^25^_D_ +193.2 (*c* 0.1, MeOH); UV (MeOH) λ_max_ 260 nm; ECD (*c* 2.9 × 10^−4^ M, MeOH) *λ*_max_ (Δ*ε*) 240 (+4.04) nm; ^1^H and ^13^C NMR data, see Table 1; ESIMS *m*/*z* 363.2158 [M + H]^+^ (calcd for C_21_H_31_O_5_^+^, 363.2166).

Penirubenoid E (**5**)*:* Colorless oil; [α]^25^_D_ +206.5 (*c* 0.1, MeOH); UV (MeOH) λ_max_ 271 nm; ECD (*c* 2.8 × 10^−4^ M, MeOH) *λ*_max_ (Δ*ε*) 290 (+11.93), 266 (−0.98), 240 (+4.69) nm; ^1^H and ^13^C NMR data, see Table 1; ESIMS *m*/*z* 421.2218 [M + H]^+^ (calcd for C_23_H_33_O_7_^+^, 421.2221).

Penirubenoid F (**6**)*:* Colorless oil; [α]^25^_D_ +42 (*c* 0.1, MeOH); ECD (*c* 5.6 × 10^−4^ M, MeOH) *λ*_max_ (Δ*ε*) 335 (−0.55), 240 (+2.41) nm; ^1^H and ^13^C NMR data, see Table 1; ESIMS *m*/*z* 361.2013 [M + H]^+^ (calcd for C_21_H_29_O_5_^+^, 361.2010).

Peniruxanthosterol A (**10**)*:* Colorless oil; [α]^25^_D_ +206.5 (*c* 0.1, MeOH); UV (MeOH) λ_max_ 338, 282 nm; ECD (*c* 2.9 × 10^−4^ M, MeOH) *λ*_max_ (Δ*ε*) 354 (+3.81), 323 (−8.70), 259 (−14.54), 226 (−37.84) nm; ^1^H and ^13^C NMR data, see Table 2; ESIMS *m*/*z* 695.3953 [M − H]^−^ (calcd for C_44_H_55_O_7_^−^, 695.3953).

Peniruxanthosterol A1 (**10a**)*:* Colorless oil; [α]^25^_D_ −93.0 (*c* 0.1, MeOH); ECD (*c* 2.8 × 10^−4^ M, MeOH) *λ* _max_ (Δε) 352 (+2.78), 299 (+2.13), 269 (−12.81), 236 (−7.08), 206 (+31.08) nm; ^1^H and ^13^C NMR data, see Table 2; ESIMS *m*/*z* 727.3850 [M − H]^−^ (calcd for C_44_H_55_O_9_^−^, 727.3852).

Peniruxanthosterol A2 (**10b**)*:* Colorless oil; [α]^25^_D_ −154.0 (*c* 0.05, MeOH); ECD (*c* 2.8 × 10^−4^ M, MeOH) *λ*_max_ (Δ*ε*) 347 (+4.27), 282 (−21.73), 228 (−26.68), 207 (+65.61) nm; ^1^H and ^13^C NMR data, see Table 2; ESIMS *m*/*z* 709.3739 [M − H]^−^ (calcd for C_44_H_53_O_8_^−^, 709.3747).

The known compounds were identified as talarolutin D (**7**) [1], talarolutin B (**8**) [1], talarolutin A (**9**) [1], 3*β*,15*β*-dihydroxyl-(22*E*,24*R*)-ergosta-5,8(14),22-trien-7-one (**11**) [16], 15*β*-hydroxyl-(22*E*,24*R*)-ergosta-3,5,8,22-tetraen-one (**12**) [17] ergosterol (**13**) [18], ergosterol peroxide (**14**) [18], (4*R*,4a*S*,9a*R*)-1,9a-dihydronidulalin A (**15**) [19], 1-hydroxy-3-hydroxymethyl-9H-xanthen-9-one (**16**) [20], nidulalin B (**17**) [21].

### 3.4. X-Ray Crystallographic Analysis

Colorless crystals of penirubenoid A (**1**) and C (**3**) were obtained by slow evaporation from MeOH/H_2_O (8:1 and 7:1, *v*/*v*), respectively, while colorless crystals of penirubenoid F(**6**) were obtained by slow evaporation from methanol. Suitable crystals were selected and mounted on loops for diffraction experiments. Data were collected at 150.15 K for **1** and **3**, and at 100.00(10) K for **6**. All structures were solved using intrinsic phasing with the SHELXT structure solution program and refined with the SHELXL refinement package (least-squares minimization) within the Olex2 interface.

Crystal Data for penirubenoid A (**1**). C_21_H_28_O_4_, *M*_r_ = 344.454, orthorhombic, space group *P*2_1_2_1_2_1_ (no. 19), *a* = 6.0607(3) Å, *b* = 14.2909(9) Å, *c* = 21.6010(12) Å, *α* = 90°, *β* = 90°, *γ* = 90°, *V* = 1870.92(18) Å^3^, *Z* = 4, *T* = 150.15 K, *μ*(Mo K*α*) = 0.083 mm^−1^, *D*_calc_ = 1.223 g/cm^3^, 17,570 reflections measured (5.7° ≤ 2*θ* ≤ 56.7°), 4626 unique (*R*_int_ = 0.0932, *R*_sigma_ = 0.0752), which were used in all calculations. The final *R*_1_ was 0.0699 (*I* > 2*σ*(*I*)) and w*R*_2_ was 0.1741 (all data). Crystallographic data have been deposited at the Cambridge Crystallographic Data Centre (CCDC 2581952).

Crystal Data for penirubenoid C (**3**). C_21_H_26_O_5_, *M*_r_ = 358.437, orthorhombic, space group *P*2_1_2_1_2_1_ (no. 19), *a* = 8.8159(6) Å, *b* = 11.6877(7) Å, *c* = 17.6814(14) Å, *α* = 90°, *β* = 90°, *γ* = 90°, *V* = 1821.8(2) Å^3^,*Z* = 4, *T* = 150.15 K, *μ*(Mo K*α*) = 0.092 mm^−1^, *D*_calc_ = 1.307 g/cm^3^, 21,798 reflections measured (4.18° ≤ 2*θ* ≤ 56.6°), 4531 unique (*R*_int_ = 0.0826, *R*_sigma_ = 0.0673), which were used in all calculations. The final *R*_1_ was 0.0639 (*I* > 2*σ*(*I*)) and w*R*_2_ was 0.1412 (all data). Crystallographic data have been deposited at the Cambridge Crystallographic Data Centre (CCDC 2581951).

Crystal Data for penirubenoid F (**6**). C_21_H_28_O_5_, *M*_r_ = 360.43, triclinic, space group *P*1 (no. 1), *a* = 5.9146(2) Å, *b* = 8.9798(4) Å, *c* = 9.1091(2) Å, *α* = 83.391(3)°, *β* = 72.379(3)°, *γ* = 78.477(3)°, *V* = 451.03(3) Å^3^, *Z* = 1, *T* = 100.00(10) K, *μ*(Cu K*α*) = 0.760 mm^−1^, *D*_calc_ = 1.327 g/cm^3^, 19,569 reflections measured (10.07° ≤ 2*θ* ≤ 148.884°), 3324 unique (*R*_int_ = 0.0470, *R*_sigma_ = 0.0273), which were used in all calculations. The final *R*_1_ was 0.0460 (*I* > 2*σ*(*I*)) and w*R*_2_ was 0.1190 (all data). Crystallographic data have been deposited at the Cambridge Crystallographic Data Centre (CCDC 2584211). Flack parameter = 0.1(3).

### 3.5. Biological Study

The ferroptosis-related assays (cell culture, cell viability, MDA and GSH levels, and C11-BODIPY^581/591^ staining) were performed as described previously [12]. Additionally, the cytotoxicity assay against MCF-7 cancer cell lines was evaluated following the reported method [22]. Detailed protocols are provided in the Supporting Information.

## Figures and Tables

**Table 1:** ^1^H and ^13^C NMR Data of **1**–**6** (*J* in Hz, δ in ppm).

No.	1 *^a^*		2 ^*b*^		3 *^c^*		4 ^*c*^		5 ^*c*^		6 ^*b*^	
	δ_H_	δ_C_	δ_H_	δ_C_	δ_H_	δ_C_	δ_H_	δ_C_	δ_H_	δ_C_	δ_H_	δ_C_
1		204.3, C		204.2, C		202.4, C	3.86, m	71.7, CH		210.3, C		206.3, C
2	5.70, d (10.2)	123.9, CH	5.75, d (10.1)	123.9, CH	5.70, d (10.2)	124.1, CH	1.99, m; 2.20, m	29.7, CH_2_	4.96, d (4.0)	68.8, CH	5.68, d (10.2)	124.5, CH
3	6.33, d (10.2)	154.7, CH	6.37, d (10.1)	154.8, CH	6.33, d (10.2)	150.5, CH	3.46, m	77.4, CH	5.31, d (4.0)	83.4, CH	6.27, d (10.2)	153.5, CH
4		36.2, C		36.3, C		41.8, C		38.0, C		37.6, C		43.2, C
5	1.66, m	50.8, CH	1.69, m	50.9, CH		78.2, C	1.87, m	42.2, CH	1.73, m	52.6, CH		79.2, C
6	β 1.49, m; α 1.79, m	19.8, CH_2_	α 1.83, m; β 1.51, m	19.8, CH_2_	β 1.85, m; α 1.79, m	25.0, CH_2_	β 1.43, m; 1.80, m	19.2, CH_2_	β 1.56, m; α 1.72, m	19.0, CH_2_	α 1.79, m; β 1.94, m	25.7, CH_2_
7	1.66, m; 2.07, m	38.9, CH_2_	2.12, dt (12.6, 6.2, 6.2); 1.69, m	38.9, CH_2_	2.27, m; 1.88, m	32.6, CH_2_	2.13, m; 1.77, m	39.9, CH_2_	1.70, m; 2.10, m	39.4, CH_2_	2.14, m; 1.78, m	34.0, CH_2_
8		83.1, C		84.0, C		83.0, C		85.4, C		82.7, C		81.6, C
9	1.90, dd (12.6, 4.2)	43.7, CH	1.90, dd (12.7, 4.3)	43.6, CH	2.70, dd (12.6, 4.5)	38.5, CH	2.24, dd (12.5, 4.1)	44.1, CH	2.15, dd (12.5, 4.4)	44.8, CH	2.56, dd (12.7, 4.3)	39.8, CH
10		47.3, C		47.4, C		53.5, C		41.4, C		50.6, C		54.6, C
11	β 2.00, dd (15.3, 12.6) α 3.23, dd (15.3, 4.2)	17.6, CH_2_	α 3.38, dd (15.4, 4.3); β 2.03, dd (15.4, 12.7)	17.4, CH_2_	α 3.36, dd (16.3, 4.5) β 2.17, dd (16.3, 12.6)	17.5, CH_2_	β 2.08, dd (15.6, 12.5) α 2.56, dd (15.6, 4.1)	14.9, CH_2_	β 1.97, dd (15.5,12.5); α 3.03 (15.5, 4.4)	16.8, CH_2_	β 1.96, m α 3.15, ddd (16.2, 4.2, 1.6)	20.0, CH_2_
12	1.28, s	20.3, CH_3_	1.33, s	20.3, CH_3_	1.33, s	18.5, CH_3_	1.30, s	20.7, CH_3_	1.298, s	20.9, CH_3_	1.33, s	21.2, CH_3_
13	β 1.079, s	21.7, CH_3_	β 1.11, s	21.8, CH_3_	β 1.26, s	23.8, CH_3_	β 0.84, s	28.5, CH_3_	β 1.24, s	21.5, CH_3_	β 1.26, s	26.0, CH_3_
14	α 1.082, s	31.0, CH_3_	α 1.12, s	31.1, CH_3_	α 1.15, s	25.4, CH_3_	α 1.05, s	21.7, CH_3_	α 0.94, s	27.5, CH_3_	α 1.12, s	24.1, CH_3_
15	1.18, s	14.9, CH_3_	1.21, s	15.0, CH_3_	1.35, s	20.6, CH_3_	0.86, s	15.3, CH_3_	1.305, s	14.6, CH_3_	1.33, s	19.2, CH_3_
1′		168.1, C		168.3, C		162.2, C		163.0, C		168.2, C		165.7, C
2′		91.1, C		87.9, C		99.5, C		98.6, C		90.5, C		100.4, C
3′		191.0, C		191.2, C		180.4, C		180.7, C		191.0, C		170.3, C
4′	2.35, dd (16.9,3.2); 2.48, dd (16.9,13.9)	42.6, CH_2_	3.86, d (12.2)	71.6, CH	6.0, s	112.0, CH	5.94, s	111.4, CH	2.39, dd (16.9,13.9); 2.52, dd (16.9, 3.2)	42.6, CH_2_	2.36, ddd (16.9, 4.2, 1.3); 2.49, dddd (16.9, 10.4, 2.0, 2.0)	34.8, CH_2_
5′	4.57, ddq (13.9, 6.3, 3.2)	75.5, CH	4.23, dq (12.2, 6.2)	79.6, CH		160.4, C		160.7, C	4.59, ddq (13.9, 6.2, 3.2)	75.8, CH	4.55, ddq (10.4, 6.3, 4.2)	73.5, CH
6′	1.43, d (6.3)	20.4, CH_3_	1.57, d (6.2)	17.9, CH_3_	2.20, s	19.2, CH_3_	2.21, s	19.1, CH_3_	1.46, d (6.2)	20.5, CH_3_	1.41, d (6.3)	20.8, CH_3_
									2.02, s	20.7, 170.0		

*^a^* ^1^H NMR at 500 MHz, ^13^C NMR at 75.5 MHz. *^b^* ^1^H NMR at 600 MHz, ^13^C NMR at 151 MHz. *^c^* ^1^H NMR at 500 MHz, ^13^C NMR at 126 MHz.

**Table 2:** The ^1^H (600 MHz) and ^13^C NMR data (151 MHz) of **10**, **10a**, **10b**, **11**, and **14** in CDCl_3_.

No.	10		10a		10b		11	14
	δ_H_	δ_C_	δ_H_	δ_C_	δ_H_	δ_C_		
1	1.60, m; 0.72, m	36.2	1.63, m; 0.50, m	38.1	1.03, m; 1.70, m	36.3	35.1	
2	1.67, m; 1.27, m	31.8	1.31, m; 1.58, m	31.0	1.80, m; 1.41, m	31.4	31.0	
3	2.82, m	71.5	2.41, m	72.1	3.37, m	70.4	69.9	
4	2.05, m	42.5	2.14, t; 1.91, m	42.9	2.17, m	42.6	41.6	
5		143.5		141.6		149.8	169.4	
6	5.86, d (3.1)	121.8	5.80, d (4.3)	119.8	5.95, s	125.1	127.2	
7	3.34, d (3.1)	41.4	4.26, dd (4.3, 2.8)	37.2		84.2	191.1	
8		124.9		84.4		61.7	127.9	
9	1.19, m	46.8	1.13, dd (12.5, 3.0)	48.6	1.43, m	51.8	46.8	
10		35.5		35.0		35.4	39.7	
11	1.50, m	19.6	1.91, m; 1.31, m	20.0	1.57, m; 1.27, m	16.6	19.2	
12	1.93, m; 1.54, m	37.7	2.01, m; 1.46, m	43.2	1.65, m; 1.39, m	39.5	35.2	
13		42.6		47.1		41.3	45.6	
14		149.4		138.0		72.0	170.0	
15	2.10, m	41.5		139.3	2.46, dd (12.6, 5.7)	38.0	69.9	
16	1.85, m; 1.73, m	37.2	2.36, dd (15.3, 10.9); 2.20, dd (15.3, 8.2)	39.7	2.18, m; 1.75, m	35.0	36.7	
17	1.92, m	54.1	2.00, m	58.5	1.96, m	53.8	53.3	
18	0.92, s	19.6	1.24, s	17.2	1.07, s	18.3	19.5	
19	0.71, s	18.9	0.93, s	19.6	1.08, s	19.9	18.9	
20	2.12, m	39.6	2.26, m	38.9	2.13, m	40.0	38.5	
21	1.08, d (6.7)	21.1	1.05, d (6.4)	21.2	1.03, d (6.3)	20.9	21.7	
22	5.30, dd (15.2, 7.7)	135.8	5.23, dd (15.2, 8.5)	135.1	5.26, dd (15.2, 7.2)	134.7	134.9	
23	5.24, dd (15.2, 7.0)	132.0	5.30, dd (15.2, 7.4)	132.4	5.30, dd (15.2, 8.2)	132.9	132.8	
24	1.89, m	42.8	1.89, m	42.7	1.90, m	43.1	42.9	
25	1.49, m	33.1	1.50, m	33.1	1.49, m	33.2	33.1	
26	0.86, d (6.7)	19.9	0.86, d (6.6)	19.9	0.87, d (6.9)	20.1	19.7	
27	0.84, d (6.7)	19.6	0.84, d (6.6)	19.6	0.86, d (6.9)	19.7	20.0	
28	0.94, d (6.9)	17.6	0.94, d (6.4)	17.6	0.96, d (6.8)	18.1	17.5	
1′	2.90, ddd (7.4, 3.3, 1.4)	46.1	3.57, br s	40.4	3.24, s	43.1		25.6
2′	5.95, d (10.0, 3.3)	138.1	5.71, d (10.1)	131.0	5.55, dd (8.6, 3.5)	137.9		127.4
3′	5.71, ddd (10.0, 6.2, 1.4)	119.8	5.86, ddd (10.1, 4.9, 2.9)	121.9	5.91, dd (8.6, 5.6)	126.8		127.3
4′	4.77, d (6.2)	67.7	4.97, d (4.9)	67.5	4.70, d (5.6)	76.0		69.3
4′a		88.4		87.4		96.2		108.4
5′	6.24, d (1.2)	106.9	6.27, s	107.2	6.30, s	108.5		151.1
5′a		157.8		157.3		157.2		111.1
6′		148.7		149.4		150.6		162.5
7′	6.28, d (1.2)	110.2	6.33, s	110.8	6.42, s	112.1		197.1
8′		160.4		160.7		162.0		82.7
8′a		107.0		107.8		107.6		159.0
9′		198.1		199.2		195.7		103.6
9′a		54.9		53.2		56.5		45.2
10′	2.26, s	22.3	2.22, s	22.3	2.32, s	22.6		22.6
11′		170.2		168.7		167.0		170.7
12′	3.57, s	53.1	3.61, s	53.2	3.60, s	52.4		53.4

## Data Availability

Data is contained within the article and Supplementary Materials.

## Supplementary Materials

The following supporting information can be downloaded at https://www.mdpi.com/article/10.3390/md24090326/s1: Figure S1: ^1^H NMR spectrum of the EtOAc extract of *Penicillium rubens* MABC05 in DMSO-*d*_6_ (400 MHz); Figures S2−S37, S43−S60, S74−S82: 1D and 2D NMR, and HRESIMS spectra of **1**–**6, 10, 10a** and **10b**; Figures S38−S42, S61−S73: ^1^H and ^13^CNMR spectra of **7**–**9** and **11**–**17**; Tables S1–S5 Calculated NMR data of **2** and **10**, Tables S6–S12: Optimized cartesian coordinates of **2**–**6** and **10**, and details for biological study.
