# Supplementary material for: Drimane-Pyrone-Type Meroterpenoids and Co-Isolated Compounds from the Deep-Sea Fungus Penicillium rubens MABC05 and Their Anti-Ferroptotic and Cytotoxic Activities
Source: Mar Drugs. 2026 Sep 17;24(9):326. doi: 10.3390/md24090326 (PMC13608478; doi:10.3390/md24090326)
Supplement: Supplementary file 1 [file marinedrugs-24-00326-s001.zip › marinedrugs-4541704-supplementary.pdf]

No.	Content	Page
**Figure S1:**	^{1}H NMR spectrum of the EtOAc extract of *Penicillium rubens* MABC05 in DMSO-d_6 (400 MHz)	3
**Figure S2:**	^{1}H NMR spectrum of **1** in in $CDCl_3$ (500 MHz)	4
**Figure S3:**	^{13}C NMR spectrum of **1** in $CDCl_3$ (75.5 MHz)	4
**Figure S4:**	HSQC spectrum of **1** in $CDCl_3$	5
**Figure S5:**	COSY spectrum of **1** in $CDCl_3$	5
**Figure S6:**	HMBC spectrum of **1** in $CDCl_3$	6
**Figure S7:**	NOESY spectrum of **1** in $CDCl_3$	6
**Figure S8:**	^{1}H NMR spectrum of **2** in in $CDCl_3$ (600 MHz)	7
**Figure S9:**	^{13}C NMR spectrum of **2** in $CDCl_3$ (151 MHz)	7
**Figure S10:**	HSQC spectrum of **2** in $CDCl_3$	8
**Figure S11:**	COSY spectrum of **2** in $CDCl_3$	8
**Figure S12:**	HMBC spectrum of **2** in $CDCl_3$	9
**Figure S13:**	NOESY spectrum of **2** in $CDCl_3$	9
**Figure S14:**	^{1}H NMR spectrum of **3** in $CDCl_3$ (500 MHz)	10
**Figure S15:**	^{13}C NMR spectrum of **3** in $CDCl_3$ (126 MHz)	10
**Figure S16:**	HSQC spectrum of **3** in $CDCl_3$	11
**Figure S17:**	COSY spectrum of **3** in $CDCl_3$	11
**Figure S18:**	HMBC spectrum of **3** in $CDCl_3$	12
**Figure S19:**	NOESY spectrum of **3** in $CDCl_3$	12
**Figure S20:**	^{1}H NMR spectrum of **4** in $CDCl_3$ (500 MHz)	13
**Figure S21:**	^{13}C NMR spectrum of **4** in $CDCl_3$ (126 MHz)	13
**Figure S22:**	HSQC spectrum of **4** in $CDCl_3$	14
**Figure S23:**	COSY spectrum of **4** in $CDCl_3$	14
**Figure S24:**	HMBC spectrum of **4** in $CDCl_3$	15
**Figure S25:**	NOESY spectrum of **4** in $CDCl_3$	15
**Figure S26:**	^{1}H NMR spectrum of **5** in $CDCl_3$ (500 MHz)	16
**Figure S27:**	^{13}C NMR spectrum of **5** in $CDCl_3$ (126 MHz)	16
**Figure S28:**	HSQC spectrum of **5** in $CDCl_3$	17
**Figure S29:**	COSY spectrum of **5** in $CDCl_3$	17
**Figure S30:**	HMBC spectrum of **5** in $CDCl_3$	18
**Figure S31:**	NOESY spectrum of **5** in $CDCl_3$	18
**Figure S32:**	^{1}H NMR spectrum of **6** in $CDCl_3$ (600 MHz)	19
**Figure S33:**	^{13}C NMR spectrum of **6** in $CDCl_3$ (151 MHz)	19
**Figure S34:**	HSQC spectrum of **6** in $CDCl_3$	20
**Figure S35:**	COSY spectrum of **6** in $CDCl_3$	20
**Figure S36:**	HMBC spectrum of **6** in $CDCl_3$	21

**Figure S37:**	NOESY spectrum of **6** in $CDCl_3$	21
**Figure S38:**	^{1}H NMR spectrum of **7** in $CDCl_3$ (400 MHz)	22
**Figure S39:**	^{13}C NMR spectrum of **7** in in $CDCl_3$ (101 MHz)	22
**Figure S40:**	^{1}H NMR spectrum of **8** in $CDCl_3$ (300 MHz)	23
**Figure S41:**	^{13}C NMR spectrum of **8** in $CDCl_3$ (75.5 MHz)	23
**Figure S42:**	^{1}H NMR spectrum of **9** in $CDCl_3$ (300 MHz)	24
**Figure S43:**	^{1}H NMR spectrum of **10** in $CDCl_3$ (500 MHz)	24
**Figure S44:**	^{13}C NMR spectrum of **10** in $CDCl_3$ (75.5 MHz)	25
**Figure S45:**	HSQC spectrum of **10** in $CDCl_3$	25
**Figure S46:**	COSY spectrum of **10** in $CDCl_3$	26
**Figure S47:**	HMBC spectrum of **10** in in $CDCl_3$	26
**Figure S48:**	NOESY spectrum of **10** in in $CDCl_3$	27
**Figure S49:**	^{1}H NMR spectrum of **10a** in $CDCl_3$ (600 MHz)	27
**Figure S50:**	^{13}C NMR spectrum of **10a** in $CDCl_3$ (151 MHz)	28
**Figure S51:**	HSQC spectrum of **10a** in $CDCl_3$	28
**Figure S52:**	COSY spectrum of **10a** in $CDCl_3$	29
**Figure S53:**	HMBC spectrum of **10a** in in $CDCl_3$	29
**Figure S54:**	NOESY spectrum of **10a** in in $CDCl_3$	30
**Figure S55:**	^{1}H NMR spectrum of **10b** in $CDCl_3$ (600 MHz)	30
**Figure S56:**	^{13}C NMR spectrum of **10b** in $CDCl_3$ (151 MHz)	31
**Figure S57:**	HSQC spectrum of **10b** in $CDCl_3$	31
**Figure S58:**	COSY spectrum of **10b** in $CDCl_3$	32
**Figure S59:**	HMBC spectrum of **10b** in $CDCl_3$	32
**Figure S60:**	NOESY spectrum of **10b** in $CDCl_3$	33
**Figure S61:**	^{1}H NMR spectrum of **11** in $CDCl_3$ (300 MHz)	33
**Figure S62:**	^{13}C NMR spectrum of **11** in $CDCl_3$ (126 MHz)	34
**Figure S63:**	^{1}H NMR spectrum of **12** in $CDCl_3$ (400 MHz)	34
**Figure S64:**	^{13}C NMR spectrum of **12** in $CDCl_3$ (151 MHz)	35
**Figure S65:**	^{1}H NMR spectrum of **13** in $CDCl_3$ (300 MHz)	35
**Figure S66:**	^{13}C NMR spectrum of **13** in $CDCl_3$ (75.5 MHz)	36
**Figure S67:**	^{1}H NMR spectrum of **14** in $CDCl_3$ (400 MHz)	36
**Figure S68:**	^{1}H NMR spectrum of **15** in $CDCl_3$ (400 MHz)	37
**Figure S69:**	^{13}C NMR spectrum of **15** in $CDCl_3$ (126 MHz)	37
**Figure S70:**	^{1}H NMR spectrum of **16** in $CDCl_3$ (300 MHz)	38
**Figure S71:**	^{13}C NMR spectrum of **16** in $CDCl_3$ (126 MHz)	38
**Figure S72:**	^{1}H NMR spectrum of **17** in $CDCl_3$ (500 MHz)	39
**Figure S73:**	^{13}C NMR spectrum of **17** in $CDCl_3$ (126 MHz)	39
**Figure S74:**	HRESIMS spectrum of compound **1**	40
**Figure S75:**	HRESIMS spectrum of compound **2**	40
**Figure S76:**	HRESIMS spectrum of compound **3**	41
**Figure S77**	HRESIMS spectrum of compound **4**	41
**Figure S78**	HRESIMS spectrum of compound **5**	42
**Figure S79**	HRESIMS spectrum of compound **6**	42
**Figure S80**	HRESIMS spectrum of compound **10**	43
**Figure S81**	HRESIMS spectrum of compound **10a**	43
**Figure S82**	HRESIMS spectrum of compound **10b**	44
**Table S1**	Shielding sensors (σ_C) of the conformer (**2**) and the calculated chemical shifts (δ_C).	45
**Table S2**	DP4$^+$ probability analysis for isomers 2I and 2II	45

**Table S3**	Shielding sensors (σ_C) of the conformer (**10**I) and the calculated chemical shifts (δ_C).	46
**Table S4**	Shielding sensors (σ_C) of the conformer (**10**II) and the calculated chemical shifts (δ_C).	47
**Table S5**	DP4$^+$ probability analysis for isomers **10**I and **10**II	48
**Table S6**	Optimized cartesian coordinates of compound **1** for ECD calculation at the b3lyp/6-31+g(d,p) level	49
**Table S7**	Optimized cartesian coordinates of compound **2** for ECD calculation at the b3lyp/6-31+g(d,p) level.	50
**Table S8**	Optimized cartesian coordinates of compound **3** for ECD calculation at the b3lyp/6-31+g(d,p) level	51
**Table S9**	Optimized cartesian coordinates of compound **4** for ECD calculation at the b3lyp/6-31+g(d,p) level	52
**Table S10**	Optimized cartesian coordinates of compound **5** for ECD calculation at the b3lyp/6-31+g(d,p) level	53
**Table S11**	Optimized cartesian coordinates of compound **6** for ECD calculation at the b3lyp/6-31+g(d,p) level	54
**Table S12**	Optimized cartesian coordinates of compound **10** for ECD calculation at the b3lyp/6-31+g(d,p) level	55-57
	Details for Biological Assay	58-59

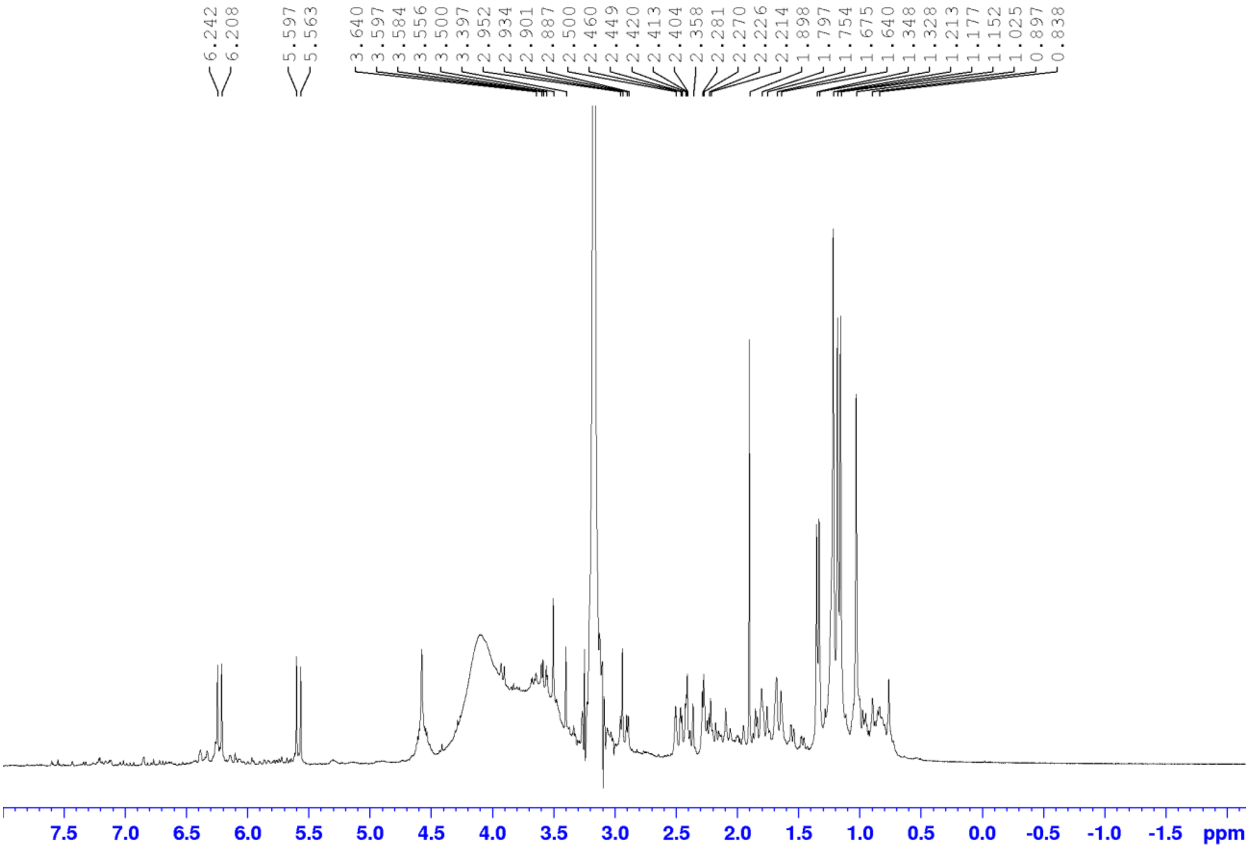

Figure S1: ^{1}H NMR spectrum of the EtOAc extract of *Penicillium rubens* MABC05 in DMSO-d_6 (400 MHz)

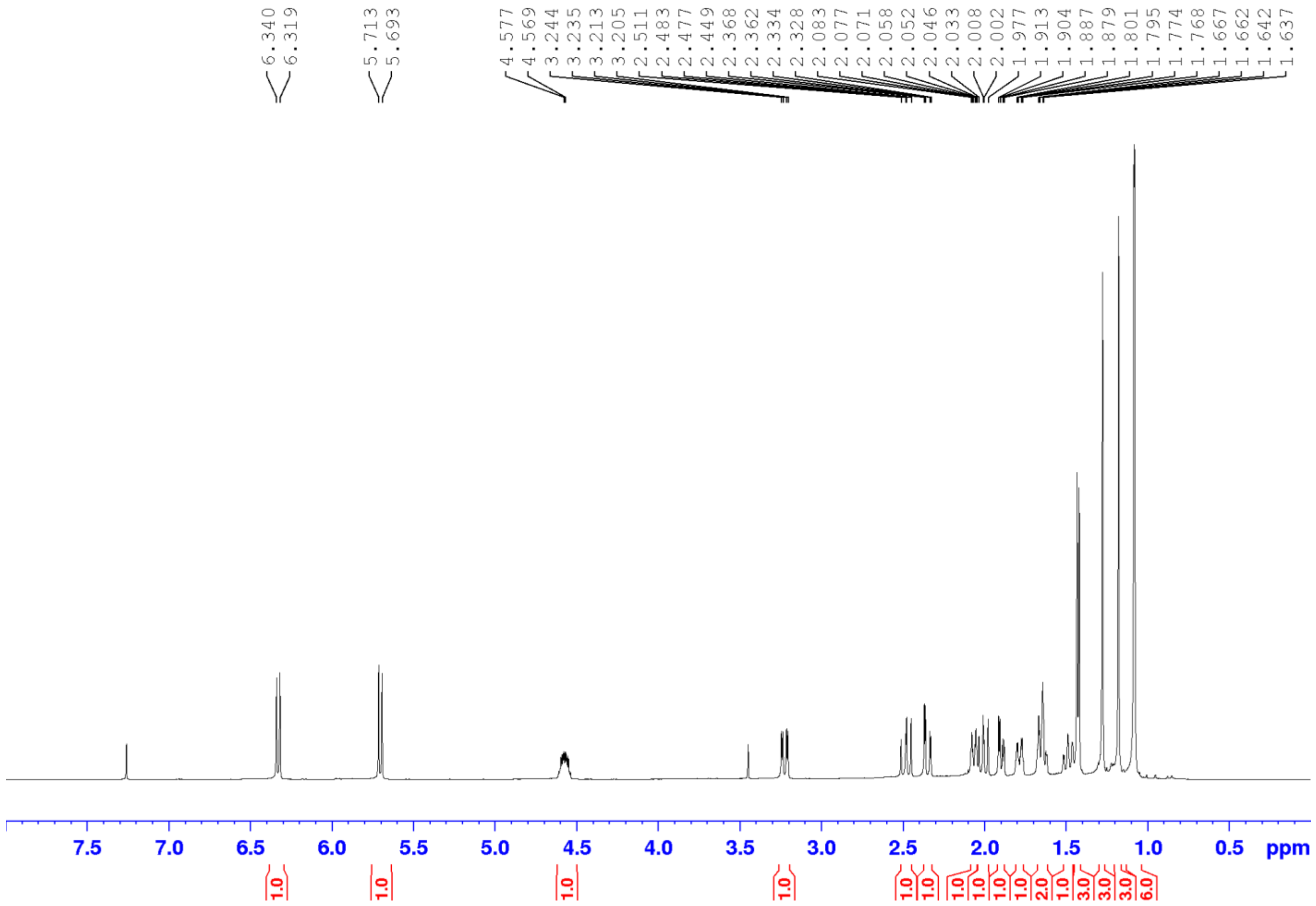

Figure S2: ^{1}H NMR spectrum of **1** in in $CDCl_3$ (500 MHz)

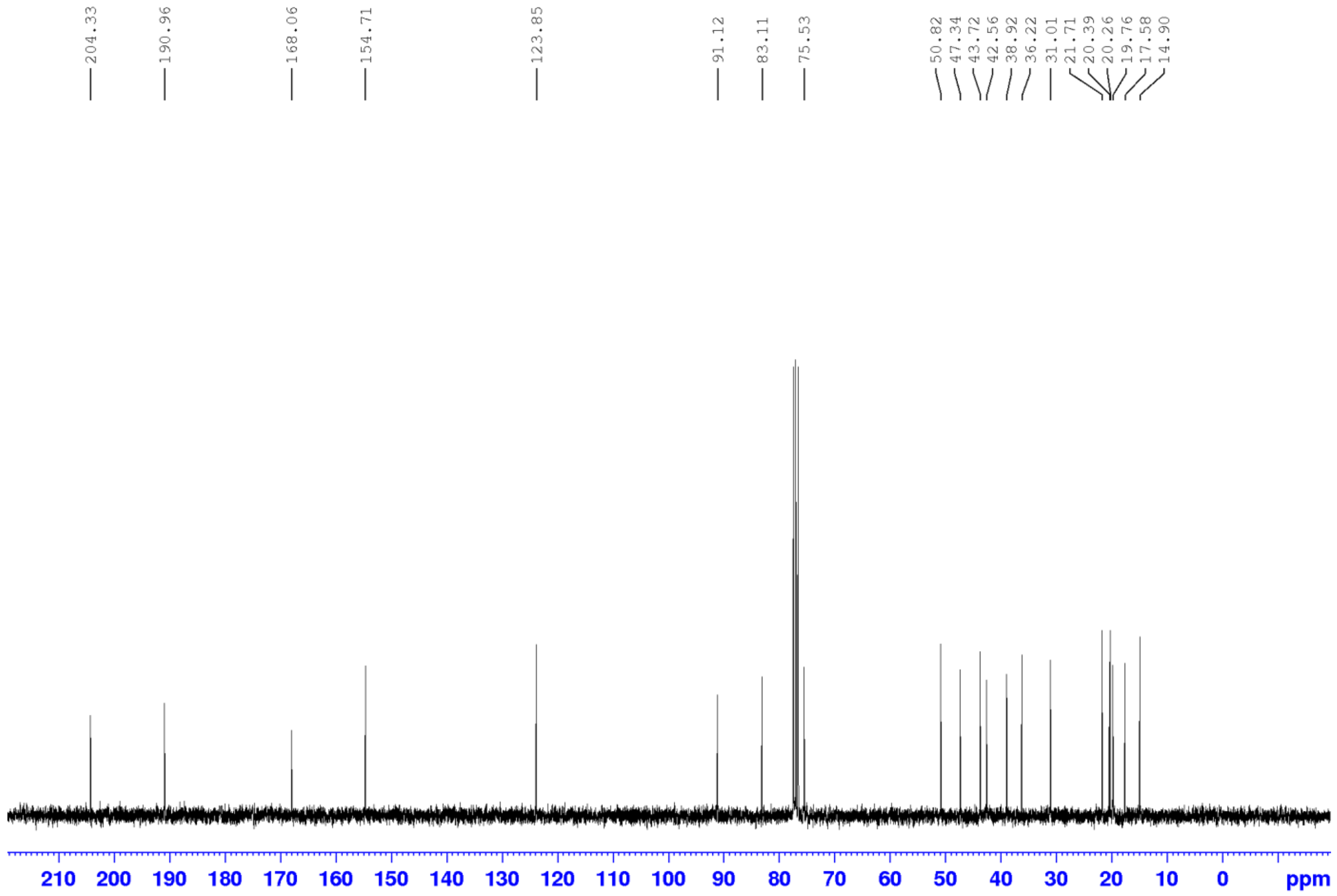

Figure S3: ^{13}C NMR spectrum of **1** in $CDCl_3$ (75.5 MHz)

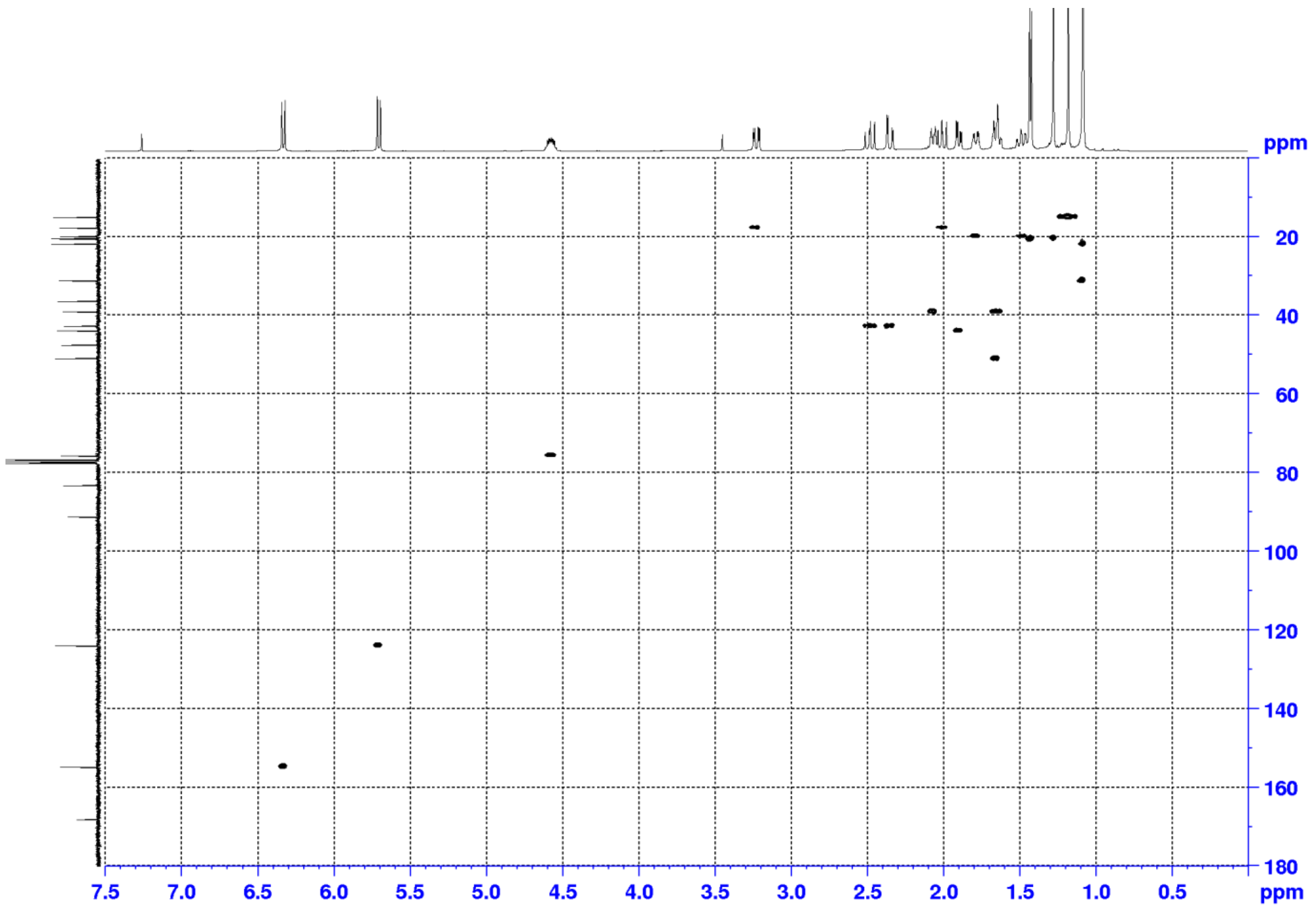

Figure S4: HSQC spectrum of **1** in $CDCl_3$

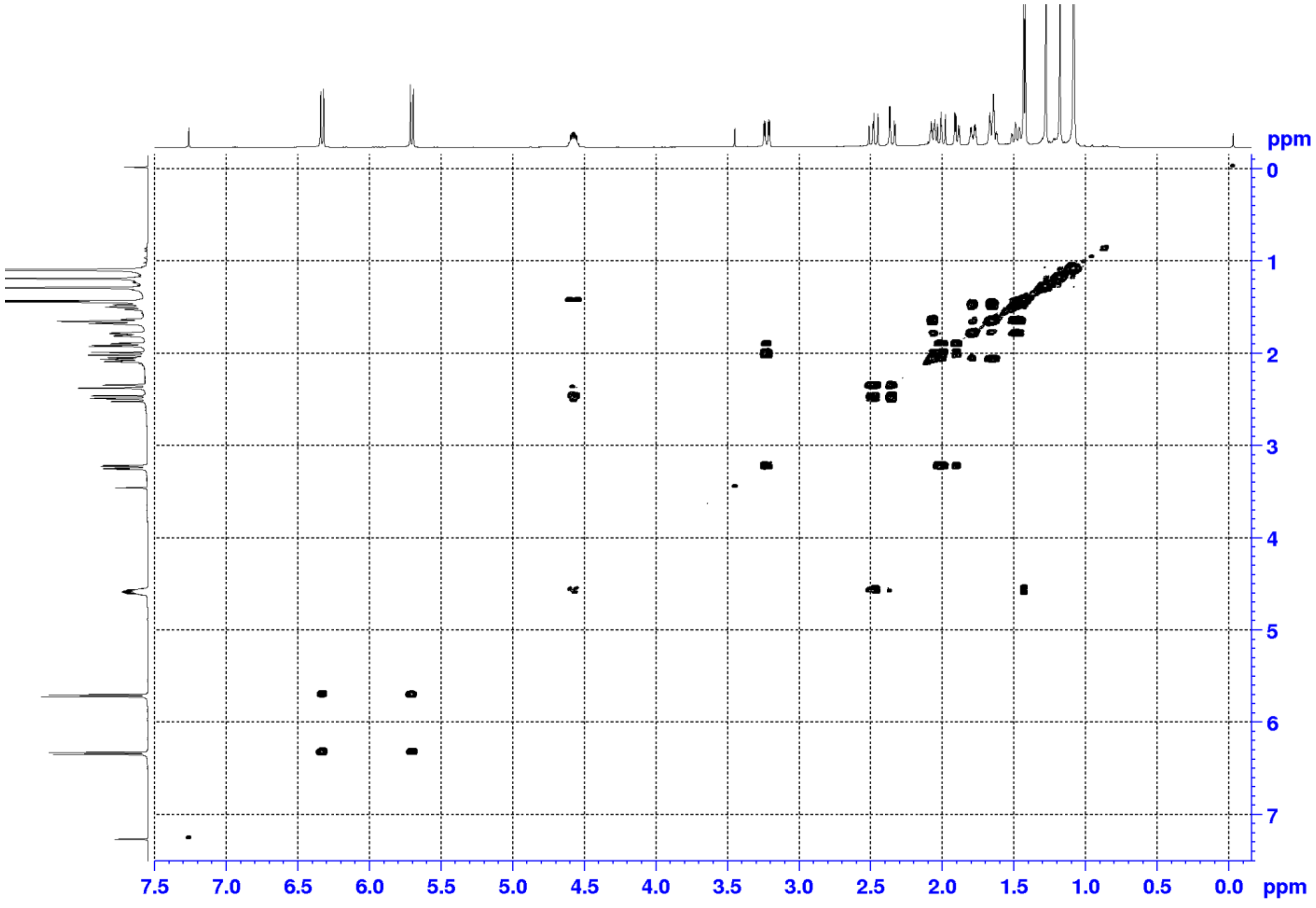

Figure S5: COSY spectrum of **1** in $CDCl_3$

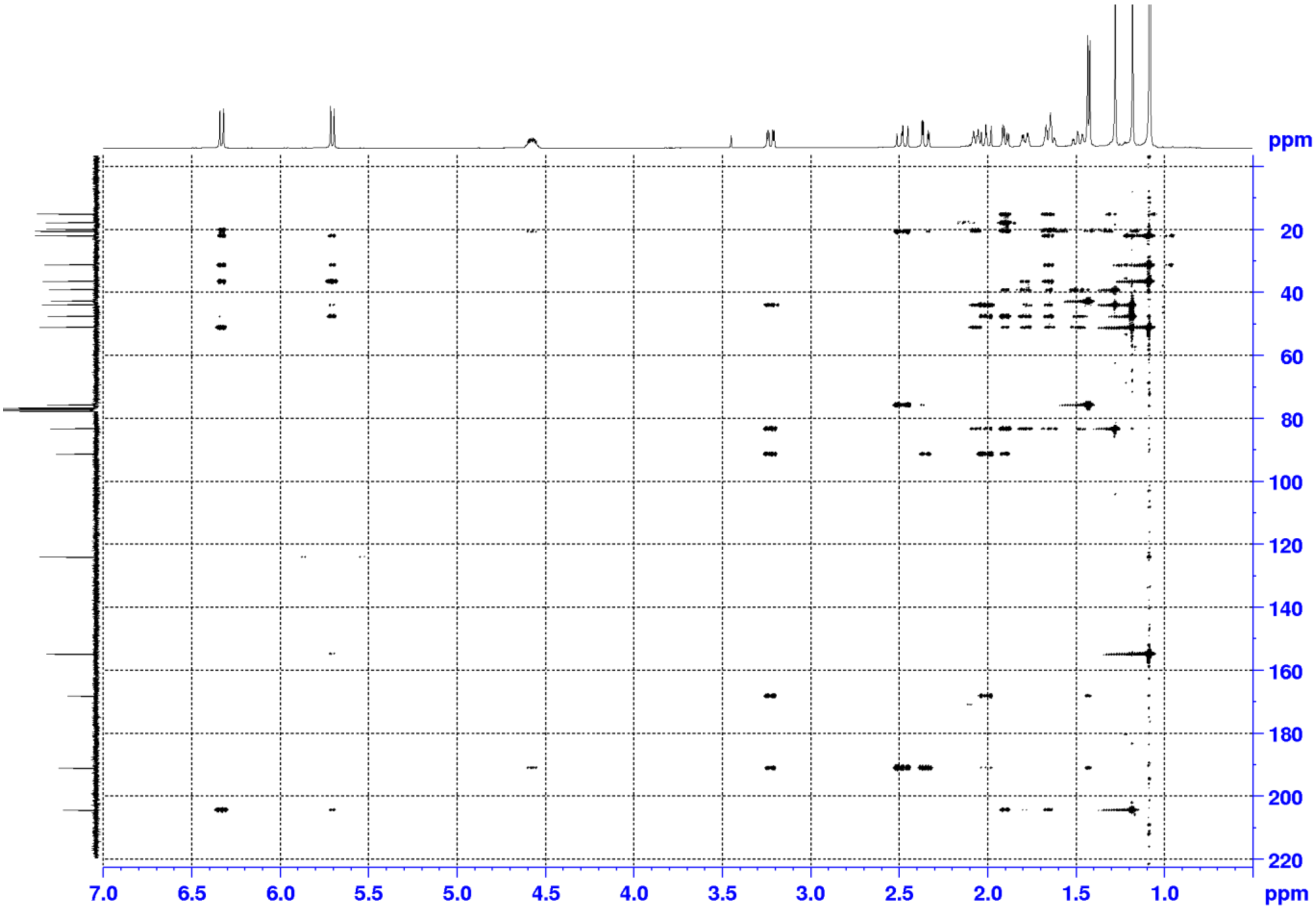

Figure S6: HMBC spectrum of **1** in $CDCl_3$

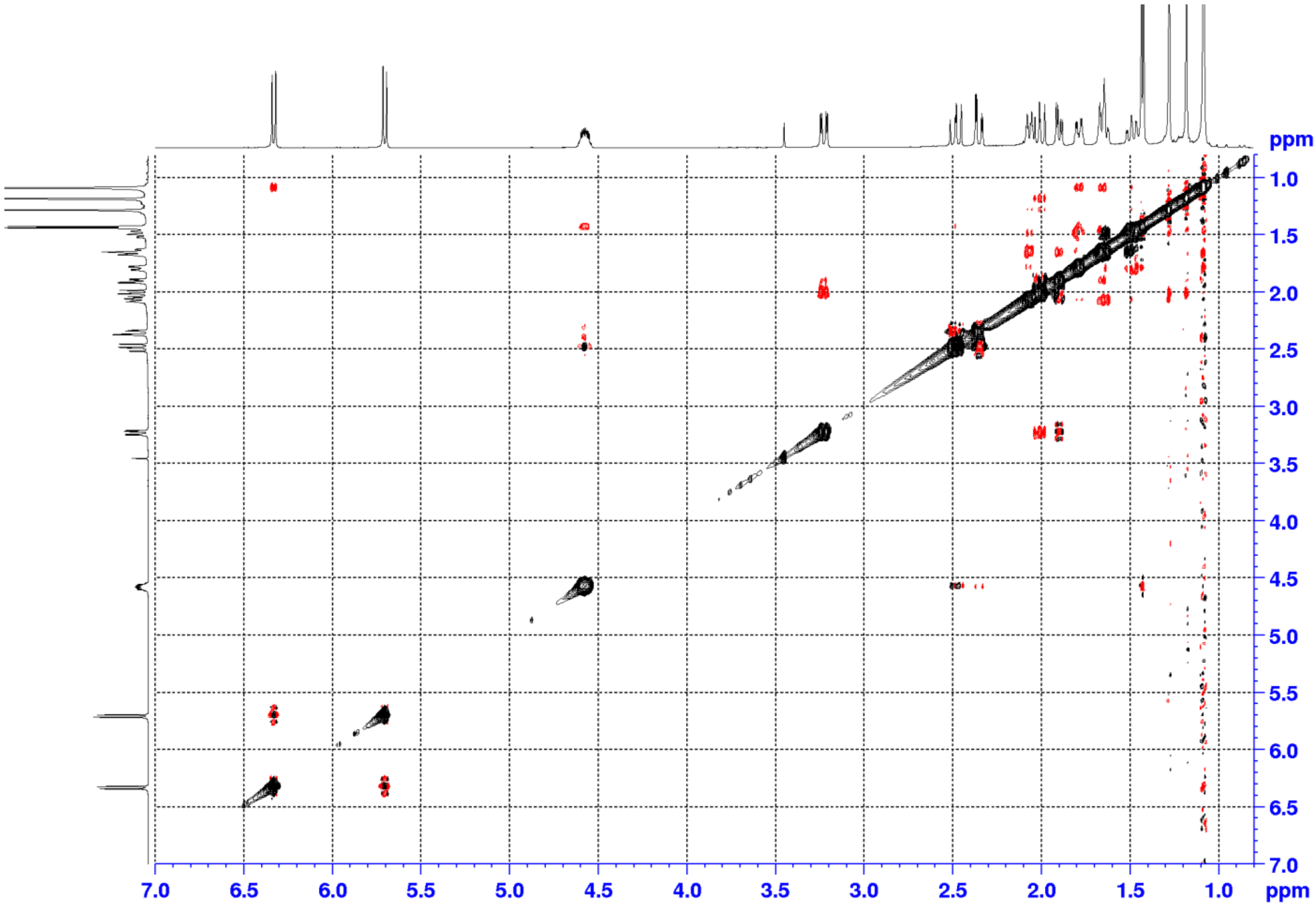

Figure S7: NOESY spectrum of **1** in $CDCl_3$

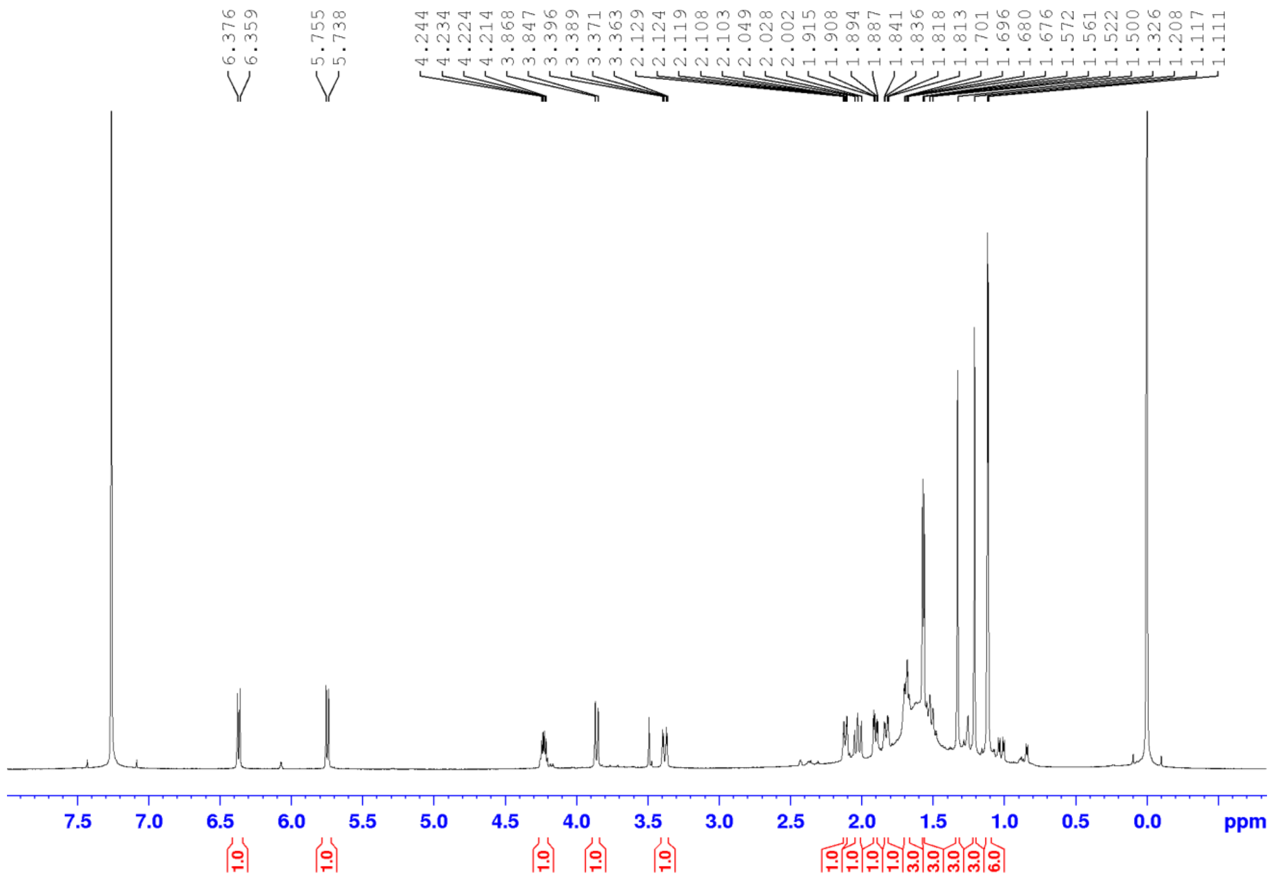

Figure S8: ^{1}H NMR spectrum of **2** in in $CDCl_3$ (600 MHz)

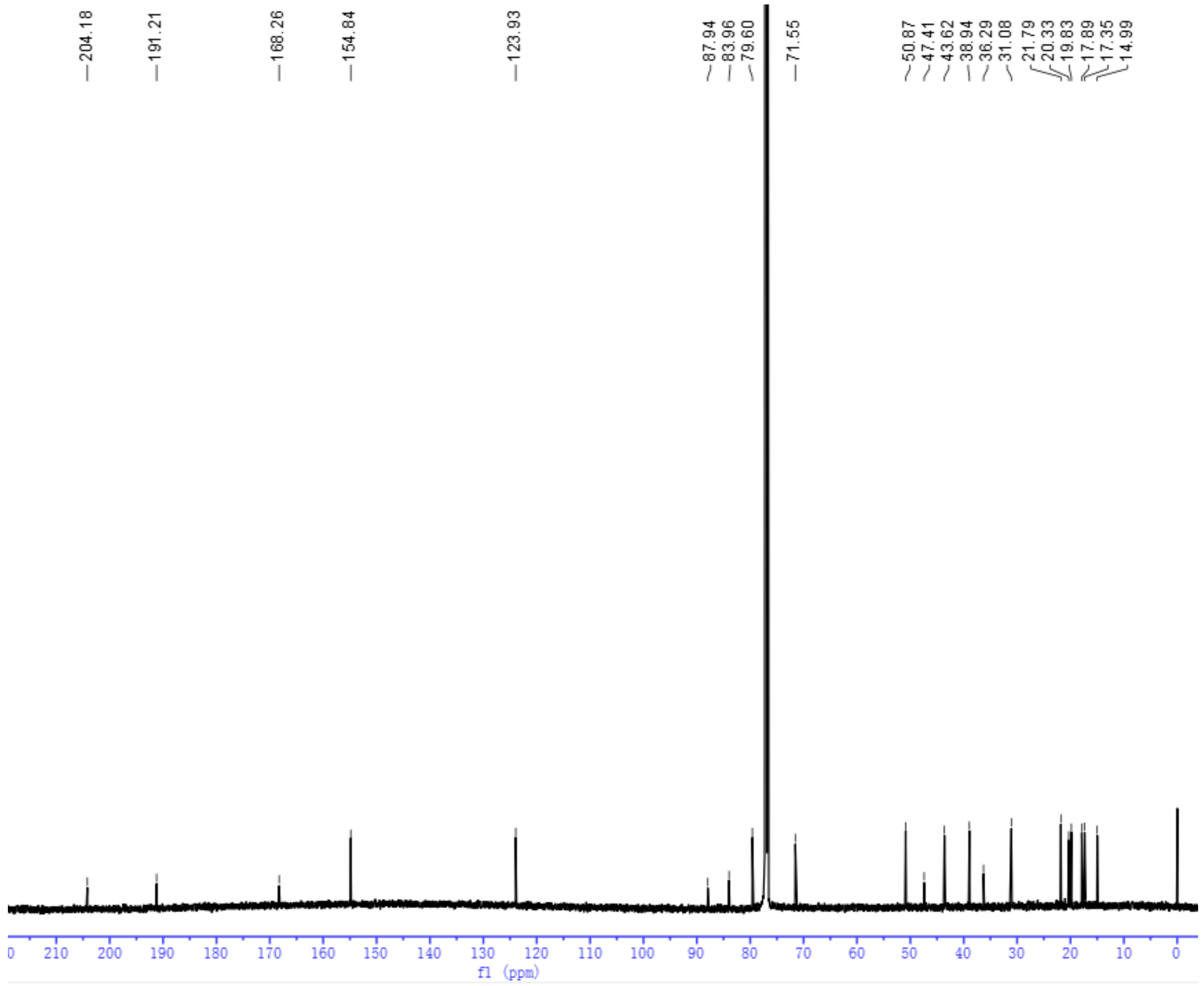

Figure S9: ^{13}C NMR spectrum of **2** in $CDCl_3$ (151 MHz)

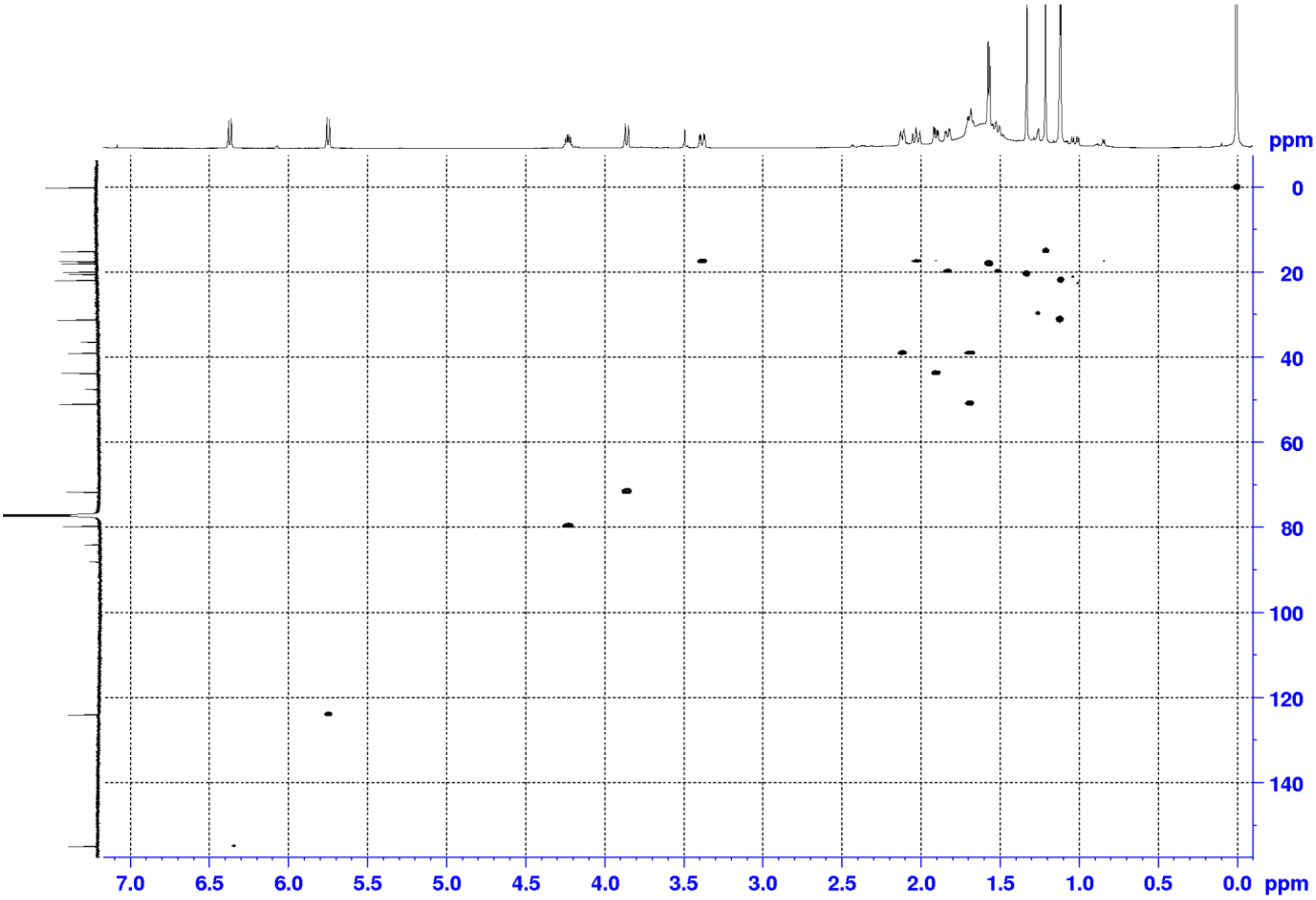

Figure S10: HSQC spectrum of **2** in $CDCl_3$

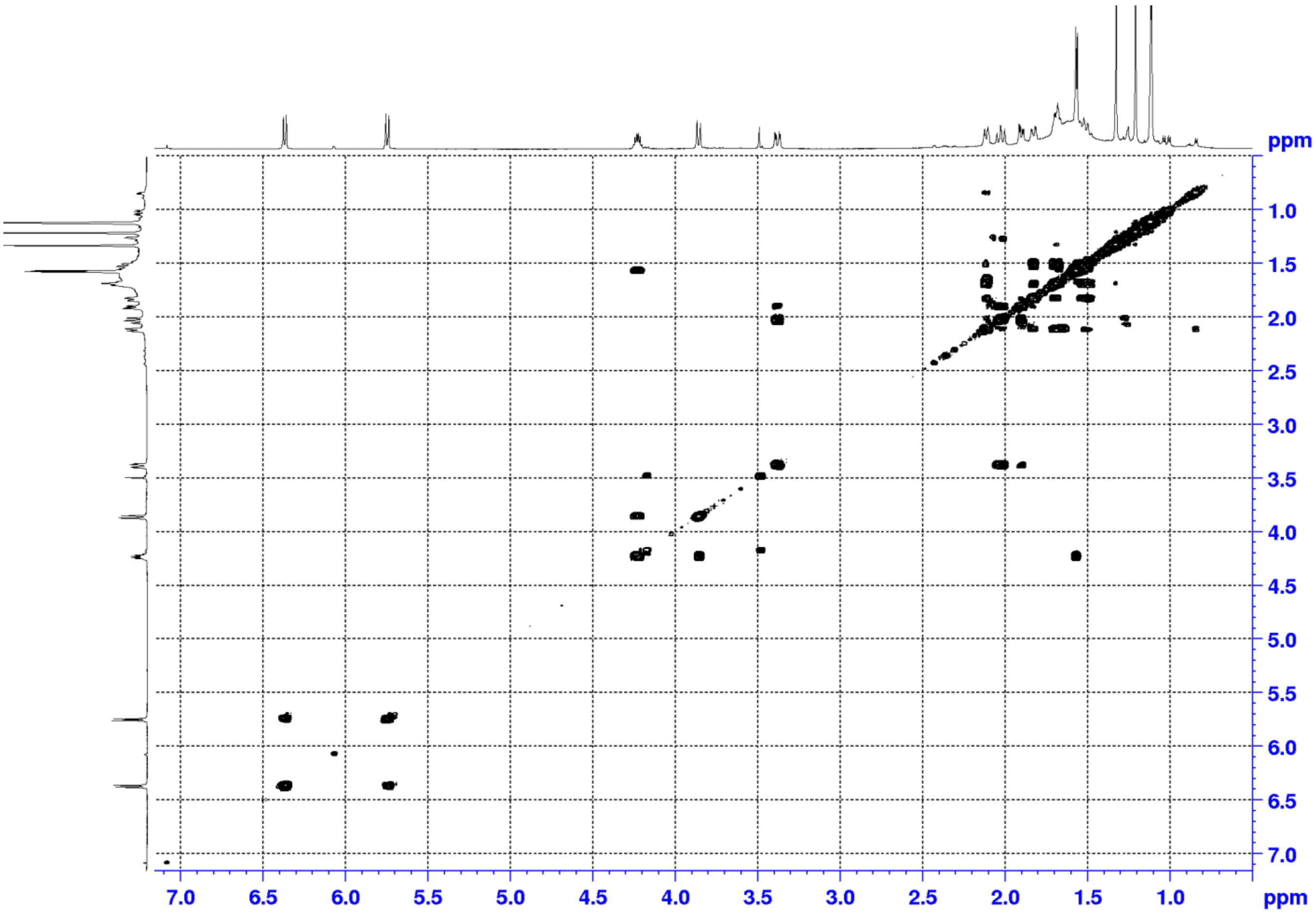

Figure S11: COSY spectrum of **2** in $CDCl_3$

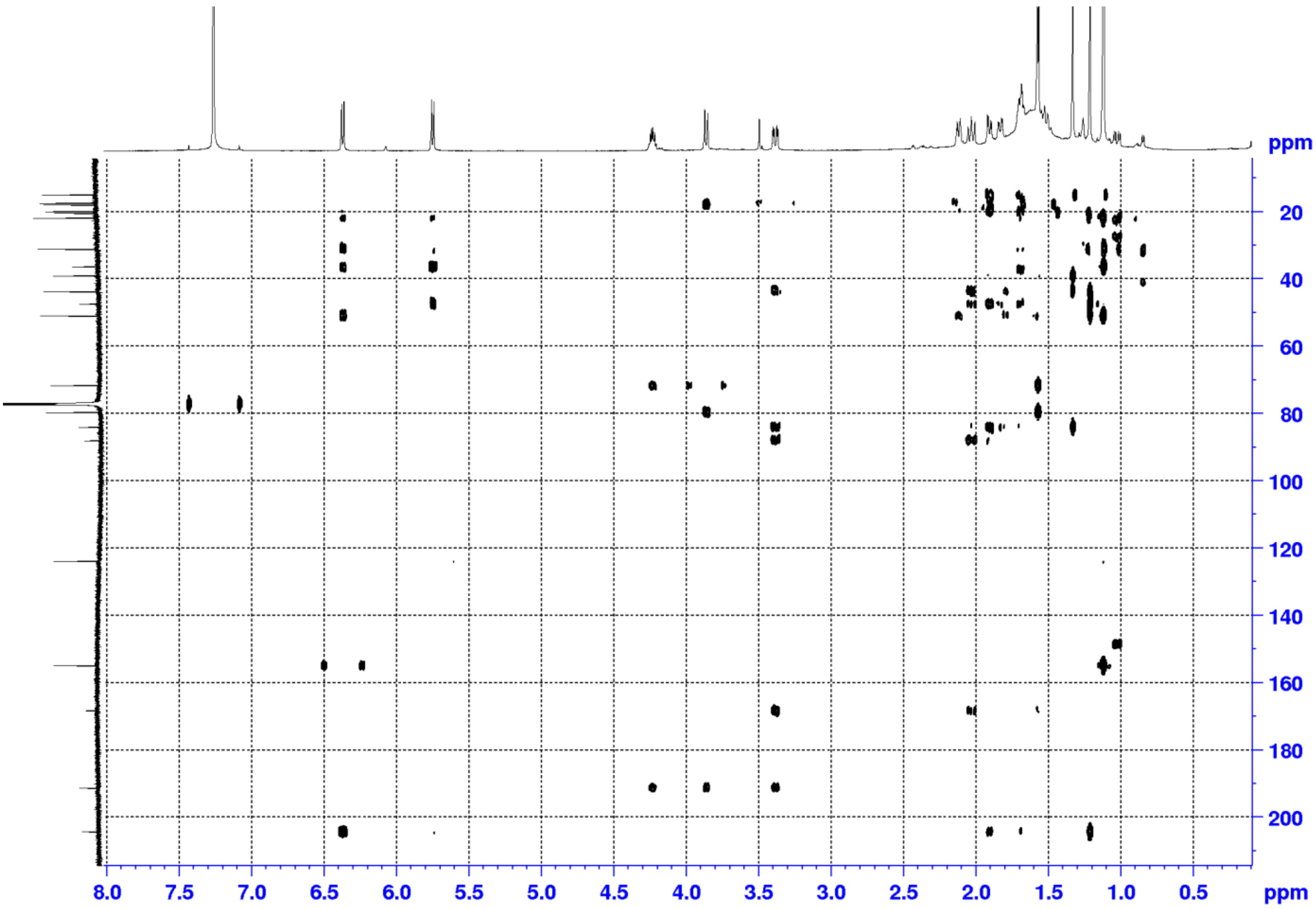

Figure S12: HMBC spectrum of **2** in $CDCl_3$

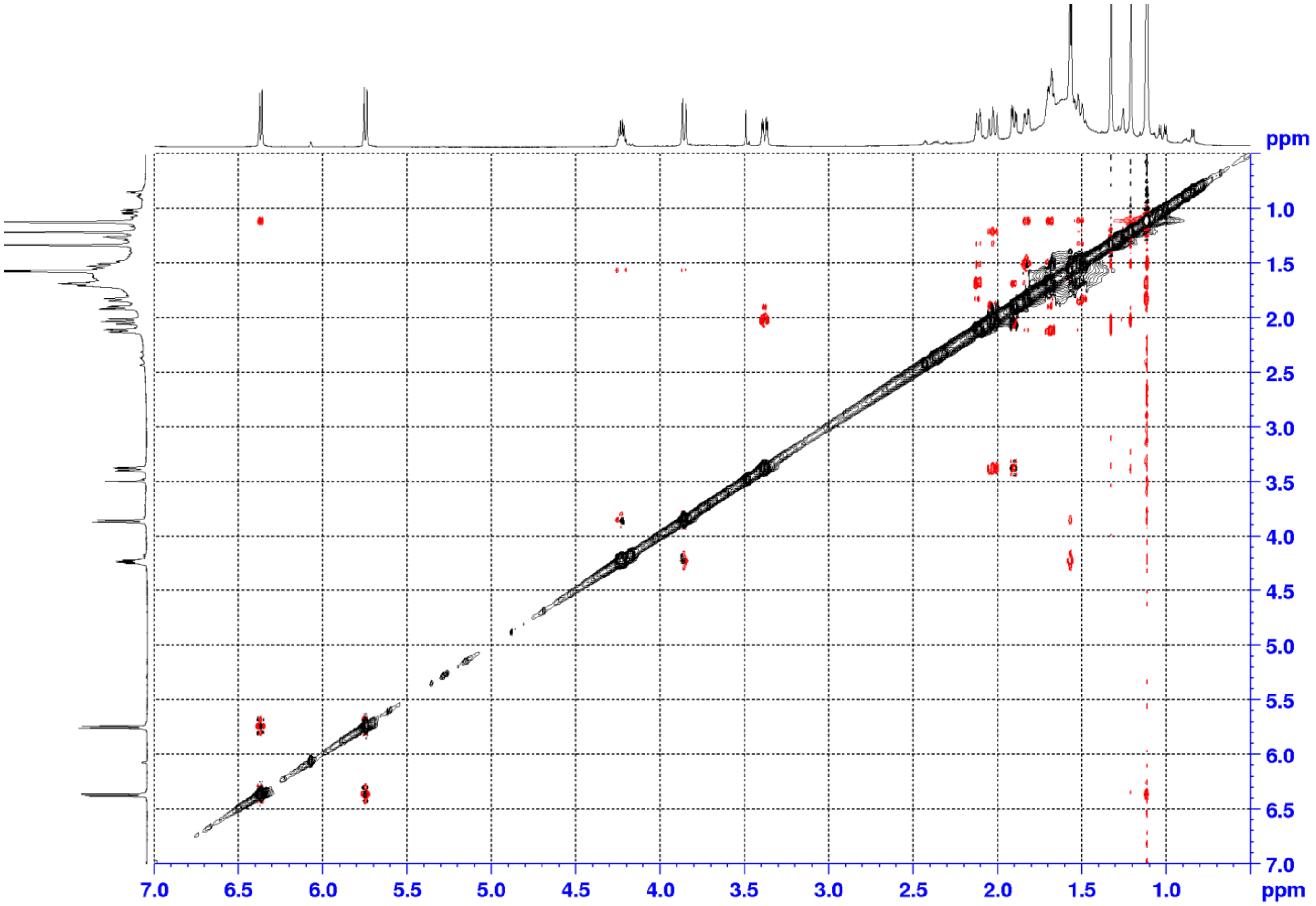

Figure S13: NOESY spectrum of **2** in $CDCl_3$

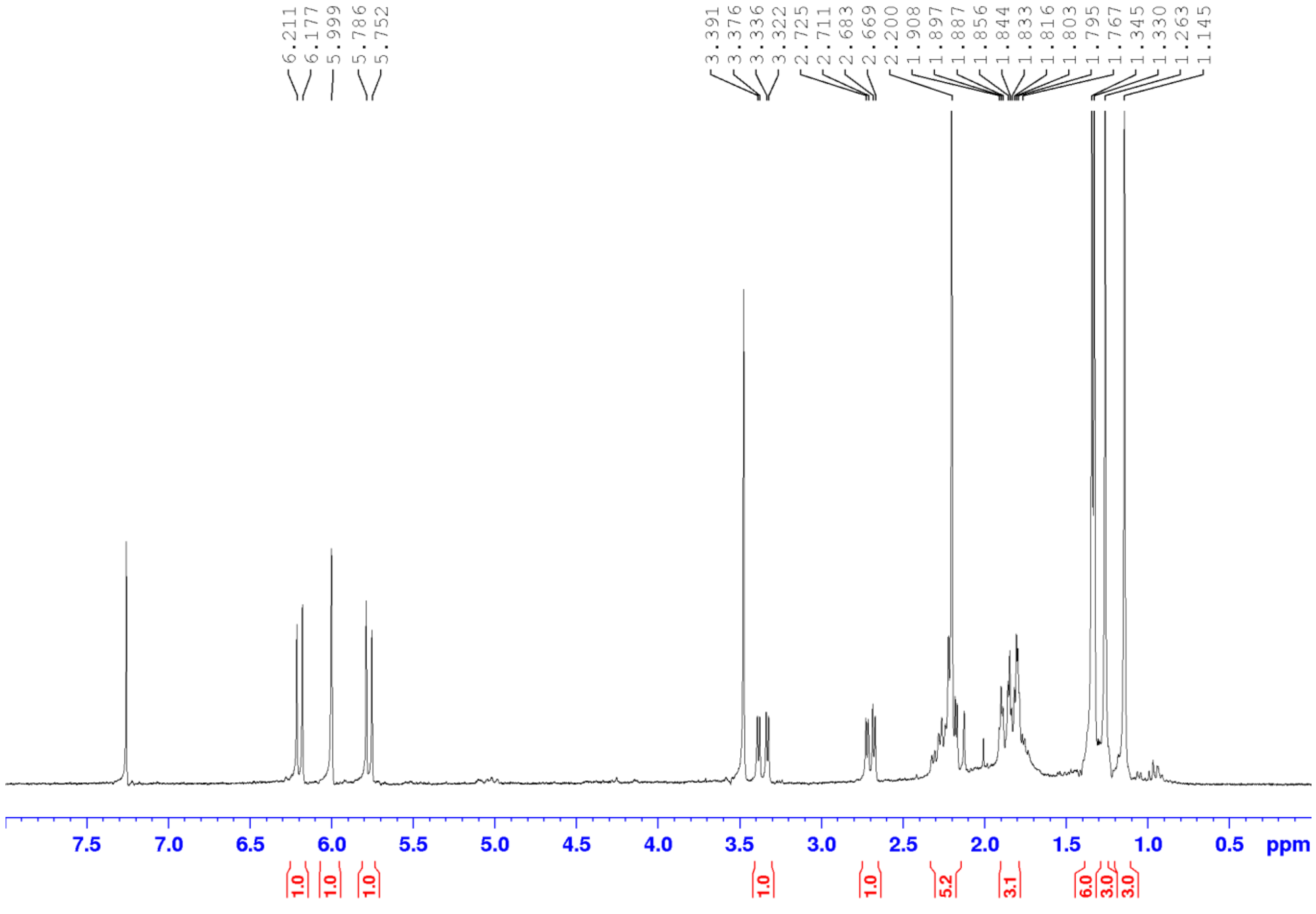

Figure S14: ^{1}H NMR spectrum of **3** in $CDCl_3$ (500 MHz)

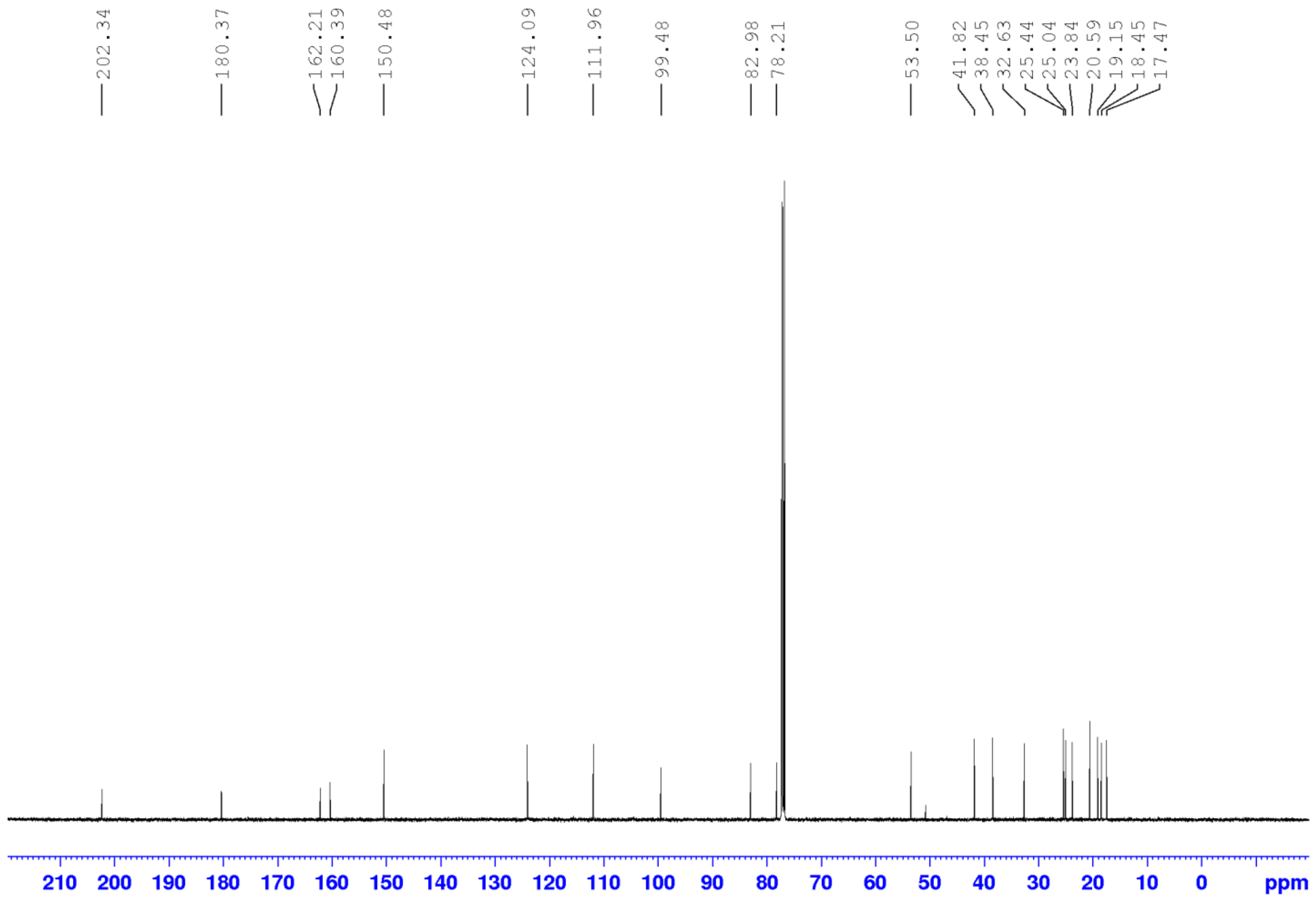

Figure S15: ^{13}C NMR spectrum of **3** in $CDCl_3$ (126 MHz)

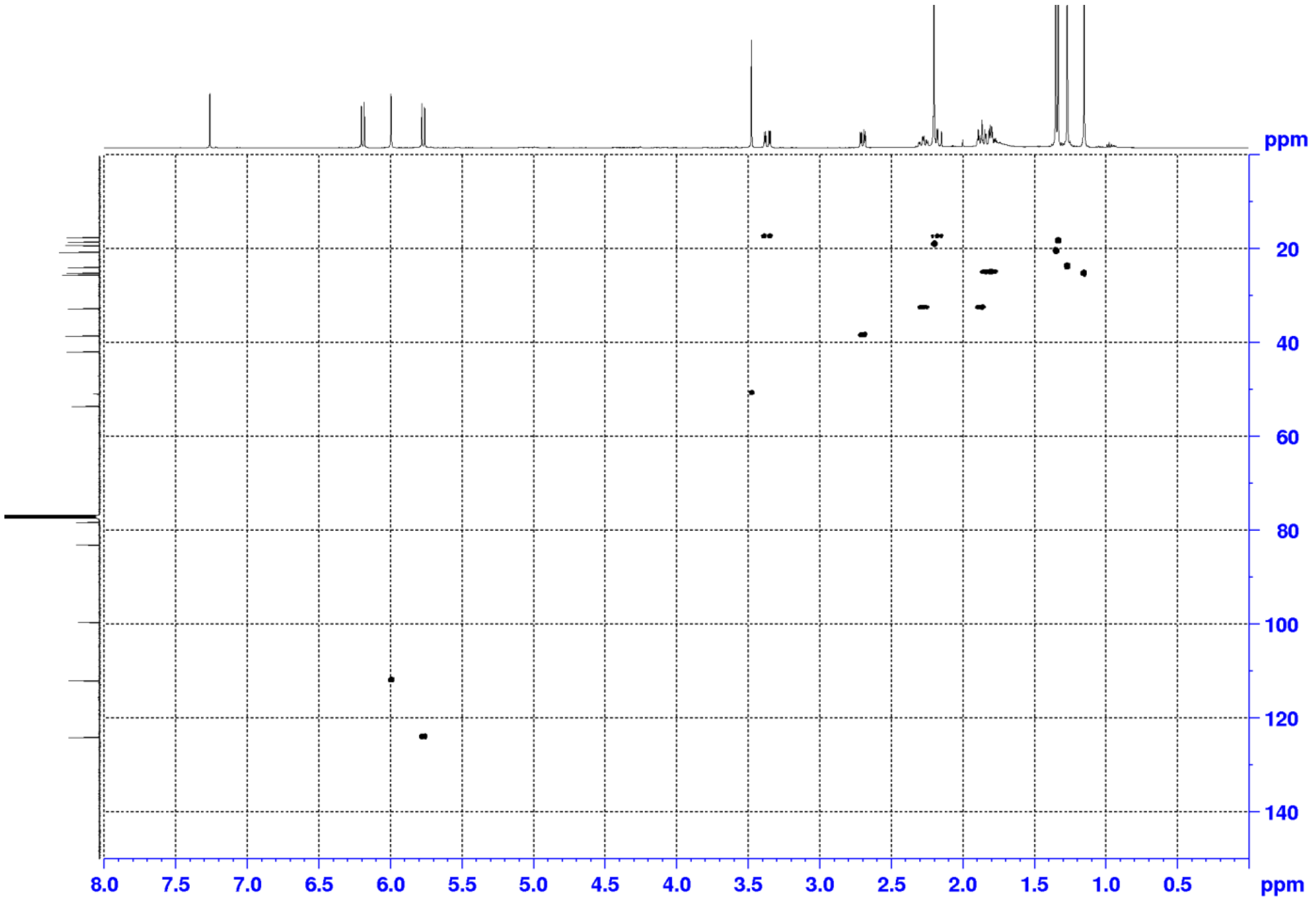

Figure S16: HSQC spectrum of **3** in $CDCl_3$

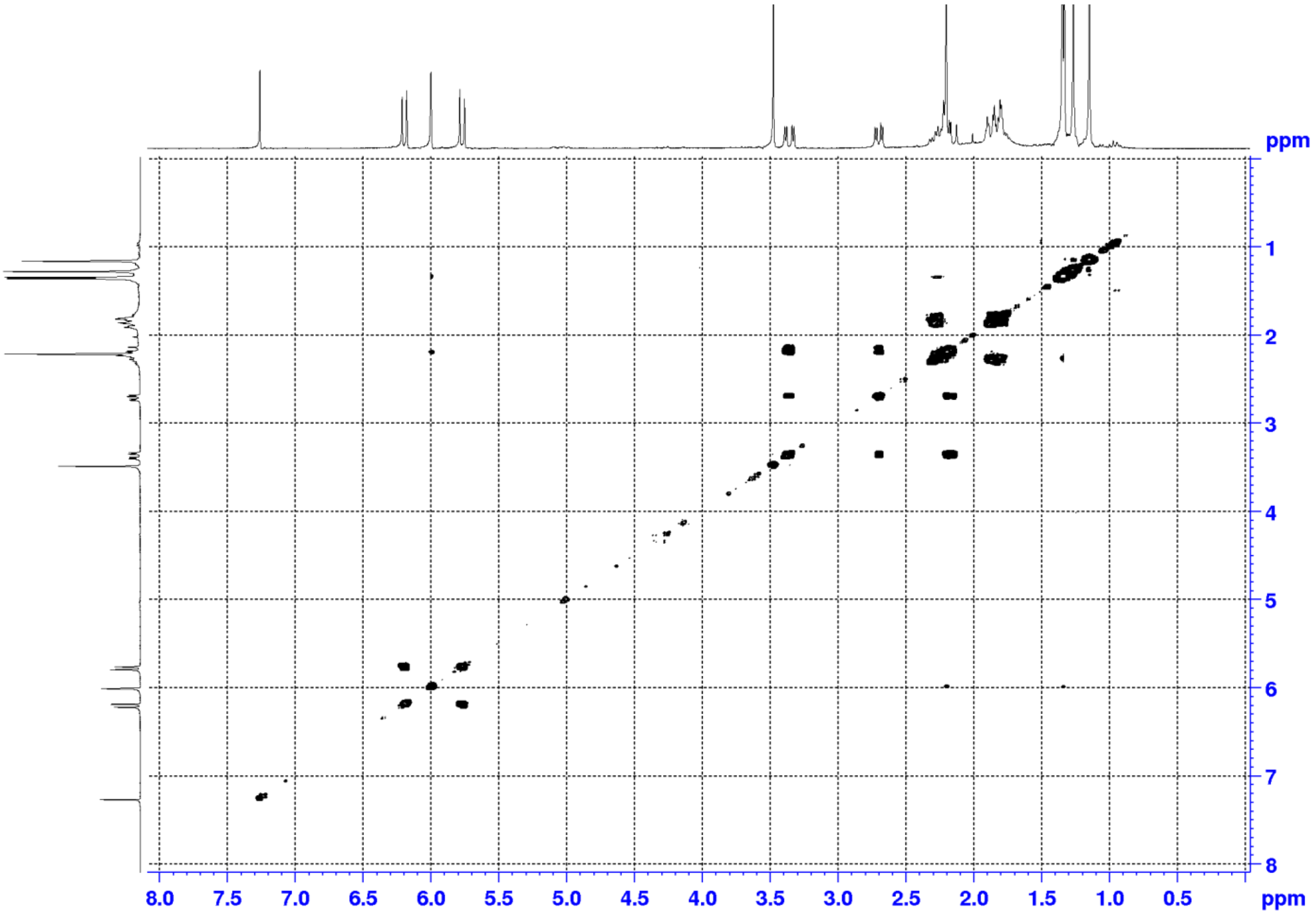

Figure S17: COSY spectrum of **3** in $CDCl_3$

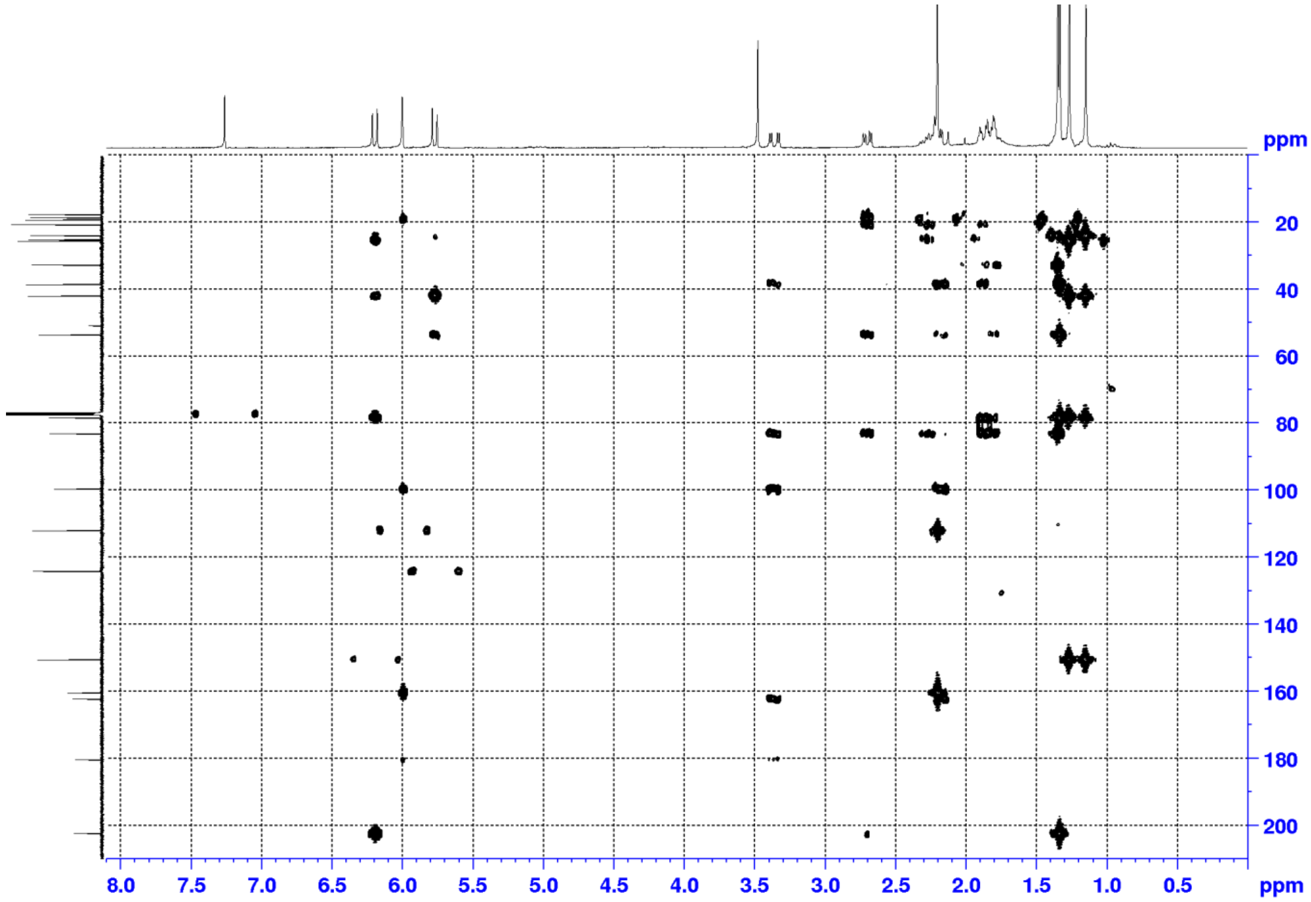

Figure S18: HMBC spectrum of **3** in $CDCl_3$

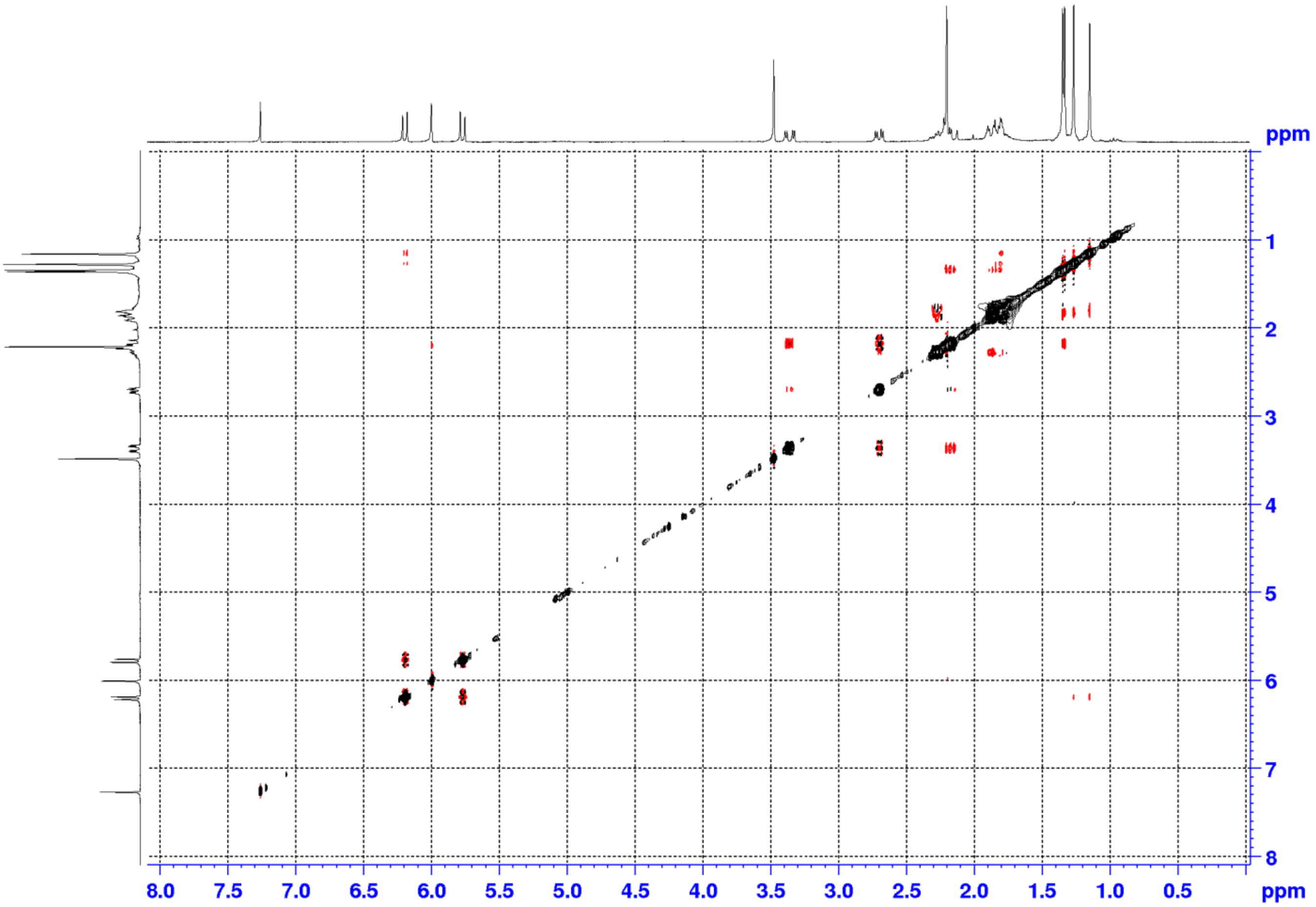

Figure S19: NOESY spectrum of **3** in $CDCl_3$

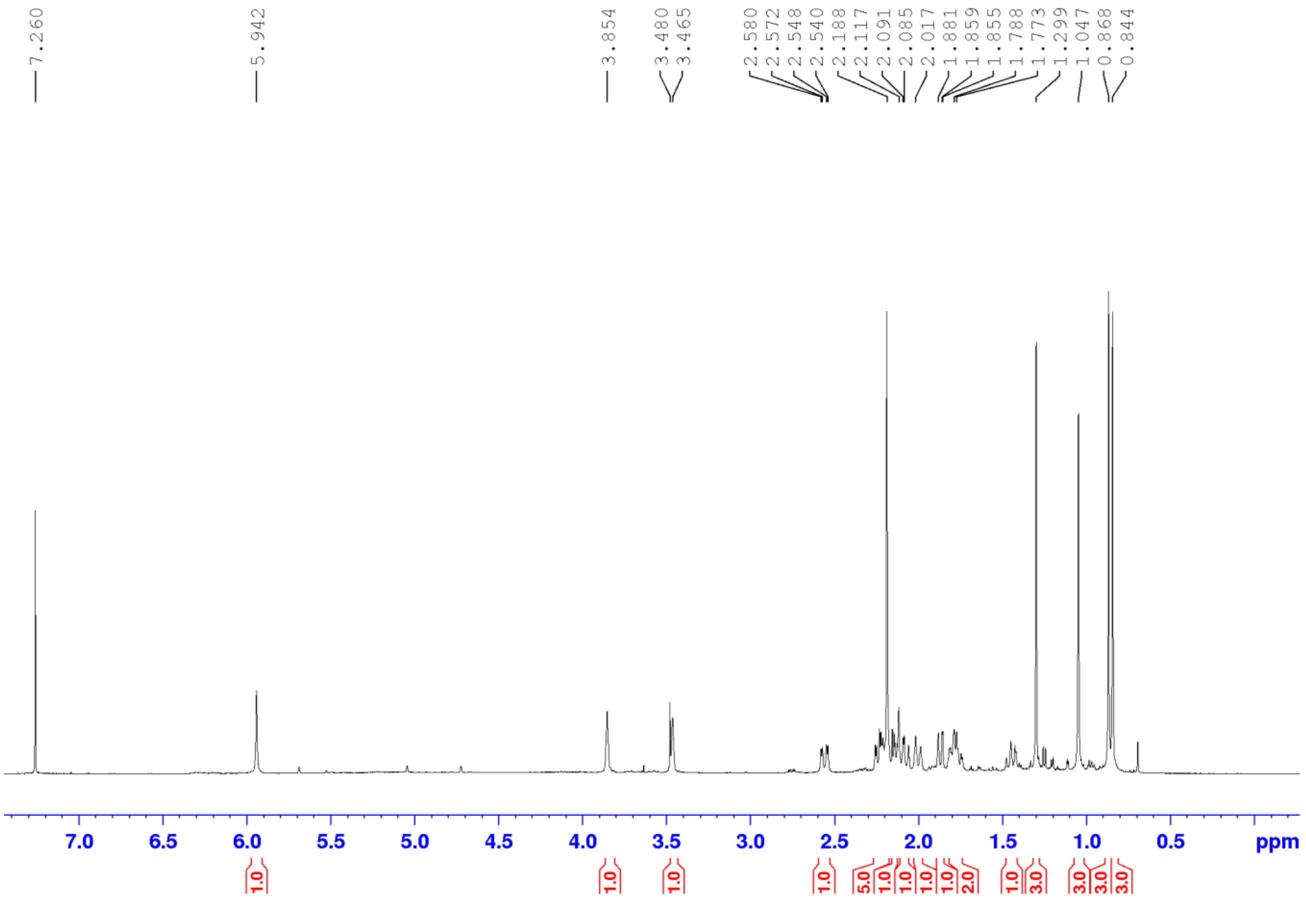

Figure S20: ^{1}H NMR spectrum of **4** in $CDCl_3$ (500 MHz)

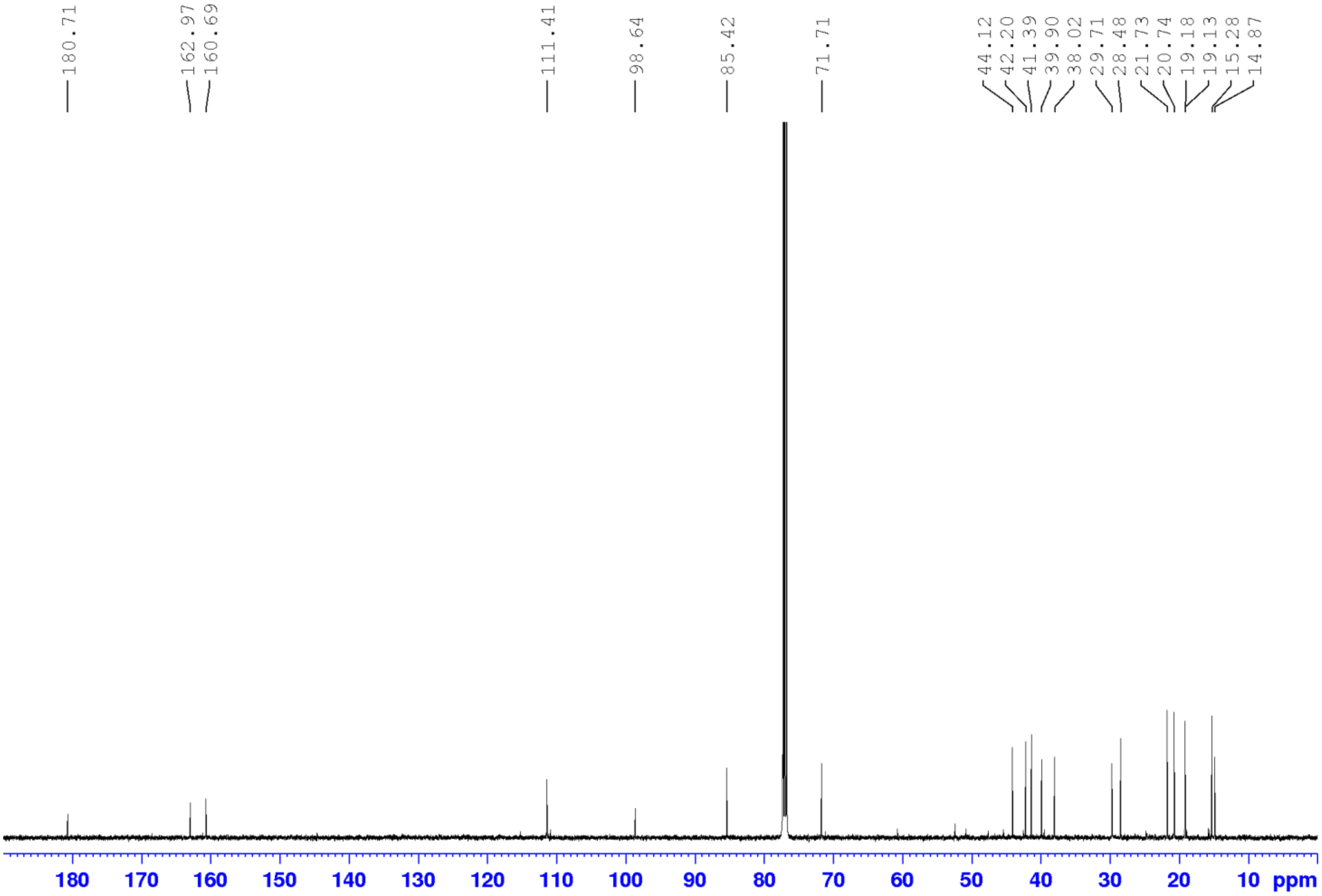

Figure S21: ^{13}C NMR spectrum of **4** in $CDCl_3$ (126 MHz)

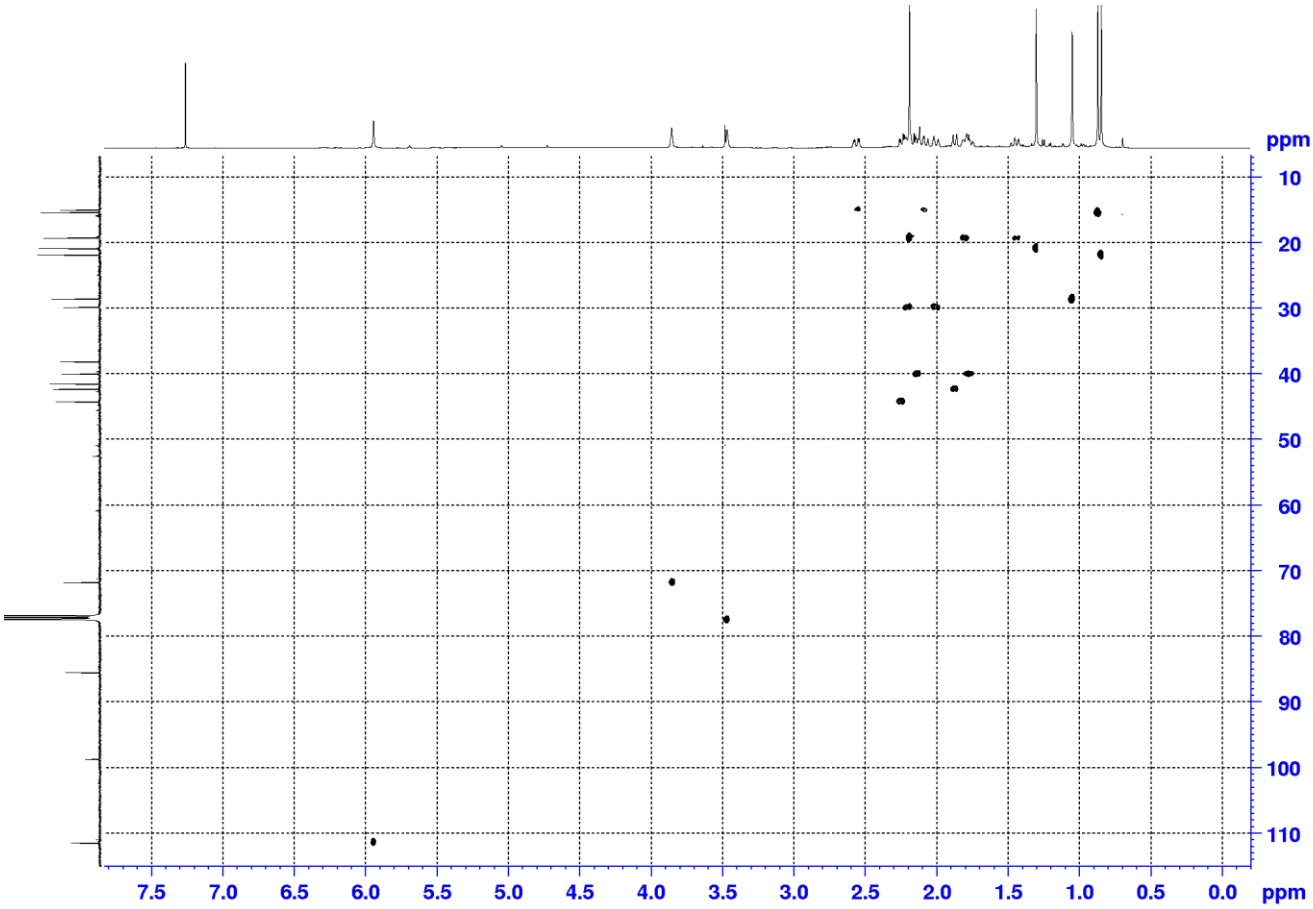

Figure S22: HSQC spectrum of **4** in $CDCl_3$

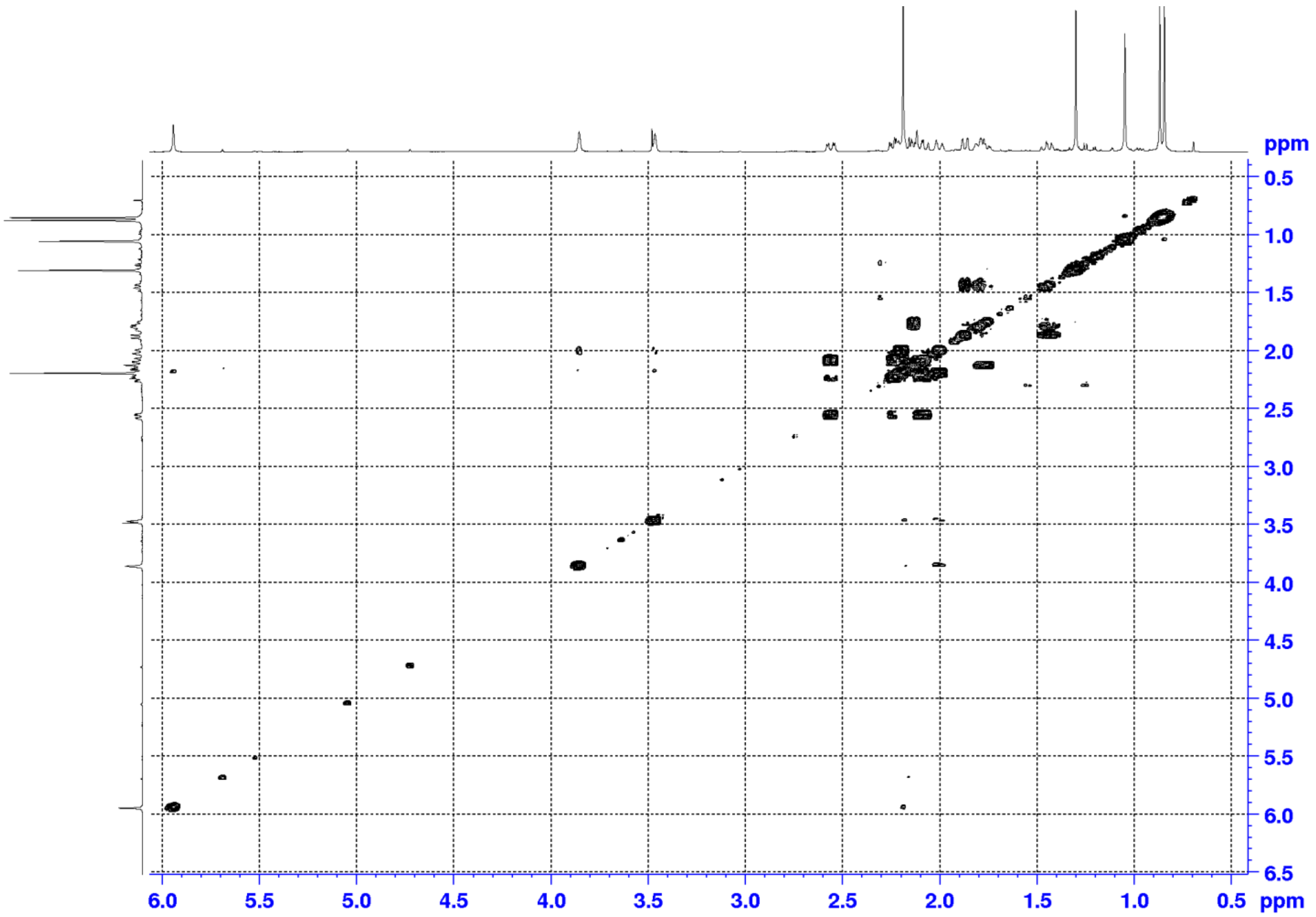

Figure S23: COSY spectrum of **4** in $CDCl_3$

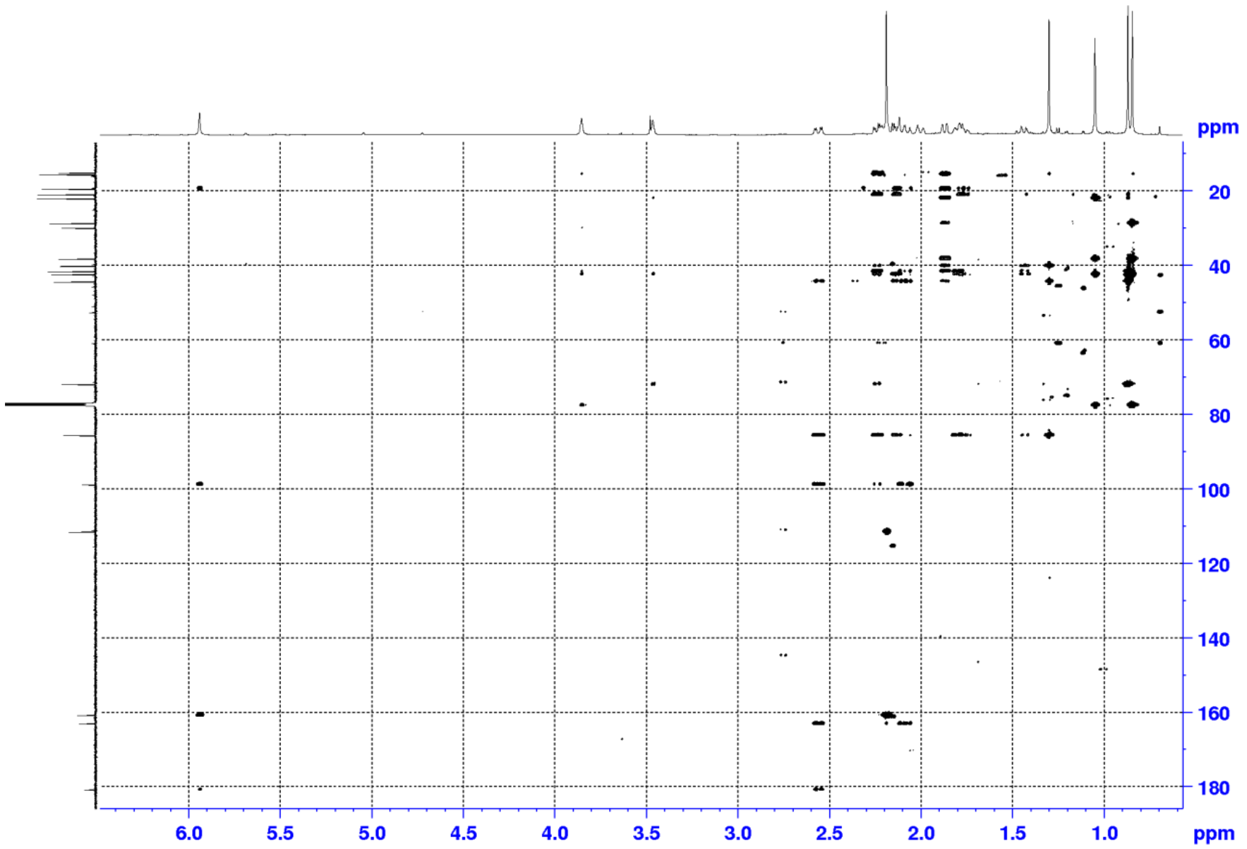

Figure S24: HMBC spectrum of **4** in $CDCl_3$

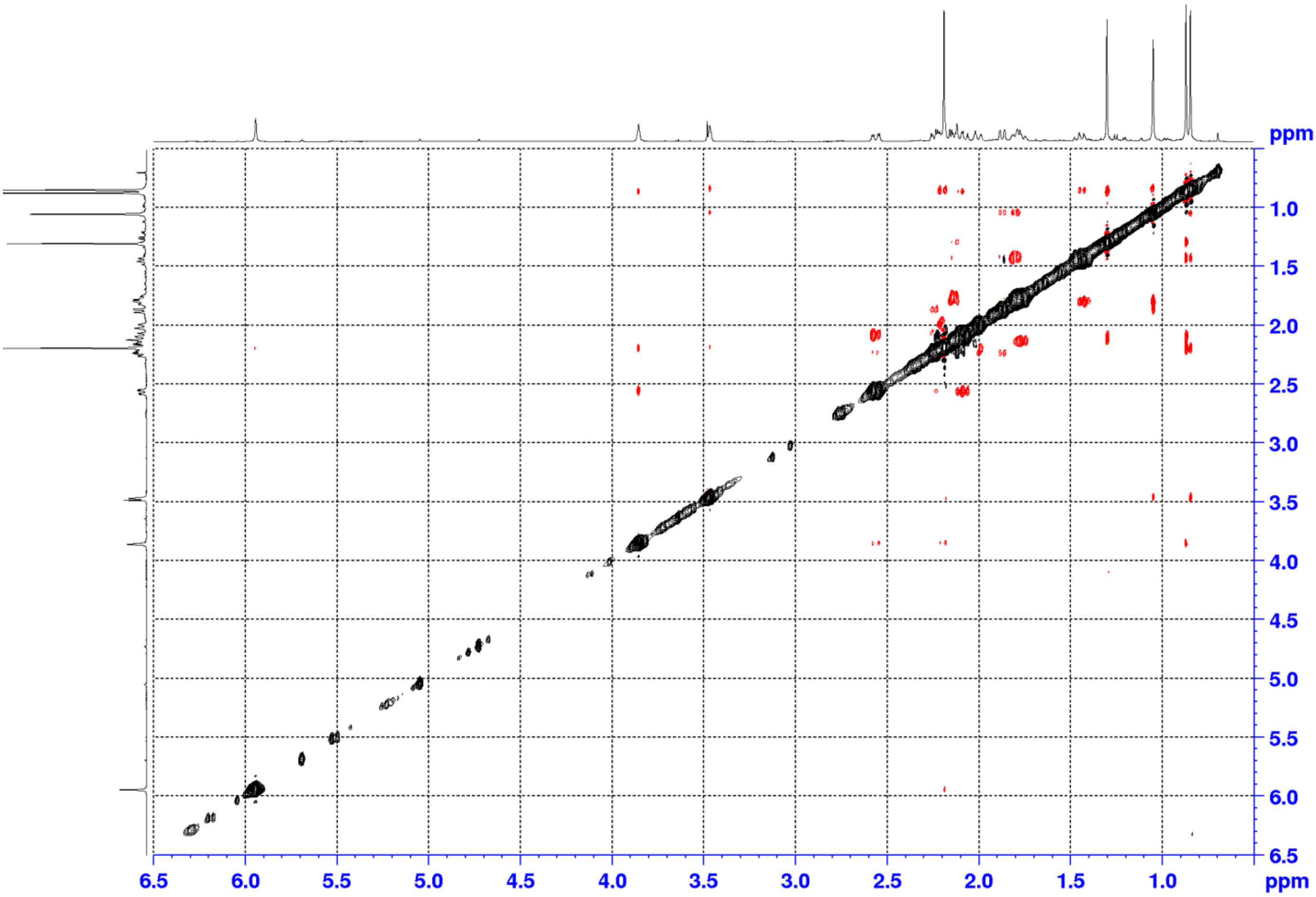

Figure S25: NOESY spectrum of **4** in $CDCl_3$

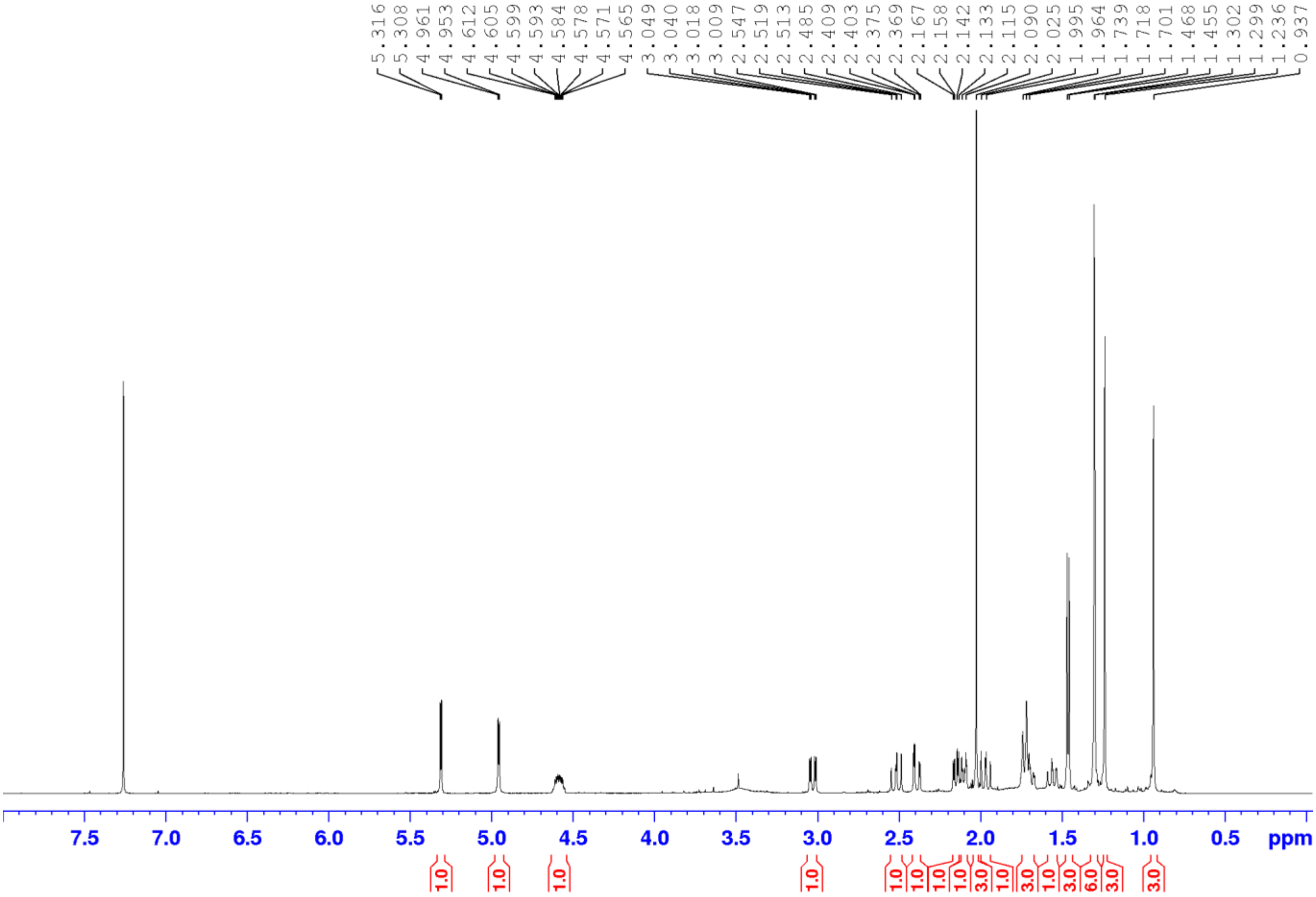

Figure S26: ^{1}H NMR spectrum of **5** in $CDCl_3$ (500 MHz)

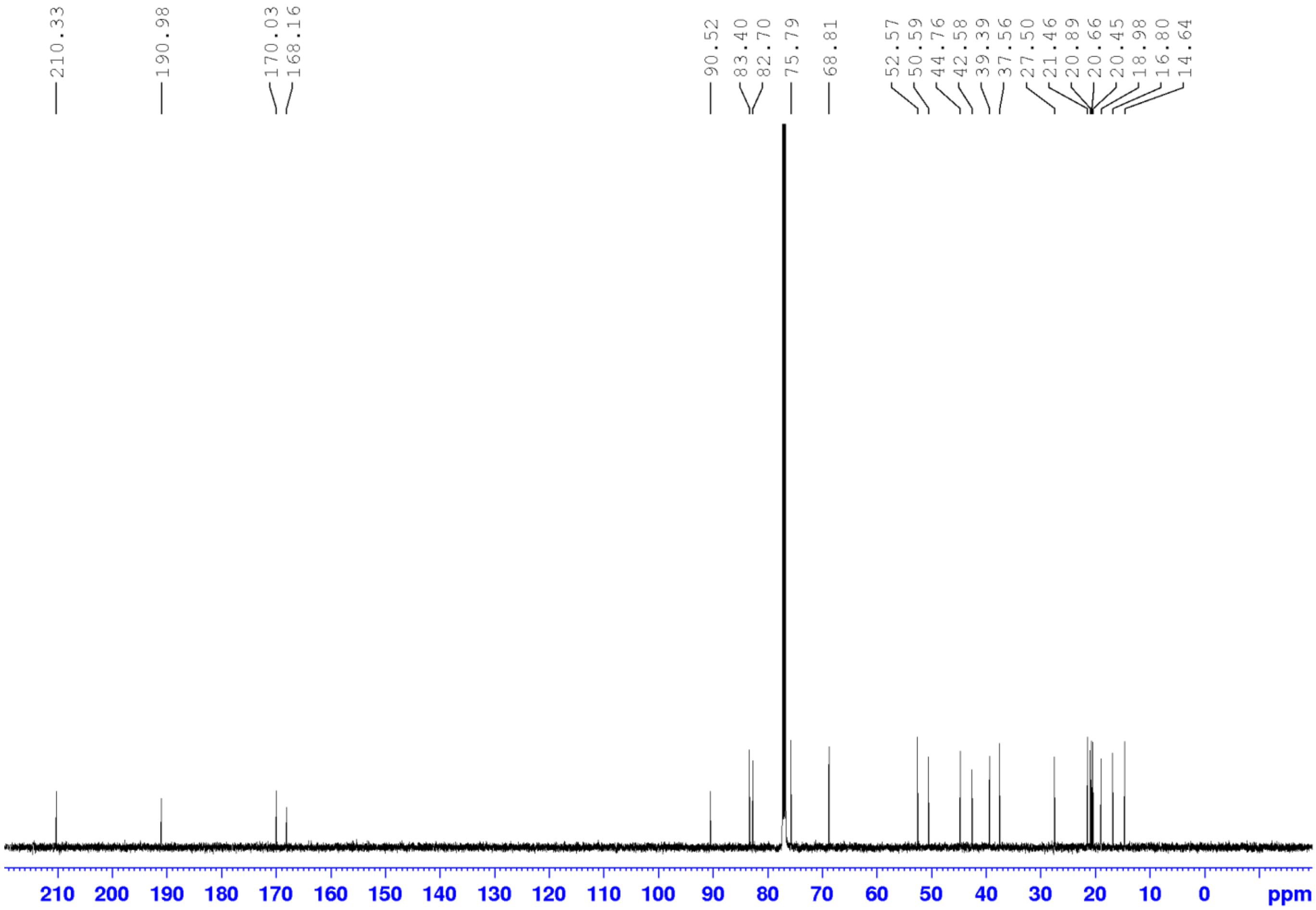

Figure S27: ^{13}C NMR spectrum of **5** in $CDCl_3$ (126 MHz)

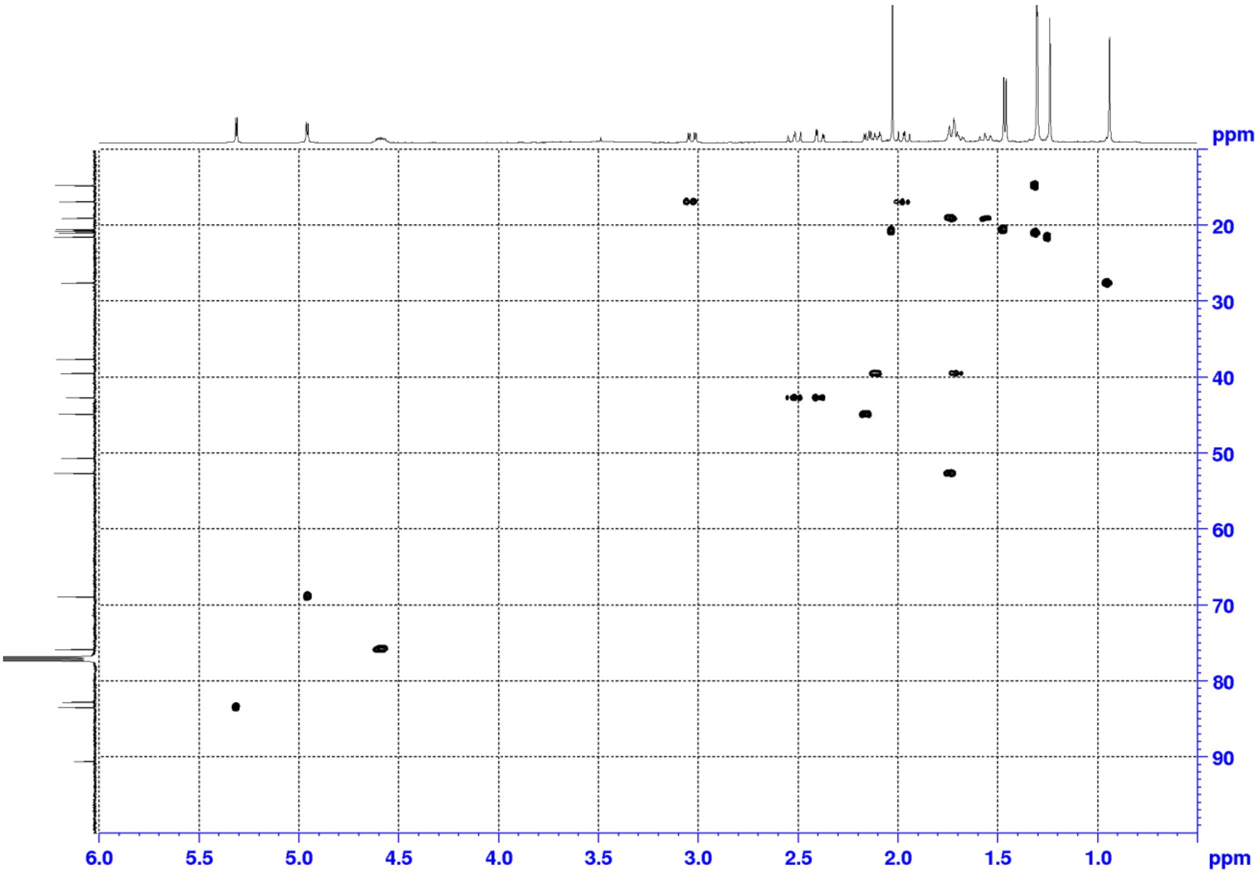

Figure S28: HSQC spectrum of **5** in $CDCl_3$

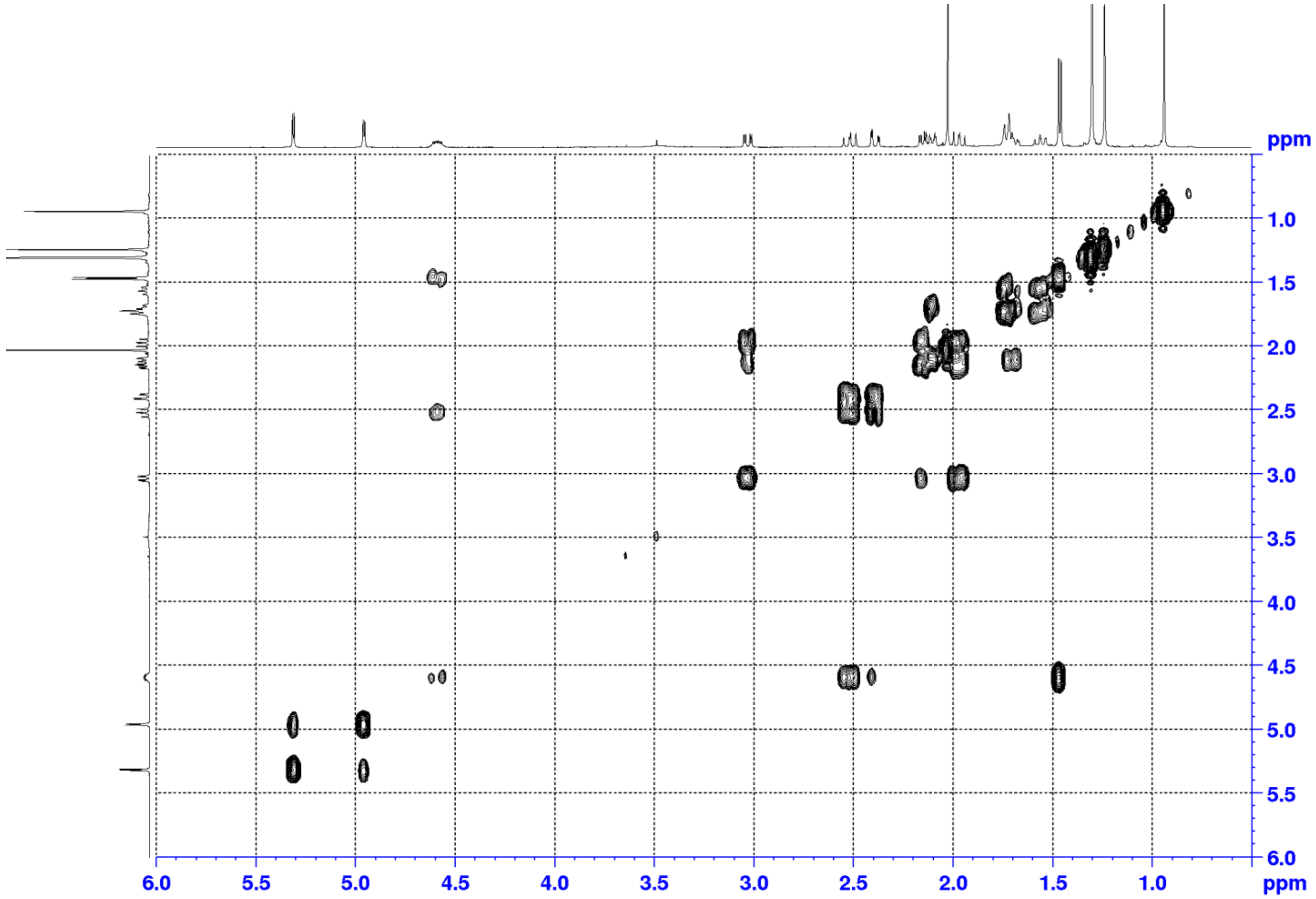

Figure S29: COSY spectrum of **5** in $CDCl_3$

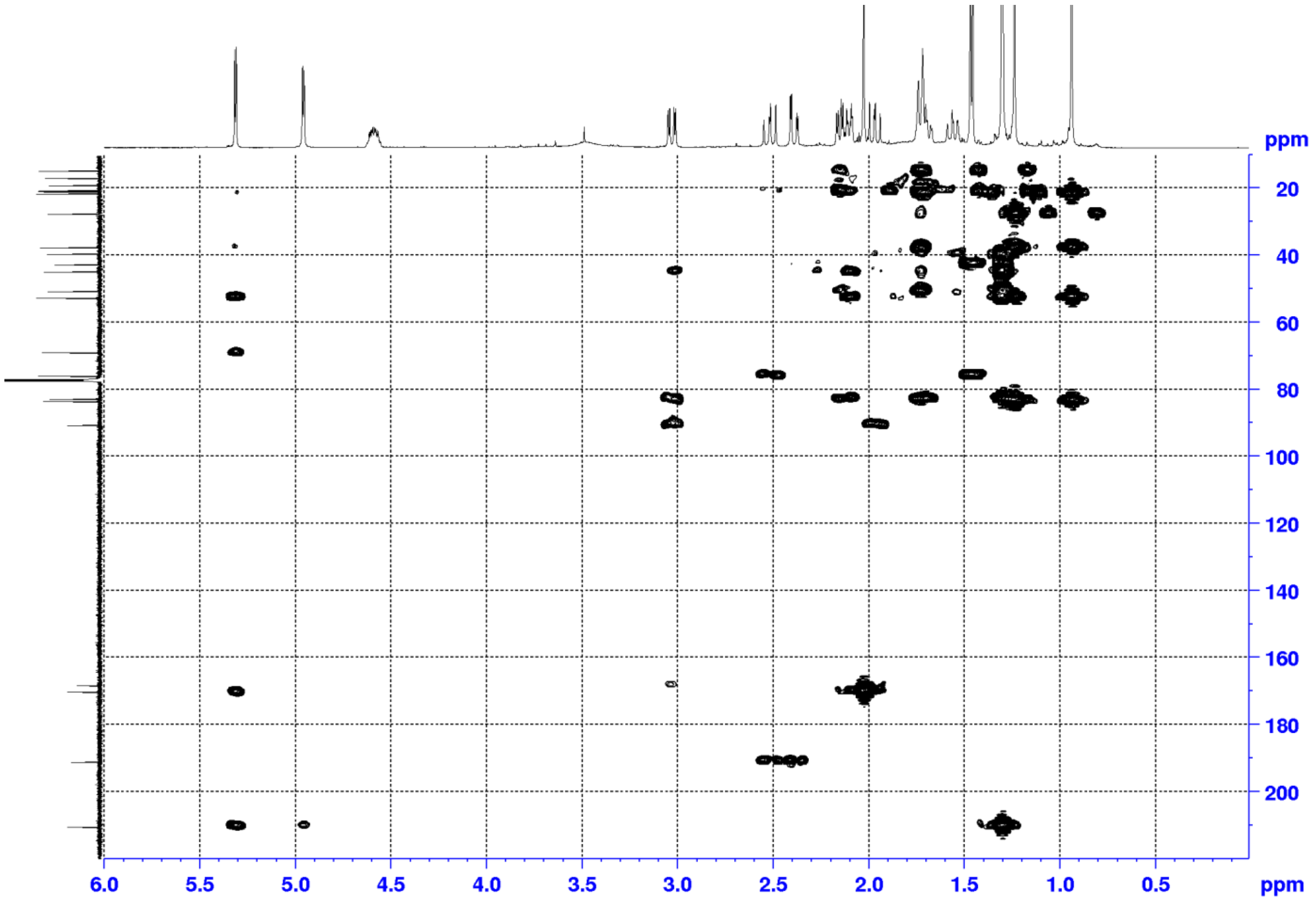

Figure S30: HMBC spectrum of **5** in $CDCl_3$

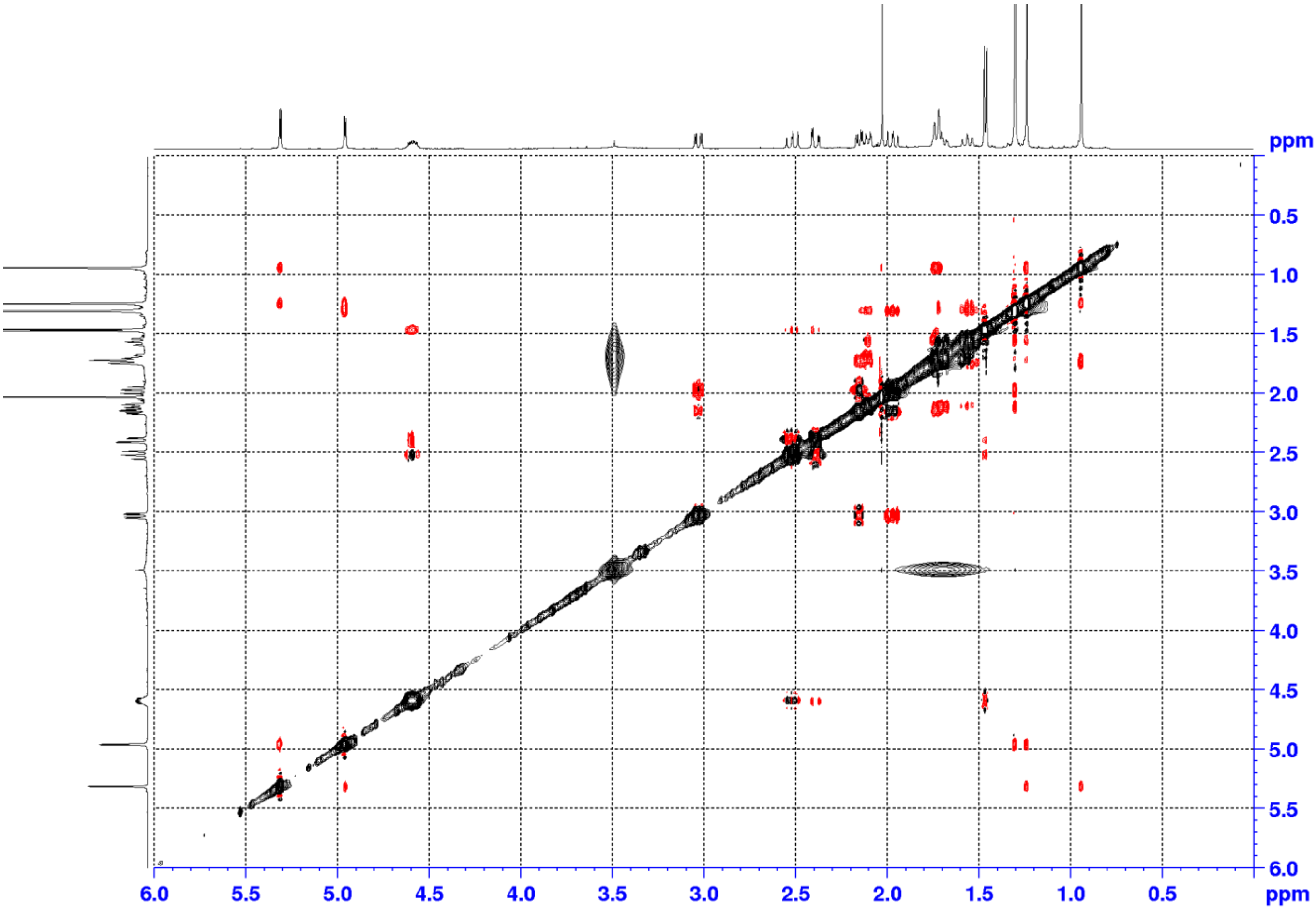

Figure S31: NOESY spectrum of **5** in $CDCl_3$

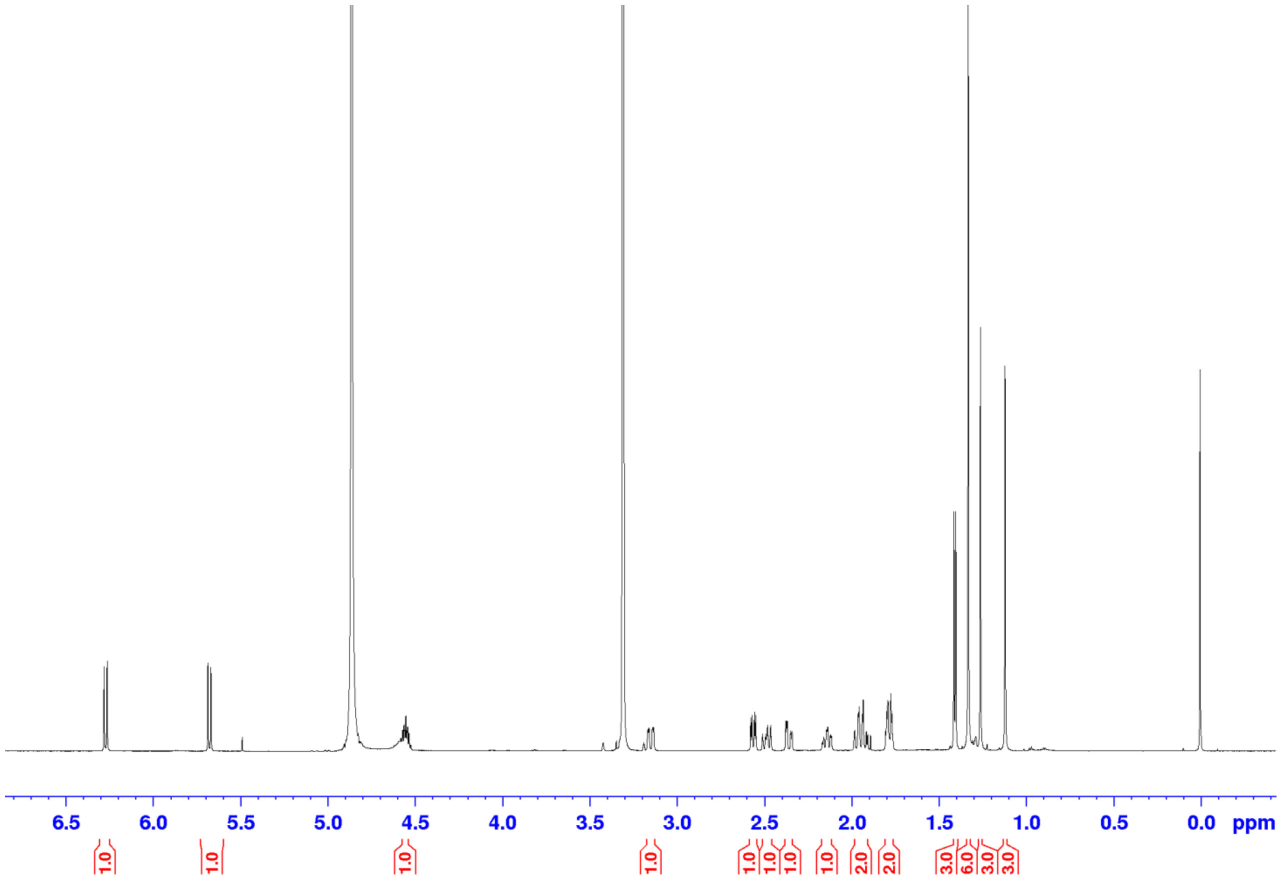

Figure S32: ^{1}H NMR spectrum of **6** in $CDCl_3$ (600 MHz)

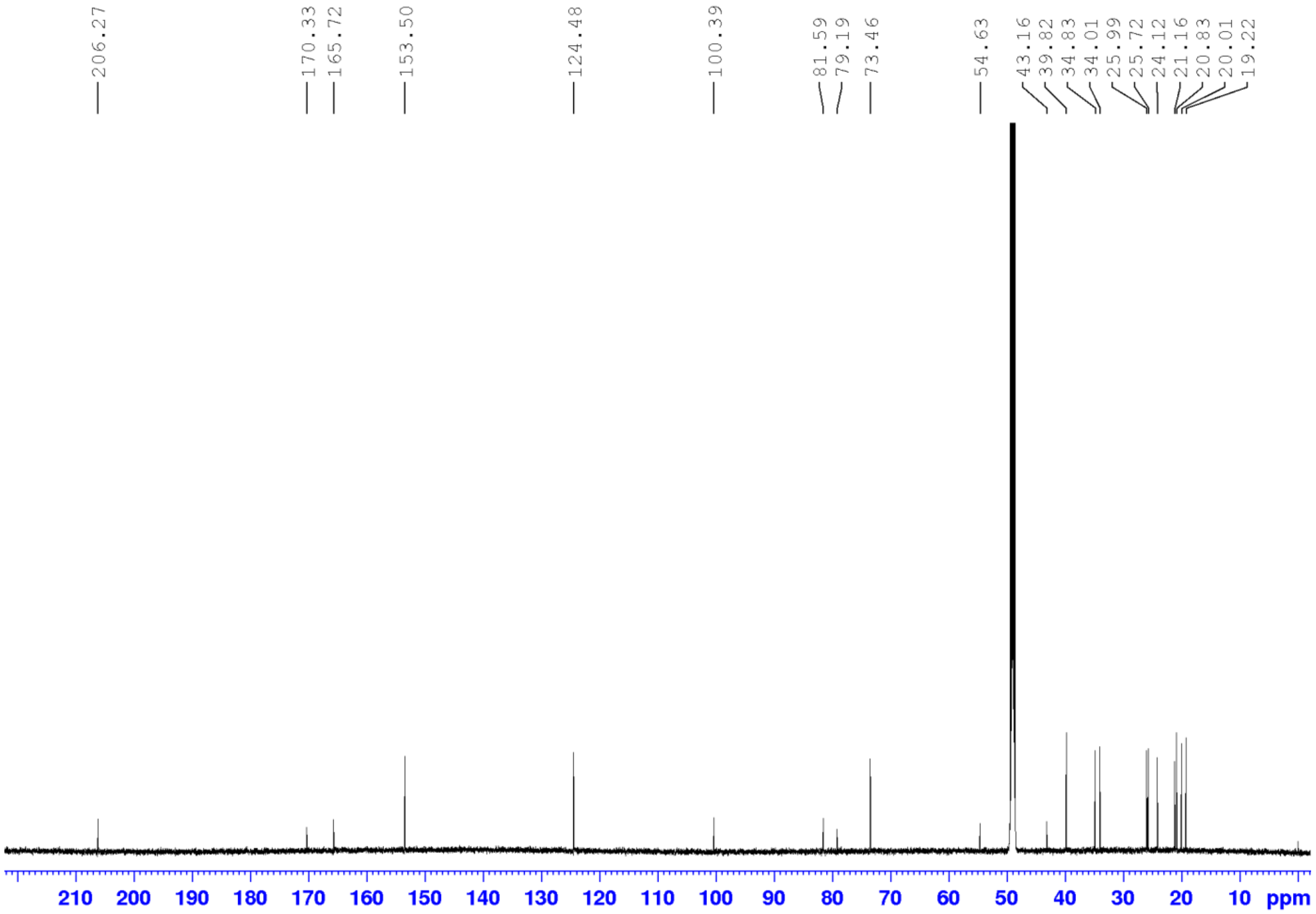

Figure S33: ^{13}C NMR spectrum of **6** in $CDCl_3$ (151 MHz)

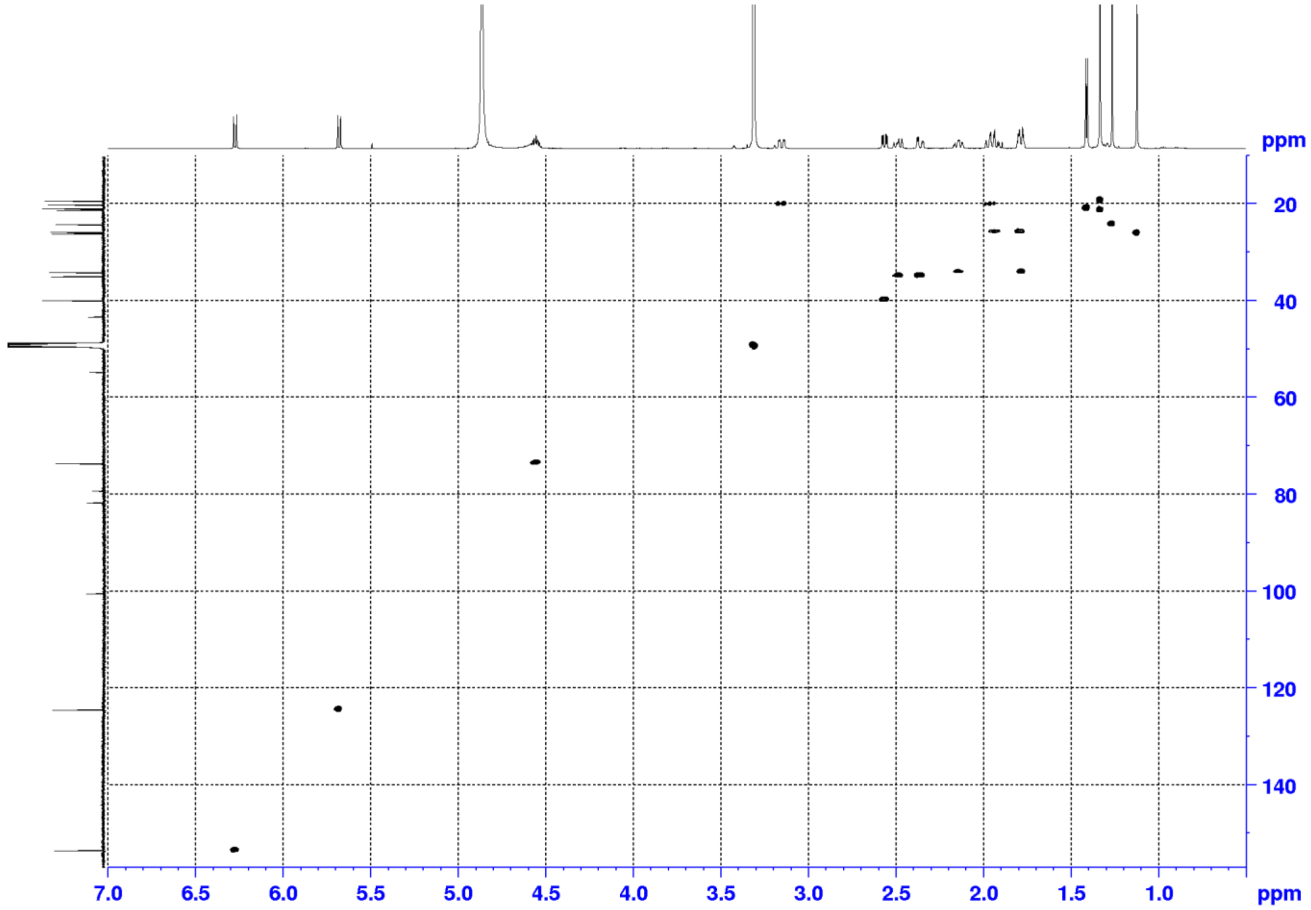

Figure S34: HSQC spectrum of **6** in $CDCl_3$

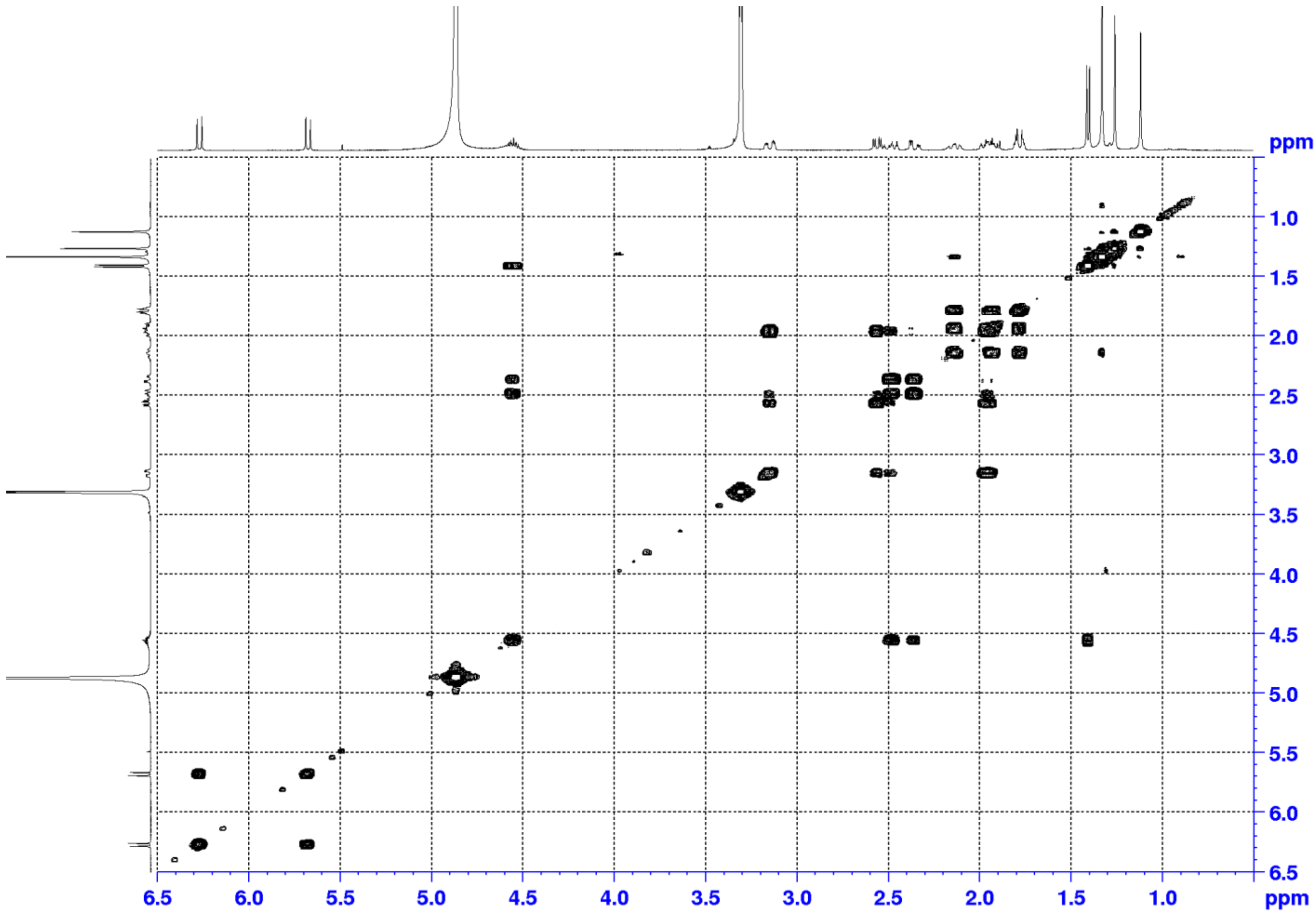

Figure S35: COSY spectrum of **6** in $CDCl_3$

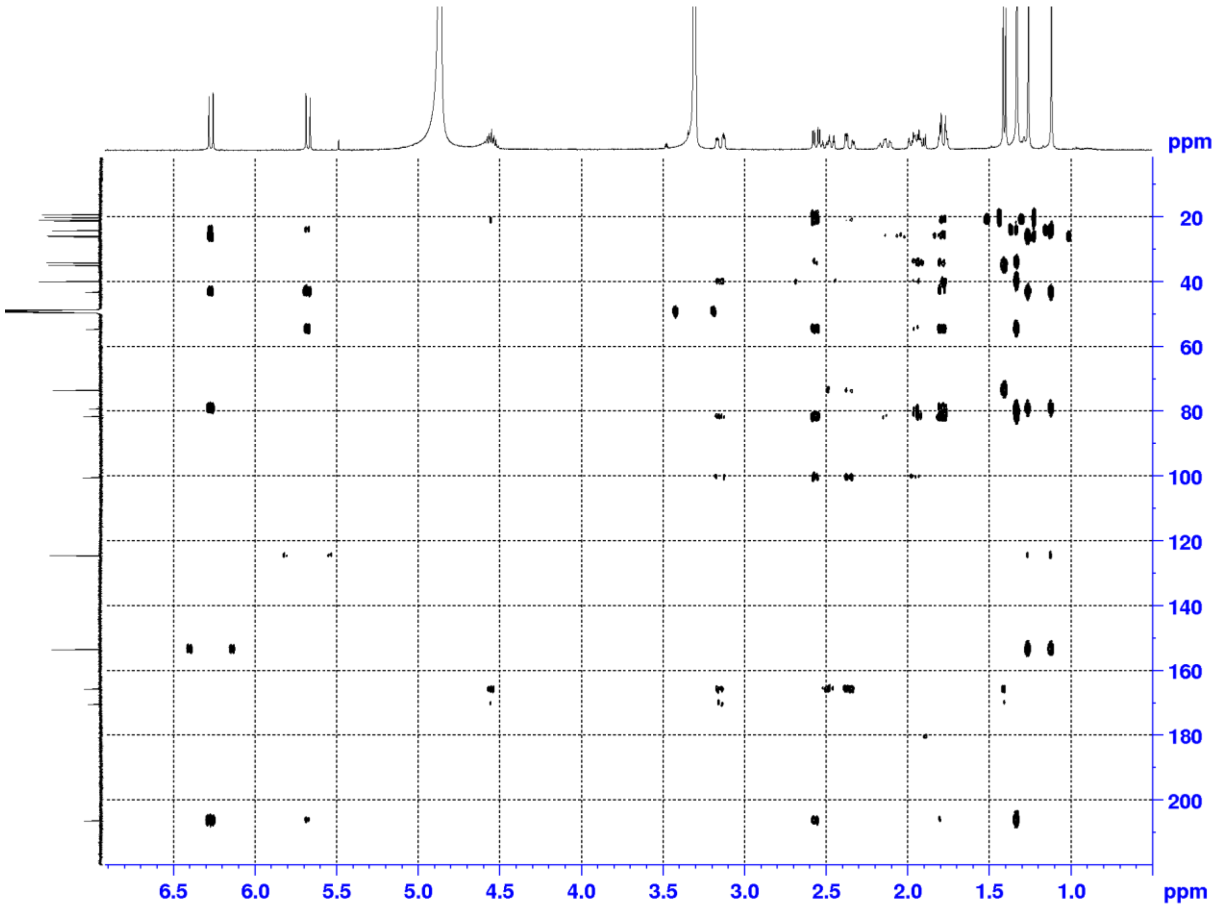

Figure S36: HMBC spectrum of **6** in $CDCl_3$

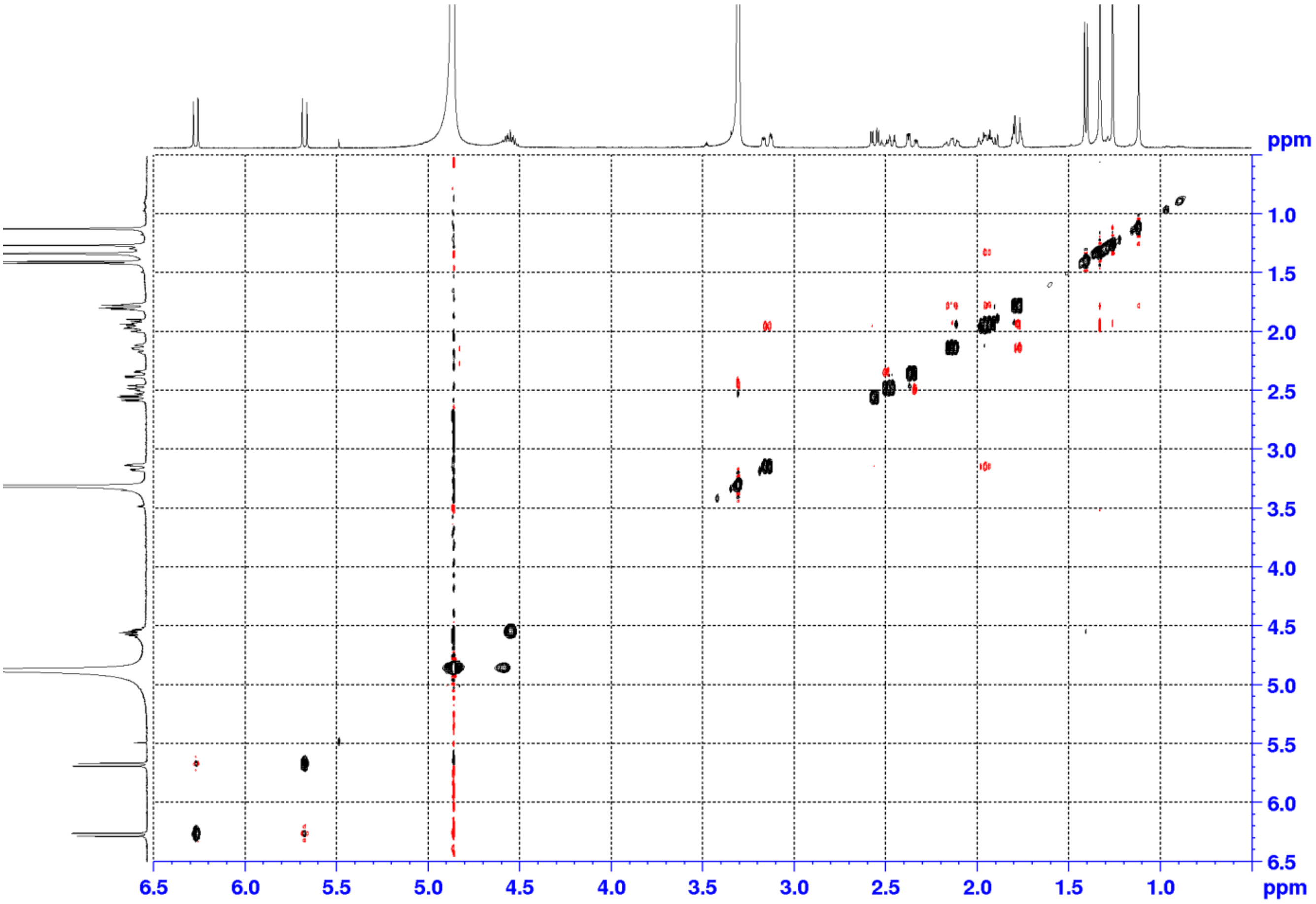

Figure S37: NOESY spectrum of **6** in $CDCl_3$

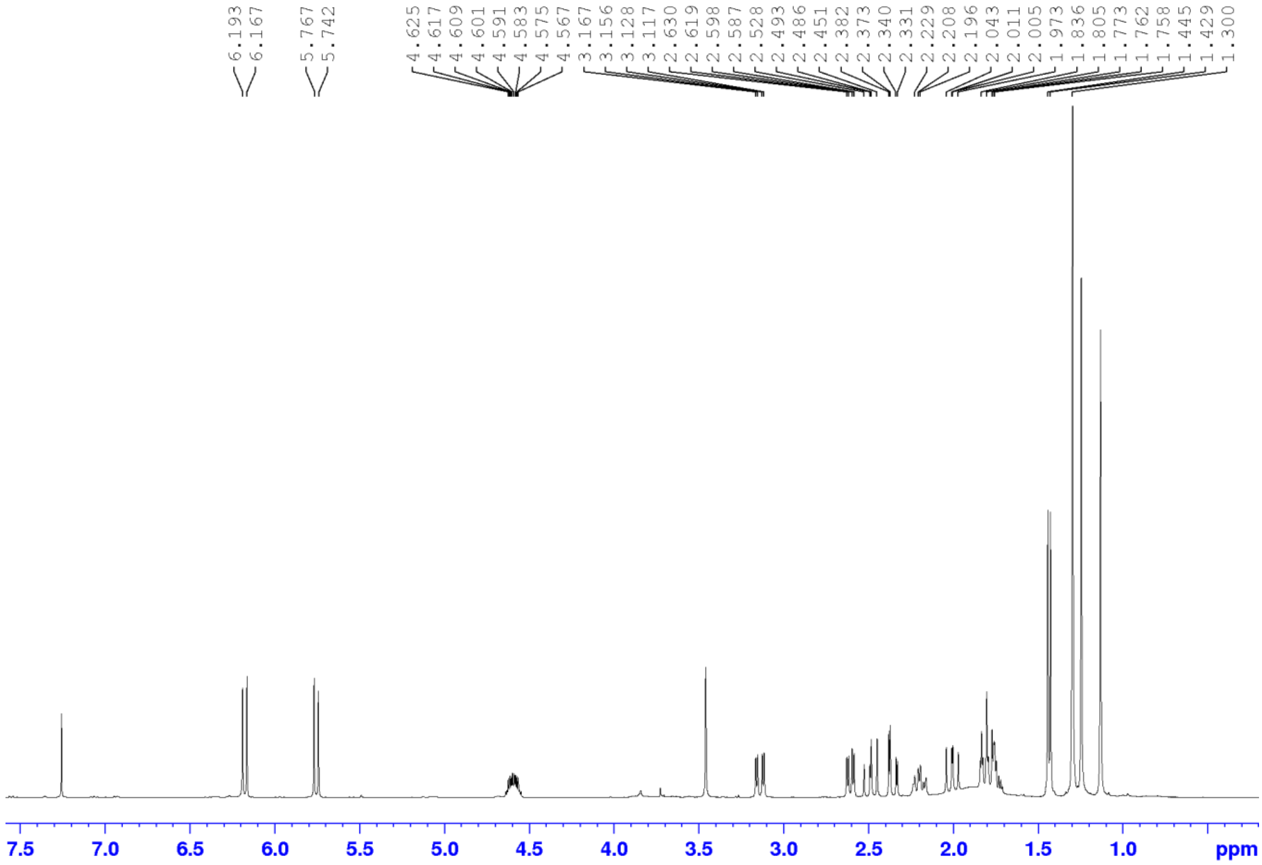

Figure S38: ^{1}H NMR spectrum of **7** in $CDCl_3$ (400 MHz)

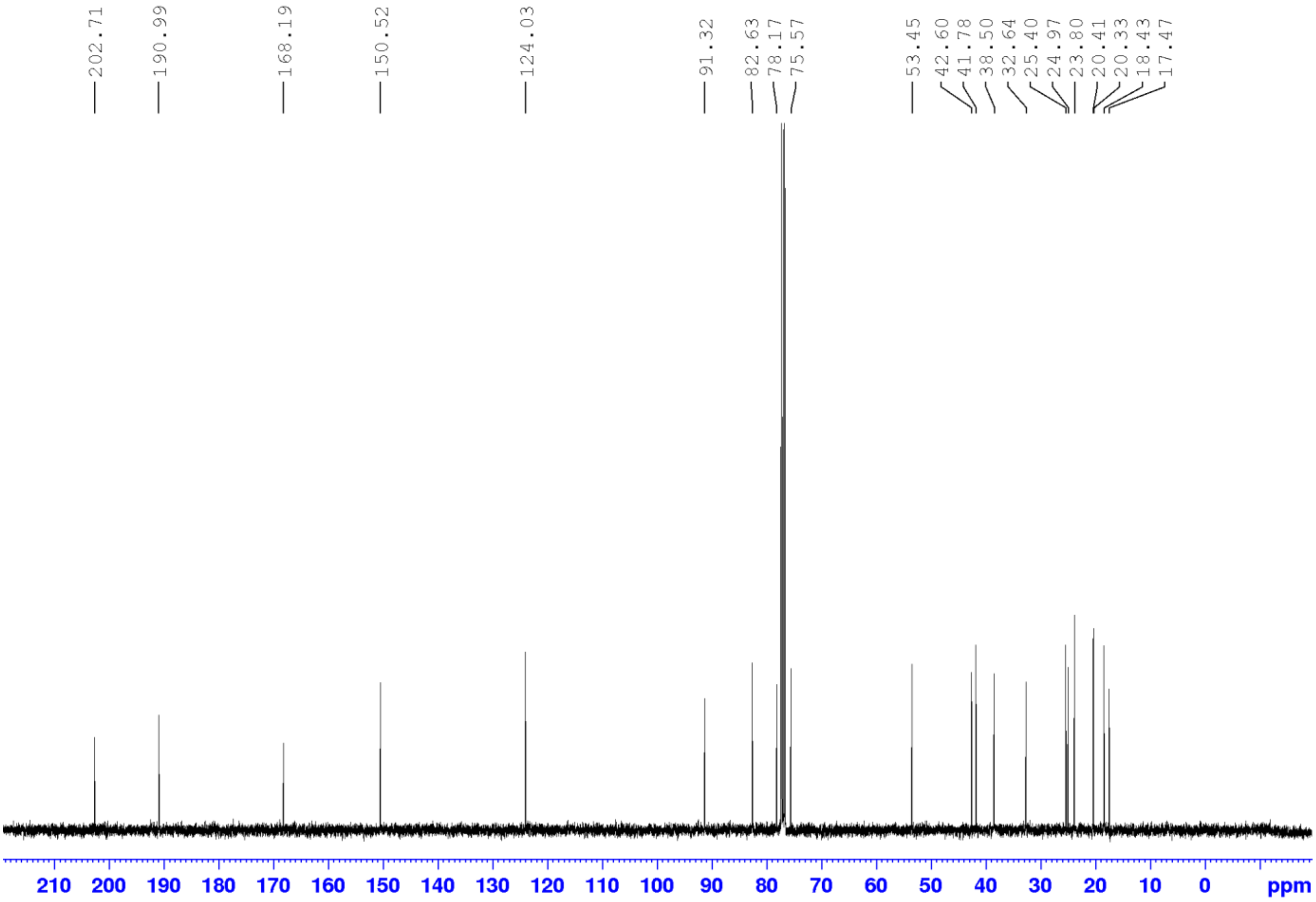

Figure S39: ^{13}C NMR spectrum of **7** in in $CDCl_3$ (101 MHz)

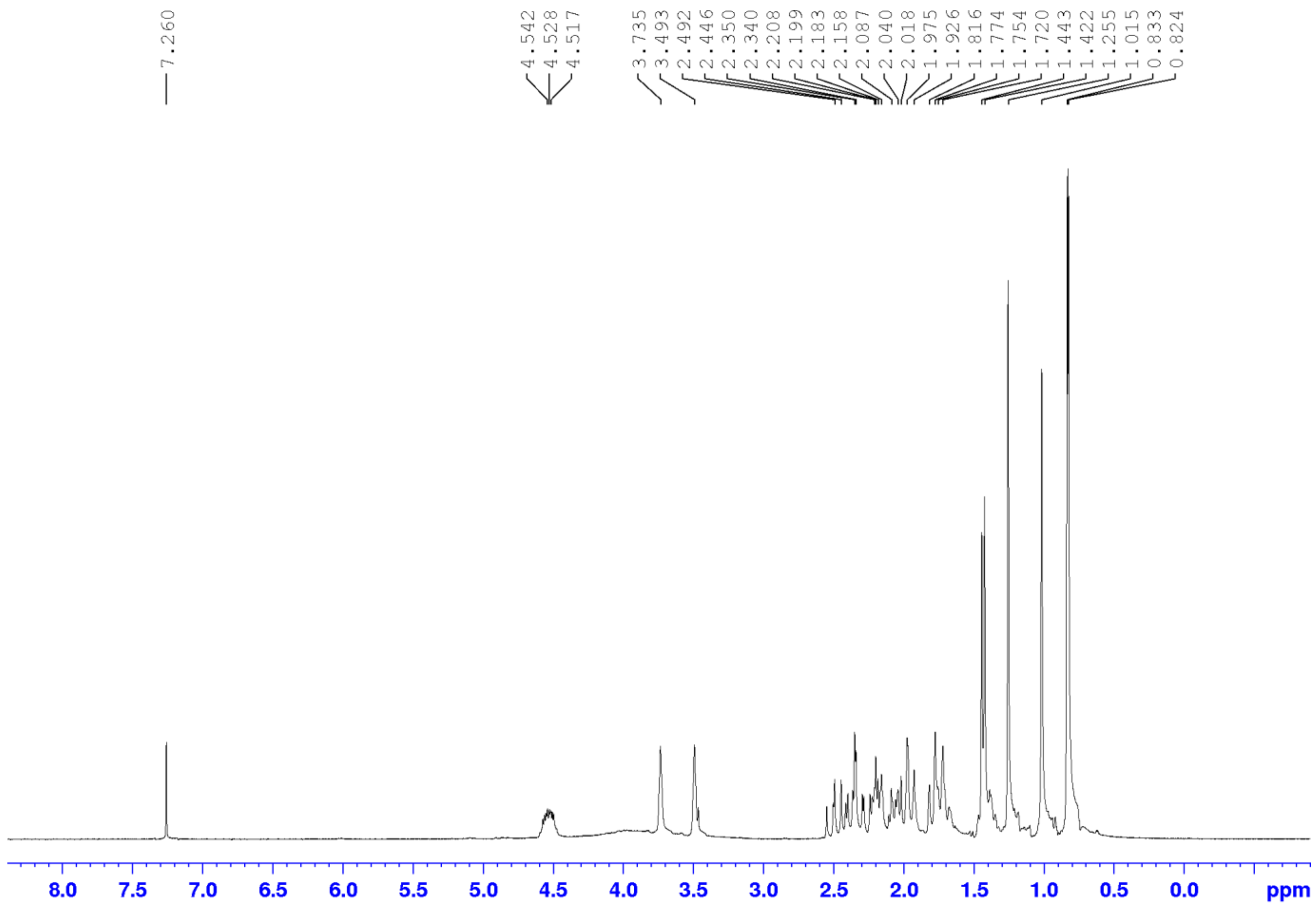

Figure S40: ^{1}H NMR spectrum of **8** in $CDCl_3$ (300 MHz)

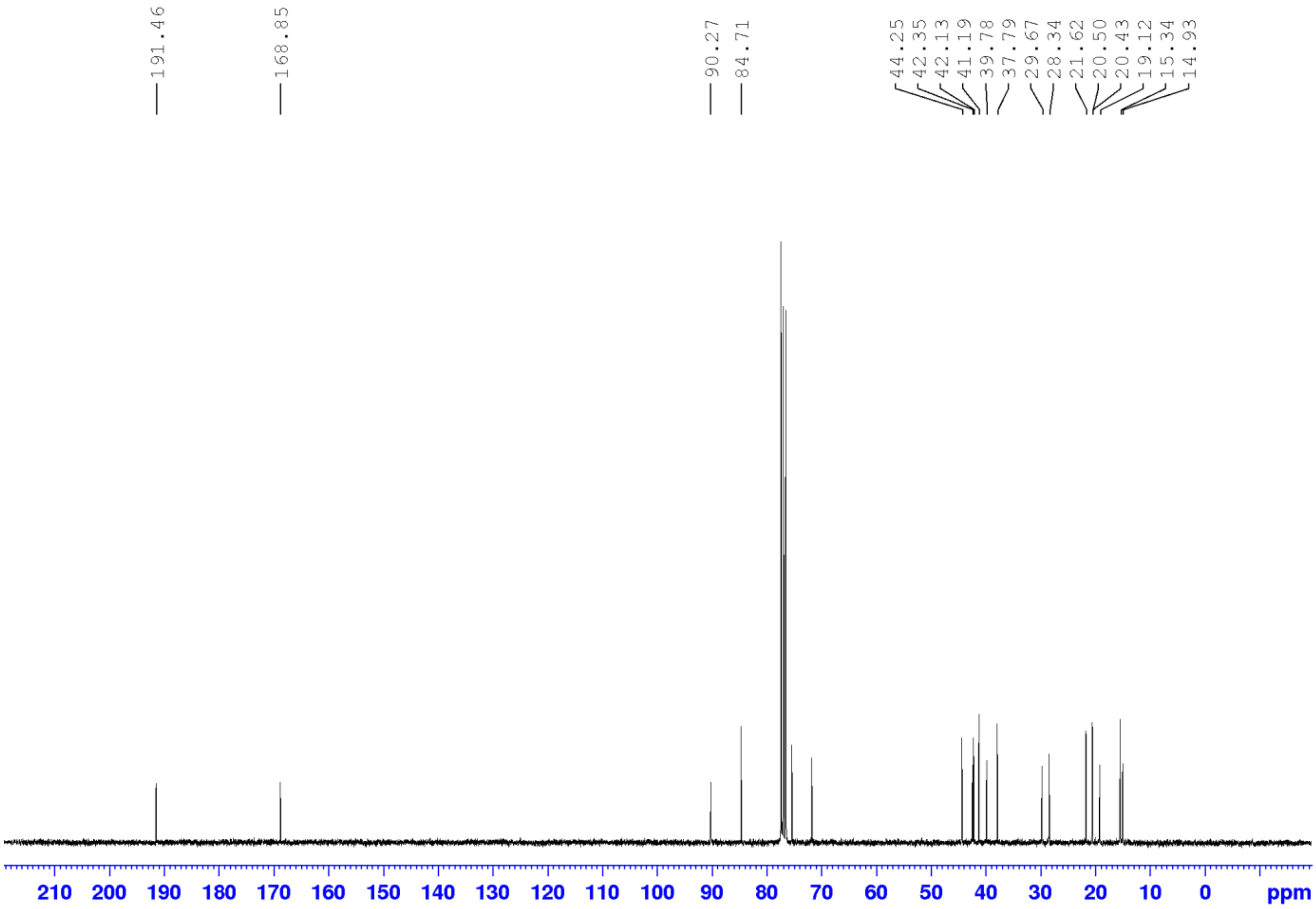

Figure S41: ^{13}C NMR spectrum of **8** in $CDCl_3$ (75.5 MHz)

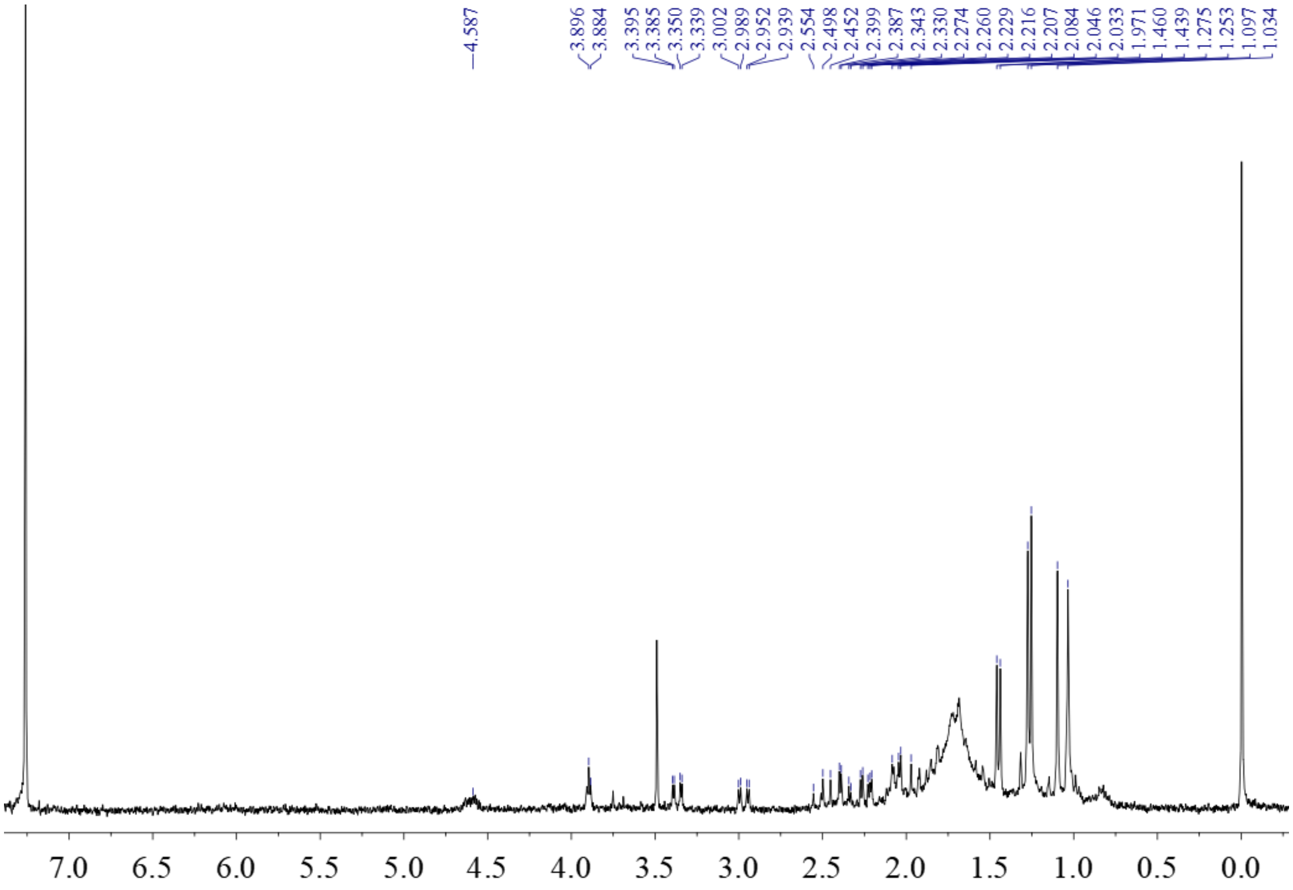

Figure S42: ^{1}H NMR spectrum of **9** in $CDCl_3$ (300 MHz)

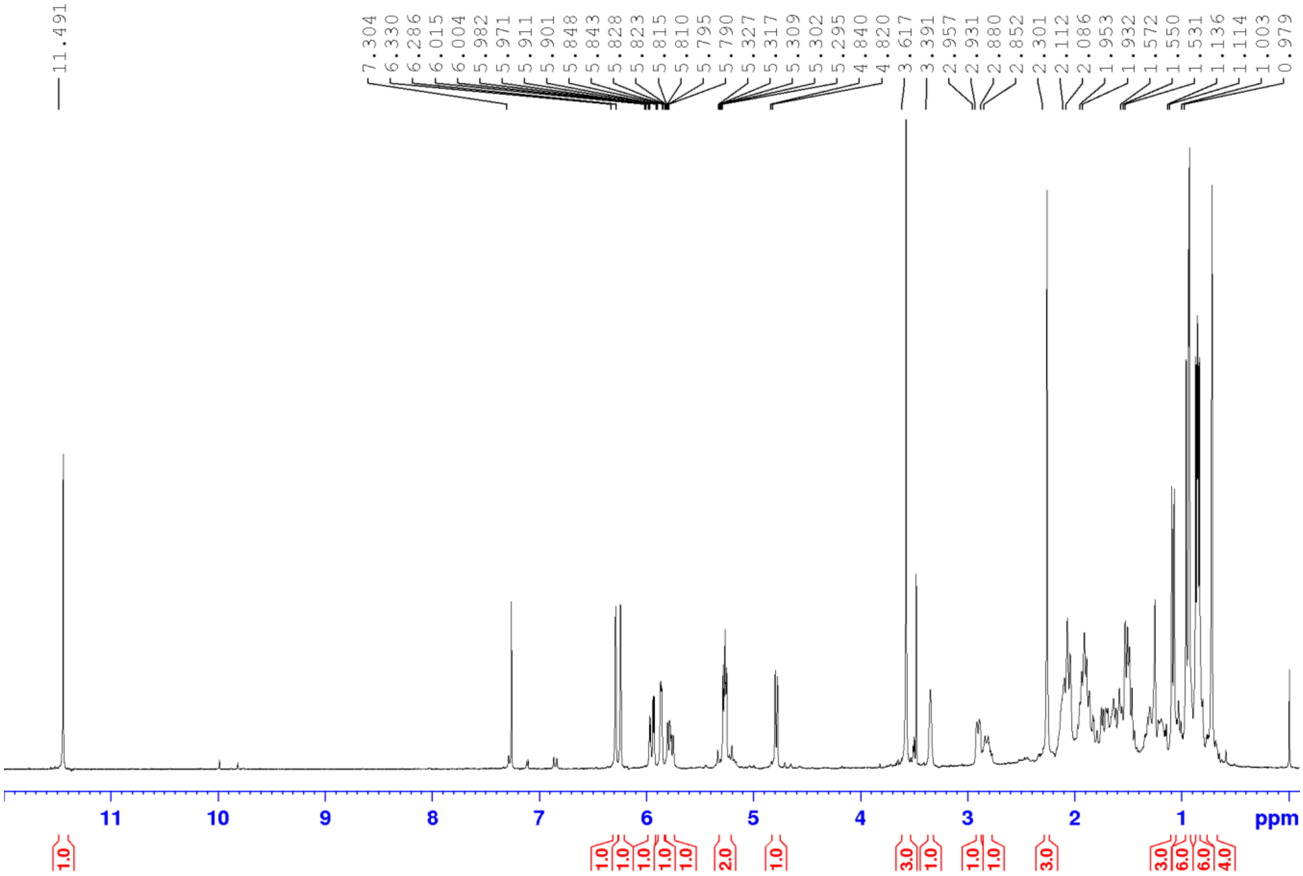

Figure S43: ^{1}H NMR spectrum of **10** in $CDCl_3$ (500 MHz)

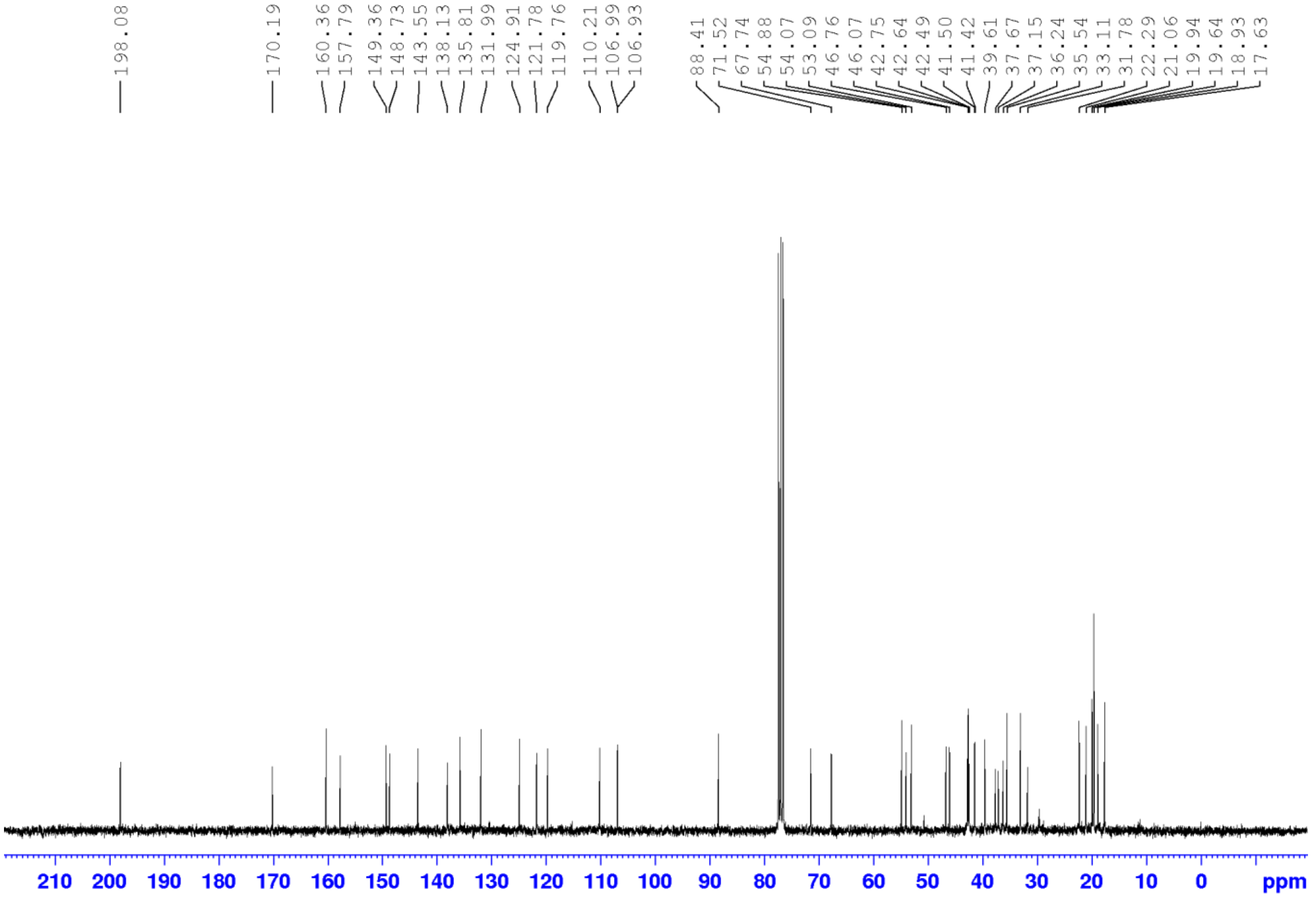

Figure S44: ^{13}C NMR spectrum of **10** in $CDCl_3$ (75.5 MHz)

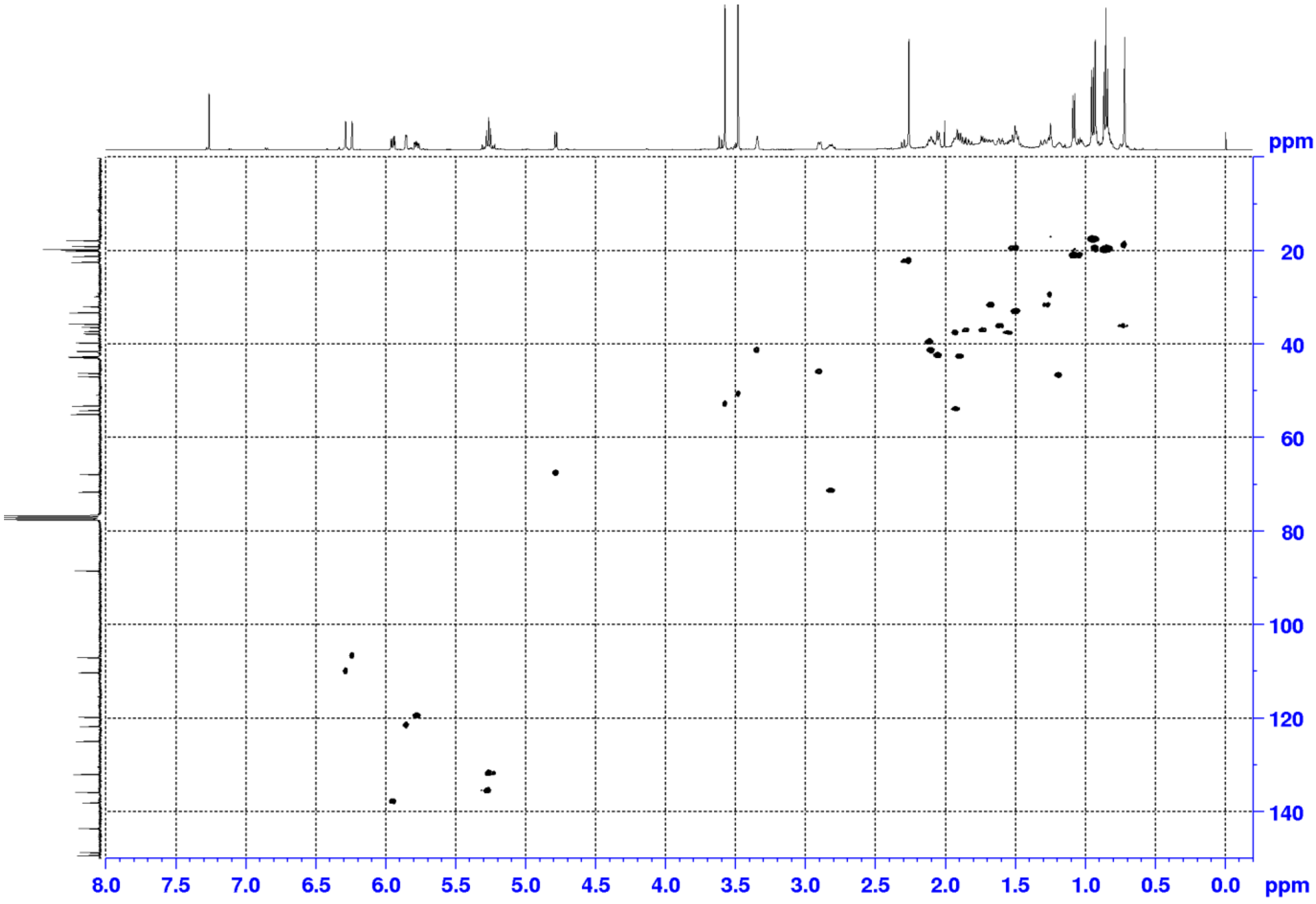

Figure S45: HSQC spectrum of **10** in $CDCl_3$

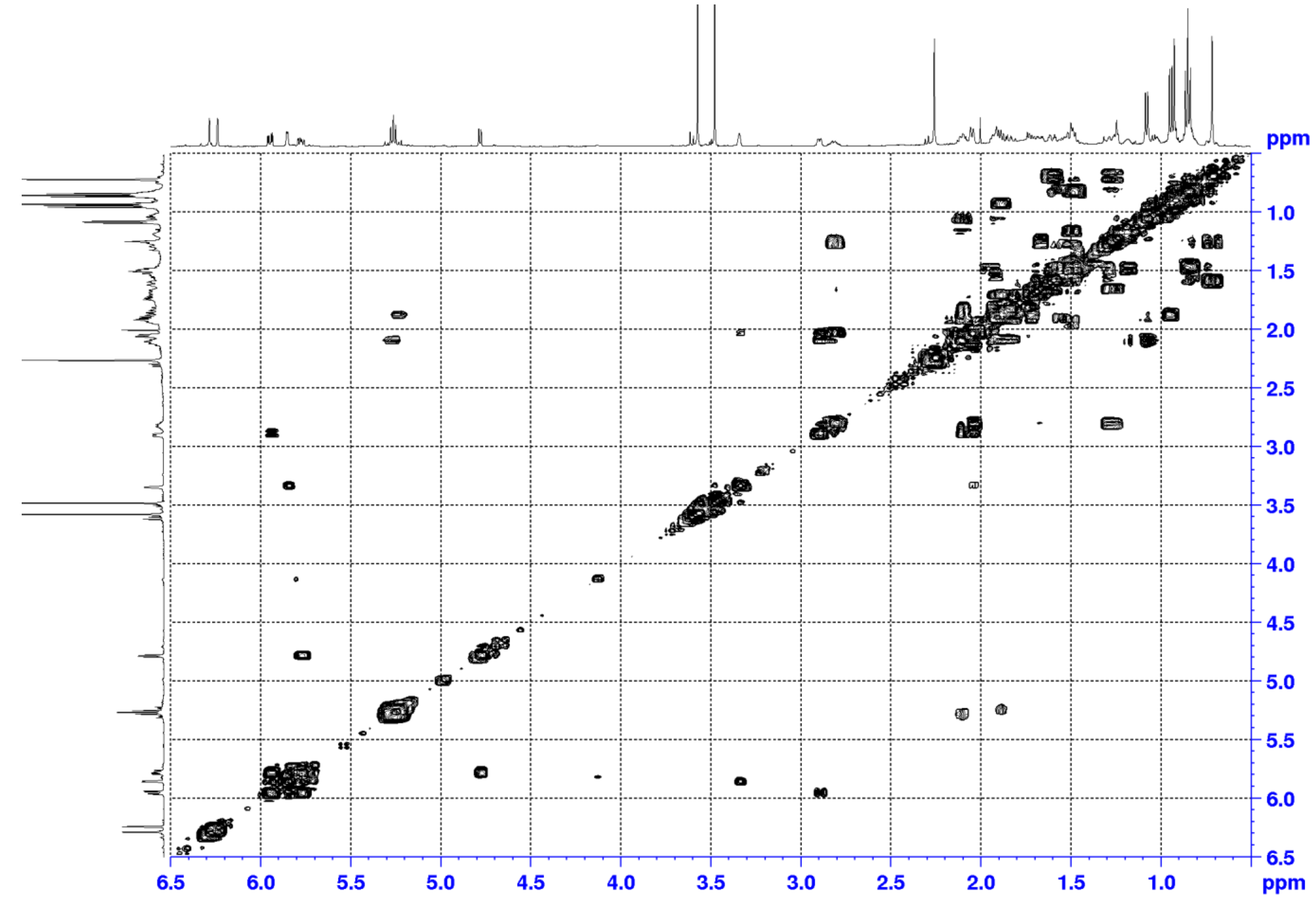

Figure S46: COSY spectrum of **10** in $CDCl_3$

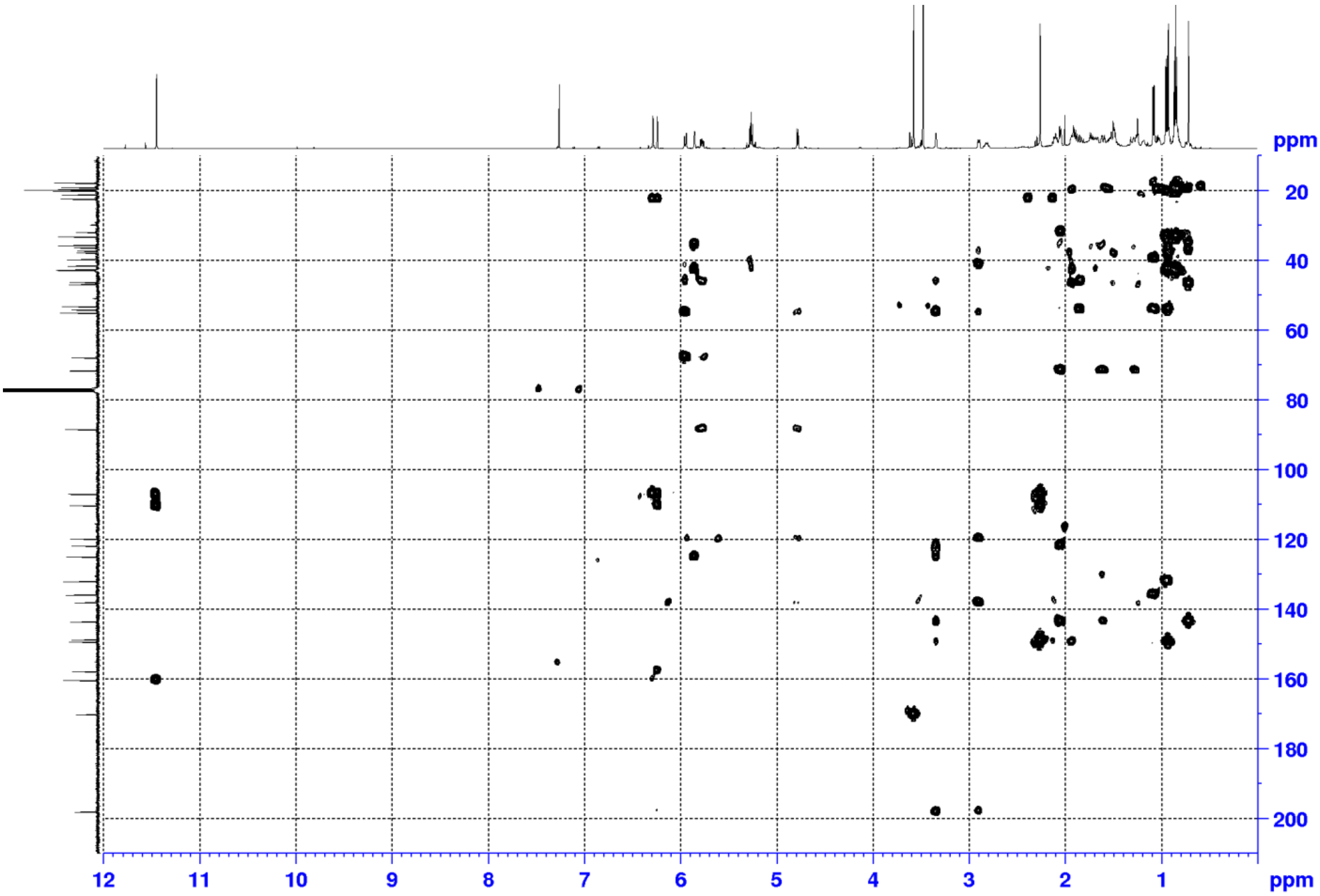

Figure S47: HMBC spectrum of **10** in in $CDCl_3$

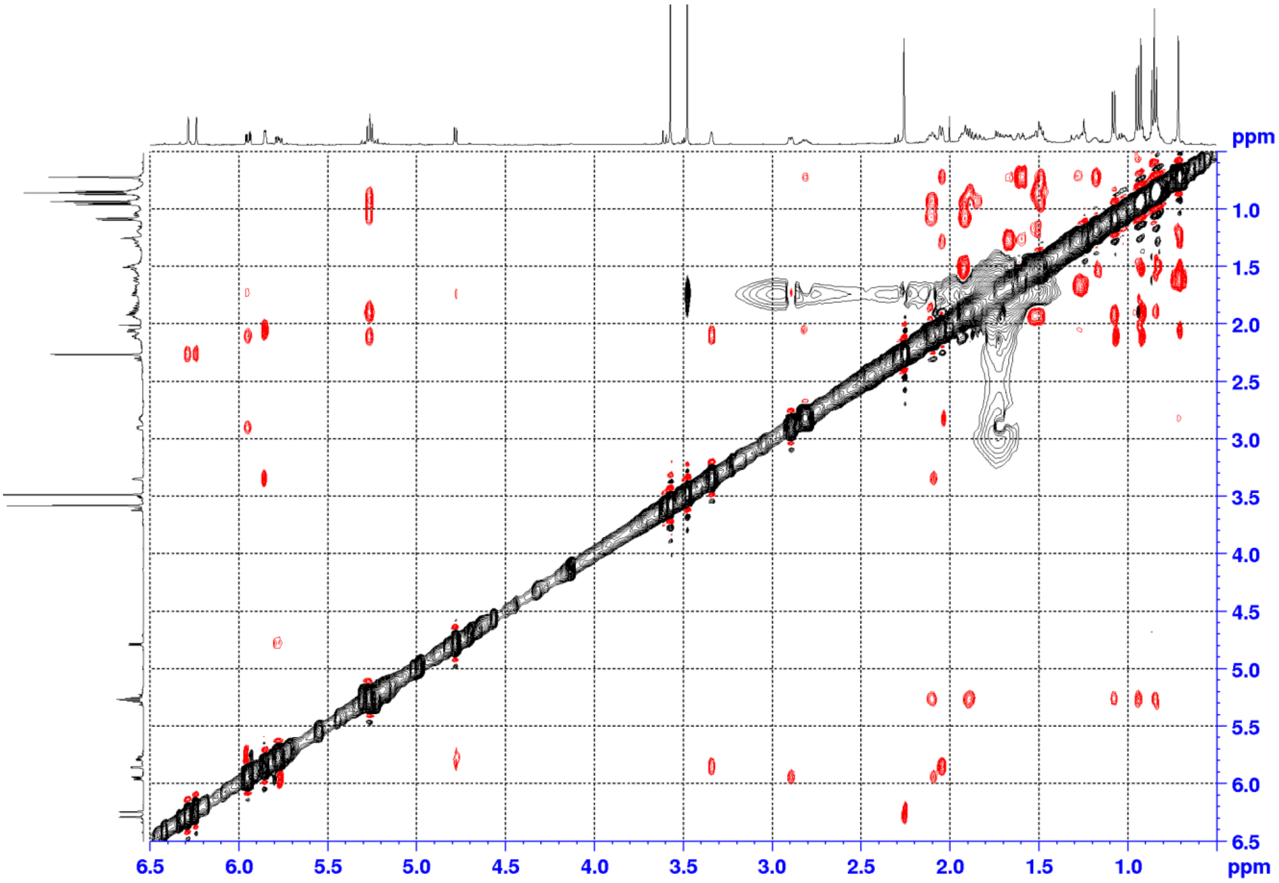

Figure S48: NOESY spectrum of **11** in in $CDCl_3$

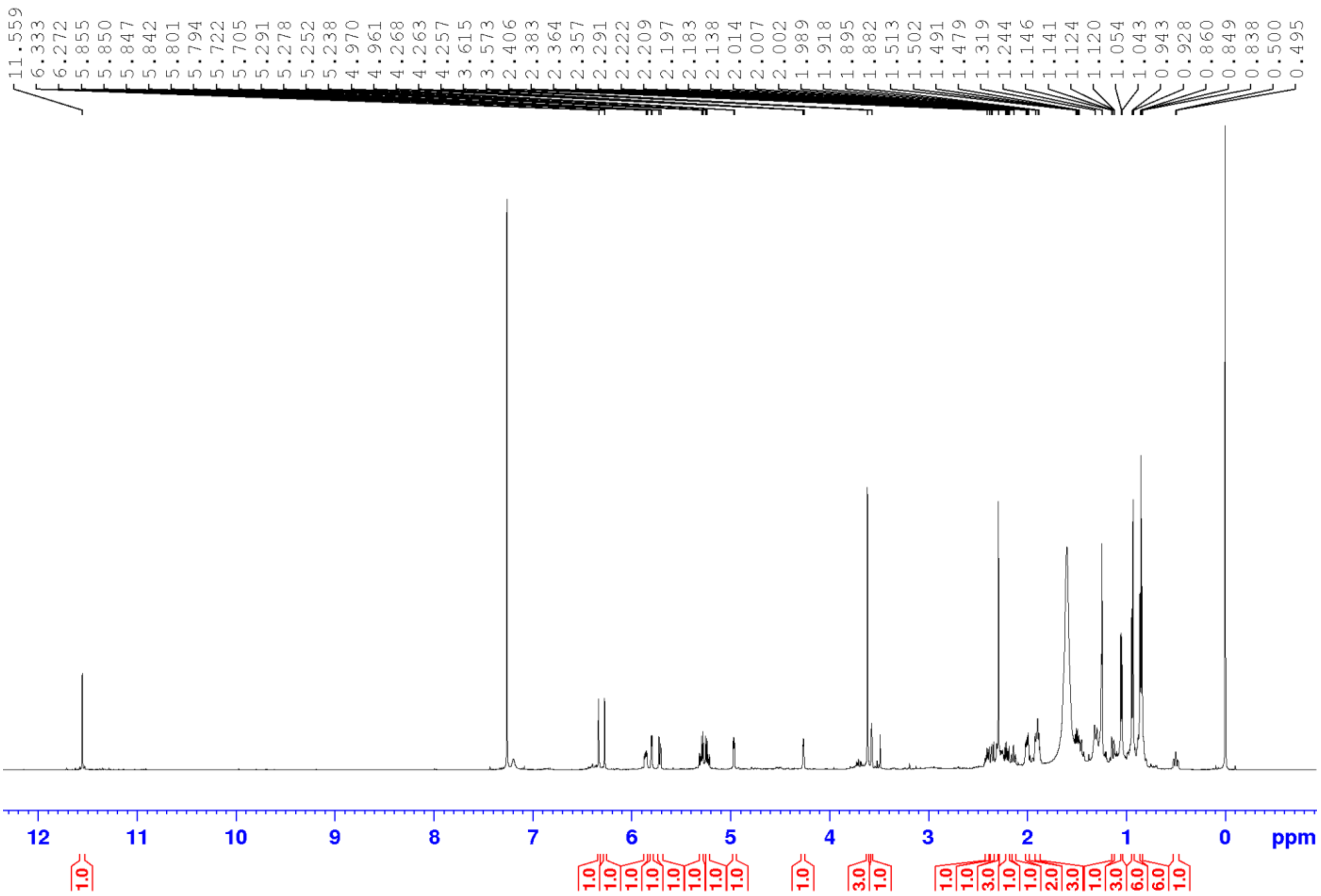

Figure S49: ^{1}H NMR spectrum of **10a** in $CDCl_3$ (600 MHz)

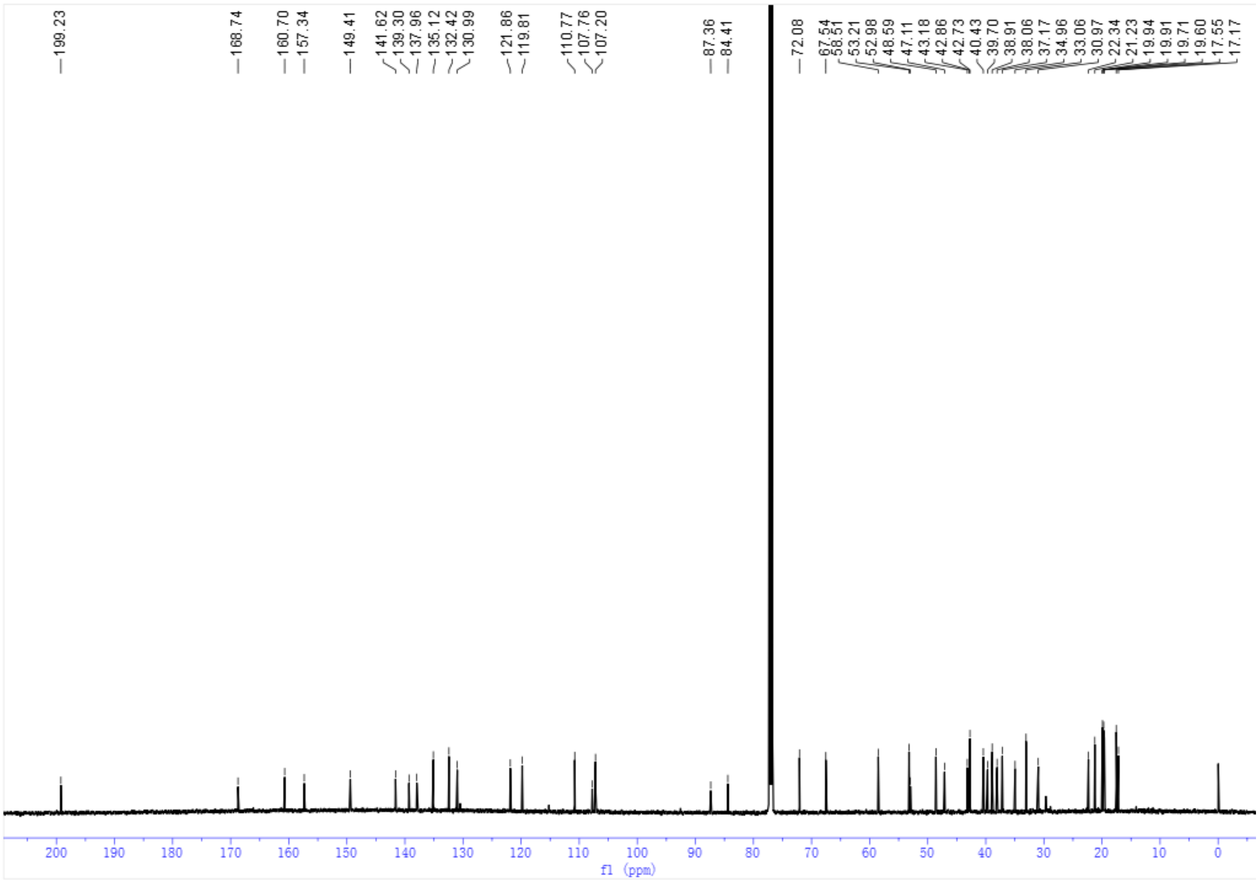

Figure S50: ^{13}C NMR spectrum of **10a** in $CDCl_3$ (151 MHz)

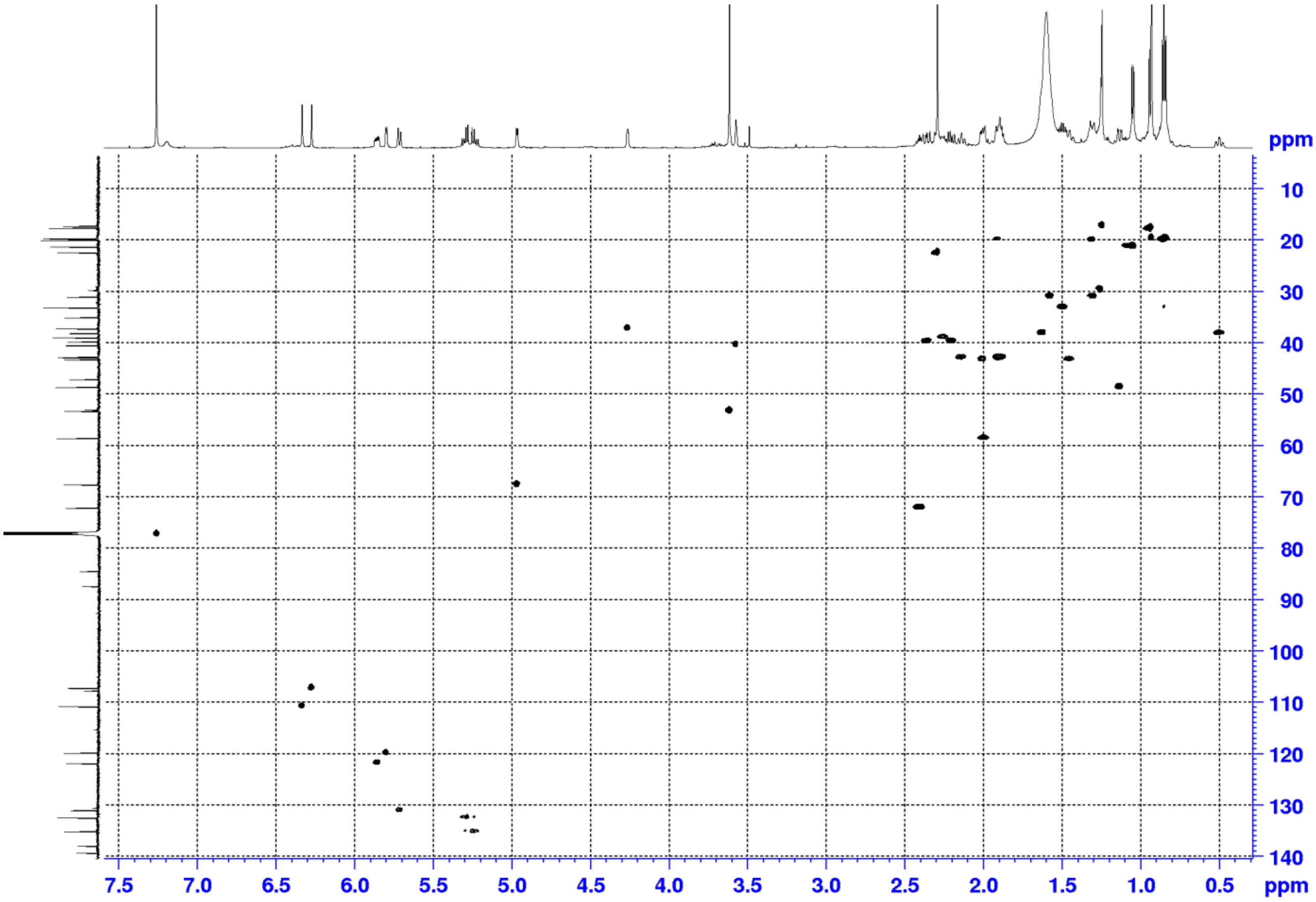

Figure S51: HSQC spectrum of **10a** in $CDCl_3$

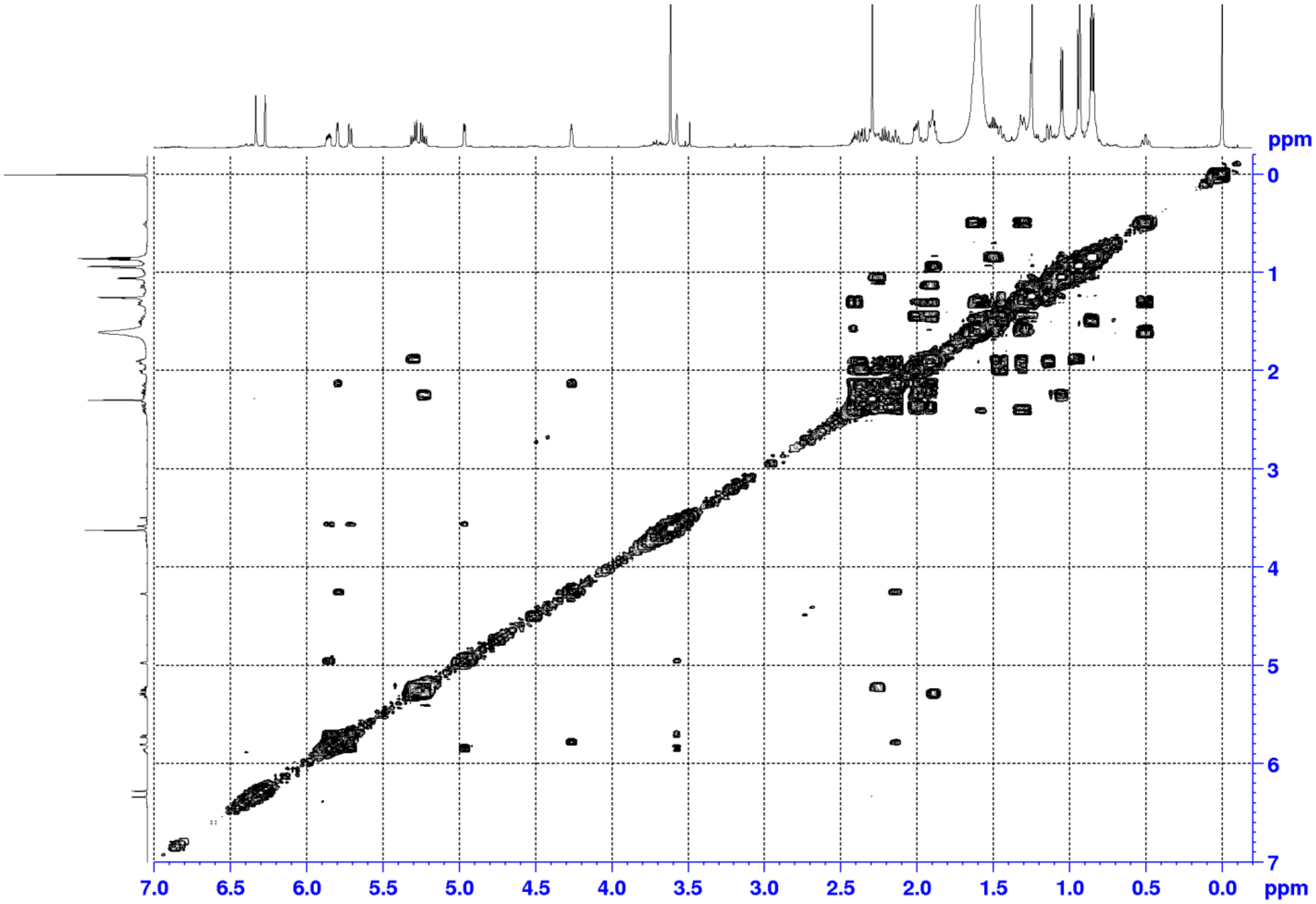

Figure S52: COSY spectrum of **10a** in $CDCl_3$

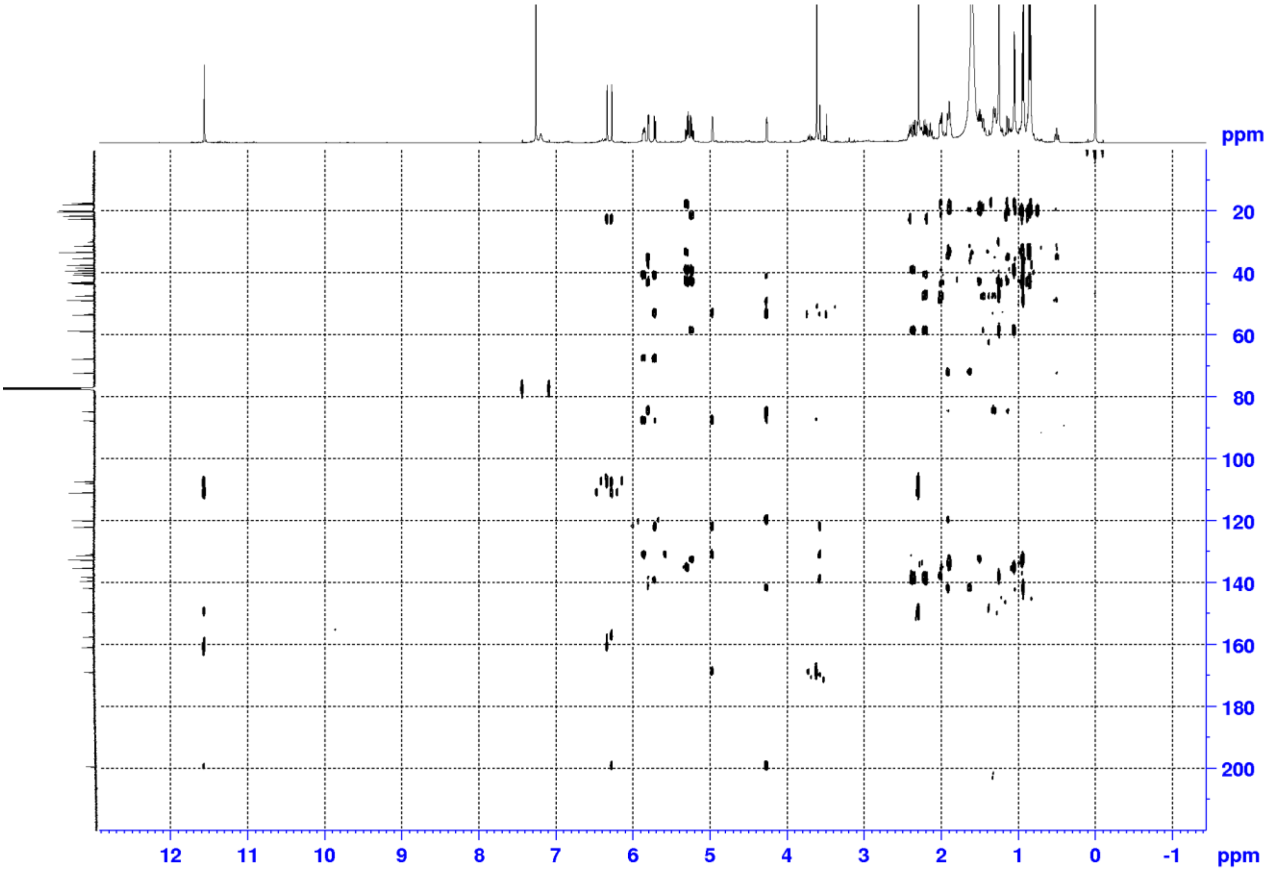

Figure S53: HMBC spectrum of **10a** in in $CDCl_3$

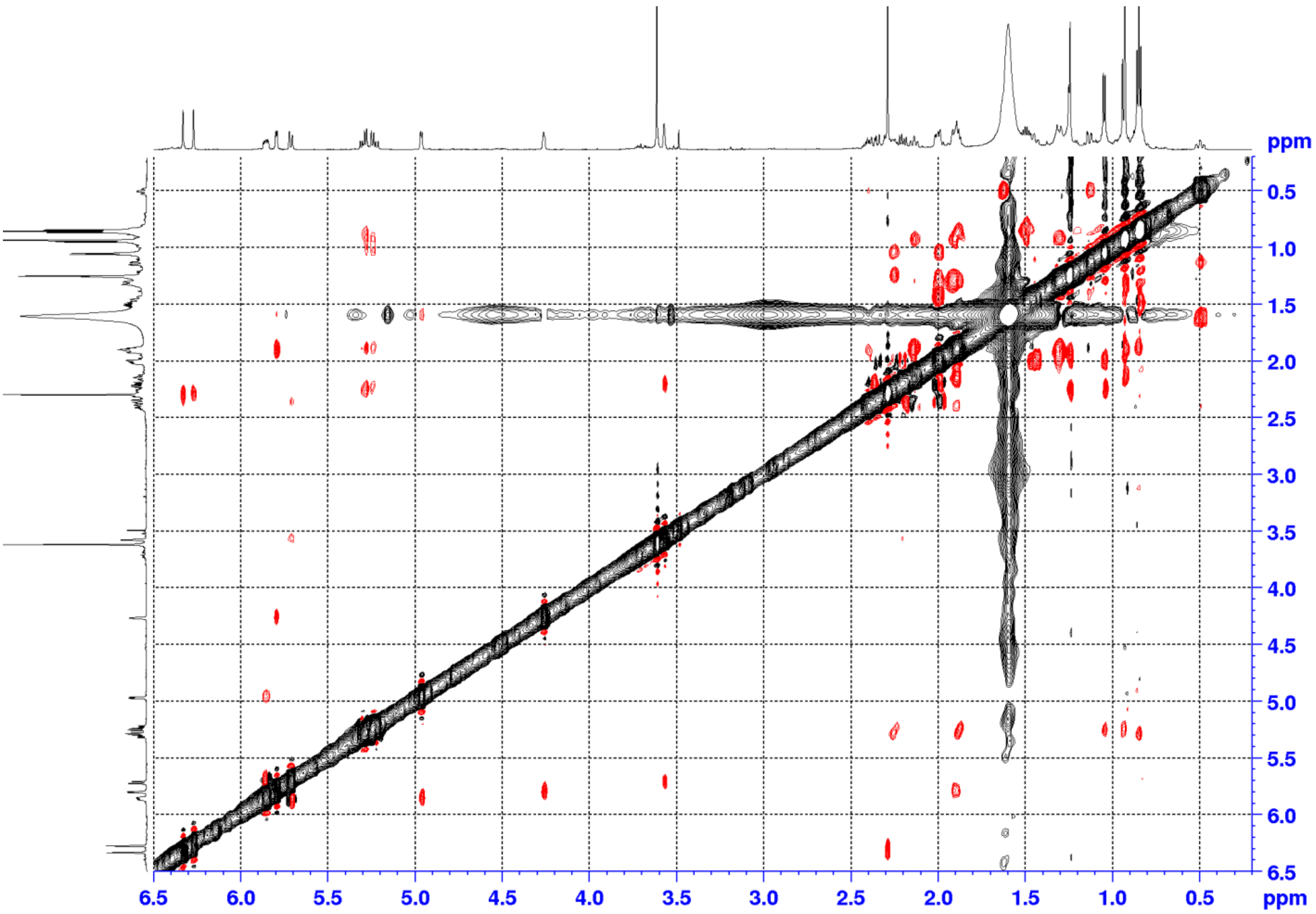

Figure S54: NOESY spectrum of **10a** in in $CDCl_3$

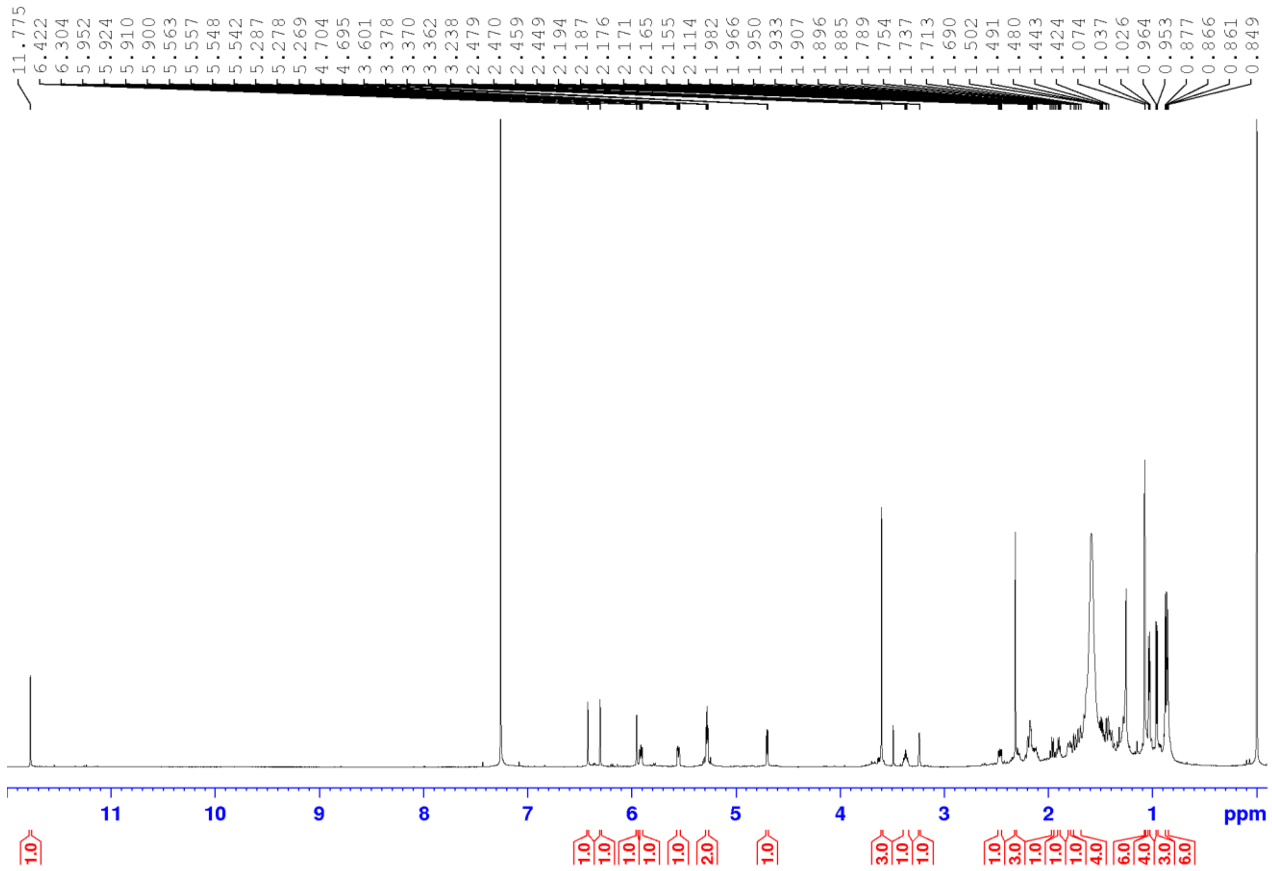

Figure S55: ^1H NMR spectrum of **10b** in $CDCl_3$ (600 MHz)

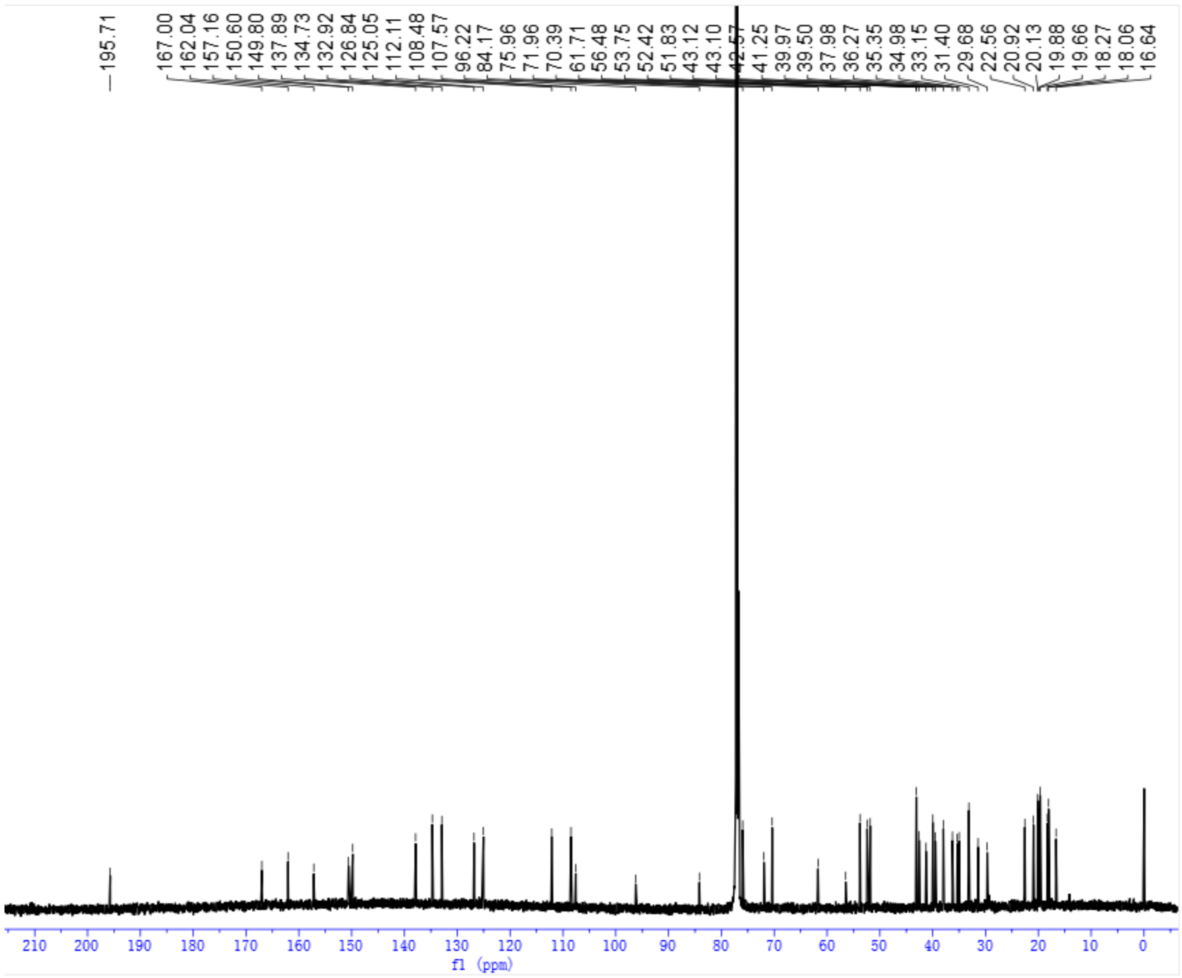

Figure S56: ^{13}C NMR spectrum of **10b** in $CDCl_3$ (151 MHz)

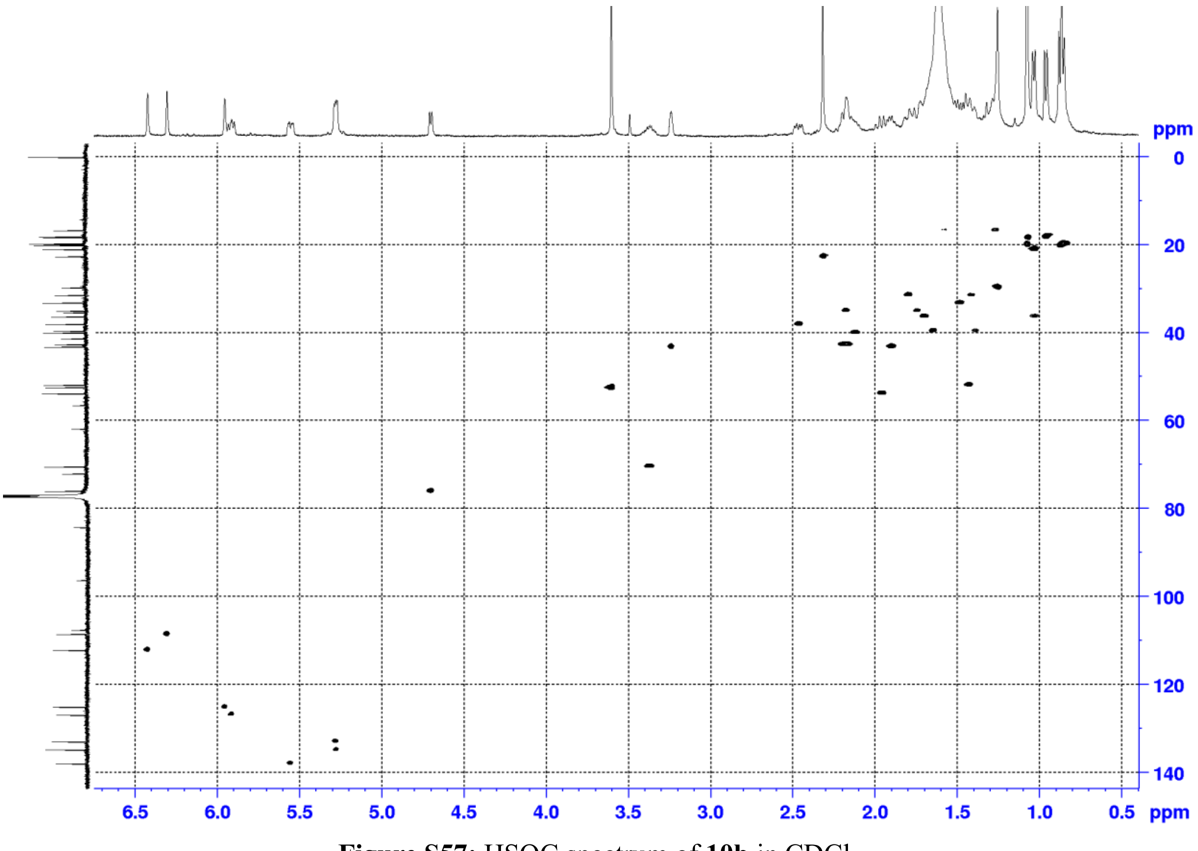

Figure S57: HSQC spectrum of **10b** in $CDCl_3$

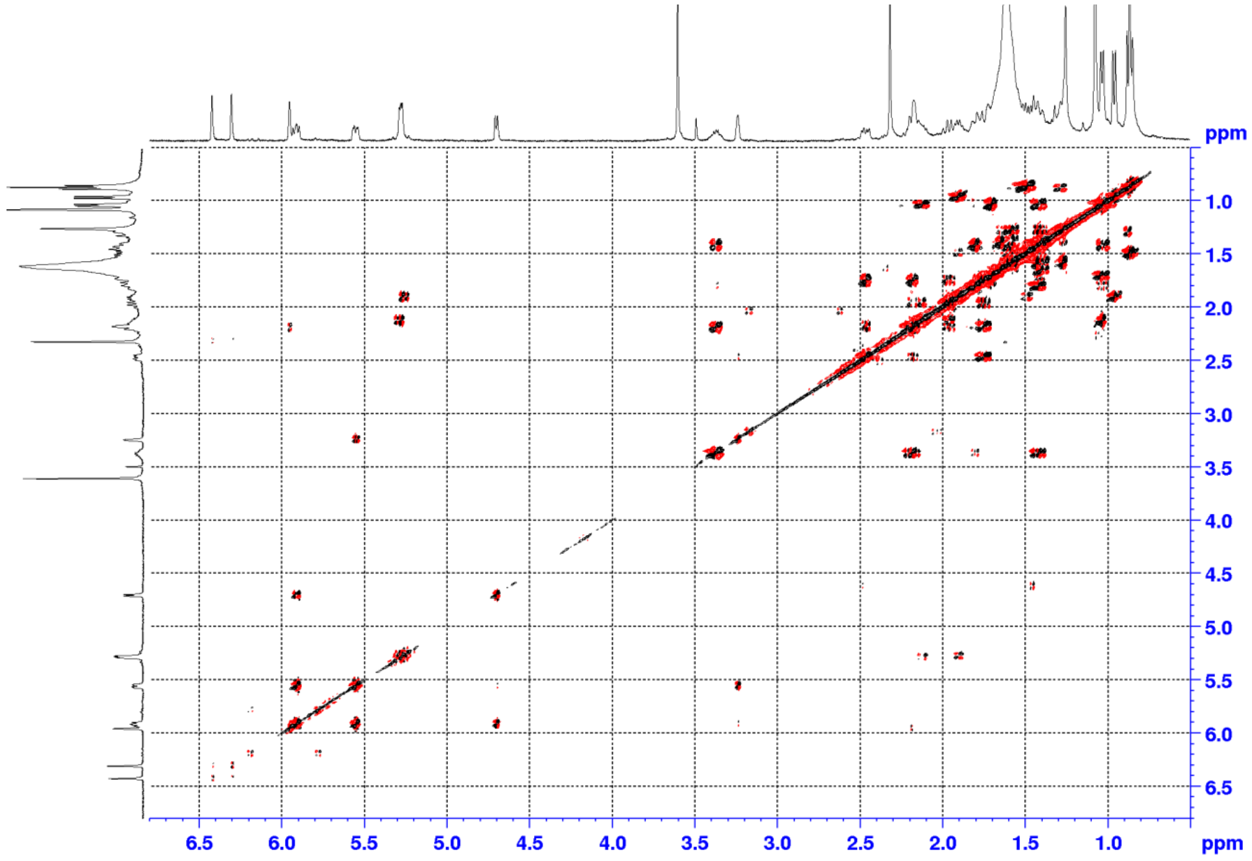

Figure S58: COSY spectrum of **10b** in $CDCl_3$

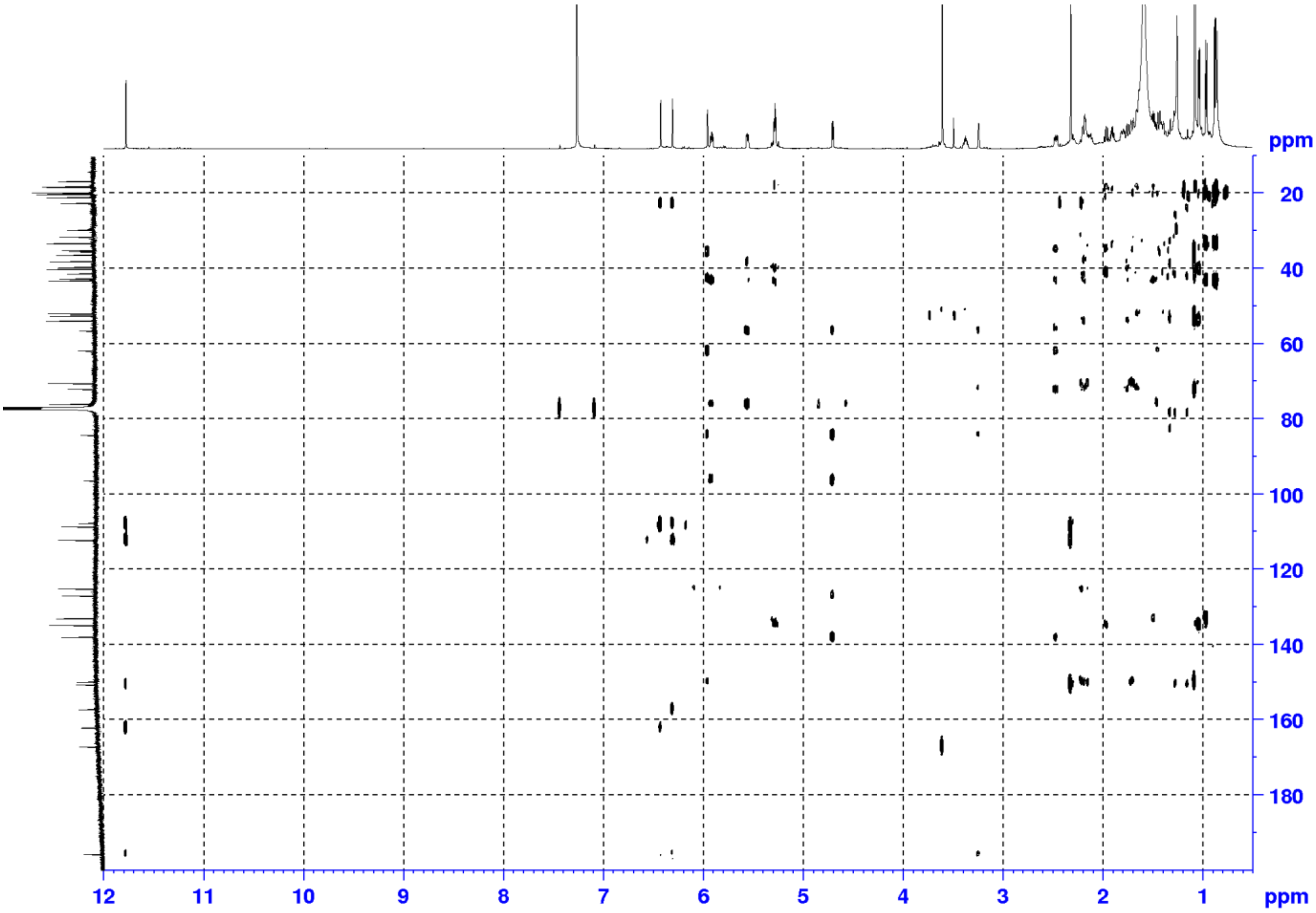

Figure S59: HMBC spectrum of **10b** in $CDCl_3$

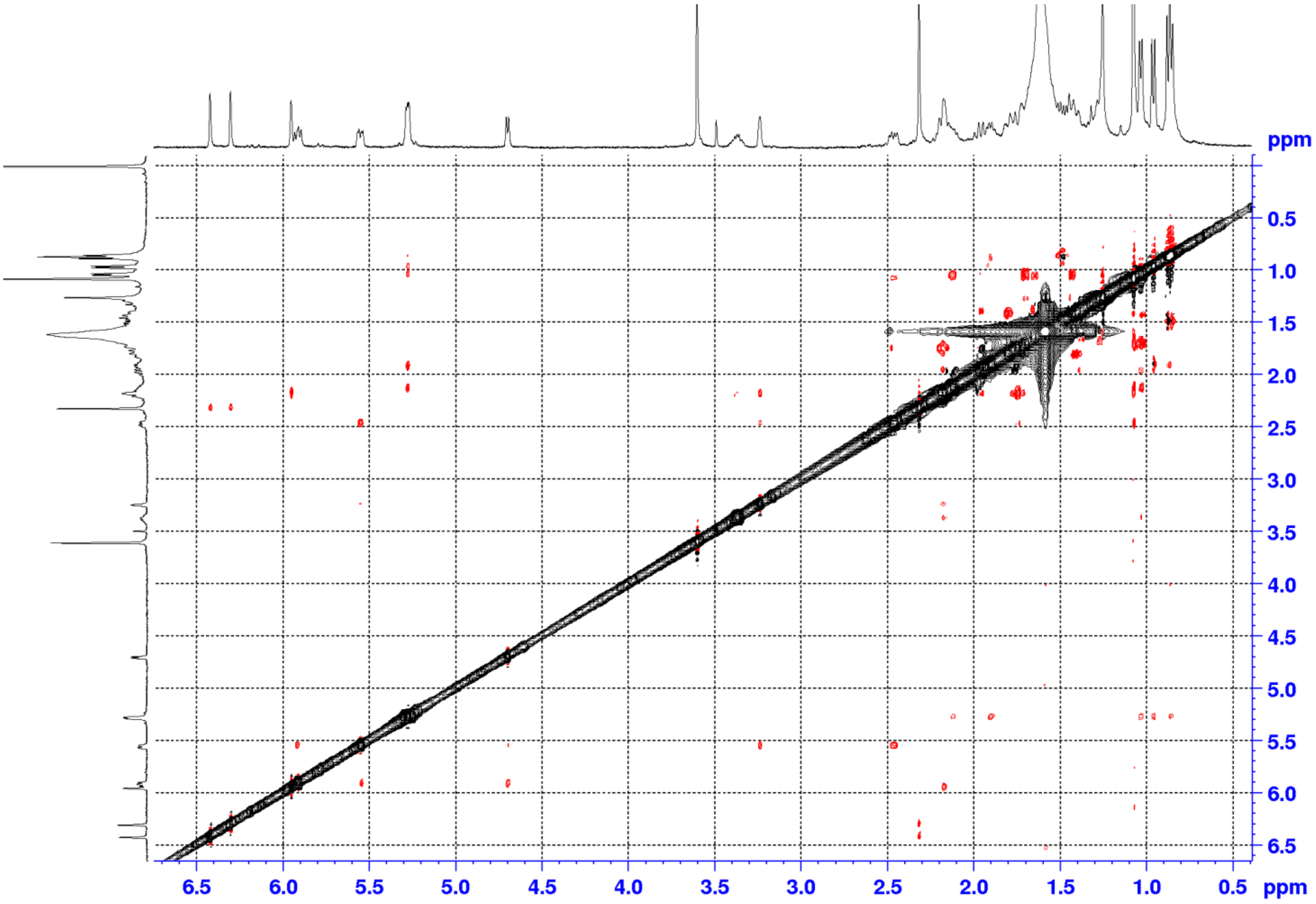

Figure S60: NOESY spectrum of **10b** in $CDCl_3$

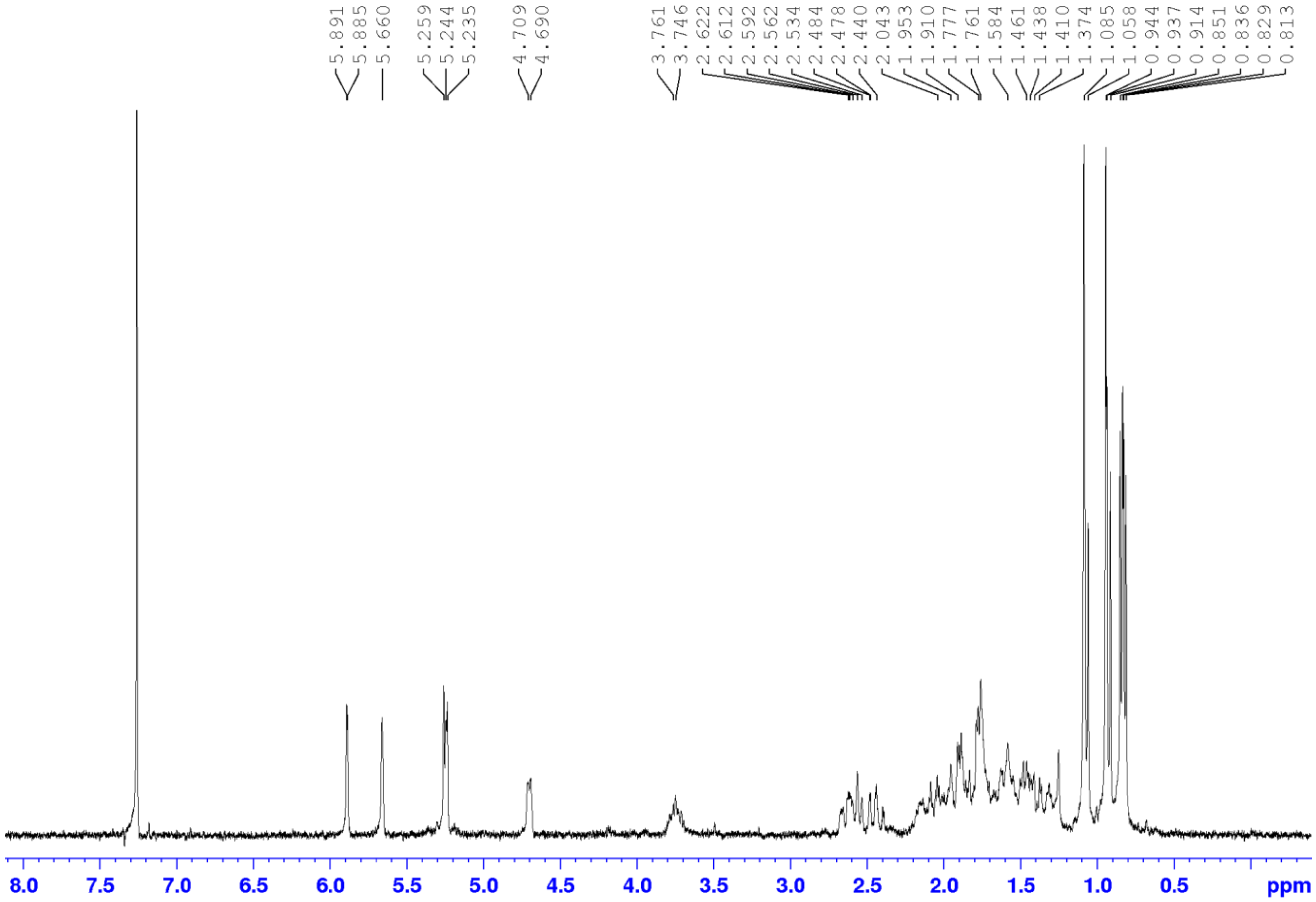

Figure S61: ^{1}H NMR spectrum of **11** in $CDCl_3$ (300 MHz)

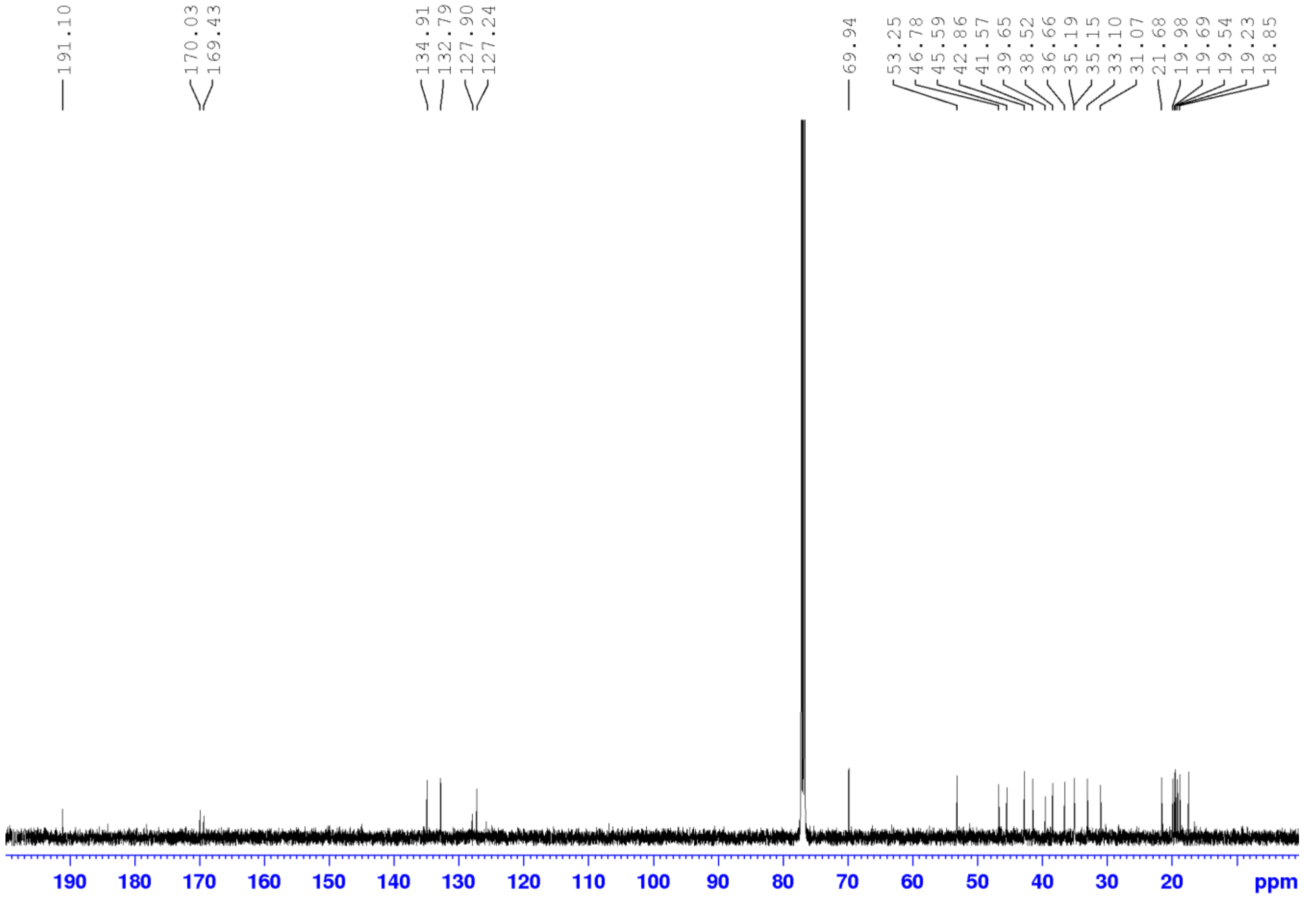

Figure S62: ^{13}C NMR spectrum of **11** in $CDCl_3$ (126 MHz)

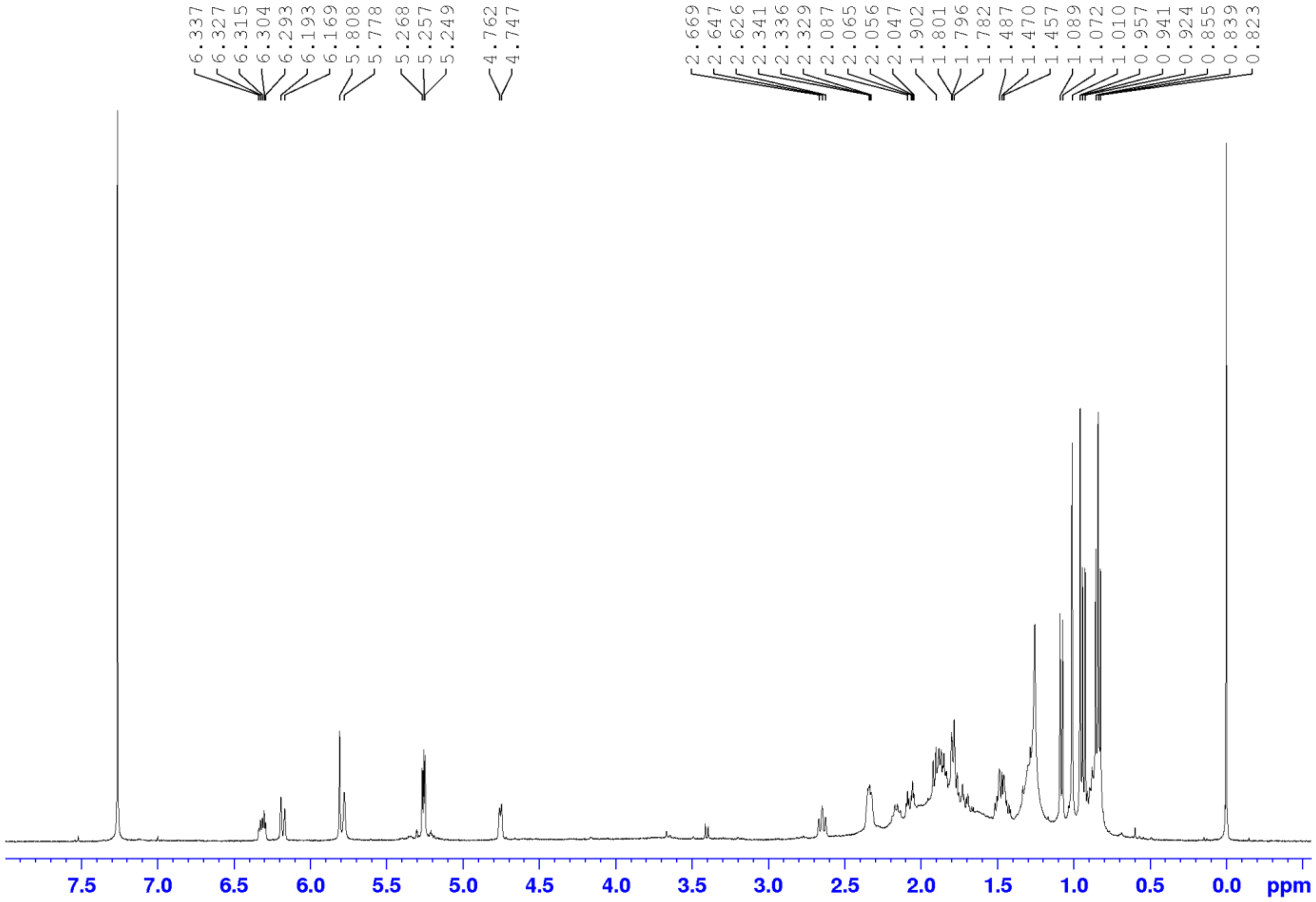

Figure S63: ^{1}H NMR spectrum of **12** in $CDCl_3$ (400 MHz)

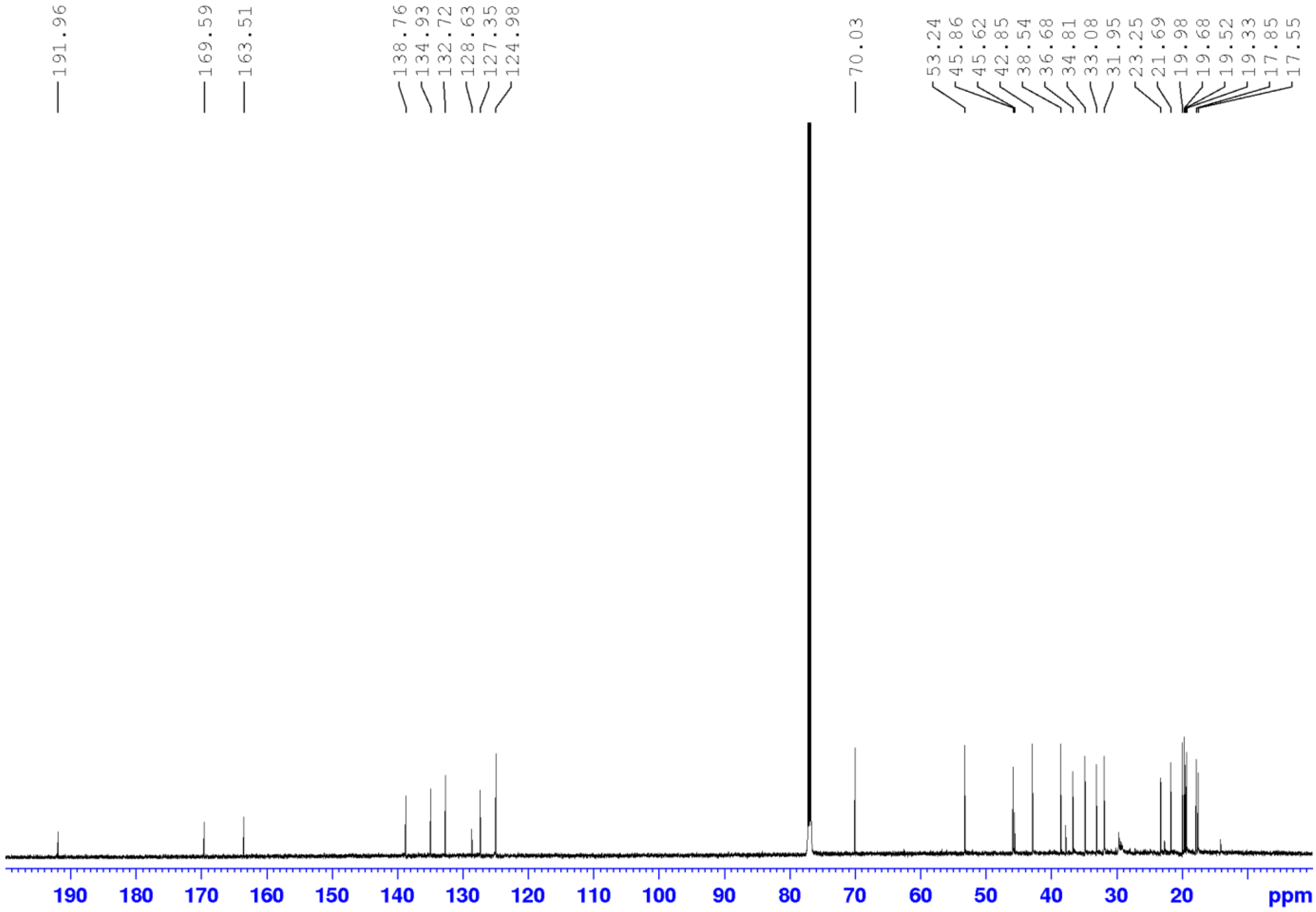

Figure S64: ^{13}C NMR spectrum of **12** in $CDCl_3$ (151 MHz)

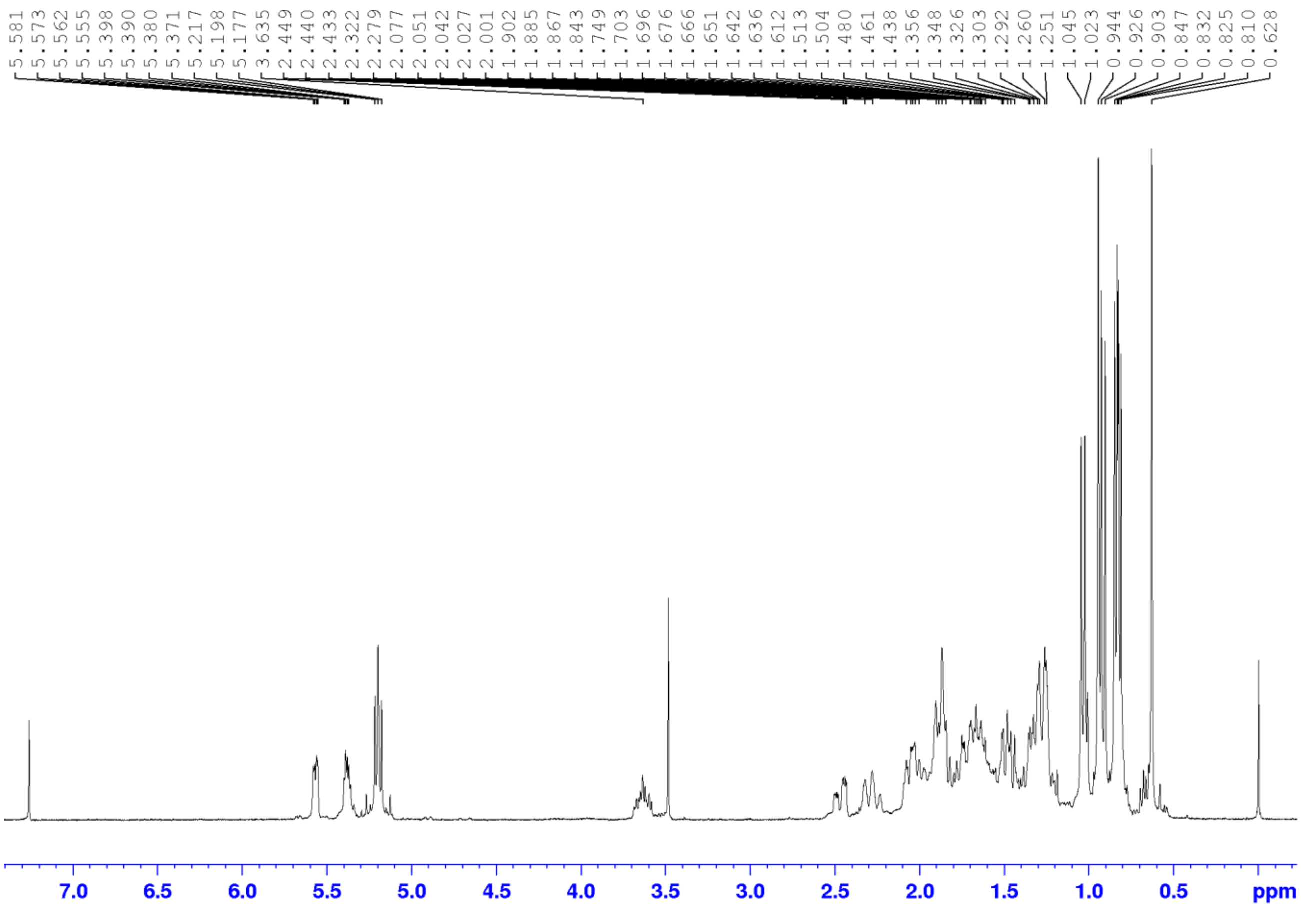

Figure S65: ^{1}H NMR spectrum of **13** in $CDCl_3$ (300 MHz)

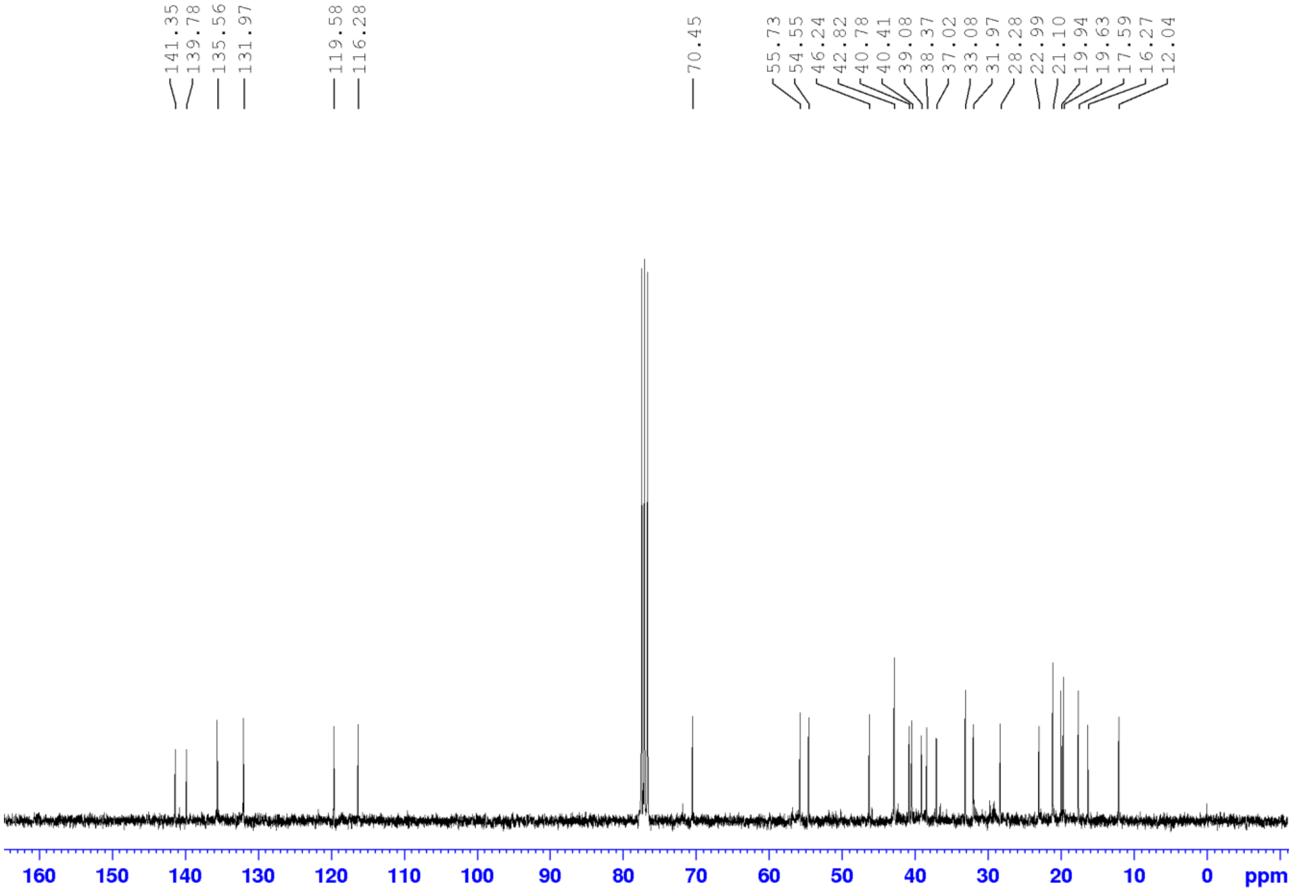

Figure S66: ^{13}C NMR spectrum of **13** in $CDCl_3$ (75.5 MHz)

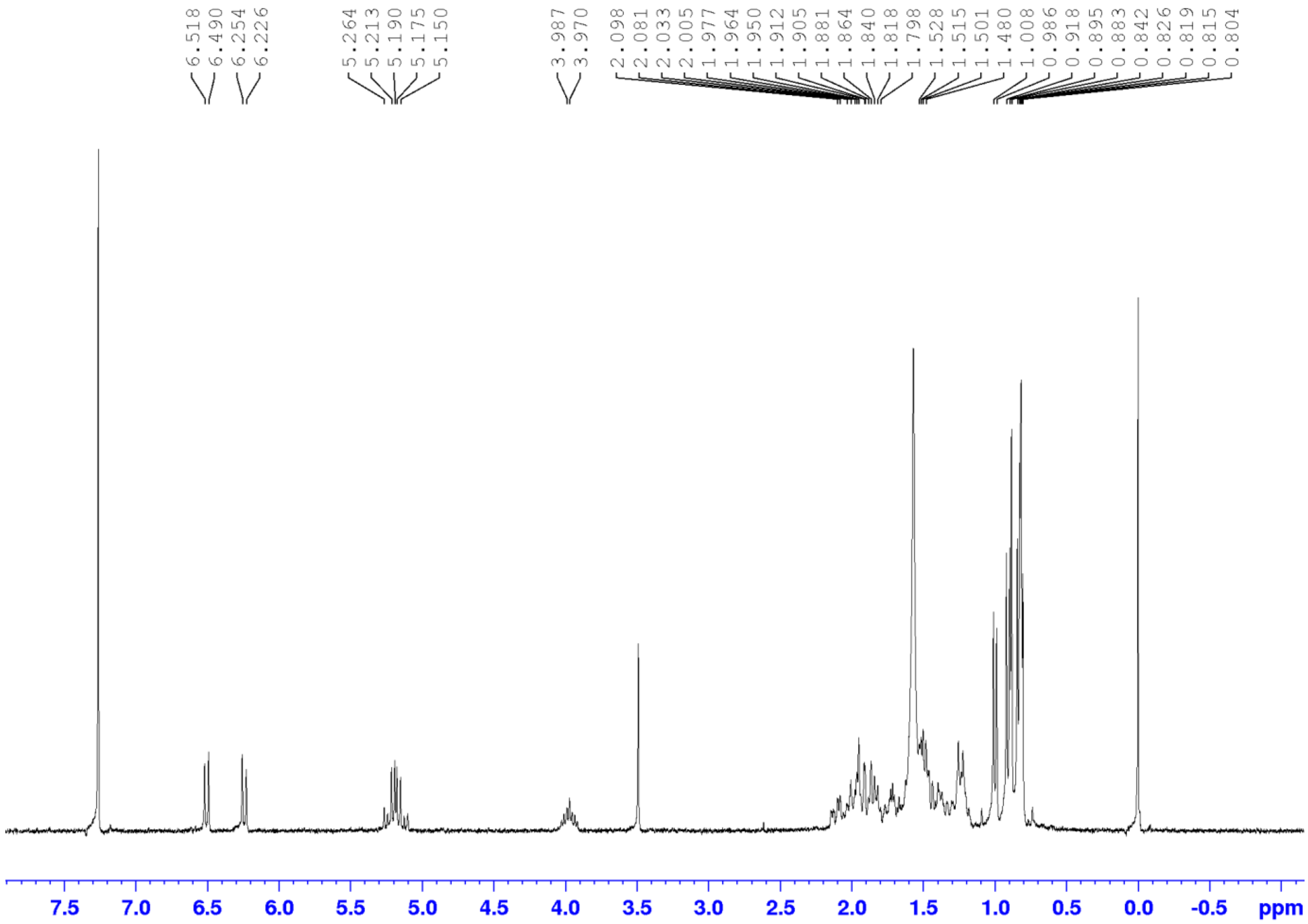

Figure S67: ^{1}H NMR spectrum of **14** in $CDCl_3$ (400 MHz)

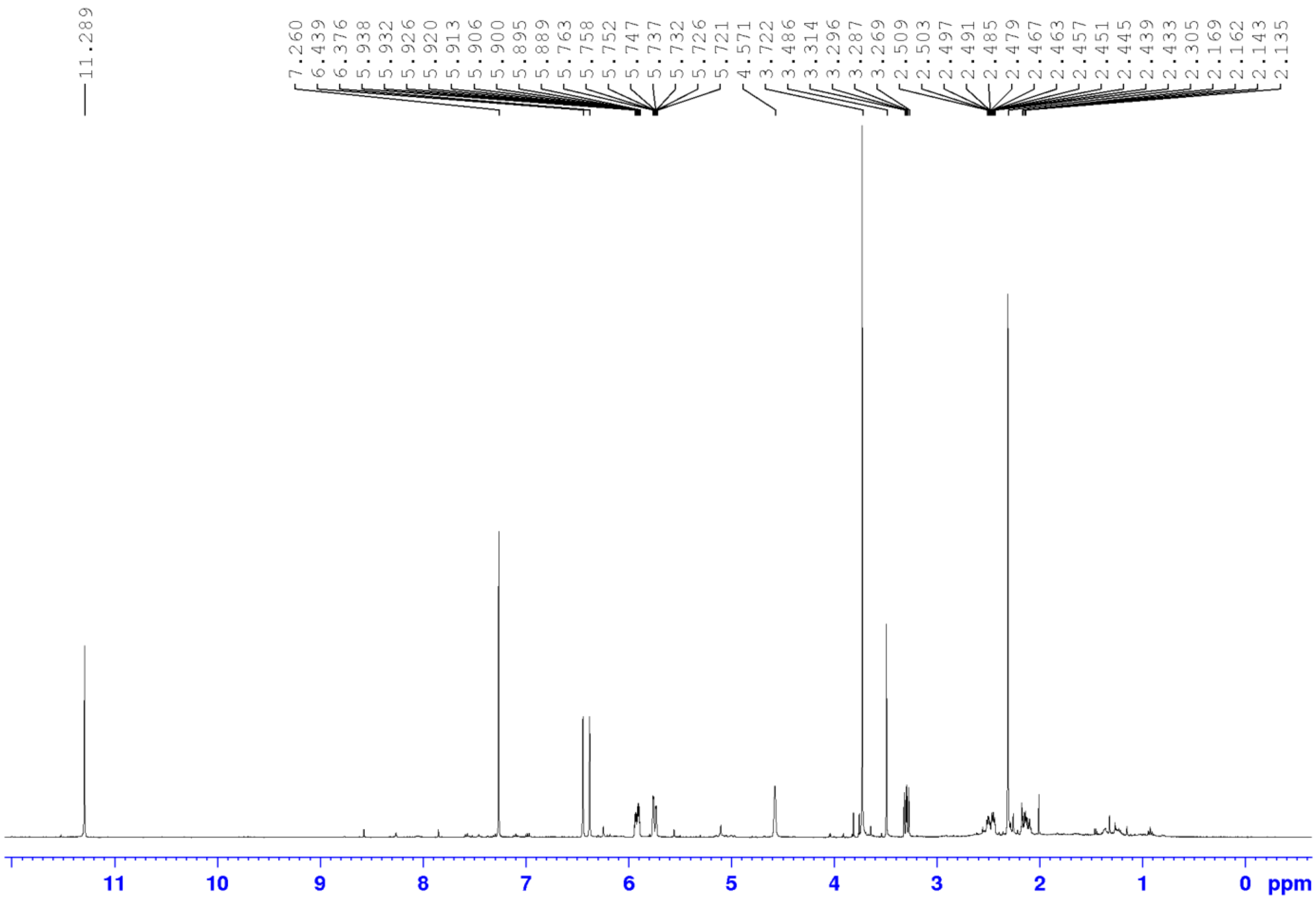

Figure S68: ^{1}H NMR spectrum of **15** in $CDCl_3$ (400 MHz)

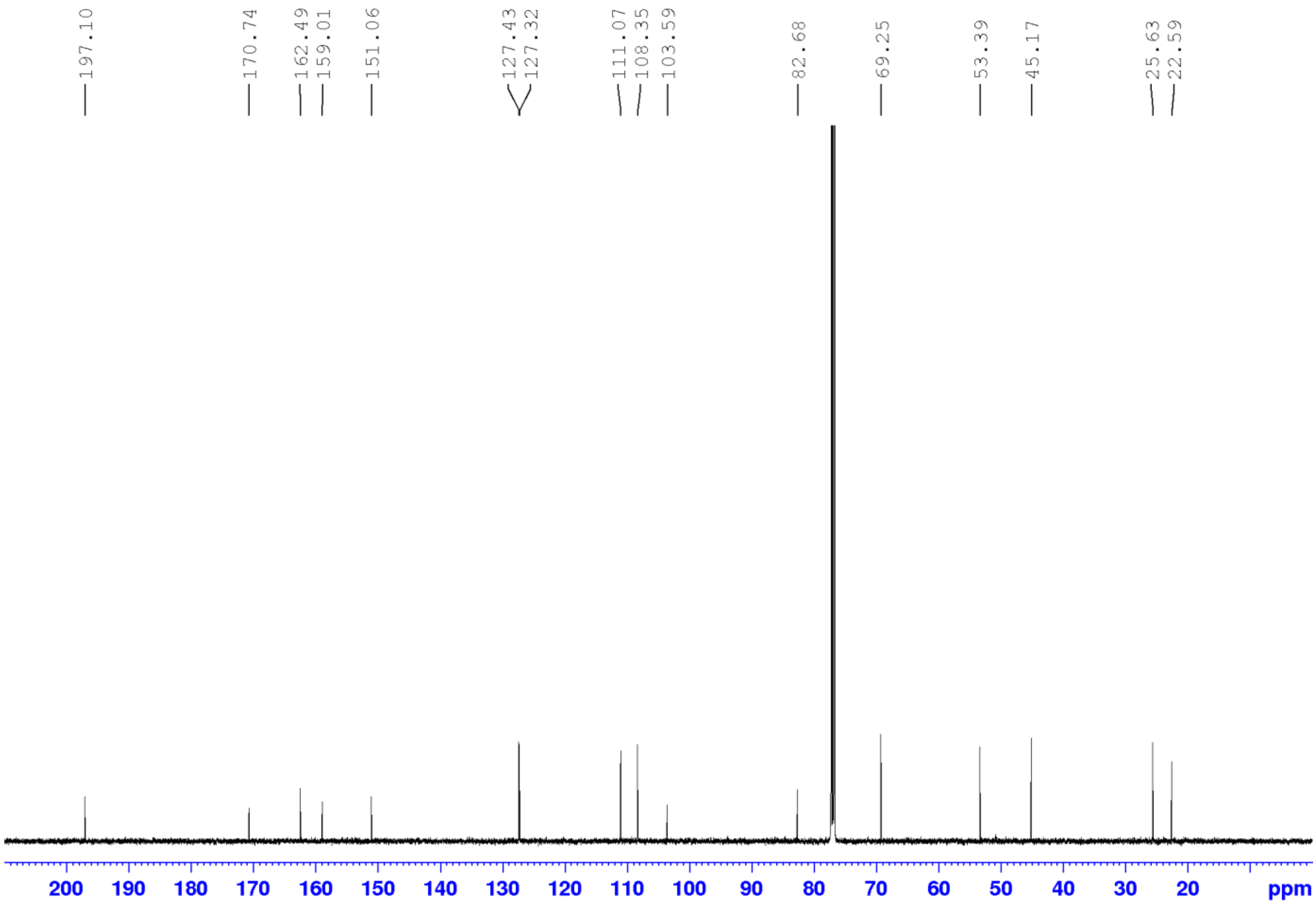

Figure S69: ^{13}C NMR spectrum of **15** in $CDCl_3$ (126 MHz)

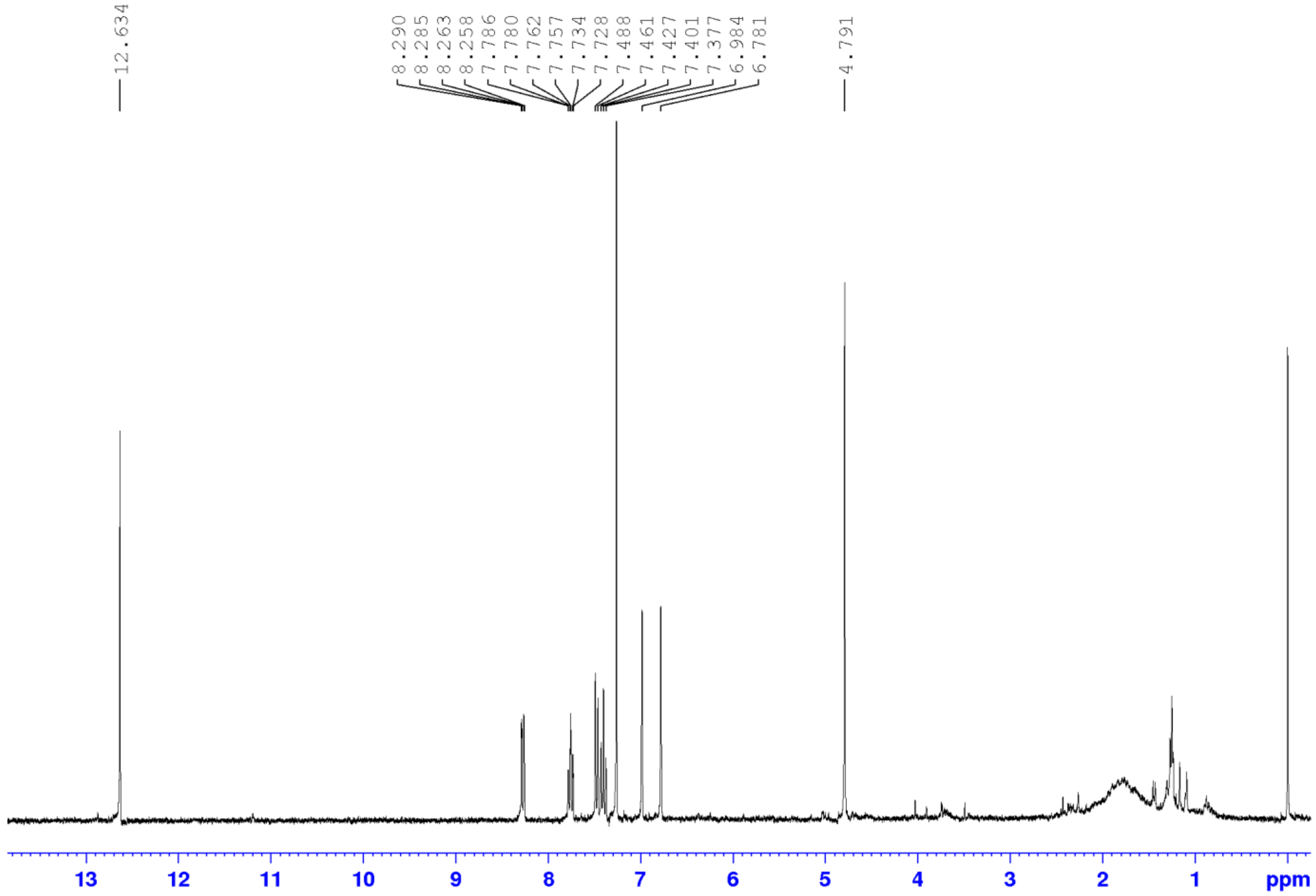

Figure S70: ^{1}H NMR spectrum of **16** in $CDCl_3$ (300 MHz)

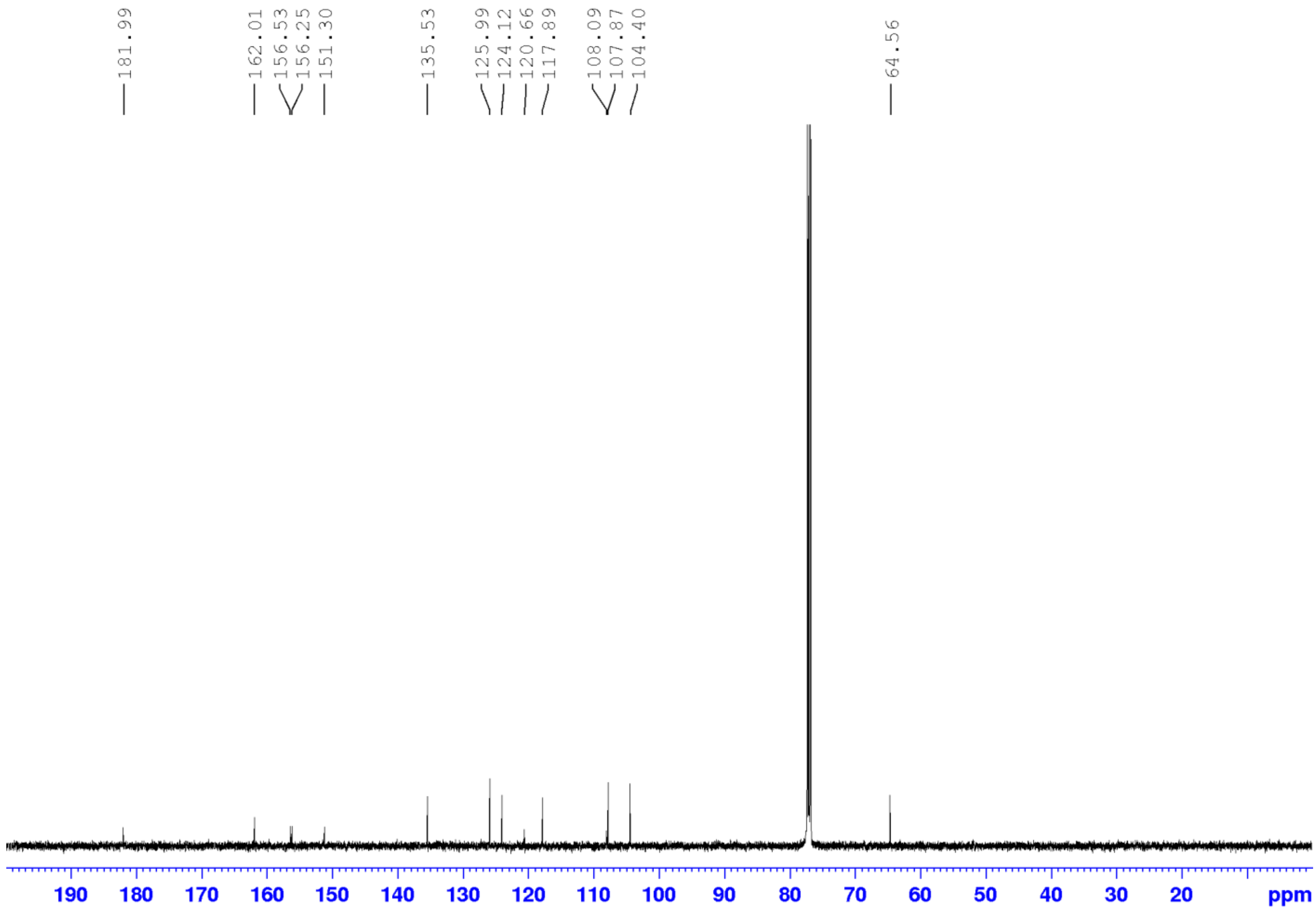

Figure S71: ^{13}C NMR spectrum of **16** in $CDCl_3$ (126 MHz)

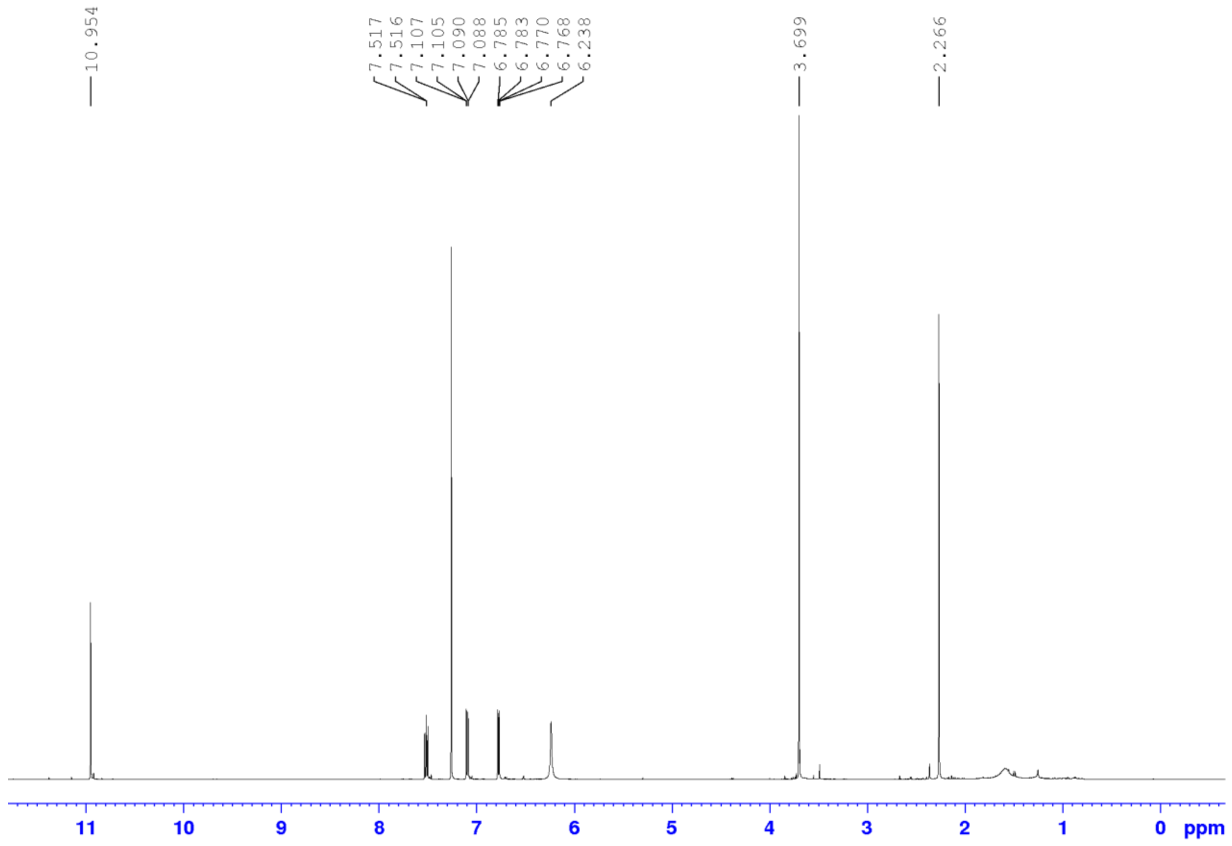

Figure S72: ^{1}H NMR spectrum of **17** in $CDCl_3$ (500 MHz)

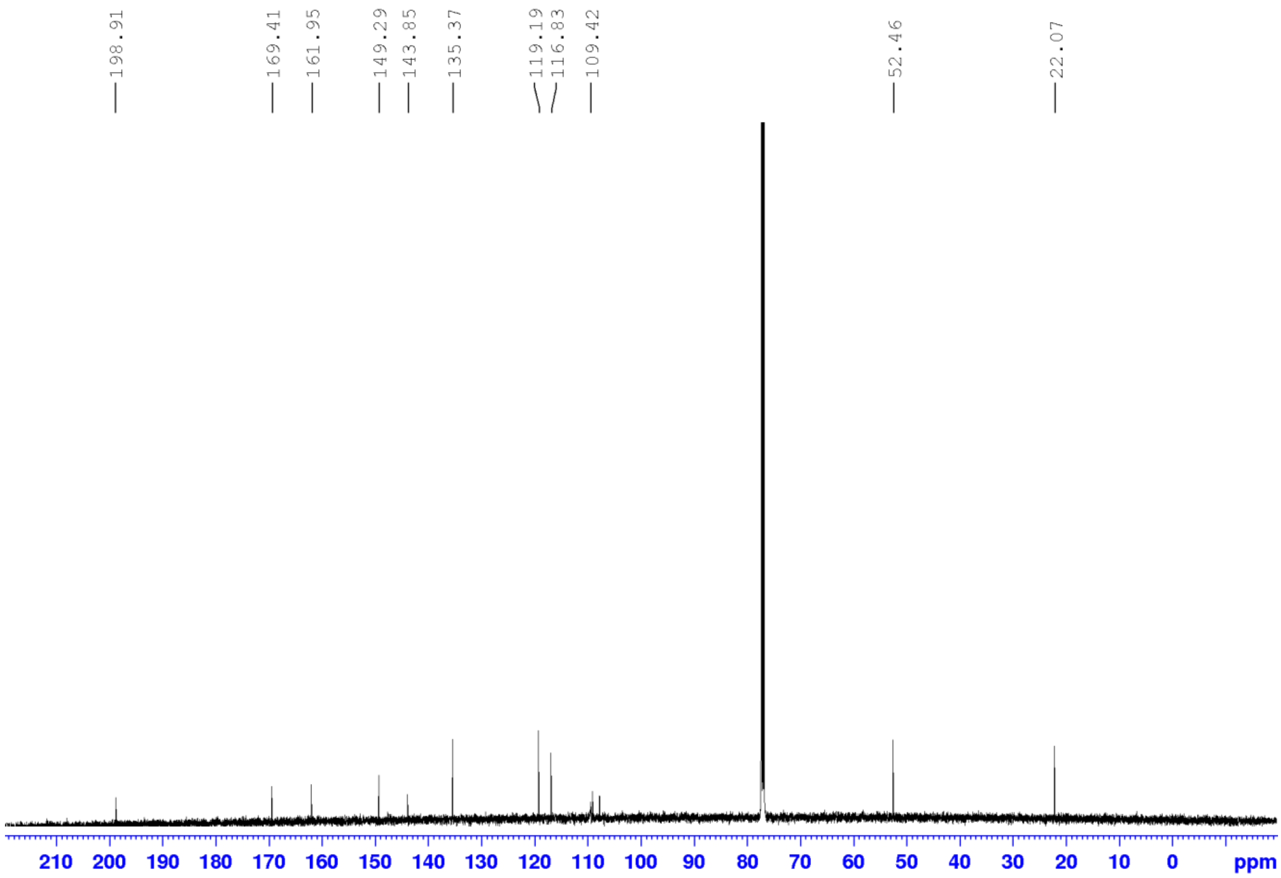

Figure S73: ^{13}C NMR spectrum of **17** in $CDCl_3$ (126 MHz)

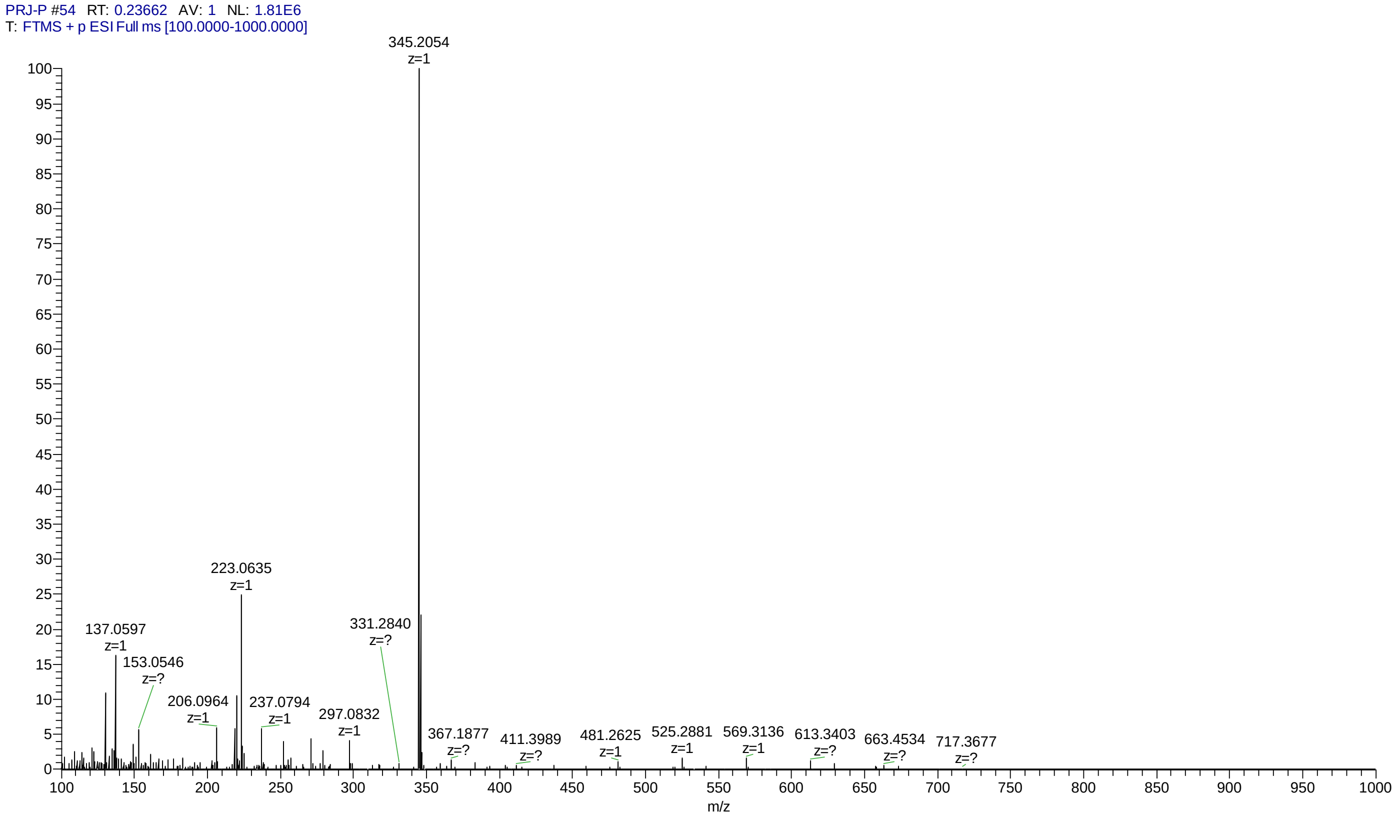

Figure S74: HRESIMS spectrum of compound **1**

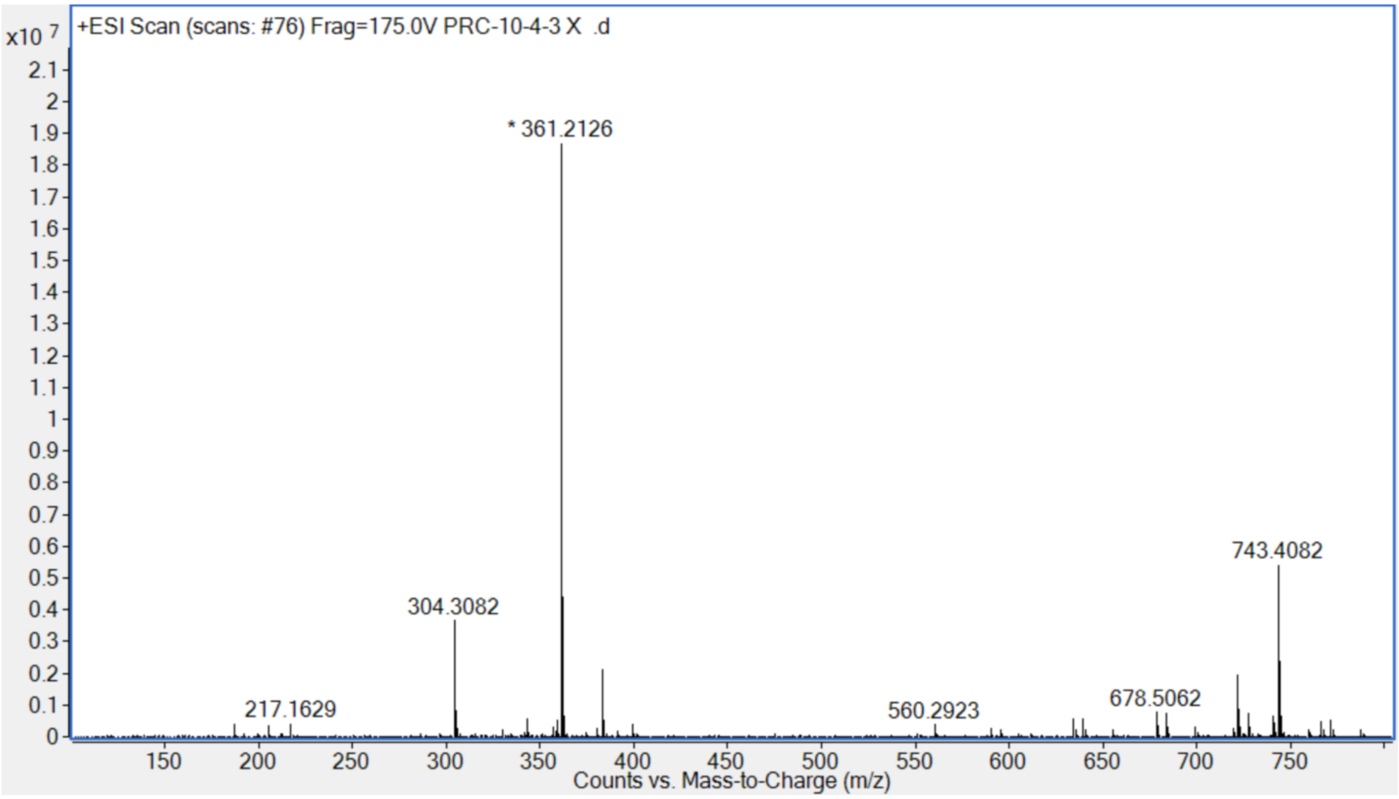

Figure S75: HRESIMS spectrum of compound **2**

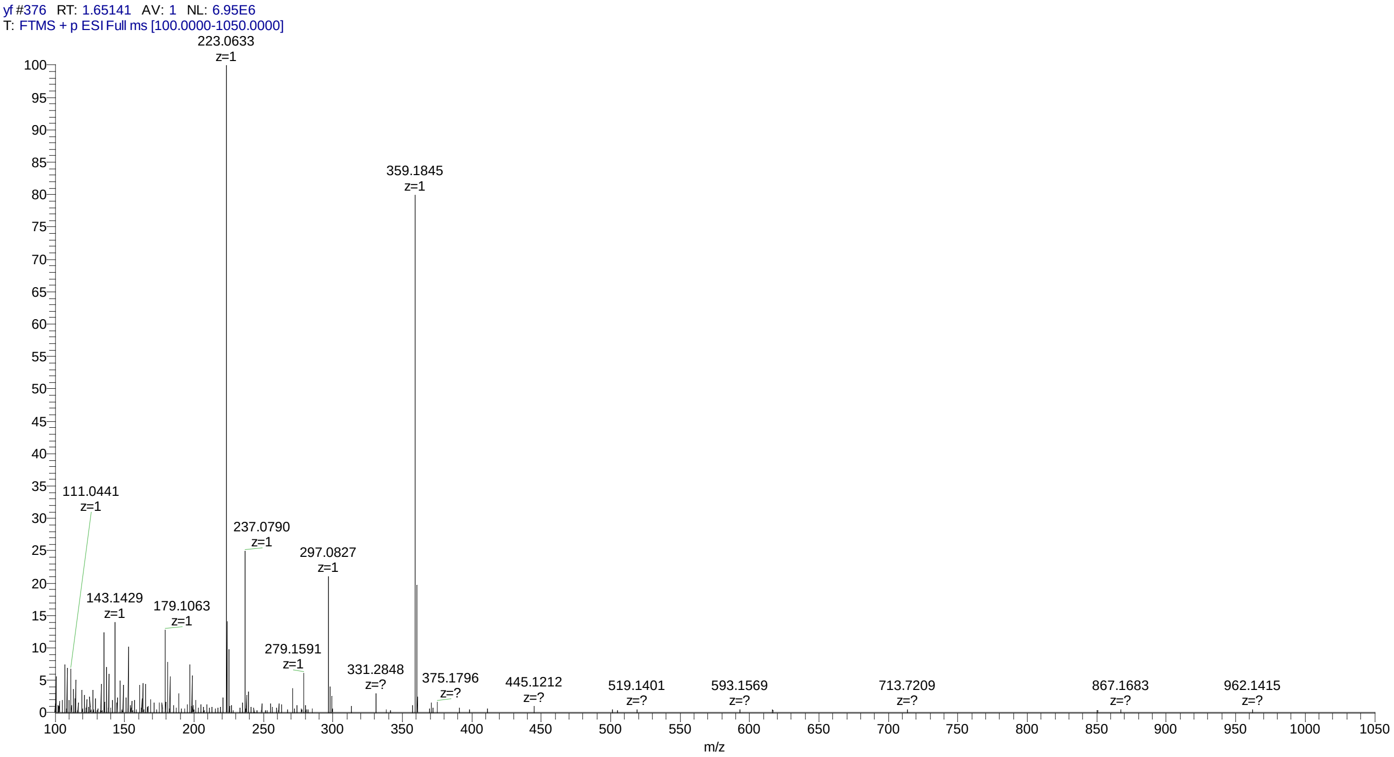

Figure S76: HRESIMS spectrum of compound **3**

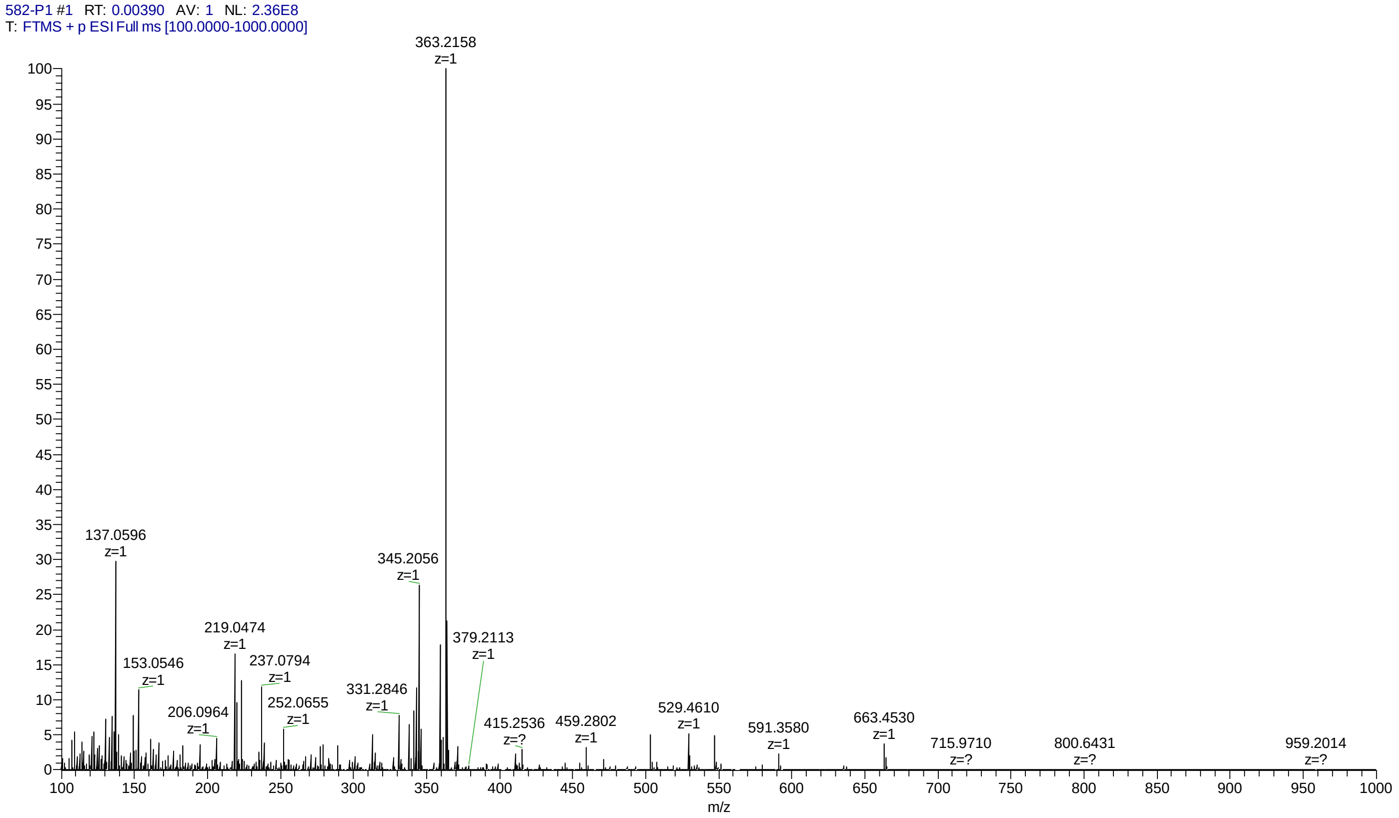

Figure S77: HRESIMS spectrum of compound **4**

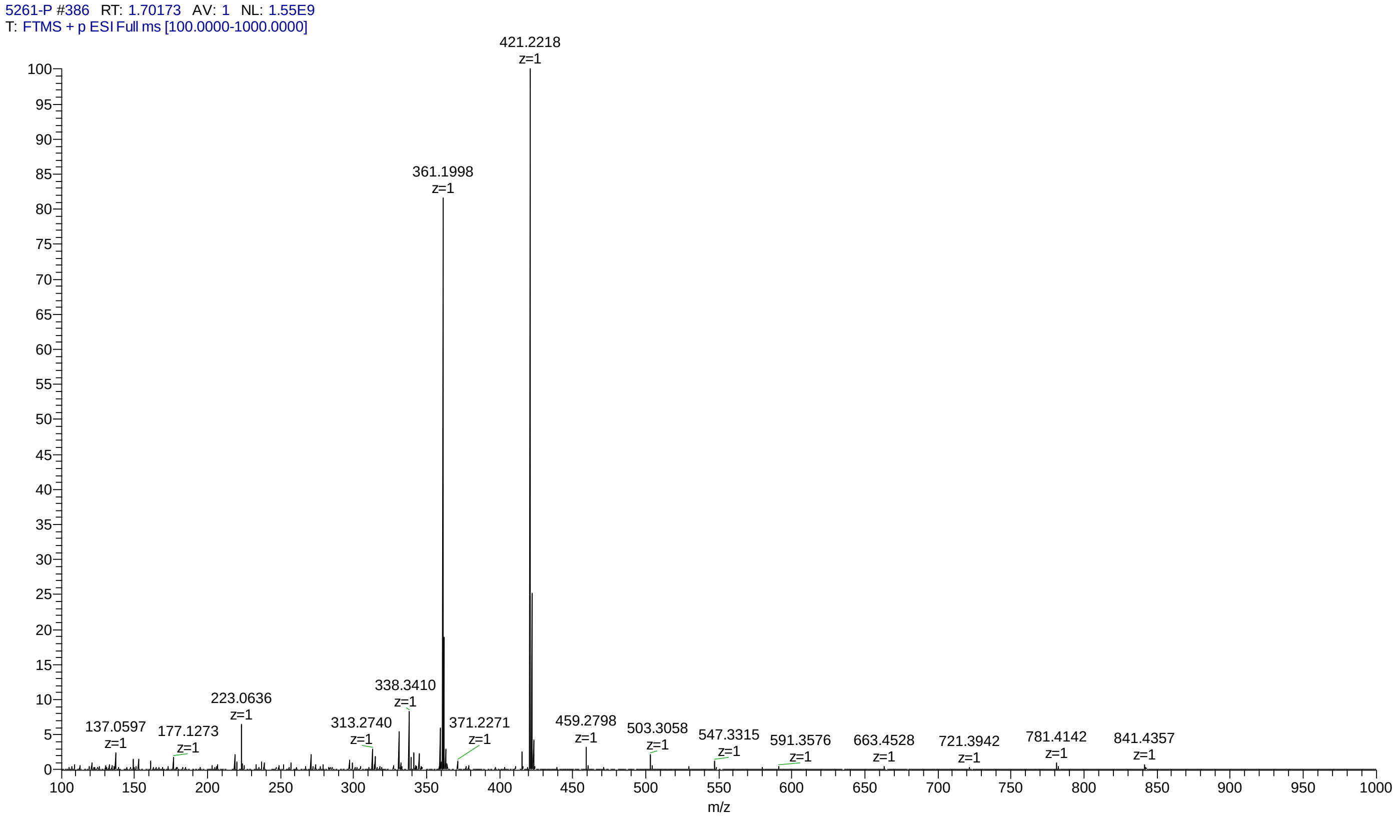

Figure S78: HRESIMS spectrum of compound **5**

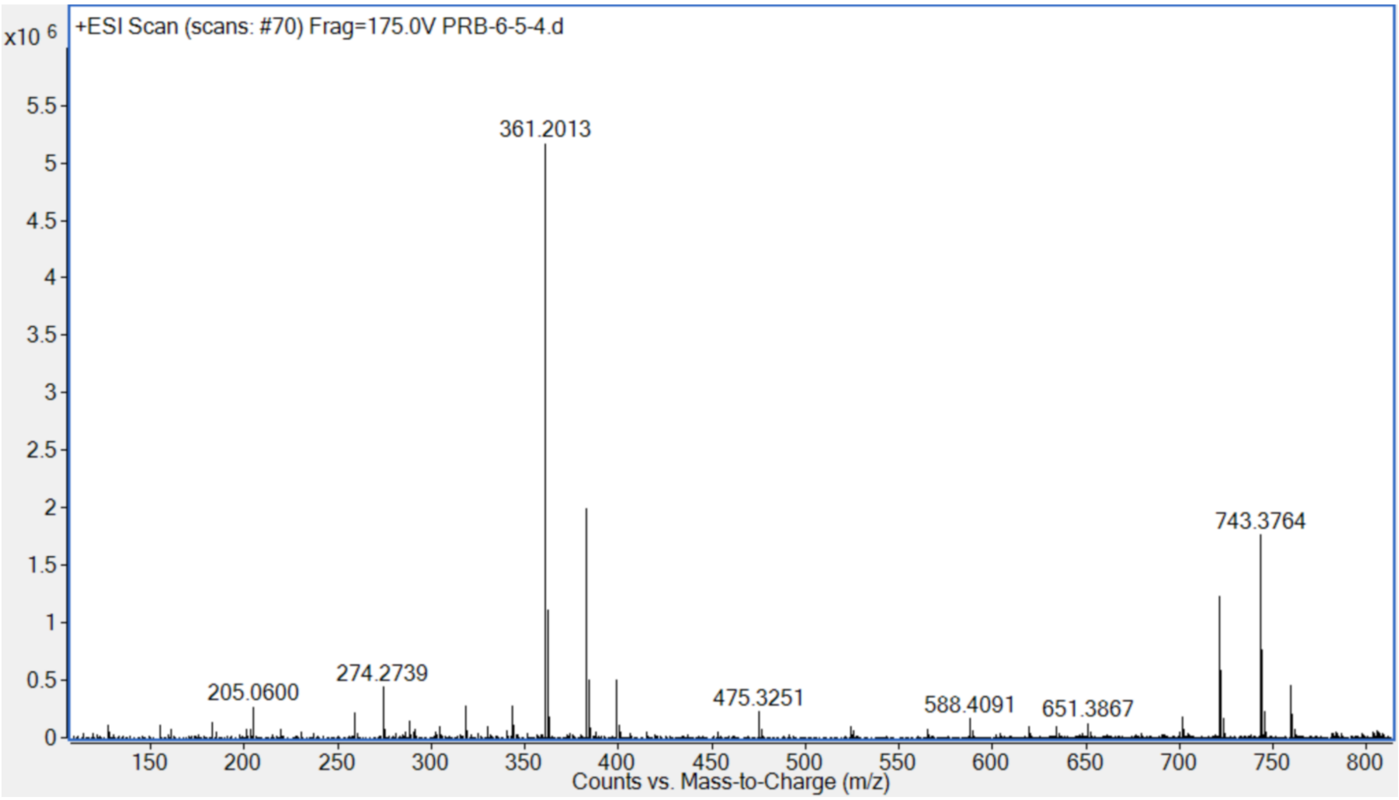

Figure S79: HRESIMS spectrum of compound **6**

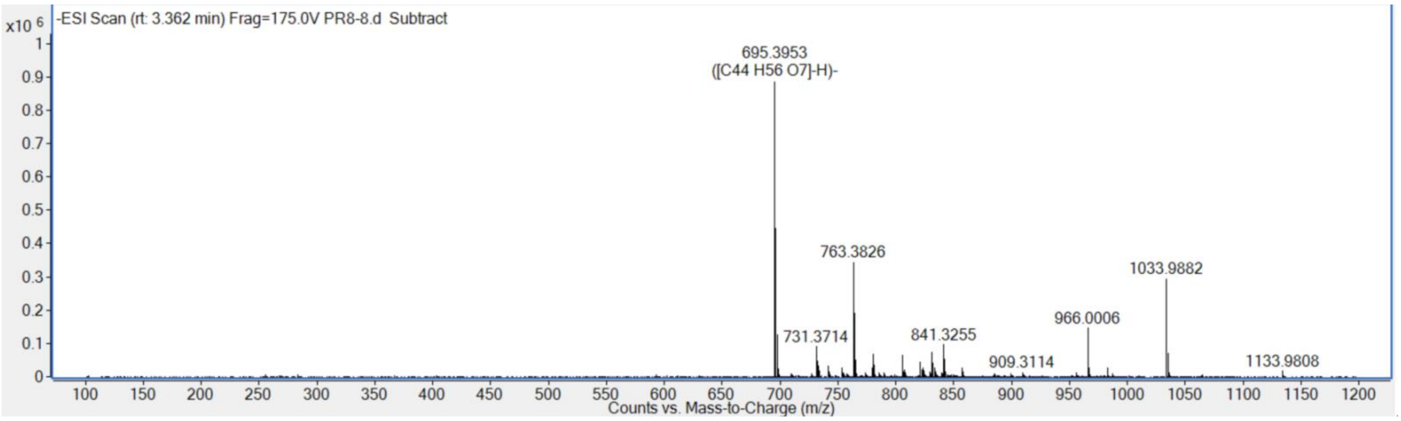

Figure S80: HRESIMS spectrum of compound **10**

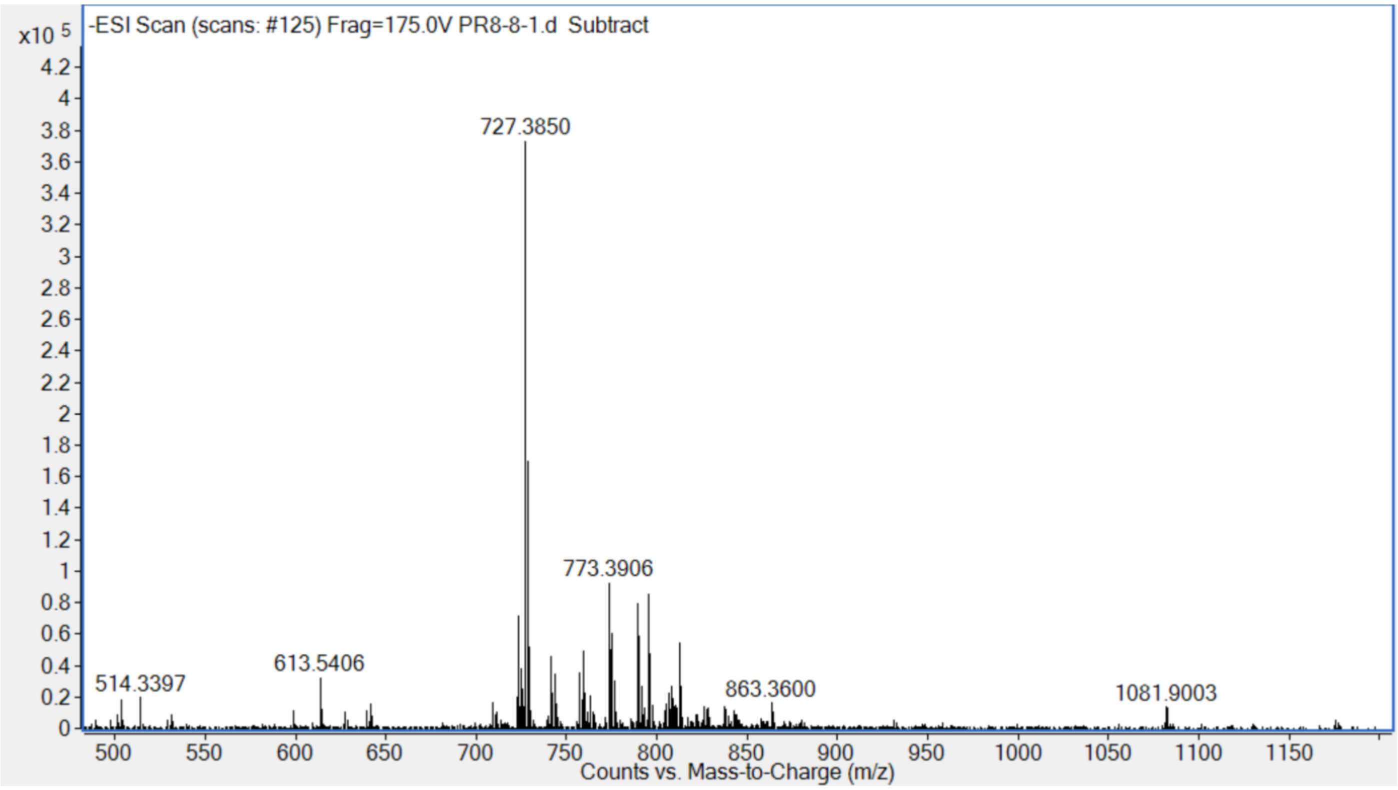

Figure S81: HRESIMS spectrum of compound **10a**

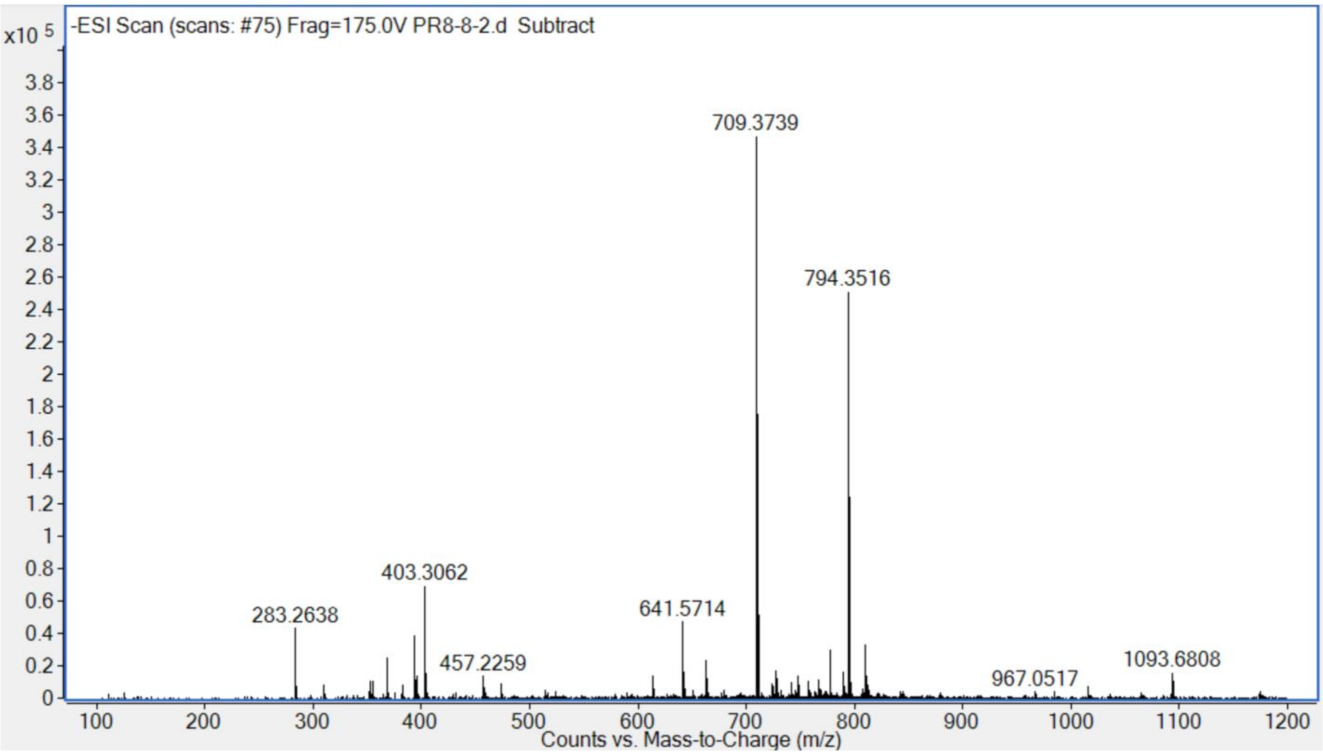

Figure S82: HRESIMS spectrum of compound **10b**

Table S1: Shielding sensors (σ_C) of the conformer (**2**) and the calculated chemical shifts (δ_C).

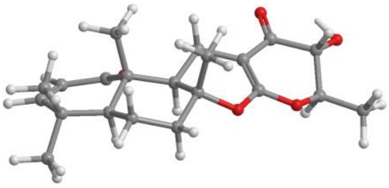
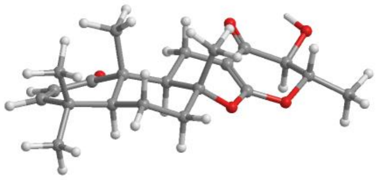

2I 2II

No.	Exp.	Shielding Tensor		Cal. for **2I**
		**2I**	**2II**	
1	204.2	-26.5499	-27.56	204.095
2	123.9	56.6528	56.91	124.2996
3	154.8	20.1338	19.07	159.3231
4	36.3	144.1495	144.14	40.38602
5	50.9	133.1427	133.27	50.94207
6	19.8	164.0273	164.26	21.32224
7	38.9	145.8471	146.02	38.75794
8	84	99.4094	98.81	83.29395
9	43.6	140.7234	140.27	43.67181
10	47.4	134.2041	134.51	49.92414
11	17.4	167.4574	167.11	18.03261
12	20.3	166.8067	165.24	18.65666
13	21.8	165.2899	165.60	20.11135
14	31.1	156.731	157.12	28.31975
15	15	170.8134	170.51	14.81404
1′	168.3	11.065	9.57	168.0205
2′	87.9	94.5773	94.53	87.92817
3′	191.2	-9.8752	-10.89	188.1032
4′	71.6	114.1102	114.10	69.19517
5′	79.6	103.078	102.60	79.77558
6′	17.9	168.5302	168.17	17.00374

Table S2: DP4$^+$ probability analysis for isomers 2I and 2II

A	B	C	D	E	F	G	H
Functional		Solvent?		Basis Set		Type of Data	
mPW1PW91		PCM	PCM	6-311+G(d,p)		Shielding Tensors	
		DP4+	70.37%	29.63%	-	-	-
Nuclei	sp2?	xperimenta	Isomer 1	Isomer 2	Isomer 3	Isomer 4	Isomer 5
C	x	204.2	-26.55	-27.56			
C	x	123.9	56.65	56.91			
C	x	154.8	20.13	19.07			
C		36.3	144.15	144.14			
C		50.9	133.14	133.27			
C		19.8	164.03	164.26			
C		38.9	145.85	146.02			
C		84	99.41	98.81			
C		43.6	140.72	140.27			
C		47.4	134.20	134.51			
C		17.4	167.46	167.11			
C		20.3	166.81	165.24			
C		21.8	165.29	165.60			
C		31.1	156.73	157.12			
C		15	170.81	170.51			
C	x	168.3	11.07	9.57			
C	x	87.9	94.58	94.53			
C	x	191.2	-9.88	-10.89			
C		71.6	114.11	114.10			
C		79.6	103.08	102.60			
C		17.9	168.53	168.17			

Table S3: Shielding sensors (σ_C) of the conformer (**10I**) and the calculated chemical shifts (δ_C).

No.	Exp.	Shielding Tensor					Cal.
		C1	C2	C3	C4	Averaged	
1	36.2	148.8547	148.7579	148.632	148.4732	148.7781	35.30826
2	31.8	151.6775	151.5686	154.8622	154.8172	152.771	31.50228
3	71.5	113.0808	112.8838	112.5291	112.4622	112.8838	69.52266
4	42.5	144.3746	144.4256	140.8598	140.9022	143.1928	40.63214
5	143.5	32.0955	33.8248	32.0327	33.5751	32.29224	146.3423
6	121.8	58.2993	55.4432	58.242	55.253	57.91054	121.923
7	41.4	140.5732	140.5178	140.8598	140.5929	140.6686	43.0382
8	124.9	52.9656	51.8032	53.0584	51.6775	52.84263	126.7538
9	46.8	135.9799	135.7556	135.9227	135.7762	135.9485	47.53746
10	35.5	146.198	146.2516	146.0021	146.0911	146.1541	37.80944
11	19.6	164.3672	164.4995	164.5157	164.5797	164.4485	20.37131
12	37.7	146.8045	146.9087	146.8857	146.9987	146.8608	37.13581
13	42.7	138.7805	138.7904	138.5706	138.7241	138.7303	44.8858
14	149.4	27.6412	28.5693	27.2838	28.3	27.64557	150.7715
15	41.5	140.9017	140.883	140.9297	141.0401	140.9288	42.79024
16	37.2	145.6205	146.0647	145.6526	146.0166	145.6998	38.24247
17	54.1	130.4044	130.2429	130.2229	130.1219	130.3372	52.88609
18	19.6	166.9308	167.0665	167.0673	167.2377	167.0133	17.92652
19	18.9	167.5803	167.6747	167.3159	167.3626	167.5167	17.44671
20	39.6	141.3345	141.3917	141.6173	141.7138	141.4545	42.28907
21	21.1	164.5353	164.6647	165.1198	165.2308	164.7676	20.0671
22	135.8	41.4938	41.3154	43.6201	43.4531	42.20282	136.8956
23	132	50.4456	50.4706	45.5852	45.642	48.79256	130.6143
24	42.8	138.9706	138.7904	140.0531	139.8596	139.3309	44.31328
25	33.1	149.3887	149.2821	148.5757	148.4732	149.1119	34.99007
26/27	19.75	168.0915	164.2829	172.2664	172.2402	168.2754341	16.72344
	19.75	168.0915	171.7768	164.927	164.9304		
28	17.6	165.8517	165.8648	173.6	173.6387	168.5218	16.48859
1′	46.1	134.8012	134.3664	135.0164	134.3196	134.8203	48.61278
2′	138.1	37.316	39.0169	37.2079	38.172	37.46935	141.4075
3′	119.8	59.3281	57.7122	59.4074	58.5144	59.18492	120.7083
4′	67.7	114.8418	114.2536	114.6172	114.2577	114.7107	67.7812
4′a	106.9	76.5738	75.9213	76.5641	75.82	76.48971	104.2134
5′	148.7	29.4778	30.0358	29.8387	30.1857	29.66681	148.8449
5′a	110.2	72.1032	72.057	72.1983	72.3176	72.14442	108.3553
6′	160.4	18.5175	18.6052	18.5274	18.6808	18.53703	159.4538
7′	198.1	-19.9629	-19.9485	-19.8333	-19.7539	-19.9158	196.1069
8′	88.5	93.2717	94.1023	92.9342	93.8113	93.27497	88.21373
8′a	157.8	22.7976	22.9991	23.3249	23.134	22.98864	155.2105
9′	107	75.346	75.652	75.4806	75.82	75.44062	105.2134
9′a	54.9	127.1754	127.0967	127.0548	127.0581	127.1404	55.93329
10′	22.3	163.2527	163.2032	163.6185	163.5219	163.3857	21.38438
11′	170.2	6.4066	6.9985	6.4591	6.9943	6.499123	170.9283
12′	53.1	132.4406	133.1546	132.4253	133.1954	132.5434	50.78316

Table S4: Shielding sensors (σ_C) of the conformer (**10II**) and the calculated chemical shifts (δ_C).

No.	Exp.	Shielding Tensor	Cal.
1	36.3	148.3349	35.88341
2	31.4	152.4049	31.98419
3	70.4	113.5769	69.18289
4	42.6	143.4487	40.56457
5	149.8	25.9468	153.1358
6	125.1	54.5915	125.6931
7	84.2	96.7599	85.29421
8	61.7	121.0991	61.97634
9	51.8	131.5138	51.99866
10	35.4	146.2486	37.88216
11	16.6	167.9991	17.04436
12	39.5	145.311	38.78042
13	41.3	140.3235	43.55863
14	72	109.863	72.74095
15	38	145.086	38.99598
16	35	149.4633	34.80236
17	53.8	132.8683	50.701
18	18.3	168.7708	16.30504
19	20	165.5241	19.4155
20	40	140.419	43.46714
21	20.9	164.7416	20.16517
22	134.7	41.51	138.2257
23	132.9	50.851	129.2767
24	43.1	138.535	45.27208
25	33.2	149.7391	34.53813
26/27	19.9	168.4241	16.63724
28	18.1	165.7044	19.24277
1′	43.1	139.946	43.92029
2′	137.9	39.8163	139.8483
3′	126.8	52.8241	127.3864
4′	76	107.9899	74.53545
4′a	96.2	86.1128	95.49454
5′	108.5	74.4244	106.6925
5′a	157.2	22.7703	156.1791
6′	150.6	27.6356	151.5179
7′	112.1	70.1614	110.7766
8′	162	17.066	161.644
8′a	107.6	75.3004	105.8532
9′	195.7	-16.2549	193.5667
9′a	56.5	125.5229	57.73817
10′	22.6	162.8001	22.0252
11′	167	10.9159	167.536
12′	52.4	133.1103	50.46915

Table S5: DP4+ probability analysis for isomers 10I and 10II

Functional		Solvent?		Basis Set		Type of Data	
mPW1PW91		PCM	PCM	6-311+G(d,p)		Shielding Tensors	
		DP4+	100.00%	0.00%	-	-	-
Nuclei	sp2?	xperimenta	Isomer 1	Isomer 2	Isomer 3	Isomer 4	Isomer 5
C		36.2	148.78	148.33			
C		31.8	152.77	152.40			
C		71.5	112.88	113.58			
C		42.5	143.19	143.45			
C	x	143.5	32.29	25.95			
C	x	121.8	57.91	54.59			
C		41.4	140.67	96.76			
C	x	124.9	52.84	121.10			
C		46.8	135.95	131.51			
C		35.5	146.15	146.25			
C		19.6	164.45	168.00			
C		37.7	146.86	145.31			
C		42.7	138.73	140.32			
C	x	149.4	27.65	109.86			
C		41.5	140.93	145.09			
C		37.2	145.70	149.46			
C		54.1	130.34	132.87			
C		19.6	167.01	168.77			
C		18.9	167.52	165.52			
C		39.6	141.45	140.42			
C		21.1	164.77	164.74			
C	x	135.8	42.20	41.51			
C	x	132	48.79	50.85			
C		42.8	139.33	138.54			
C		33.1	149.11	149.74			
C		19.75	168.275434	168.4241			
C		19.75	168.275434	168.4241			
C		17.6	168.5218	165.7044			
C		46.1	134.8203	139.946			
C	x	138.1	37.46935	39.8163			
C	x	119.8	59.18492	52.8241			
C		67.7	114.7107	107.9899			
C	x	106.9	76.48971	86.1128			
C	x	148.7	29.66681	74.4244			
C	x	110.2	72.14442	22.7703			
C	x	160.4	18.53703	27.6356			
C	x	198.1	-19.9158	70.1614			
C		88.5	93.27497	17.066			
C	x	157.8	22.98864	75.3004			
C	x	107	75.44062	-16.2549			
C		54.9	127.1404	125.5229			
C		22.3	163.3857	162.8001			
C	x	170.2	6.499123	10.9159			
C		53.1	132.5434	133.1103			

Table S6. Optimized cartesian coordinates of compound **1** for ECD calculation at the b3lyp/6-31+g(d,p) level.

Atom	Coordinates		
C	-3.41518300	2.09801700	-0.78966000
C	-4.36245300	1.15661000	-0.63529900
C	-4.09635600	-0.29570200	-0.33720000
C	-2.55621500	-0.58074500	-0.34914900
C	-1.63699200	0.56268400	0.23136800
C	-1.98412200	1.81165600	-0.60380000
C	-2.18413000	-1.95774600	0.22268400
C	-0.73502900	-2.31326500	-0.12301400
C	0.23149200	-1.28355000	0.45665200
C	-0.13507400	0.16066000	0.00769000
O	1.53508000	-1.60080900	-0.20421800
C	2.49562100	-0.68388500	-0.15404800
C	2.31438800	0.62053600	0.25011400
C	0.92553100	1.13062100	0.56330200
O	3.65213400	-1.22167000	-0.55310000
C	4.71229200	-0.26759100	-0.92217100
C	4.82367400	0.80251300	0.15192700
C	3.47610700	1.43532400	0.45487200
O	3.41555600	2.60032400	0.91209700
O	-1.13925600	2.57400500	-1.08763100
C	-1.94033100	0.93667400	1.70729200
C	0.46136700	-1.51262500	1.95369200
C	5.96961200	-1.08330900	-1.13744400
H	-2.29114400	-0.61783100	-1.41660000
C	-4.81711700	-0.68575200	0.97889100
C	-4.76652700	-1.09903400	-1.48569000
H	-0.00727500	0.14195900	-1.08337400
H	-3.66662700	3.11093200	-1.09574500
H	-5.40762900	1.42778000	-0.78615800
H	-2.33748900	-1.98445000	1.30697000
H	-2.84047400	-2.72366100	-0.20170400
H	-0.61660200	-2.32813400	-1.21338200
H	-0.47600000	-3.31037600	0.25099200
H	0.79746000	2.11707700	0.11620400
H	0.81368400	1.26411800	1.64578000
H	4.38044400	0.18692000	-1.86415000
H	5.21926300	0.37133000	1.08282000
H	5.52039500	1.58011000	-0.17486600
H	-1.88289200	0.06615100	2.36317600
H	-1.22561800	1.67994700	2.07122500
H	-2.93491400	1.37173200	1.81450700
H	1.15558500	-0.79029100	2.38867500
H	-0.47724400	-1.45656200	2.50804400
H	0.87537400	-2.51578100	2.09742200
H	6.28749300	-1.56694900	-0.20765700
H	5.80737400	-1.85091100	-1.90075700
H	6.77405700	-0.42450600	-1.47981100
H	-5.87630500	-0.41119300	0.91560500
H	-4.39603700	-0.19308500	1.85723700
H	-4.76648500	-1.76774400	1.13611500
H	-4.34568600	-0.82685800	-2.45987400
H	-4.63018300	-2.17577400	-1.34153600
H	-5.84396300	-0.90128100	-1.51239900

Table S7. Optimized cartesian coordinates of compound **2** for ECD calculation at the b3lyp/6-31+g(d,p) level.

Atom	Coordinates		
C	-3.55389900	2.19885100	-0.75812000
C	-4.54334700	1.29730700	-0.63455600
C	-4.34557300	-0.17027700	-0.35906800
C	-2.81962400	-0.52404100	-0.36190400
C	-1.85601500	0.56534000	0.24972500
C	-2.13936900	1.84724400	-0.55993100
C	-2.51430900	-1.92789000	0.18450200
C	-1.07832600	-2.34060900	-0.15278000
C	-0.07442800	-1.36464200	0.45453100
C	-0.37159200	0.10112300	0.02602900
O	1.22313200	-1.73188900	-0.20095200
C	2.21882400	-0.86171400	-0.13314600
C	2.09238400	0.44313500	0.29440100
C	0.72721600	1.01608500	0.60117200
O	3.35250600	-1.43594500	-0.55003000
C	4.46190100	-0.54069100	-0.90217200
C	4.61453300	0.47995400	0.22212700
C	3.28513700	1.20607500	0.45195200
O	3.32472600	2.40863300	0.79512900
O	-1.25650200	2.58417000	-1.01416000
C	-2.15403500	0.91864300	1.73190800
C	0.13400700	-1.62649700	1.94839300
C	5.67243400	-1.41905200	-1.13199800
C	-5.09731000	-0.54633500	0.94363400
C	-5.03982000	-0.92391200	-1.52653400
H	-0.23906200	0.09426200	-1.06457900
O	5.63499500	1.40879000	-0.10691800
H	-3.75641900	3.22761400	-1.04714000
H	-5.57342400	1.61721100	-0.79333500
H	-2.54591600	-0.55147700	-1.42743500
H	-2.68040400	-1.97164200	1.26638500
H	-3.19843100	-2.65539200	-0.26315200
H	-0.86758500	-3.35412800	0.20683400
H	-0.94809800	-2.34242800	-1.24185600
H	0.61339100	1.14339500	1.68390900
H	0.65053000	2.01083700	0.16130700
H	4.16724800	-0.02301500	-1.82387500
H	4.87021300	-0.04976000	1.15517800
H	-2.15848400	0.03000700	2.36567200
H	-1.40127800	1.60838400	2.12332700
H	-3.12204100	1.40981200	1.83961200
H	0.50451700	-2.64810300	2.07989600
H	0.85382100	-0.93993500	2.39938800
H	-0.80626500	-1.53949600	2.49553400
H	5.46732500	-2.14954500	-1.92071100
H	6.51695100	-0.80082700	-1.44959700
H	5.95002900	-1.95021000	-0.21534000
H	-5.08710800	-1.63068100	1.09252800
H	-6.14513300	-0.23391300	0.86822100
H	-4.67209700	-0.07497800	1.83165500
H	-4.59309600	-0.66079400	-2.49164000
H	-4.95929500	-2.00775900	-1.39500900
H	-6.10554400	-0.67213800	-1.56396800
H	5.32206700	2.27604400	0.21464000

Table S8. Optimized cartesian coordinates of compound **3** for ECD calculation at the b3lyp/6-31+g(d,p) level.

Atom		Coordinates	
O	-3.56110800	2.72149100	0.65671800
O	2.10142500	-0.50214200	-1.71801800
H	2.46222100	-1.28675000	-2.15577000
O	-1.69104500	-1.58286500	-0.17000300
O	-3.81757300	-1.17661000	-0.49070600
O	1.02883800	2.61610100	-1.00048100
C	-3.62354200	1.51379900	0.29552100
C	1.85737900	1.85048200	-0.49948000
C	-2.63452000	-0.64635400	-0.11045600
C	1.48142100	0.57694300	0.29035200
C	-2.46123700	0.65598000	0.26004900
C	-0.02007100	0.18112800	0.04865900
H	-0.14389900	0.16896800	-1.03895700
C	-4.87668900	0.90598300	-0.12221400
H	-5.77638900	1.51120600	-0.13226000
C	0.57911100	-2.29612100	-0.08072300
H	0.43989600	-2.33804600	-1.16503900
H	0.34081000	-3.28752000	0.31991700
C	2.42138100	-0.55324000	-0.30272600
C	-4.94227600	-0.38910900	-0.50599200
C	-1.07952500	1.15996200	0.59569900
H	-0.99166300	1.29808100	1.67943200
H	-0.94571200	2.14492500	0.14677300
C	2.03357700	-1.93735400	0.24359600
H	2.68956100	-2.69677200	-0.19530900
H	2.20352500	-1.96709500	1.32340500
C	4.23534200	1.21487000	-0.39233900
H	5.28597800	1.49402000	-0.47135100
C	1.75243700	0.92048800	1.78038300
H	2.73201300	1.37480400	1.92355100
H	1.69039300	0.03998500	2.42068300
H	1.01421200	1.64441800	2.13384900
C	-0.38941600	-1.26552000	0.49519800
C	3.96794900	-0.25050100	-0.14629300
C	3.29485200	2.15488000	-0.58454700
H	3.55740500	3.17664100	-0.84807100
C	4.78965800	-1.01992400	-1.21948800
H	4.60328100	-2.09762500	-1.16459800
H	5.85708600	-0.86090400	-1.03531300
H	4.57529900	-0.67755800	-2.23532800
C	4.56508200	-0.66486800	1.22542000
H	4.03575000	-0.24564900	2.08051500
H	5.60556100	-0.32624600	1.27964000
H	4.57053400	-1.75466000	1.32491400
C	-0.62852200	-1.49817500	1.99073000
H	-1.30318700	-0.76120300	2.43228600
H	0.31011200	-1.47449600	2.54726600
H	-1.06966300	-2.49094100	2.12461600
C	-6.13942300	-1.14258300	-0.96938800
H	-5.98032300	-1.52458500	-1.98453900
H	-7.01701900	-0.49360400	-0.96685300
H	-6.32788400	-2.00180500	-0.31532300

Table S9. Optimized cartesian coordinates of compound **4** for ECD calculation at the b3lyp/6-31+g(d,p) level.

Atom		Coordinates	
C	-3.25661900	2.02558600	0.27255900
C	-4.08180800	1.03960700	-0.55392100
C	-3.79297700	-0.45165900	-0.21090900
C	-2.24284600	-0.69269800	-0.23358800
C	-1.36376800	0.31155400	0.59760700
C	-1.74215500	1.77417300	0.18764700
C	-1.82236300	-2.14996300	0.03503100
C	-0.35387300	-2.38556100	-0.34951500
C	0.59166200	-1.42705500	0.37617700
C	0.13031500	0.04060500	0.18673500
O	1.88456600	-1.56957100	-0.37435500
C	2.77936400	-0.59886800	-0.22523300
C	2.55932400	0.63532800	0.31605600
C	1.17149200	1.01676900	0.76532900
O	3.97227700	-1.00915000	-0.71234700
C	5.05398900	-0.16473900	-0.66749600
C	4.93919000	1.06703400	-0.12138100
C	3.67551500	1.54625400	0.41398400
O	3.56633100	2.69525900	0.92606800
C	-1.55810400	0.20845500	2.13352900
C	0.90166100	-1.89159800	1.80111600
C	6.26786600	-0.78662000	-1.26361800
H	-1.96410900	-0.49908000	-1.27951700
C	-4.48407400	-0.78659800	1.13104300
C	-4.46872300	-1.34168700	-1.28371200
H	0.15396500	0.18484800	-0.89930400
O	-1.25768100	2.13293600	-1.12484400
O	-3.78737000	1.35686700	-1.94191600
H	-3.58591300	1.98073900	1.31304700
H	-3.46333200	3.04453000	-0.07449900
H	-5.15104300	1.22808100	-0.38626800
H	-1.23878600	2.46469800	0.87238900
H	-1.98449300	-2.42251200	1.08402700
H	-2.43522800	-2.83563200	-0.55706700
H	-0.05942000	-3.42083800	-0.14012600
H	-0.23717800	-2.22323000	-1.42805100
H	1.13738100	1.05793700	1.86058000
H	0.96656100	2.03290600	0.41529100
H	5.80567000	1.71814200	-0.08645700
H	-2.40857200	0.79786900	2.47918100
H	-1.72610200	-0.81630200	2.46784400
H	-0.68289500	0.58886300	2.66720300
H	1.43323600	-2.84745500	1.75319800
H	1.52335800	-1.17932500	2.34992100
H	-0.01597900	-2.05049900	2.36965800
H	6.52550300	-1.71006400	-0.73207500
H	6.08793400	-1.04517000	-2.31364800
H	7.11102700	-0.09570600	-1.20686200
H	-4.23467800	-1.80246300	1.45418300
H	-5.57279300	-0.74289500	1.00460800
H	-4.22353300	-0.10481300	1.94199000
H	-4.53686100	-2.38140500	-0.95004200
H	-5.49502600	-1.00009500	-1.47096200
H	-3.92592300	-1.33946900	-2.23591300
H	-1.92255400	1.83767500	-1.77280500
H	-4.32359600	0.80001800	-2.52499900

Table S10. Optimized cartesian coordinates of compound **5** for ECD calculation at the b3lyp/6-31+g(d,p) level.

Atom	Coordinates		
C	-3.00502400	-0.97757800	-1.36665200
C	-3.85637900	-0.24442700	-0.31407700
C	-3.31447000	1.17049400	0.05147200
C	-1.75815600	1.14507700	0.25530600
C	-0.88250500	0.47053700	-0.88246300
C	-1.48992000	-0.91315500	-1.15190600
C	-1.15665900	2.50210800	0.66531500
C	0.24589800	2.30668500	1.24993500
C	1.18385800	1.64340800	0.24401800
C	0.58481100	0.32176500	-0.32082300
O	2.36227600	1.24563100	1.07169700
C	3.21792200	0.37027500	0.55502600
C	2.98005300	-0.38168300	-0.57330300
C	1.62770900	-0.33438200	-1.24869400
O	4.33062600	0.33766600	1.29359500
C	5.20063600	-0.83389200	1.09354900
C	5.39660100	-1.06706200	-0.39601400
C	4.06939800	-1.12711500	-1.13383900
C	-3.99799300	1.61620900	1.36677600
C	-3.79820700	2.14734700	-1.04937800
O	-0.85435600	-1.95053700	-1.30294700
O	3.97312900	-1.76287600	-2.20912000
C	6.47418100	-0.56037900	1.86562200
C	-0.91472600	1.22857700	-2.24591700
C	1.71669600	2.65387100	-0.77656200
O	-3.43354900	-2.31711900	-1.53109600
O	-4.19206500	-1.08231400	0.84195000
C	-3.31151700	-1.80027800	1.55320800
O	-2.10377000	-1.78980400	1.35835300
C	-4.00092000	-2.61775300	2.60858400
H	-3.18844500	-0.43266100	-2.30317100
H	-4.85298300	-0.11722900	-0.74214500
H	-1.61168800	0.49199100	1.12149400
H	-1.12670200	3.20440500	-0.17478800
H	-1.77947500	2.96755100	1.43308100
H	0.17817900	1.66337500	2.13555800
H	0.67258400	3.26405300	1.57013700
H	0.48487500	-0.33094700	0.55644800
H	1.71006600	0.20002400	-2.20258200
H	1.32041100	-1.35053300	-1.49409400
H	4.65641100	-1.67954100	1.53235500
H	5.99791900	-0.25763300	-0.83451400
H	5.93810800	-2.00448300	-0.55358900
H	-3.86923100	2.69027200	1.52512700
H	-5.07492300	1.42249600	1.31734200
H	-3.59824100	1.09186000	2.24041600
H	-4.88665300	2.25763200	-0.98662200
H	-3.35720300	3.13784300	-0.90506600
H	-3.56201200	1.82066800	-2.06367800
H	7.00581800	0.30280900	1.45130000
H	6.25325900	-0.36982700	2.92064100
H	7.12991900	-1.43491700	1.80571600
H	-1.79362200	0.98131700	-2.84261900
H	-0.92032000	2.30919500	-2.09982700
H	-0.04315000	0.97080900	-2.85079300
H	0.90115300	3.11655100	-1.33443900
H	2.24878800	3.44677300	-0.24134800
H	2.40662900	2.20427200	-1.49402300
H	-2.62374900	-2.86316800	-1.54567600
H	-4.64258900	-1.97769200	3.22187200
H	-4.63953800	-3.36702600	2.12821200
H	-3.25964400	-3.11532400	3.23472200

Table S11. Optimized cartesian coordinates of compound **6** for ECD calculation at the b3lyp/6-31+g(d,p) level.

Atom	Coordinates		
C	-3.33143600	2.21772000	-0.37883700
C	-4.28606000	1.28318500	-0.23508700
C	-4.04394700	-0.20345800	-0.11560100
C	-2.51330600	-0.51939800	-0.34167700
C	-1.53438300	0.54400900	0.32552400
C	-1.89926600	1.87684700	-0.36185000
C	-2.13697000	-1.94980700	0.07826800
C	-0.68879800	-2.30613000	-0.28084200
C	0.30472100	-1.34311600	0.37011200
C	-0.04184200	0.13868200	0.03734400
O	1.59816000	-1.63979300	-0.30099100
C	2.57983200	-0.72786500	-0.22875300
C	2.40476800	0.53875700	0.23923200
C	1.04253500	1.05401700	0.64216900
C	3.89868500	-1.20479800	-0.75612200
C	5.02359300	-0.46255900	-0.04922800
O	4.77429300	0.98262800	-0.08577000
C	3.53540900	1.45374300	0.21705300
O	3.44091200	2.66726500	0.43314800
O	-1.06693600	2.65355000	-0.83922600
C	-1.77087400	0.77799900	1.84289900
C	0.52842000	-1.69117900	1.84691100
C	6.39252300	-0.68318100	-0.66067200
O	-2.35780400	-0.38576100	-1.78073100
C	-4.60369400	-0.71554000	1.23874200
C	-4.91835300	-0.87372000	-1.21421500
H	0.09345200	0.21464800	-1.05001600
H	-3.57856800	3.26220900	-0.55298100
H	-5.33226800	1.58738200	-0.25925600
H	-2.29021700	-2.06574900	1.15498300
H	-2.81009900	-2.65642300	-0.41624200
H	-0.46967600	-3.33100300	0.03789600
H	-0.54389400	-2.28074000	-1.36785000
H	0.96705900	1.10557000	1.73403900
H	0.91739800	2.07134500	0.27142900
H	3.94366800	-1.04047800	-1.84171500
H	4.00446800	-2.27913100	-0.57740000
H	5.03126500	-0.74139700	1.01166300
H	-1.72294900	-0.15177900	2.40982800
H	-1.00926900	1.45349100	2.23987300
H	-2.73600900	1.24363600	2.03790300
H	0.95577100	-2.69726900	1.90564000
H	1.21298200	-0.99933700	2.34269900
H	-0.41034700	-1.69664300	2.40285400
H	7.15327700	-0.11616200	-0.11513000
H	6.40613000	-0.37552400	-1.71168600
H	6.65091600	-1.74574600	-0.60453400
H	-1.43735700	-0.53008800	-2.03800300
H	-4.65270600	-1.80870500	1.24018000
H	-5.62595800	-0.34292900	1.36482600
H	-4.02604600	-0.39827900	2.10586400
H	-4.73089000	-0.46130100	-2.20710700
H	-4.74389100	-1.95352600	-1.24922100
H	-5.97459200	-0.71442700	-0.97219900

Table S12. Optimized cartesian coordinates of compound **10** for ECD calculation at the b3lyp/6-31+g(d,p) level.

atom	C1 30.98%			C2 49.41%			C3 19.61%		
C	-4.821579	-3.943434	0.179633	-4.82158	-3.94343	0.179633	-4.981854	-3.825651	0.254934
C	-5.537427	-2.626434	-0.121987	-5.53743	-2.62643	-0.12199	-5.648723	-2.498341	-0.082101
C	-4.843615	-1.913521	-1.28189	-4.84362	-1.91352	-1.28189	-4.918792	-1.835918	-1.258335
C	-3.356227	-1.713973	-1.057116	-3.35623	-1.71397	-1.05712	-3.428083	-1.673128	-1.027453
C	-2.551159	-2.930947	-0.584398	-2.55116	-2.93095	-0.5844	-2.669569	-2.905402	-0.519721
C	-3.345497	-3.693984	0.514611	-3.3455	-3.69398	0.514611	-3.499834	-3.615312	0.589285
C	-2.800566	-0.52951	-1.354127	-2.80057	-0.52951	-1.35413	-2.833443	-0.512192	-1.341343
C	-1.319607	-0.254237	-1.374209	-1.31961	-0.25424	-1.37421	-1.343919	-0.286727	-1.356148
C	-0.510838	-1.410651	-0.826829	-0.51084	-1.41065	-0.82683	-0.578821	-1.457569	-0.777087
C	-1.207131	-2.434874	0.03752	-1.20713	-2.43487	0.03752	-1.31502	-2.439314	0.10296
C	0.81635	-1.330719	-0.949179	0.81635	-1.33072	-0.94918	0.750845	-1.426002	-0.893263
C	1.839895	-2.251655	-0.304131	1.839895	-2.25166	-0.30413	1.738832	-2.367221	-0.222681
C	1.151004	-2.981197	0.87227	1.151004	-2.9812	0.87227	1.019245	-3.047147	0.964908
C	-0.229021	-3.541733	0.476543	-0.22902	-3.54173	0.476543	-0.37744	-3.568222	0.573842
C	1.462374	-0.111437	-1.558756	1.462374	-0.11144	-1.55876	1.441438	-0.243786	-1.526013
C	2.933972	-0.193629	-1.077926	2.933972	-0.19363	-1.07793	2.906746	-0.365621	-1.034959
C	2.941338	-1.202887	0.101661	2.941338	-1.20289	0.101661	2.873338	-1.347984	0.166844
C	2.38387	-3.270809	-1.334666	2.38387	-3.27081	-1.33467	2.252893	-3.425105	-1.228952
C	-2.306658	-3.850641	-1.806823	-2.30666	-3.85064	-1.80682	-2.445059	-3.859915	-1.719148
O	-6.897033	-2.822267	-0.511152	-6.89703	-2.82227	-0.51115	-7.014755	-2.767981	-0.395734
C	4.369971	-1.703965	0.437102	4.369971	-1.70397	0.437102	4.283225	-1.88701	0.52242
C	5.287924	-0.529648	0.697555	5.287924	-0.52965	0.697555	5.23395	-0.73509	0.763267
C	4.415731	-2.657156	1.649481	4.415731	-2.65716	1.649481	4.292981	-2.8141	1.755391
C	6.410553	-0.25552	0.025512	6.410553	-0.25552	0.025512	6.369201	-0.508137	0.095657
C	7.332162	0.911973	0.289378	7.332162	0.911973	0.289378	7.32274	0.63435	0.358293
C	7.481708	1.773152	-0.980431	7.481708	1.773152	-0.98043	7.538911	1.452338	-0.930736
C	8.717883	0.486029	0.867777	8.717883	0.486029	0.867777	8.649012	0.143225	1.021352
C	8.582423	-0.07178	2.291677	8.582423	-0.07178	2.291677	9.485231	-0.786284	0.127407
C	9.487923	-0.499323	-0.026339	9.487923	-0.49932	-0.02634	9.503148	1.315104	1.530728
O	-2.341291	1.986411	-3.350778	-2.34129	1.986411	-3.35078	-2.270863	1.932151	-3.397173
C	-6.107772	1.965711	3.032655	-6.10777	1.965711	3.032655	-6.062891	2.274076	2.976374
O	-1.553383	0.286772	3.541128	-1.55338	0.286772	3.541128	-1.599172	0.389501	3.543385
O	0.065491	0.439886	1.504101	0.065491	0.439886	1.504101	0.037885	0.42684	1.515399
C	-2.463998	0.879748	2.753244	-2.464	0.879748	2.753244	-2.479328	0.999462	2.734776
C	-3.746736	1.107853	3.264587	-3.74674	1.107853	3.264587	-3.753564	1.295035	3.232066
C	-4.726647	1.713122	2.477012	-4.72665	1.713122	2.477012	-4.702021	1.923155	2.423973
C	-4.426878	2.109322	1.16209	-4.42688	2.109322	1.16209	-4.380592	2.267783	1.099851
C	-0.858915	0.915245	0.827215	-0.85892	0.915245	0.827215	-0.861932	0.920916	0.819566
C	-2.152096	1.247338	1.415121	-2.1521	1.247338	1.415121	-2.145432	1.318116	1.389024
C	0.741386	1.24185	-1.160806	0.741386	1.24185	-1.16081	0.765655	1.14199	-1.162208
C	-0.745152	1.093324	-0.692333	-0.74515	1.093324	-0.69233	-0.729424	1.056695	-0.70285
C	-1.541037	2.394744	-1.018893	-1.54104	2.394744	-1.01889	-1.474599	2.376844	-1.070406

C	-1.518774	2.797757	-2.516479	-1.51877	2.797757	-2.51648	-1.428173	2.738194	-2.577499
C	-0.084253	2.842657	-2.968825	-0.08425	2.842657	-2.96883	0.010864	2.724197	-3.017488
C	0.891255	2.17724	-2.337858	0.891255	2.17724	-2.33786	0.957891	2.044106	-2.359233
H	-1.505434	-1.894168	0.951997	-1.50543	-1.89417	0.951997	-1.602745	-1.869268	1.002851
H	1.298603	1.658441	-0.317142	1.298603	1.658441	-0.31714	1.331131	1.556776	-0.323203
H	1.424375	-0.178998	-2.655674	1.424375	-0.179	-2.65567	1.406303	-0.334685	-2.621377
H	-1.043929	-0.130144	-2.433591	-1.04393	-0.13014	-2.43359	-1.057704	-0.197597	-2.416339
O	-2.918444	2.294666	-0.630651	-2.91844	2.294666	-0.63065	-2.857798	2.338267	-0.689695
C	-3.159103	1.881824	0.649249	-3.1591	1.881824	0.649249	-3.120852	1.972727	0.600446
H	2.551283	-0.678911	0.985647	2.551283	-0.67891	0.985647	2.496518	-0.791263	1.036543
C	-0.947375	3.599186	-0.215976	-0.94738	3.599186	-0.21598	-0.84248	3.57946	-0.294774
O	0.013874	3.562828	0.52076	0.013874	3.562828	0.52076	0.103808	3.524575	0.45988
O	-1.674655	4.709582	-0.427612	-1.67466	4.709582	-0.42761	-1.517715	4.712916	-0.55201
C	-1.24659	5.875766	0.299898	-1.24659	5.875766	0.299898	-1.049551	5.881986	0.145794
H	-4.928142	-4.603585	-0.691282	-4.92814	-4.60359	-0.69128	-5.105424	-4.503268	-0.600223
H	-5.307482	-4.454254	1.023979	-5.30748	-4.45425	1.023979	-5.497954	-4.291604	1.102736
H	-5.489161	-1.980373	0.772079	-5.48916	-1.98037	0.772079	-5.586222	-1.834173	0.797453
H	-5.013772	-2.530961	-2.177403	-5.01377	-2.53096	-2.1774	-5.100348	-2.473	-2.137525
H	-5.331005	-0.951449	-1.470514	-5.33101	-0.95145	-1.47051	-5.364934	-0.858006	-1.482856
H	-3.297093	-3.109197	1.444534	-3.29709	-3.1092	1.444534	-3.435697	-3.012301	1.506407
H	-2.848674	-4.648652	0.724167	-2.84867	-4.64865	0.724167	-3.037571	-4.581622	0.822864
H	-3.435352	0.265956	-1.728397	-3.43535	0.265956	-1.7284	-3.438833	0.295807	-1.737437
H	1.773409	-3.803567	1.242592	1.773409	-3.80357	1.242592	1.611466	-3.882063	1.356078
H	1.025875	-2.274573	1.702434	1.025875	-2.27457	1.702434	0.914526	-2.318887	1.779024
H	-0.649197	-4.088672	1.328482	-0.6492	-4.08867	1.328482	-0.821189	-4.079587	1.435803
H	-0.100217	-4.275693	-0.327109	-0.10022	-4.27569	-0.32711	-0.27023	-4.325961	-0.210703
H	3.58272	-0.558778	-1.884067	3.58272	-0.55878	-1.88407	3.546663	-0.770916	-1.828951
H	3.334517	0.780448	-0.780611	3.334517	0.780448	-0.78061	3.339601	0.600282	-0.757049
H	1.578338	-3.900311	-1.725782	1.578338	-3.90031	-1.72578	1.428584	-4.03527	-1.61123
H	2.851178	-2.7747	-2.192575	2.851178	-2.7747	-2.19258	2.740193	-2.962222	-2.094271
H	3.1332	-3.929977	-0.88091	3.1332	-3.92998	-0.88091	2.978184	-4.099034	-0.75821
H	-1.618781	-3.374988	-2.514499	-1.61878	-3.37499	-2.5145	-1.736399	-3.423369	-2.431697
H	-1.877345	-4.813255	-1.507386	-1.87735	-4.81326	-1.50739	-2.049695	-4.828445	-1.393822
H	-3.236995	-4.063785	-2.342522	-3.237	-4.06379	-2.34252	-3.37755	-4.056382	-2.257473
H	-7.343613	-3.285016	0.215185	-7.34361	-3.28502	0.215185	-7.434439	-1.923558	-0.623869
H	4.763105	-2.242726	-0.436495	4.763105	-2.24273	-0.4365	4.664621	-2.456524	-0.336797
H	4.991687	0.12495	1.522176	4.991687	0.12495	1.522176	4.950549	-0.054017	1.571079
H	5.452729	-2.916872	1.889156	5.452729	-2.91687	1.889156	5.320521	-3.098442	2.007436
H	3.872877	-3.587706	1.460265	3.872877	-3.58771	1.460265	3.72366	-3.731973	1.582661
H	3.974961	-2.187118	2.537394	3.974961	-2.18712	2.537394	3.861046	-2.311714	2.629849
H	6.694635	-0.910983	-0.801294	6.694635	-0.91098	-0.80129	6.643068	-1.187883	-0.714738
H	6.859765	1.54096	1.057967	6.859765	1.54096	1.057967	6.843942	1.296525	1.094502
H	8.146934	2.626906	-0.801472	8.146934	2.626906	-0.80147	8.203061	2.307646	-0.76568
H	6.508804	2.161917	-1.300728	6.508804	2.161917	-1.30073	6.581939	1.8391	-1.297895
H	7.895624	1.19675	-1.816388	7.895624	1.19675	-1.81639	7.972798	0.840017	-1.730159

H	9.314168	1.40949	0.930138	9.314168	1.40949	0.930138	8.338527	-0.44056	1.899911
H	9.565923	-0.308002	2.715292	9.565923	-0.308	2.715292	10.36395	-1.150763	0.672519
H	8.095899	0.650739	2.958366	8.095899	0.650739	2.958366	8.914329	-1.662256	-0.199382
H	7.982359	-0.989144	2.302164	7.982359	-0.98914	2.302164	9.849632	-0.267695	-0.767569
H	10.487112	-0.68651	0.384229	10.48711	-0.68651	0.384229	10.353125	0.946654	2.117449
H	9.618165	-0.119744	-1.045877	9.618165	-0.11974	-1.04588	8.920491	1.98714	2.172703
H	8.97441	-1.466116	-0.089615	8.97441	-1.46612	-0.08962	9.911947	1.910432	0.705616
H	-1.809953	1.232232	-3.648661	-1.80995	1.232232	-3.64866	-1.763078	1.151678	-3.667011
H	-6.197568	1.613009	4.064056	-6.19757	1.613009	4.064056	-6.258975	1.758133	3.920752
H	-6.347984	3.035909	3.017955	-6.34798	3.035909	3.017955	-6.141231	3.352975	3.163332
H	-6.871693	1.458455	2.430799	-6.87169	1.458455	2.430799	-6.858597	2.012489	2.269716
H	-0.711864	0.239046	3.015708	-0.71186	0.239046	3.015708	-0.757407	0.292051	3.025477
H	-3.953872	0.807016	4.286534	-3.95387	0.807016	4.286534	-3.978887	1.028436	4.25966
H	-5.164569	2.606328	0.540454	-5.16457	2.606328	0.540454	-5.092874	2.780119	0.461148
H	-1.972562	3.78995	-2.577331	-1.97256	3.78995	-2.57733	-1.848054	3.742802	-2.668955
H	0.136476	3.440754	-3.850267	0.136476	3.440754	-3.85027	0.259553	3.292384	-3.911164
H	1.909148	2.259308	-2.719633	1.909148	2.259308	-2.71963	1.981704	2.08348	-2.731811
H	-1.29081	5.688036	1.375416	-1.29081	5.688036	1.375416	-1.119469	5.73155	1.225795
H	-1.942799	6.664922	0.016345	-1.9428	6.664922	0.016345	-1.704785	6.691757	-0.174869
H	-0.223299	6.143321	0.024463	-0.2233	6.143321	0.024463	-0.010949	6.09442	-0.120056

Details for Biological Assay

1. Cell culture

HK-2 cell lines were maintained in MEM (with NEAA, Procell) supplemented with 10% fetal bovine serum (FBS, Lonsera), 100 U/mL penicillin, and 100 μg/mL streptomycin (Procell) at 37 °C in a humidified 5% CO_2 incubator (STIK). MCF-7 cell lines were maintained in MEM (Procell) supplemented with 10% fetal bovine serum (FBS, Lonsera), 0.01 mg/mL Recombinant Human Insulin Solution (Procell), 100 U/mL penicillin, and 100 μg/mL streptomycin (Procell) at 37 °C in a humidified 5% CO_2 incubator (STIK).

2. Ferroptosis inhibition assay

The ferroptosis inhibitory activity of compounds **1**–**17** was evaluated in RSL3-induced HK-2 cells using the CCK-8 assay, except for compounds **2**, **6**, **9**, **12**, and **14** (which were not tested due to insufficient sample amounts). HK-2 cells in logarithmic phase were seeded as described in 96-well plates. At 70–80% confluence, cells were pretreated for 2 h with compounds, the ferroptosis inhibitor Ferrostatin-1 (Fer-1, 1 μM, positive control), or DMSO (vehicle control in blank and ferroptosis groups). Ferroptosis was then induced by adding 0.4 μM RSL3 to all groups except the blank control, followed by 24 h co-incubation. To further characterize compound **15**, dose-dependent assays (5–30 μM) were performed. Post-treatment viability was determined by CCK8 assay as describe above.

3. GSH and MDA assays

Cells were cultured in 6-well plates until reaching 70–80 % confluency. Experimental treatments included: control with culture medium, RSL3 exposure (0.4 μM), and pretreatment with compound **15** (7.5, 15 or 20 μM) for 2 h followed by co-administration of 0.4 μM RSL3. After 24-hour incubation, cells were harvested for subsequent quantification of GSH and malondialdehyde (MDA) levels using commercial assay kits (Nanjing Jiancheng; Cat. No. A006-2-1 for GSH, Beyotime; Cat. No. S0131S for MDA) according to manufacturer′s protocols.

4. Lipid peroxidation analysis

Lipid peroxidation was assessed using C11 BODIPY™ 581/591. HK-2 cells (1.5×10^5 cells/well) in 6-well plates were cultured for 24 h. Post-treatment, cells were incubated with 1 mL of probe solution (2 μM final concentration) for 30 min at 37 °C. Peroxidation levels were visualized using inverted fluorescence microscopy (OLYMPUS, Japan, model CKX53).

5. Cytotoxicity assay

The cytotoxicity of compounds against MCF-7 cell lines was evaluated using the CCK-8 assay. Cells in the logarithmic growth phase were harvested and prepared as single-cell suspensions in complete DMEM. Subsequently, the cells were seeded into 96-well plates at 7.0×10^4 cells/mL (100 μL/well) and cultured until 70–80 % confluence. The tested compounds above (10 μM) or DMSO (negative control), were added to triplicate wells. After 24 h incubation, 10 μL of CCK-8 reagent was added to each well, followed by incubation at 37 ˚C for 3 h. To further characterize compounds **11** and **16**, dose-dependent assays (0.3–20 μM) were performed. The absorbance at 450 nm was recorded on a Synergy H1 microplate reader (BioTek Instruments, Inc.) and used to calculate cell viability.

6. Statistical analysis

All experiments were performed in triplicate. Data were processed using GraphPad Prism 8.0 (GraphPad Software Inc., San Diego, CA, USA) and expressed as mean ± SD. Statistical significance was determined by

unpaired *t*-tests ($^{\#}p < 0.05$, $^{\#\#}p < 0.01$, $^{\#\#\#}p < 0.001$, $^{\#\#\#\#}p < 0.0001$ vs. Control; $^{*}p < 0.05$, $^{**}p < 0.01$, $^{***}p < 0.001$, $^{****}p < 0.0001$ vs. RSL3-treated groups).
